# Design, eco-friendly synthesis, molecular modeling and anticancer evaluation of thiazol-5(4*H*)-ones as potential tubulin polymerization inhibitors targeting the colchicine binding site†

**DOI:** 10.1039/c9ra10094f

**Published:** 2020-01-15

**Authors:** Abeer M. El-Naggar, Ibrahim H. Eissa, Amany Belal, Amira A. El-Sayed

**Affiliations:** Chemistry Department, Faculty of Science, Ain Shams University Abbassiya Cairo 11566 Egypt elsayedam@sci.asu.edu.eg; Medicinal Chemistry Department, Faculty of Pharmacy, Beni-Suef University Beni-Suef 62415 Egypt; Pharmaceutical Medicinal Chemistry & Drug Design Department, Faculty of Pharmacy (Boys), Al-Azhar University Cairo 11884 Egypt Ibrahimeissa@azhar.edu.eg

## Abstract

In recent years, suppressing tubulin polymerization has been developed as a therapeutic approach for cancer treatment. Thus, new derivatives based on thiazol-5(4*H*)-ones have been designed and synthesized in an eco-friendly manner. The synthesized derivatives have the same essential pharmacophoric features of colchicine binding site inhibitors. The anti-proliferative activity of the new derivatives was evaluated on three human cancer cell lines (HCT-116, HepG-2, and MCF-7) using MTT assay procedure and colchicine was used as a positive control. Compounds 4f, 5a, 8f, 8g, and 8k showed superior antiproliferative activities against the three tested cell lines with IC_50_ values ranging from 2.89 to 9.29 μM. Further investigation for the most active cytotoxic agents as tubulin polymerization inhibitors was also performed in order to explore the mechanism of their anti-proliferative activity. Tubulin polymerization assay results were found to be comperable with the cytotoxicity results. Compounds 4f and 5a were the most potent tubulin polymerization inhibitors with an IC_50_ value of 9.33 and 9.52 nM, respectively. Further studies revealed the ability of 5a to induce apoptosis and arrest cell cycle growth at the G2/M phase. Molecular docking studies were also conducted to investigate possible binding interactions between the target compounds and the tubulin heterodimer active site. From these studies, it was concluded that inhibition of tubulin polymerization yields the reported cytotoxic activity.

## Abstract 2

In recent years, suppressing tubulin polymerization has been developed as a therapeutic approach for cancer treatment.

## Graphical abstract

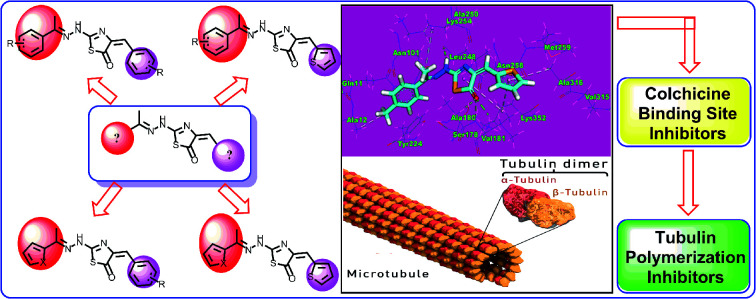

## 1. Introduction

Cancer is a complex, widespread, and lethal disease. It begins when cells start to grow beyond their usual boundaries, then can invade adjoining parts of the body and spread to other organs.^1^ Many of the currently available antitumor drugs are unable to differentiate between normal and neoplastic cells, and are also unable to overcome primary or secondary resistance mechanisms evolved in the tumor cells.^2,3^ Thus, there is a pressing need for new antitumor agents with high potency, and less toxicity in non-cancerous cells, that are able to act on unique targets.

Microtubules, the key components of the cytoskeleton are essential in all eukaryotic cells. Microtubules are composed of α-tubulin and β-tubulin heterodimers arranged in the form of slender filamentous tubes that can be many micrometres long.^4^ They are highly dynamic polymers and their polymerization dynamics are tightly regulated both spatially and temporally.^5^ They are crucial in the development and maintenance of cell shape and cell division (mitosis).^6^ During mitosis process, the duplicated chromosomes of a cell are separated into two identical sets before cleavage of the cell into two daughter cells.^7^

The importance of microtubules in mitosis and cell division makes it an important target for anticancer drugs.^8^ Microtubules and their dynamics are considered targets for diverse groups of antimitotic drugs (with various tubulin-binding sites) that have been used with great success in the treatment of cancer.^9^ In view of the success of this class of drugs, it has been argued that microtubules represent the best cancer target to be identified so far, and it seems likely that drugs of this class will continue to be important chemotherapeutic agents, even as more selective approaches are developed^10^

The tubulin heterodimer contains at least three distinct drug binding sites: the paclitaxel (taxanes alkaloid), vinblastine (vinca alkaloid), and colchicine binding sites.^11^ For the first two of these sites, there are many drugs in current use in clinical oncology.^12,13^ All the marketed tubulin inhibitors bind to the paclitaxel and vinblastine binding sites are highly potent but the clinical use is limited for several reasons: (i) they are prone to develop multi-drug resistance, (ii) they are highly lipophilic and have to be solubilized by surfactants which can cause hypersensitivity reactions in patients, (iii) they have to be administered intravenously due to poor water solubility which is not convenient for patients and may lead to poor patient compliance.^14^

Tubulin inhibitors that bind to the colchicine binding site can largely overcome the above drawbacks and have therapeutic advantages over taxanes and vinca alkaloids. For example, they can be administered orally owing to the higher water solubility, they do not require surfactants for solubilization, thus are devoid of surfactant-induced hypersensitivity reaction. More importantly they are less prone to develop multi-drug resistance. Therefore, tubulin inhibitors that bind to colchicine binding site have received extraordinary attention in the last ten years.^15^

Colchicine binding site inhibitors (CBSIs) exert their biological effects by inhibiting tubulin assembly and suppressing microtubule formation.^14^ Colchicine I itself binds to tubulin very tightly, but neither colchicine nor compounds that bind to the colchicine binding site on tubulin have yet found significant use in cancer treatment.^16^ Combretastatin A-1 (CA-1) II and combretastatin A-4 (CA-4) III are two combretastatin analogs, both showed similar microtubule inhibitory activity but have limited water solubility.^17^ In order to improve the water solubility, both compounds were prepared as prodrugs of monosodium phosphate salt, and they can be transformed into the active components CA-1 and CA-4 *in vivo*.^18,19^ In phase II clinical trial, CA-4P showed no bone marrow toxicity, stomatitis, and hair loss.^20^ Ombrabulin IV is another CA-4 analog which has better solubility, oral bioavailability, improved anti-cancer activity and decreased toxicity.^14^

ZD6126 V is a NAC (*N*-acetylcolchicinenol) phosphate prodrug which showed microtubule inhibitory activity *in vivo*. Moreover, it showed no obvious neurotoxicity and displayed good antitumor activity.^21,22^ E7010 VI is an orally bioavailable sulfonamide that inhibits tubulin polymerization by binding to the colchicine binding site. It exhibited a broad spectrum of antitumor activity *in vitro* and *in vivo*.^23^

Plinabulin VII is in a world-wide Phase III clinical trial for non-small cell lung cancer.^24^ Plinabulin blocks the polymerization of tubulin in a unique manner, resulting in multi-factorial effects including an enhanced immune-oncology response,^25^ activation of the JNK pathway and disruption of the tumor blood supply.^26^

Indibulin VIII has shown promising anticancer activity with a minimal neurotoxicity in preclinical animal studies and in Phase I clinical trials for cancer chemotherapy.^27^ The antitumor activity of indibulin is believed to be primarily related to its effects on microtubules.^28^

**Fig. 1:**
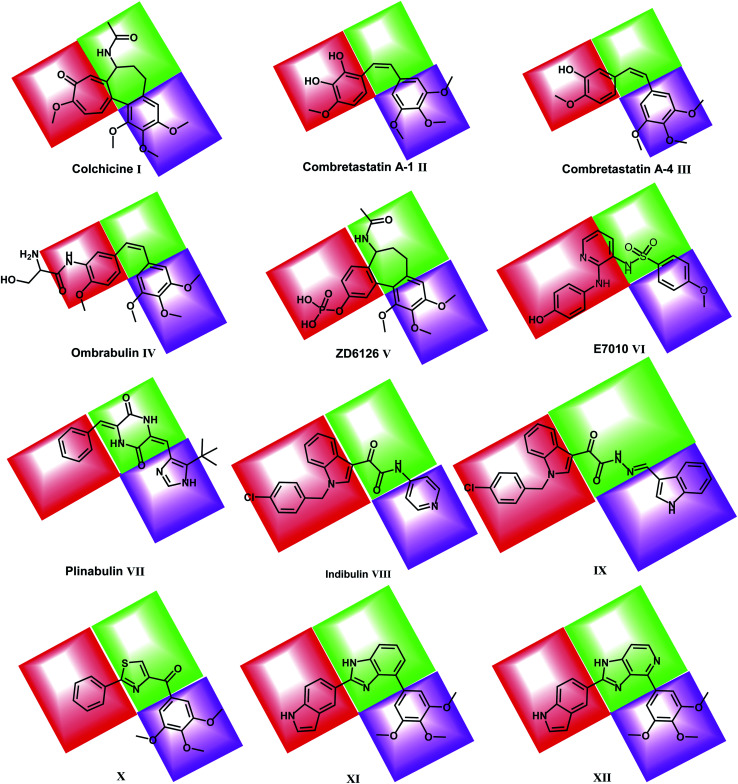
Reported colchicine binding site inhibitors.

Recently, many molecules (*e.g.* compounds IX, X, XI & XII) interacting with the colchicine binding site have been designed and synthesized with significant structural diversity. These compounds were modified and tested in order to find a highly potent, low toxicity agent for treatment of cancers^29,30^ (Fig. 1).

In the present work, our research group synthesized a series of thiazol-5(4*H*)-ones having the same pharmacophoric features of CBSIs and targeting the colchicine binding site, to examine their effect as anticancer agents with potential inhibitory effect on tubulin assembly.

### 1.1. Rational drug design

The colchicine binding site is positioned at the interface between the α and β subunits of the tubulin protein, with the major part of it buried in the β subunit and lined by the helices 7 and 8. The cavity, which is funnel shaped, has a volume of about 600 Å and opens up towards the α subunit of the interface surrounded by residues Asn101α, Thr179α, Ala180α, Val181α, Thr314β, Asn349β, Asn350β, and Lys352β. The other, β subunit, end of the cavity is surrounded by residues Tyr202β, Val238β, Thr239β, Cys241β, Leu242β, Leu248β, Leu252β, Leu255β, Ile378β, and Val318β and forms the narrow funnel end-like part of the cavity. The predominance of hydrophobic residues confer a strong hydrophobic character to this part of the cavity. At the wider portion, the cavity is surrounded by Ala250β, Asp251β, Lys254β, Asn258β, Met259β, Ala316β, Ala317β, Thr353β and Ala354β making it moderately polar/moderately hydrophobic.^31^

**Fig. 2:**
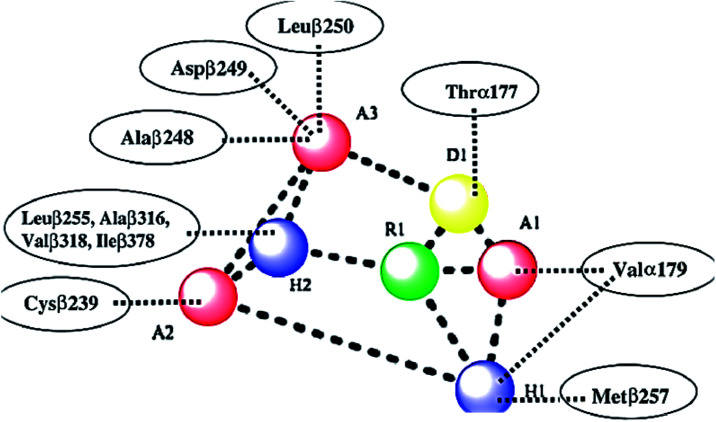
Seven pharmacophoric features: three hydrogen bond acceptors (A1, A2 & A3), one hydrogen bond donor (D1), two hydrophobic centers (H1 & H2), and one planar group (R1) (based on ref. 14 and 32).

As shown in Fig. 2, colchicine binding site inihbitors have the following seven pharmacophoric points: three hydrogen bond acceptors (A1, A2, and A3), one hydrogen bond donor (D1), two hydrophobic centers (H1 and H2) and one planar group (R1).^14,32^ Depending on these previously reported facts we can say that the molecules that will have these seven pharmacophoric features will be considered as promising tubulin inhibitors.

**Fig. 3:**
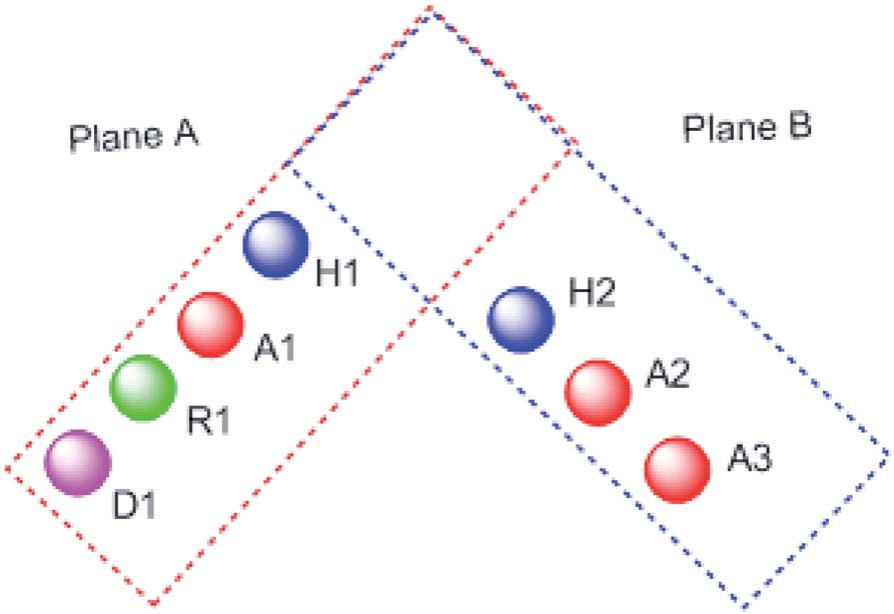
The pharmacophoric model with two planes: plane A (red) points A1, D1, H1 and R1, Plane B (blue) consists of points A2, A3, and H2, and (based on ref. 32).

It worth mentioning that, the seven pharmacophoric features can be partitioned among two planes. Features A1, D1, H1, and R1 lie in plane A, and features A2, A3, and H2 lie in plane B. Relative to one another, the two planes have a tilt of about 45° and match the shape of the colchicine site^32^ (Fig. 3).

**Fig. 4:**
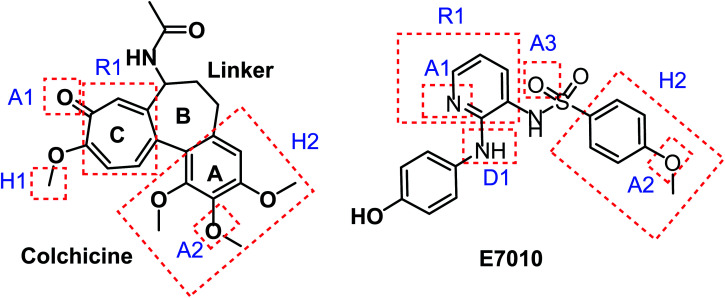
Pharmacophoric points of colchicine and E7010 as CBSIs.

Taking colchicine, as a lead compound for synthesis of the new derivatives, it is formed of three parts: ring A, ring B (linker), and ring C. Structure–activity study reveals that the A and C ring of colchicine comprise the minimal structural feature of the molecule needed for its high affinity binding to tubulin.^33^ The changes to the linker region affect the cytotoxic activity of the most reported colchicine binding site inihbitors.^34^Fig. 4 showed the pharmacophoric points of colchicine and E7010 as representative examples of CBSIs.

**Fig. 5:**
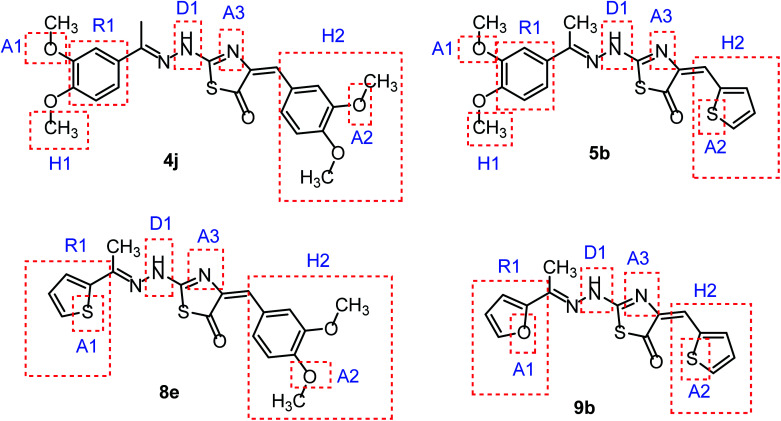
Some new thiazol-5(4*H*)-ones having the same essential pharmacophoric features of the reported CBSIs.

In continuation for our previous work of design and synthesis of new anticancer agents,^35–45^ the main target of this work was the synthesis of new thiazol-5(4*H*)-ones having the same essential pharmacophoric features of the reported CBSIs (Fig. 5). The core of our molecular design rational comprised bioisosteric modification strategies of CBSIs at three different positions.

**Fig. 6:**
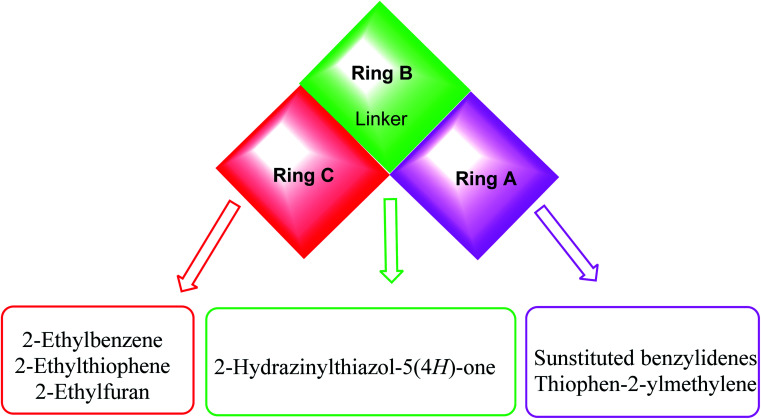
Summary for the possible modifications of CBSIs.

The first position was the ring A, where different substituted benzylidenes (compounds 4a–k & 8a–l) and thiophen-2-ylmethylene moiety (compounds 5a,b & 9a,b) were used as bioisosteres for ring A. The second position was the ring C, where different substituted 2-ethylbenzene moieties (compounds 4a–k & 5a,b), 2-ethylthiophene moiety (compounds 8a–l & 9a), and 2-ethylfuran moiety (compound 9b) were used as a bioisostere for ring C. The third position was the linker region, where 2-hydrazinylthiazol-5(4*H*)-one moiety was used to occupy the linker region in all compounds (Fig. 6).

**Fig. 7:**
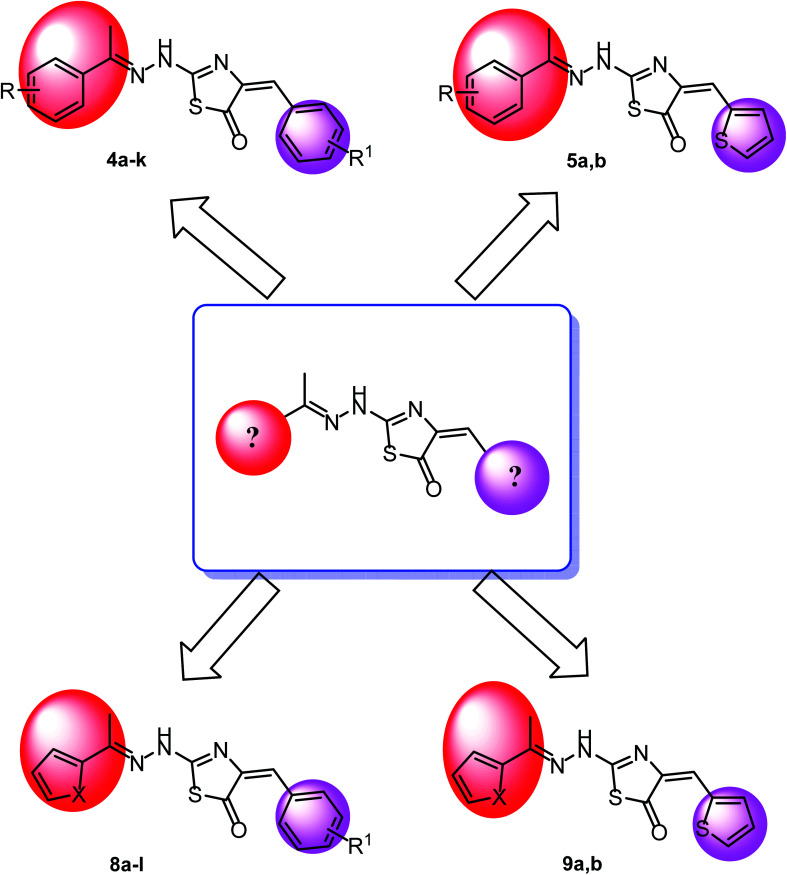
Rational of molecular design of the new proposed CBSIs.

The wide variety of modifications enabled us to study the SAR of these compounds as effective anti-cancer agents with potential tubulin polymerization inhibitory activity which is considered as a crucial objective of our work. All modification pathways and molecular design rationale were illustrated and summarized in Fig. 7.

## 2. Results and discussion

### 2.1. Chemistry

On the bases of green considerations and in continuation of our earlier endeavors^46–48^ toward the development of eco-friendly synthetic routes for heterocyclic systems, we report herein facile routes to various thiazol-5(4*H*)-one derivatives. In addition to conventional method, ultra-sound irradiation and microwave irradiation (solvent less) techniques were used in the synthesis of the new members. The reactions have been worked well in a one-pot fashion and were completed in a few minutes, with the desired products obtained in good yields.

**Scheme 1:**
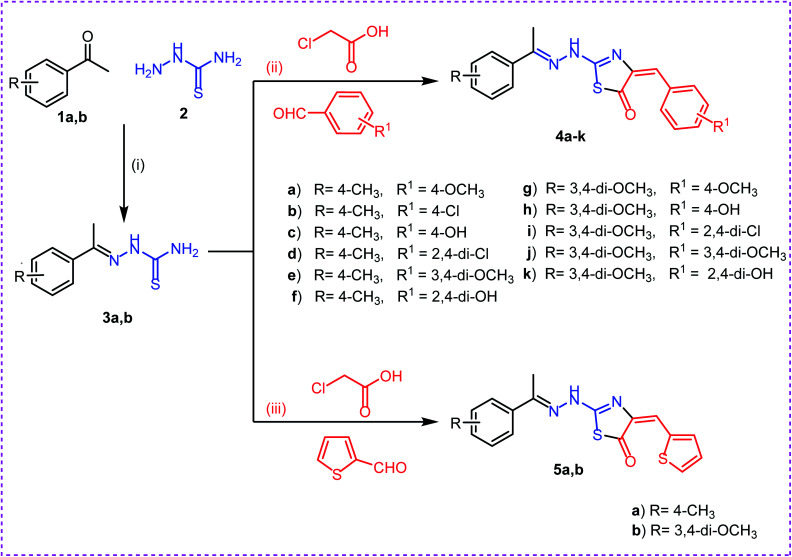
Synthesis of target compounds 4a–k and 5a,b. Reagents and conditions: (i), (ii) and (iii): absolute ethanol and gl. acetic acid/reflux.

**Scheme 2:**
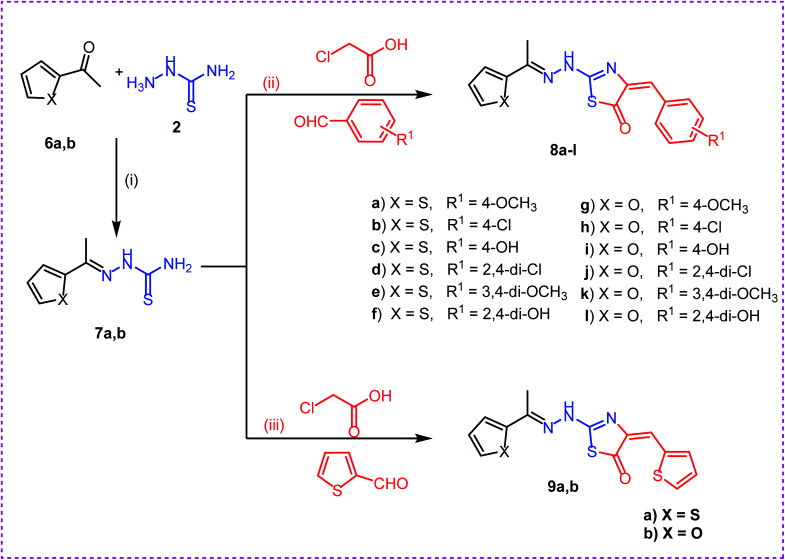
Synthesis of target compounds 8a–l and 9a,b. Reagents and conditions: (i), (ii) and (iii): absolute ethanol and gl. acetic acid/reflux.

The synthetic strategies adopted to obtain the target compounds are depicted in (Schemes 1 & 2). Firstly, refluxing of aryl ketones namely, 4-methyl acetophenone 1a and 3,4-methoxy acetophenone 1b with thiosemicarbazide 2 in absolute ethanol with catalytic amount of glacial acetic acid afforded the key intermediates thiosemicarbazones 3a,b. Reaction of thiosemicarbazones 3a,b with chloroacetic acid and appropriate substituted aromatic aldehydes namely, 4-methoxy benzaldehyde, 4-chloro benzaldehyde, 4-hydroxy benzaldehyde, 2,4-dichloro benzaldehyde, 3,4-dimethoxy benzaldehyde, and 2,4-dihydroxy benzaldehyde in ethanol and catalytic amount of glacial acetic acid afforded the target compounds 4a–k, respectively. Next, reaction of thiosemicarbazones 3a,b with chloroacetic acid and thiophene-2-carbaldehyde in ethanol and catalytic amount of glacial acetic acid afforded the target compounds 5a,b, respectively (Scheme 1).

Secondly, reaction of aryl ketones namely, 2-acetyl thiophene 6a and 2-acetyl furan 6b with thiosemicarbazide 2 in absolute ethanol with catalytic amount of glacial acetic acid gave the corresponding thiosemicarbazones 7a,b. Reaction of thiosemicarbazones 7a,b with chloroacetic acid and appropriate substituted aromatic aldehydes namely, 4-methoxybenzaldehyde, 4-chlorobenzaldehyde, 4-hydroxybenzaldehyde, 2,4-dichlorobenzaldehyde, 3,4-dimethoxybenzaldehyde, and 2,4-dihydroxybenzaldehyde in ethanol and catalytic amount of glacial acetic acid afforded the target compounds 8a–l, respectively. Next, reaction of thiosemicarbazones 7a,b with chloroacetic acid and thiophene-2-carbaldehyde in ethanol and catalytic amount of glacial acetic acid afforded the target compounds 9a,b, respectively (Scheme 2).

Finally, we repeated the two consequence steps of the reaction under both ultrasonic (in ethanol and catalytic amount of acetic acid) and microwave irradiation solvent-free conditions without isolation of the intermediates 3a,b and 7a,b. The reaction mixture afforded the same products with increased yield and shortage in the reaction time under ultrasonic method. Surprisingly, the yield was increased dramatically to 92%.

The structures of the synthesized compounds were established based on spectral data. The IR spectra of compound 3a,b showed the presence of NH_2_ and NH absorptions at a range of 3376–3151 cm^−1^. The ^1^H NMR spectra of compounds 3a,b exhibited singlet signals of methyl group at 2.32 ppm, singlet signals of NH group at 8.20 ppm, and singlet signals of NH_2_ at a range of 10.04–10.12 ppm.

For compounds 4a–k, 5a,b, 8a–l, and 9a,b the IR spectra showed absorption bands at ranges of 3115–3441, 1688–1722 and 1605–1648 corresponding to NH, C=O, C=N groups, respectively. The ^1^H NMR spectrum of 4a–k, 5a,b, 8a–l, and 9a,b showed characteristic peaks attributed to imine methyl protons between 2.47 ppm and 3.92 ppm. The proton present in the Schiff base appeared at a range of 8.25–8.66 ppm. The characteristic peaks due to NH proton appeared between 10.07 and 12.87 ppm as singlet peaks. The ^13^C NMR spectra of compounds 4a–k, 5a,b, 8a–l, and 9a,b provided additional evidence in support of the proposed structures. All spectra that supports elucidating the chemical structures of the new derivatives are supplied with this research work as ESI file.†

### 2.2. Biological evaluation

#### 2.2.1. *In vitro* anti-proliferative activity

The synthesized compounds were tested for their *in vitro* cytotoxic activities using standard MTT method,^49–51^ against a group of human cancer cell lines namely; colorectal carcinoma (HCT-116), hepatocellular carcinoma (HepG-2), and breast cancer (MCF-7). Colchicine was used as a positive control. The results of cytotoxicity test were reported as growth inhibitory concentration (IC_50_) values and summarized in Table 1.

**Table 1:** *In vitro* anti-proliferative activities of the tested compounds and *in vitro* tubulin polymerization inhibition

Comp.	IC_50_^a^ (μM)			IC_50_ (nM) Tubulin polymerization inhibition
	HCT-116	HepG-2	MCF-7	
3a	NA^b^	NA^b^	NA^b^	NT^c^
3b	17.33 ± 1.4	12.91 ± 0.4	22.38 ± 1.0	44.47 ± 2.3
4a	45.61 ± 3.5	34.04 ± 30.9	NA^b^	NT^c^
4b	18.17 ± 1.3	11.14 ± 0.1	22.41 ± 1.0	NT^c^
4c	38.45 ± 2.5	35.20 ± 0.8	41.80 ± 1.2	NT^c^
4d	10.64 ± 0.4	**6.60 ± 0.1** d	14.77 ± 0.6	19.68 ± 0.8
4e	12.95 ± 0.2	12.44 ± 0.1	18.20 ± 0.5	47.39 ± 1.8
4f	**5.66 ± 0.1** d	**2.89 ± 0.1** d	**4.46 ± 0.1** d	**9.33 ± 0.3** d
4g	12.88 ± 0.6	8.68 ± 0.1	17.38 ± 0.5	NT^c^
4h	41.81 ± 1.6	37.53 ± 0.8	48.83 ± 1.4	NT^c^
4i	40.51 ± 1.1	31.86 ± 0.8	40.11 ± 1.9	NT^c^
4j	22.85 ± 0.7	32.60 ± 0.9	43.30 ± 1.8	NT^c^
4k	11.71 ± 0.3	12.21 ± 0.4	13.91 ± 0.5	50.01 ± 0.4
5a	**6.41 ± 0.1** d	**3.34 ± 0.1** d	**4.51 ± 0.6** d	**9.52 ± 0.3** d
5b	18.40 ± 0.2	17.52 ± 0.7	21.75 ± 1.0	NT^c^
7a	NA^b^	NA^b^	NA^b^	NT^c^
7b	NA^b^	NA^b^	NA^b^	NT^c^
8a	35.53 ± 2.0	26.61 ± 0.9	32.12 ± 1.2	NT^c^
8b	18.65 ± 0.6	19.48 ± 0.7	18.18 ± 0.6	NT^c^
8c	17.24 ± 0.9	12.23 ± 0.3	19.02 ± 0.5	NT^c^
8d	48.57 ± 1.8	45.32 ± 1.2	NA^b^	NT^c^
8e	34.66 ± 1.0	27.79 ± 0.5	25.82 ± 0.6	NT^c^
8f	**8.96 ± 0.2** d	**3.23 ± 0.1** d	**7.65 ± 0.2** d	11.59 ± 0.2
8g	**6.59 ± 0.1** d	**4.69 ± 0.1** d	**9.29 ± 0.1** d	14.17 ± 0.3
8h	12.03 ± 0.3	10.32 ± 0.6	20.00 ± 0.4	38.37 ± 1.4
8i	15.55 ± 0.4	12.52 ± 0.2	22.69 ± 0.7	47.77 ± 2.1
8j	NA^b^	NA^b^	NA^b^	**NT** c
8k	**5.55 ± 0.1** d	**4.58 ± 0.1** d	**9.15 ± 0.2** d	13.50 ± 0.8
8l	10.28 ± 0.1	**3.73 ± 0.1** d	**7.89 ± 0.5** d	13.16 ± 0.9
9a	24.18 ± 0.7	21.90 ± 0.9	33.59 ± 0.9	39.11 ± 1.0
9b	NA^b^	45.09 ± 0.7	NA^b^	**NT** c
Colchicine	9.30 ± 0.2	7.44 ± 0.2	10.45 ± 0.3	10.65 ± 0.2

a IC_50_ values are the mean ± S.D. of three separate experiments.

b NA: compounds having IC_50_ value > 50 μM.

c NT: compounds not tested for their tubulin polymerization assay.

d Bold figures indicate superior potency than colchicine.

The tested compounds exhibited different degrees of anti-proliferative activities against the three tested cell lines. Their activities range from excellent, good, moderate to weak.

In general, compounds 4f, 5a, 8f, 8g, and 8k showed superior antiproliferative activities against the three cell lines with IC_50_ values ranging from 2.89 to 9.29 μM. The cytotoxic activities of such compounds were higher than that of the reference drug, colchicine (IC_50_ = 9.30, 7.44, and 10.45 μM against HCT-116, HepG-2, and MCF-7, respectively). Compound 4f, as representative example, was 1.64, 2.57 and 2.34 times as active as colchicine against HCT-116, HepG-2, and MCF-7, respectively. Also, compound 5a, was 1.45, 2.23 and 2.32 times as active as colchicine against HCT-116, HepG-2, and MCF-7, respectively.

Additionally, compound 8l exhibited superior activities against HepG-2, and MCF-7 with IC_50_ values of 3.73 and 7.89 μM, respectively, whereas, compound 4d was higher than colchicine against only HepG-2 cells with IC_50_ value of 6.60 μM.

Moreover, several compounds such as 4e, 4g, 4k, 8b, 8c, and 8h demonstrated strong anti-proliferative activities over all examined cell lines with IC_50_ values ranging from 10.32 to 20.00 μM. Also, compounds 3b, 4b, 4d, 5b, and 8i showed strong anti-proliferative activities against only two cell lines with IC_50_ values ranging from 10.64 to 18.40 μM.

Additionally, compounds 4b, 4j, 5b, 8a, 8e, 8i, and 9a displayed moderate anti-proliferative activities against at least one cell line with IC_50_ values ranging from 21.90 to 27.79 μM.

On the other hand, compounds 4a, 4c, 4h, 4i, 4j, 8a, and 8d displayed weak anti-proliferative activities against at least two cell lines with IC_50_ values ranging from 31.86 to 48.83 μM.

Finally, compounds 3a, 7a, 7b, and 8j showed no activity against any of the tested cancer cell lines. In addition, compound 9b was inactive against HCT-116 and MCF-7 and compounds 4a, 8d revealed to be inactive against MCF-7 only.

#### 2.2.2. Tubulin polymerization assay

To investigate whether the cytotoxic activity of the synthesized compounds was related to an interaction with the tubulin system, an *in vitro* tubulin polymerization assay was performed for the most cytotoxic members. The inhibition assay on microtubule polymerization was evaluated turbidimetrically using a fluorescent plate reader.^52^ Colchicine was used as a positive control (Table 1).

Compounds 4f and 5a were the most potent tubulin polymerization inhibitors with an IC_50_ values of 9.33 and 9.52 nM, respectively. These compounds had activities higher than that of colchicine (IC_50_ = 10.65 nM). Additionally, compounds 8f, 8k, and 8l showed promising activities nearly equal to colchicine with IC_50_ values of 11.59, 13.50, and 13.16 nM, respectively. Also, compounds 4d and 8g showed strong tubulin polymerization inhibitory activities with IC_50_ values of 19.68 and 14.17 nM, respectively. Finally, compounds 3b, 4e, 4k, 8h, 8i and 9a exhibited moderate tubulin polymerization inhibitory activities with IC_50_ values ranging from 38.37 to 50.01 nM. The results strongly implicated a direct interaction between the examined compounds and tubulin. It can be concluded that the cytotoxic activity of the synthesized compounds may derive from an interaction with tubulin and an interference with microtubule assembly.

#### 2.2.3. Cell cycle analysis

To gain a better insight into the impact of compound 5a on cancer cell growth inhibition, its impact on cell cycle distribution and apoptosis induction was assessed using HepG-2 cells according to the method outlined by Wang *et al.*^53^ In fact, anticancer agents hinder cancer cell growth and multiplication by arresting cell division at distinct checkpoints, and that cells resist apoptosis are highly resistant to cancer treatment.^54^ In the present work, HepG-2 cell line was treated with compound 5a at a concentration equals its IC_50_ value on tubulin (9.52 nM) for 24 h.

**Fig. 8:**
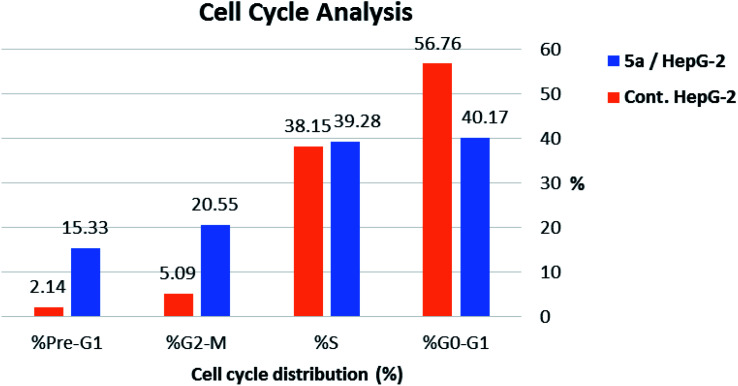
Cell cycle analysis and apoptosis effect in HepG-2 cell line when treated with compound 5a.

**Fig. 9:**
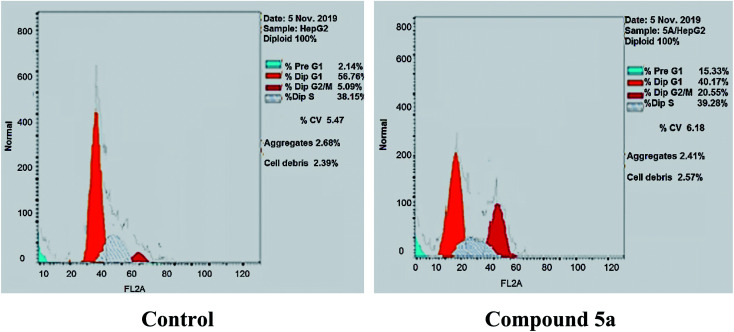
HepG-2 cells distribution upon treatment with compound 5a.

As shown in Table 2, Fig. 8 and 9, the percentage of HepG-2 cells at S phase was increased from 38.15% to 39.28% after incubation with compound 5a. Additionally, cells in G2/M phase markedly increased from 5.09% to 20.55% and the G1 phase decreased from 56.76% in control to 40.17%, indicating that compound 5a caused cell arrest at G2/M phase. Also, it was found that the cells increased from 2.14% to 15.33% at pre-G1 phase, indicating that compound 5a caused apoptosis at pre-G1 phase.

**Table 2:** Effect of compound 5a on cell cycle progression in HepG-2 cells

Sample	Cell cycle distribution (%)			
	% G0-G1	% S	% G2-M	% Pre-G1
5a/HepG-2	40.17	39.28	20.55	15.33
Cont. HepG-2	56.76	38.15	5.09	2.14

#### 2.2.4. Annexin V-FITC apoptosis assay

**Fig. 10:**
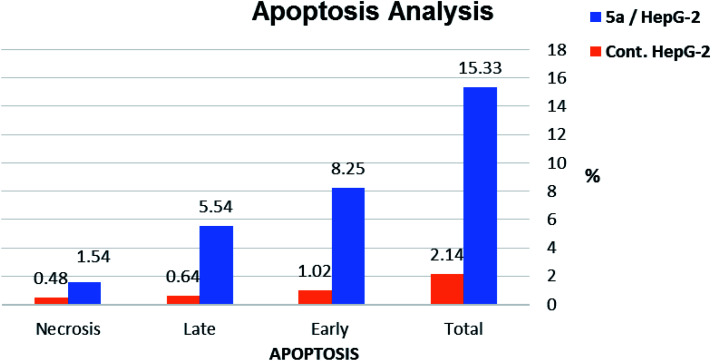
Percentage of induced cell death by compound 5a on HepG-2 cells.

**Fig. 11:**
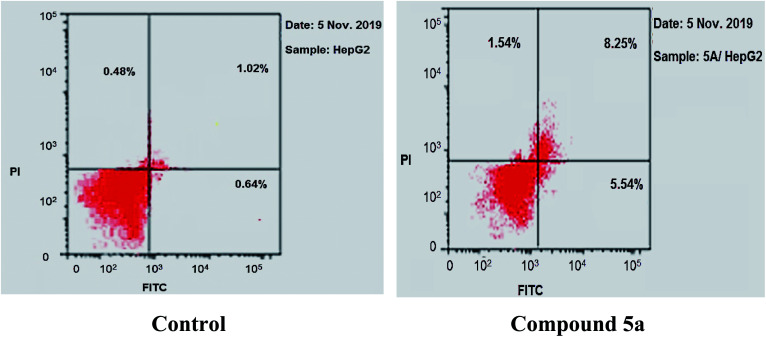
Induced apoptosis on HepG-2 cells by compound 5a.

To further confirm Apoptotic effect of compound 5a in HepG-2 cells, Annexin V and PI double staining assay was performed.^55^ In this test, HepG-2 cells were incubated with compound 5a at concentration of 2.5 μM for 24 h. The results were reported in Table 3, Fig. 10 and 11.

**Table 3:** Apoptosis and necrosis percent induced by compound 5a in HepG-2 cells

Sample	Apoptosis			Necrosis
	Total	Early	Late	
5a/HepG-2	15.33	8.25	5.54	1.54
Cont. HepG-2	2.14	1.02	0.64	0.48

The results revealed that compound 5a induced total apoptotic effect equal 13.79% which was eight time more than the control (1.66%). In details, compound 5a obviously induced early apoptosis by 8.25% and enhanced late apoptosis by 5.54% when compared with the untreated control HepG-2 cells (1.02% and 0.64%, respectively).

### 2.3. Docking studies

Molecular docking studies were conducted to give a guidance of molecular binding modes of the tested molecules inside the pocket of tubulin heterodimers. The selected compounds have been docked against tubulin heterodimers using MOE2014 to determine the free energy and binding mode. The selection of the most promising molecules depended on the rightbinding mode and the binding free energy (Δ*G*).^56^

**Fig. 12:**
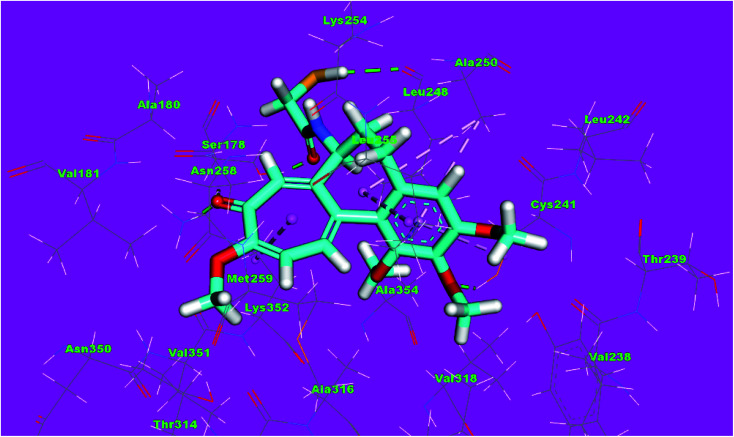
3D structure of co-crystallized ligand, DAMA-colchicine, docked into the active site of tubulin.

The binding free energies of the synthesized compounds and the reference ligand were summarized in Table 4. The binding mode of the co-crystallized ligand, DAMA-colchicine, exhibited an energy binding of −13.08 kcal mol^−1^. The ring A (trimethoxy phenyl moiety) formed a hydrogen bond with Cys241. Also, it formed five hydrophobic interactions with Ala250, Leu255 and Cys241. The 2-mercaptoacetamide moiety formed two hydrogen bonds with Leu248 and Ser178. The ring C formed one hydrogen bonding and one hydrophobic interaction with Lys352 (Fig. 12).

**Table 4:** The docking binding free energies of the synthesized and co-crystallized ligand (DAMA-colchicine) compounds against tubulin

Comp.	Binding free energy (kcal mol^−1^)	Comp.	Binding free energy (kcal mol^−1^)
4a	−9.67	8b	−10.18
4b	−10.72	8c	−10.08
4c	−9.35	8d	−11.11
4d	−11.44	8e	−13.19
4e	−12.44	8f	−9.99
4f	−11.80	8g	−11.00
4g	−12.55	8h	−10.31
4h	−12.22	8i	−10.02
4i	−12.53	8j	−10.98
4j	−12.20	8k	−12.28
4k	−14.35	8L	−10.15
5a	−10.41	9a	−9.26
5b	−12.71	9b	−10.52
8a	−10.48	DAMA-colchicine	−13.08

**Fig. 13:**
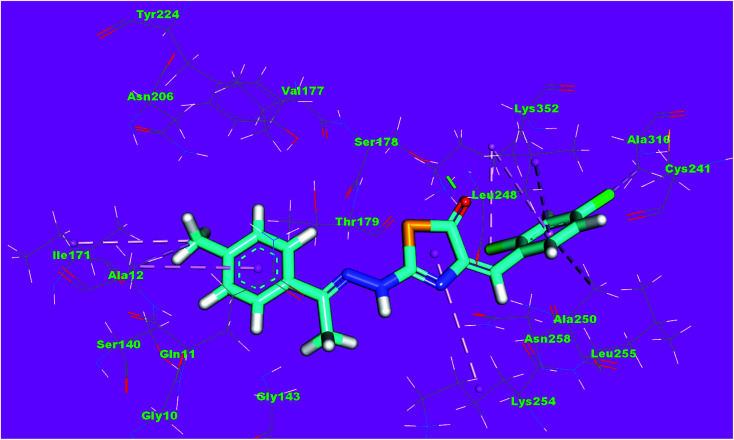
3D structure of compound 4d docked into the active site of tubulin.

Compound 4d as a representative example showed a binding mode like that of DAMA-colchicine, with affinity value of −11.44 kcal mol^−1^. The 2,4-dichlorophenyl moiety formed five hydrophobic interactions with Ala316, Lys352, Leu248 and Ala250. The thiazol moiety formed one hydrogen bond with Ser178 and one hydrophobic interaction with Lys254. The *p*-tolyl moiety formed three hydrophobic interactions with Ile171 and Ala12 (Fig. 13).

**Fig. 14:**
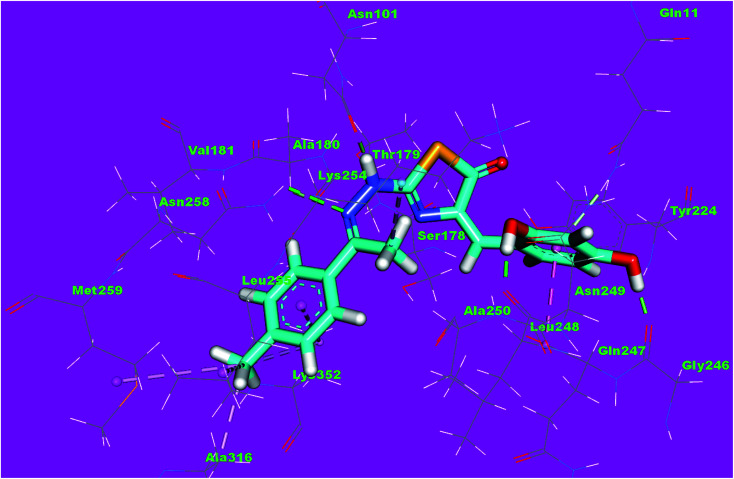
3D structure of compound 4f docked into the active site of tubulin.

The binding mode of compound 4f exhibited an affinity value of −11.80 kcal mol^−1^. The 2,4-dihydroxybenzyl moiety formed two hydrogen bonds with Gly246 and Leu248. Also, it formed two hydrophobic interactions with Gln247 and Gln11. The hydrazinyl moiety formed two hydrogen bonds with Asn258 and Asn101. The *p*-tolyl moiety formed five hydrophobic interactions with Leu255, Met259, Ala316, and Lys352 (Fig. 14).

**Fig. 15:**
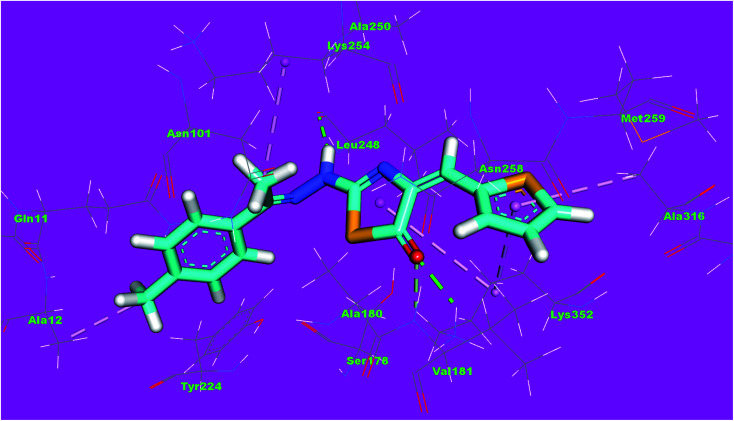
3D structure of compound 5a docked into the active site of tubulin.

The binding mode of compound 5a exhibited an affinity value of −10.41 kcal mol^−1^. The thiophene moiety formed two hydrophobic interactions with Ala316 and Lys352. The hydrazinylthiazol moiety formed two hydrogen bonds with Lys352 and Lys254. Also, it formed one hydrophobic interaction with Lys352. The *p*-tolyl moiety formed one hydrophobic interaction with Ala12 (Fig. 15).

**Fig. 16:**
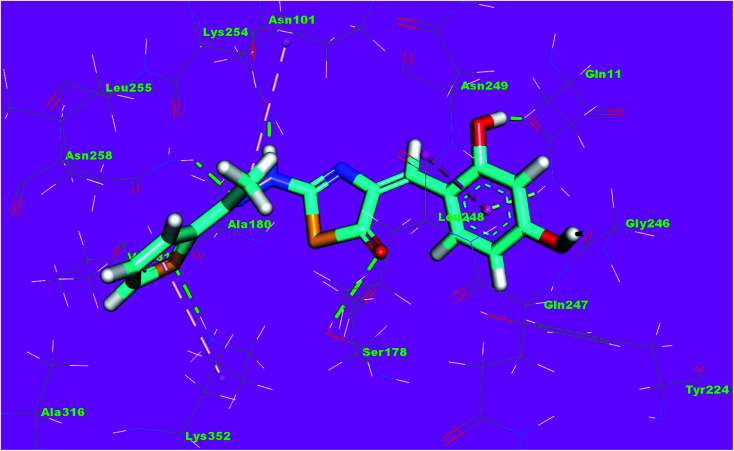
3D structure of compound 8f docked into the active site of tubulin.

The binding mode of compound 8f exhibited an affinity value of −13.19 kcal mol^−1^. The 2,4-dihydroxyphenyl moiety formed two hydrogen bonds with Gln11 and Gly246. In addition, it formed one hydrophobic interaction with Leu248. The hydrazinylthiazol moiety formed three hydrogen bonds with Ser178, Asn101 and Asn258. The thiophene moiety formed one hydrophobic interaction and one hydrogen bond with Lys352 (Fig. 16).

### 2.4. Structure–activity relationship (SAR)

As outlined in the rationale molecular design, it was aimed at studying the SAR of the newly synthesized thiazol-5(4*H*)-one derivatives as potential tubulin polymerization inhibitors.

Initially, the effect of the ring A on the activity was explored. Comparing the cytotoxic activity of compounds 5a incorporating thiophen-2-ylmethylene as a ring A with compounds 4f incorporating substituted benzylidenes as a ring A, indicated that the substituted benzylidenes is more advantageous than thiophen-2-ylmethylene moiety. For the substituted benzylidenes, the cytotoxic activities were decreased in the order of 3,4-dimethoxy benzylidenes 8k > 4-methoxy benzylidenes 8g > 2,4-dihydroxy benzylidenes 8l > 4-chloro benzylidenes 8h > 4-hydroxy benzylidenes 8i > 2,4-dichloro benzylidenes 8j.

Then, the impact of the ring C was investigated. The decreased IC_50_ values of compounds 4c, 4d, 4e, and 4f incorporated 1-ethyl-4-methylbenzene as ring C, than those of their corresponding members 4h, 4i, 4j, and 4k incorporating 1-ethyl-4-methoxybenzene as ring C, indicated that 1-ethyl-4-methylbenzene moiety is advantageous. Also, the decreased IC_50_ value of compound 5a incorporating 1-ethyl-4-methylbenzene as ring C than the corresponding member 5b 1-ethyl-4-methoxybenzene as ring C, confirm the positive effect of 1-ethyl-4-methylbenzene moiety. In addition, the higher activity of compound 9a incorporated 2-ethylthiophene moiety as ring C than compounds 9b with 2-ethylfuran moiety, revealed that 2-ethylthiophene moiety is more preferred biologically than 2-ethylfuran one.

## 3. Conclusion

To sum up, thirty-one new derivatives based on thiazol-5(4*H*)-one were designed and eco-friendly synthesized using conventional, ultrasound irradiation and microwave-assisted methods. The synthesized derivatives were evaluated for their anti-proliferative activities against a group of three human cancer cell lines including; colorectal carcinoma (HCT-116), hepatocellular carcinoma (HepG-2), and breast cancer (MCF-7) using MTT assay. Compound 4f has appeared as the most active member against all examined cells with IC_50_ values of 5.66 ± 0.1, 2.89 ± 0.1, and 4.46 ± 0.1 μM, respectively, as it was 1.64, 2.57 and 2.34 times more active than colchicine (IC_50_ = 9.30 ± 0.2, 7.44 ± 0.2, and 10.45 ± 0.3 μM, respectively). In addition, compounds 5a, 8f, 8g and 8k showed excellent anti-proliferative activity against the three tested cell lines with IC_50_ values ranging from 3.23 to 9.29 μM. Moreover, the most active compounds have been studied for their inhibitory activities of tubulin polymerization. Tubulin polymerization assay findings were consistent with cytotoxicity data results. Moreover, compound 5a arrested the cell cycle in the G2/M phase and induced apoptosis in HepG-2 cells. Docking experiments assisted these findings by anticipating potential binding interactions between the target compounds and the active sites of tubulin heterodimers. The most effective candidates in the quest for strong and selective antineoplastic agents will serve as valuable lead compounds and merit further investigations.

## 4. Experimental

### 4.1. Chemistry

All melting points were measured on a Gallen Kamp melting point apparatus (Sanyo Gallen Kamp, UK) and were uncorrected. The Microwave reactions were done by Microsynth instrument type MA143 (Micro wave flux). The ultrasound-assisted reactions were performed in Digital Ultrasonic Cleaner CD-4830 (35 KHz, 310 W). The IR spectra were recorded on a Pye-Unicam SP-3-300 infrared spectrophotometer (KBr dicks) and expressed in wave number (cm^−1^). ^1^H NMR spectra were run at 300 and 400 MHz, on a Varian Mercury VX-300 and Bruker Avance III NMR spectrometer, respectively, while ^13^C NMR spectra were run at 100 MHz. TMS was used as an internal standard in deuterated dimethylsulphoxide (DMSO-*d*_*6*_). The mass spectra were recorded on Shimadzu GCMS-QP-1000EX mass spectrometer at 70 eV. Elemental analyses were performed on CHN analyzer and all compounds were within ± 0.4 of the theoretical values. The reactions were monitored by thin-layer chromatography (TLC) using TLC sheets coated with UV fluorescent silica gel Merck 60 F254 plates and were visualized using UV lamp and different solvents as mobile phases. All reagents and solvents were purified and dried by standard techniques.

#### 4.1.1. General procedure for synthesis of compounds 3a,b & 7a,b

A solution of thiosemicarbazide (10 mmol) and aryl ketones (10 mmol) namely, 4-methyl acetophenone 1a, 3,4-methoxy acetophenone 1b, 2-acetyl thiophene 6a and 2-acetyl furan 6b, in anhydrous ethanol (20 mL) and few drops of g. acetic acid was refluxed for 2 h. The formed product was filtered, washed with ethanol, dried and crystallized from ethanol to give compounds 3a,b & 7a,b, respectively.

##### 4.1.1.1. (*E*)-2-(1-(*p*-Tolyl)ethylidene)hydrazine-1-carbothioamide (3a)

White crystals; mp 240–242 °C; IR (KBr, cm^−1^): 3376, 3230, 3151 (NH_2_, NH), 3053 (CH aromatic), 2933 (CH aliphatic), 1590 (C=N); ^1^H NMR (DMSO-*d*_*6*_) *δ* ppm: 2.32 (s, 3H, CH_3_), 2.41 (s, 3H, CH_3_), 7.16 (d, 2H, *J* = 6.6 Hz, Ar–H, H_3_, H_5_ of phenyl), 7.44 (d, 2H, *J* = 6.6 Hz, Ar–H, H_2_, H_6_ of phenyl), 8.20 (s, 2H, NH_2_, D_2_O exchangeable), 10.12 (s, 1H, NH, D_2_O exchangeable); anal. calcd for C_10_H_13_N_3_S (207.3): C, 57.94; H, 6.32; N, 20.27; found: C, 57.73; H, 6.22; N, 20.15%.

##### 4.1.1.2. (*E*)-2-(1-(3,4-Dimethoxyphenyl)ethylidene)hydrazine-1-carbothioamide (3b)

White crystals (yield 85%); mp 205–207 °C °C; IR (KBr, cm^−1^): 3375, 3267, 3157 (NH_2_, NH), 3099 (CH aromatic), 2931 (CH aliphatic), 1599 (C=N), 1245 (C=S); ^1^H NMR (DMSO-*d*_*6*_) *δ* ppm: 2.32 (s, 3H, CH_3_), 3.80 (s, 3H, OCH_3_), 6.91 (s, 3H, OCH_3_), 6.97 (d, 1H, *J* = 8 Hz, Ar–H, H_5_ of phenyl), 7.47 (d, 2H, *J* = 8 Hz, Ar–H, H_2_, H_6_ of phenyl), 8.20 (s, 2H, NH_2_, D_2_O exchangeable), 10.04 (s, 1H, NH, D_2_O exchangeable); ^13^C NMR (DMSO-*d*_*6*_) *δ* ppm: 179.0, 150.6, 149.0, 148.7, 130.8, 120.4, 111.4, 110.0, 56.1, 55.9, 14.4; anal. calcd for C_11_H_15_N_3_O_2_S (253.3): C,52.16; H, 5.97; N, 16.59; found: C, 51.91; H, 5.88; N, 16.48%.

##### 4.1.1.3. (*E*)-2-(1-(Thiophen-2-yl)ethylidene)hydrazine-1-carbothioamide (7a)

White crystals (yield 75%); mp 122–124 °C; IR (KBr, cm^−1^): 3407, 3241, 3145 (NH_2_, NH), 2979 (CH aliphatic), 1606 (C=N); ^1^H NMR (DMSO-*d*_*6*_) *δ* ppm: 2.29 (s, 3H, CH_3_), 6.47 (s, 2H, NH_2_, D_2_O exchangeable), 7.02–704 (m, 1H, Ar–H), 7.31 (d, 1H, Ar–H), 7.35 (d, 1H, Ar–H), 8.73 (s, 1H, NH, D_2_O exchangeable); anal. calcd for C_7_H_9_N_3_S_2_ (199.0): C, 42.19; H, 4.55; N, 21.09; found: C, 41.95; H, 4.46; N, 21.00%.

##### 4.1.1.4. (*E*)-2-(1-(Furan-2-yl)ethylidene)hydrazine-1-carbothioamide (7b)

White crystals; mp 122–124 °C; IR (KBr, cm^−1^): 3346, 3249, 3132 (NH_2_, NH), 3053 (CH aromatic), 2933 (CH aliphatic), 1602 (C=N); ^1^H NMR (DMSO-*d*_*6*_) *δ* ppm: 2.26 (s, 3H, CH_3_), 6.56–6.57 (m, 1H, Ar–H), 7.07 (d, 1H, Ar–H), 7.73 (s, 2H, NH_2_, D_2_O exchangeable), 8.25 (d, 1H, Ar–H), 10.35 (s, 1H, NH, D_2_O exchangeable); anal. calcd for C_7_H_9_N_3_OS (183.2): C, 45.89; H, 4.95 N, 22.93; found: C, 45.76; H, 4.86; N, 22.80%.

#### 4.1.2. General procedure for synthesis of compounds 4a–k, 5a,b, 8a–l & 9a,b

##### 4.1.2.a. Conventional method

A mixture of starting compounds 3a,b & 7a,b (10 mmol), chloroacetic acid (1 mmol), and the appropriate aromatic aldehyde (10 mmol) namely, 4-methoxy benzaldehyde, 4-chloro benzaldehyde, 4-hydroxy benzaldehyde, 2,4-dichloro benzaldehyde, 3,4-dimethoxy benzaldehyde, and 2,4-dihydroxy benzaldehyde was refluxed in a mixture of ethanol (20 mL) and acetic acid glacial (5 mL) for 3 h. After cooling, the formed precipitate were filtered and crystallized from methanol to afford the corresponding compounds 4a–k, 5a,b, 8a–l & 9a,b, respectively.

##### 4.1.2.b. Under microwave method

A mixture of thiosemicarbazide (10 mmol), appropriate aryl ketones (10 mmol) namely, 4-methyl acetophenone 1a, 3,4-methoxy acetophenone 1b, 2-acetyl thiophene 6a and 2-acetyl furan 6b, and glacial acetic acid (2 mL) was added to the reaction vessel placed into the microwave reactor. The mixture was allowed to react under microwave irradiation of 200–400 W at 120 °C for 2 min. After the reaction completion (monitored by TLC), a mixture of chloroacetic acid (10 mmol) and appropriate aromatic aldehydes (10 mmol) namely, 4-methoxy benzaldehyde, 4-chloro benzaldehyde, 4-hydroxy benzaldehyde, 2,4-dichloro benzaldehyde, 3,4-dimethoxy benzaldehyde, and 2,4-dihydroxy benzaldehyde was added to the reaction vessel. The reaction mixture was then subjected to microwave irradiation of 200–400 W at 120 °C for 2 min. with continuous stirring *via* the automatic mode. The reaction was monitored using TLC. After completion of the reaction and cooling, the product was obtained and crystallized from the proper solvent to give the corresponding title compounds 4a–k, 5a,b, 8a–l & 9a,b.

##### 4.1.2.c. Under sonication method

A mixture of thiosemicarbazide (10 mmol) and appropriate aryl ketones (10 mmol) namely, 4-methyl acetophenone 1a, 3,4-methoxy acetophenone 1b, 2-acetyl thiophene 6a and 2-acetyl furan 6b, in anhydrous ethanol (20 mL) with catalytic amount of glacial acetic acid was placed in Erlenmyer flask (50 mL) and subjected to ultrasound waves at room temperature for 10 min. A mixture of chloroacetic acid (10 mmol) and (1 mmol) of appropriate aromatic aldehydes namely, 4-methoxy benzaldehyde, 4-chloro benzaldehyde, 4-hydroxy benzaldehyde, 2,4-dichloro benzaldehyde, 3,4-dimethoxy benzaldehyde, and 2,4-dihydroxy benzaldehyde was added to the reaction vessel, and subjected to ultrasound waves at room temperature for 15 min. The formed precipitate was filtered, dried, and crystallized from the appropriate solvent to afford the target compounds 4a–k, 5a,b, 8a–l & 9a,b. Reaction time and yield of the conventional, ultrasonic and microwave procedures were summarized in Table 5.

**Table 5:** Reaction time and yield of both conventional, ultrasonic and microwave assisted synthesis of the synthesized compounds

Comp.	Time (min)			Yield		
	C^a^	US^a^	MW^a^	C^a^	US^a^	MW^a^
4a	180 + 240	10 + 15	2 + 2	55	77	88
4b	180 + 240	10 + 15	2 + 2	54	79	90
4c	180 + 240	10 + 15	2 + 2	56	77	88
4d	180 + 240	10 + 15	2 + 2	54	66	90
4e	180 + 240	10 + 15	2 + 2	57	60	92
4f	180 + 240	10 + 15	2 + 2	49	62	93
4g	180 + 240	10 + 15	2 + 2	58	67	92
4h	180 + 240	10 + 15	2 + 2	55	78	90
4i	180 + 240	10 + 15	2 + 2	53	62	95
4j	180 + 240	10 + 15	2 + 2	56	78	91
4k	180 + 240	10 + 15	2 + 2	50	77	88
5a	180 + 240	10 + 15	2 + 2	57	68	92
5b	180 + 240	10 + 15	2 + 2	60	77	93
8a	180 + 240	10 + 15	2 + 2	62	79	94
8b	180 + 240	10 + 15	2 + 2	56	71	93
8c	180 + 240	10 + 15	2 + 2	54	62	95
8d	180 + 240	10 + 15	2 + 2	55	78	91
8e	180 + 240	10 + 15	2 + 2	51	77	88
8f	180 + 240	10 + 15	2 + 2	52	79	90
8g	180 + 240	10 + 15	2 + 2	56	79	90
8h	180 + 240	10 + 15	2 + 2	50	70	92
8i	180 + 240	10 + 15	2 + 2	53	73	93
8j	180 + 240	10 + 15	2 + 2	55	67	90
8k	180 + 240	10 + 15	2 + 2	45	69	95
8l	180 + 240	10 + 15	2 + 2	54	78	91
9a	180 + 240	10 + 15	2 + 2	60	60	90
9b	180 + 240	10 + 15	2 + 2	61	77	94

a C: conventional, US: ultrasonic, MW: microwave.

###### 4.1.2.c.1. 4-((*E*)-4-Methoxybenzylidene)-2-(2-((*E*)-1-(*p*-tolyl)ethylidene)hydrazinyl) thiazol-5(4*H*)-one (4a)

Yellow crystals; mp 248–250 °C; IR (KBr, cm^−1^): 3112 (NH), 3009 (CH-aromatic), 2973 (CH aliphatic), 1722 (C=O), 1636 (C=N); ^1^H NMR (DMSO-*d*_*6*_) *δ* ppm: 3.31 (s, 6H, 2CH_3_), 3.86 (s, 3H, OCH_3_), 6.98–7.06 (m, 4H, Ar–H), 7.67–7.72 (d, 4H, Ar–H), 8.31 (s, 1H, C=CH̲ olefinic), 11.88 (s, 1H, NH, D_2_O exchangeable); anal. calcd for C_20_H_19_N_3_O_2_S (365.5): C, 65.73; H, 5.24; N, 11.50; found: C, 65.51; H, 5.16; N, 11.41%.

###### 4.1.2.c.2. 4-((*E*)-4-Chlorobenzylidene)-2-(2-((*E*)-1-(*p*-tolyl)ethylidene)hydrazinyl) thiazol-5(4*H*)-one (4b)

Pale yellow crystals; mp 266–268 °C; IR (KBr, cm^−1^): 3112 (NH), 3027 (CH-aromatic), 2938 (CH aliphatic), 1709 (C=O), 1644 (C=N); ^1^H NMR (DMSO-*d*_*6*_) *δ* ppm: 3.31 (s, 3H, CH_3_), 3.88 (s, 3H, CH_3_), 7.47–7.96 (m, 4H, Ar–H), 7.70–7.80 (m, 4H, Ar–H), 8.39 (s, 1H, C=CH̲ olefinic), 11.98 (s, 1H, NH, D_2_O exchangeable); anal. calcd for C_19_H_16_ClN_3_OS (369.9): C, 61.70; H, 4.36; N, 11.36; found: C, 61.55; H, 4.26; N, 11.24%.

###### 4.1.2.c.3. 4-((*E*)-4-Hydroxybenzylidene)-2-(2-((*E*)-1-(*p*-tolyl)ethylidene)hydrazinyl) thiazol-5(4*H*)-one (4c)

Yellow crystals; mp 272–274 °C; IR (KBr, cm^−1^): 3420 (OH), 3170 (NH), 3035 (CH-aromatic), 2924 (CH aliphatic), 3170 (NH), 1689 (C=O), 1638 (C=N); ^1^H NMR (DMSO-*d*_*6*_) *δ* ppm: 2.49 (s, 3H, CH_3_), 3.85 (s, 3H, CH_3_), 6.82–6.86 (m, 4H, Ar–H), 7.56–7.63 (m, 4H, Ar–H), 8.25 (s, 1H, C=CH̲ olefinic), 9.94 (s, 1H, NH, D_2_O exchangeable), 11.83 (s, 1H, OH, D_2_O exchangeable); anal. calcd for C_19_H_17_N_3_O_2_S (351.4): C, 64.94; H, 4.88; N, 11.96; found: C, 64.75; H, 4.80; N, 11.85%.

###### 4.1.2.c.4. 4-((*E*)-2,4-Dichlorobenzylidene)-2-(2-((*E*)-1-(*p*-tolyl)ethylidene)hydrazinyl) thiazol-5(4*H*)-one (4d)

White crystals; mp > 300 °C; IR (KBr, cm^−1^): 3115 (NH), 3066 (CH-aromatic), 2945 (CH aliphatic), 1720 (C=O), 1644 (C=N); ^1^H NMR (DMSO-*d*_*6*_) *δ* ppm: 3.30 (s, 3H, CH_3_), 3.98 (s, 3H, CH_3_), 7.47 (d, 2H, Ar–H), 7.74 (d, 2H, Ar–H), 7.94 (d, 2H, Ar–H, *J* = 8.4), 8.65 (s, 1H, C=CH̲ olefinic), 8.66 (s, 1H of Ar–H), 12.09 (s, 1H, NH, D_2_O exchangeable); anal. calcd for C_19_H_15_Cl_2_N_3_OS (404.3): C, 56.44; H, 3.74; N, 10.39; found: C, 56.28; H, 3.65; N, 10.27%.

###### 4.1.2.c.5. 4-((*E*)-3,4-Dimethoxybenzylidene)-2-(2-((*E*)-1-(*p*-tolyl)ethylidene)hydrazinyl) thiazol-5(4*H*)-one (4e)

Off white crystals; mp 254–256 °C; IR (KBr, cm^−1^): 3114 (NH), 2949 (CH aliphatic), 1704 (C=O), 1648 (C=N); ^1^H NMR (DMSO-*d*_*6*_) *δ* ppm: 3.32 (s, 3H, CH_3_), 3.67 (s, 3H, CH_3_), 3.83 (s, 3H, OCH_3_), 3.88 (s, 3H, OCH_3_), 6.94–7.33 (m, 6H, Ar–H), 8.27 (s, 1H, C=CH̲ olefinic + s, 1H of Ar–H), 11.80 (s, 2H, NH, D_2_O exchangeable), ^13^C NMR (DMSO-*d*_*6*_) *δ* ppm: 178.2, 161.7, 160.4, 158.3, 158.1, 157.1, 153.5, 142.6, 142.0, 139.4, 130.1, 129.2 (2), 1284 (2), 112.3, 110.2, 56.6, 56.1, 33.4, 13.7; anal. calcd for C_21_H_21_N_3_O_3_S (395.5): C, 63.78; H, 5.35; N, 10.63; found: C, 63.59; H, 5.27; N, 10.50%.

###### 4.1.2.c.6. 4-((*E*)-2,4-Dihydroxybenzylidene)-2-(2-((*E*)-1-(*p*-tolyl)ethylidene)hydrazinyl) thiazol-5(4*H*)-one (4f)

Red crystals; mp over 300 °C; IR (KBr, cm^−1^): 3441 (OH), 3204 (NH), 3037 (CH-aromatic), 2935 (CH aliphatic), 1697 (C=O), 1625 (C=N); ^1^H NMR (DMSO-*d*_*6*_) *δ* ppm: 2.47 (s, 3H, CH_3_), 3.93 (s, 3H, CH_3_), 6.29–6.37 (m, 4H, Ar–H), 7.33 (d, 2H, Ar–H, *J* = 8.4), 8.47 (s, 1H, C=CH̲ olefinic + s, 1H of Ar–H), 10.08 (s, 1H, NH, for thiazole tautomer, D_2_O exchangeable), 11.02 (s, 1H, 2OH, D_2_O exchangeable), 11.94 (s, 1H, NH, for thiazole tautomer D_2_O exchangeable); ^13^C NMR (DMSO-*d*_*6*_) *δ* ppm: 174.0, 161.8 (2), 160.6, 158.7(2), 133.0, 132.1, 110.8, 110.0, 109.0(2), 108.4(2), 102.8 (2), 102.0, 40.5, 19.7; anal. calcd for C_19_H_17_N_3_O_3_S (367.4): C, 62.11; H, 4.66; N, 11.44; found: C, 61.89; H, 4.57; N, 11.31%.

###### 4.1.2.c.7. 2-(2-((*E*)-1-(3,4-Dimethoxyphenyl)ethylidene)hydrazinyl)-4-((*E*)-4-methoxy benzylidene) thiazol-5(4*H*)-one (4g)

White crystals; mp 240–242 °C; IR (KBr, cm^−1^): 3149 (NH), 3035 (CH-aromatic), 2964 (CH aliphatic), 1721 (C=O), 1621 (C=N); ^1^H NMR (DMSO-*d*_*6*_) *δ* ppm: 3.82 (s, 3H, CH_3_), 3.84 (s, 3H, OCH_3_), 3.85 (s, 3H, OCH_3_), 3.86 (s, 3H, OCH_3_), 6.96 (d, 2H, Ar–H, *J* = 8 Hz), 7.01 (s, 1H, Ar–H), 7.33 (d, 2H, Ar–H, *J* = 8 Hz), 7.47 (d, 1H, Ar–H, *J* = 8 Hz), 7.68 (d, 1H, Ar–H, *J* = 8 Hz), 8.31(s, 1H, C=CH̲ olefinic), 11.87 (s, 1H, NH, D_2_O exchangeable); ^13^C NMR (DMSO-*d*_*6*_) *δ* ppm: 160.1, 152.3, 151.0, 148.8, 130.8(2), 129.7, 123.5, 120.3(2), 114.7, 111.5(2), 111.2, 110.6, 109.6 (2), 55.9, 55.7, 55.6, 14.2; anal. calcd for C_21_H_21_N_3_O_4_S (411.5): C, 61.30; H, 5.14; N, 10.21; found: C, 61.08; H, 5.04; N, 10.11%.

###### 4.1.2.c.8. 2-(2-((*E*)-1-(3,4-Dimethoxyphenyl)ethylidene)hydrazinyl)-4-((*E*)-4-hydroxy benzylidene)thiazol-5(4*H*)-one (4h)

Yellow crystals; mp 290–292 °C; IR (KBr, cm^−1^): 3231 (NH), 2924 (CH aliphatic), 1687 (CO), 1639 (C=N); ^1^H NMR (DMSO-*d*_*6*_) *δ* ppm: 3.43 (s, 3H, CH_3_), 3.82 (s, 3H, CH_3_), 3.84 (s, 3H, CH_3_), 6.81 (d, 2H, Ar–H, *J* = 8.4 Hz), 6.97 (d, 2H, Ar–H, *J* = 8.4 Hz), 7.35–7.57 (m, 3H, Ar–H), 8.25 (s, 1H, C=CH̲ olefinic), 9.96 (s, 1H, NH, D_2_O exchangeable), 11.87 (s, 1H, OH, D_2_O exchangeable); anal. calcd for C_20_H_19_N_3_O_4_S (397.4): C, 60.44; H, 4.82; N, 10.57; found: C, 60.24; H, 4.73; N, 10.46%.

###### 4.1.2.c.9. 4-((*E*)-2,4-Dichlorobenzylidene)-2-(2-((*E*)-1-(3,4-dimethoxyphenyl)ethylid-ene)hydrazineyl) thiazol-5(4*H*)-one (4i)

Off white crystals; mp > 300 °C; IR (KBr, cm^−1^): 3114, (NH), 3066 (CH-aromatic), 2944 (CH aliphatic), 1721 (CO), 1644 (C=N); ^1^H NMR (DMSO-*d*_*6*_) *δ* ppm: 3.78 (s, 3H, CH_3_), 3.82 (s, 3H, OCH_3_), 3.91 (s, 3H, OCH_3_), 7.51–7.62 (m, 3H, Ar–H), 7.94 (m, 2H, Ar–H, *J* = 8.4 Hz), 8.56 (s, 1H, C=CH̲ olefinic + 1H, Ar–H), 12.06 (s, 1H, NH, D_2_O exchangeable); anal. calcd for C_20_H_17_C_l2_N_3_O_3_S (450.3): C, 53.34; H, 3.81; N, 9.33; found: C, 53.15; H, 3.72; N, 9.22%.

###### 4.1.2.c.10. 4-((*E*)-3,4-Dimethoxybenzylidene)-2-(2-((*E*)-1-(3,4-dimethoxyphenyl)ethylid-ene)hydrazinyl)thiazol-5(4*H*)-one (4j)

Yellow crystals; mp 232–236 °C; IR (KBr, cm^−1^): 3433, (NH), 2944 (CH aliphatic), 1707 (CO), 1648 (C=N); ^1^H NMR (DMSO-*d*_*6*_) *δ* ppm: 3.61 (s, 3H, CH_3_), 3.74 (s, 3H, OCH_3_), 3.79 (s, 3H, OCH_3_), 3.81 (s, 3H, OCH_3_), 3.86 (s, 3H, OCH_3_), 6.99 (d, 1H, Ar–H, *J* = 8 Hz), 7.05–7.15 (m, 3H, Ar–H), 7.55 (s, 1H, Ar–H), 8.29 (s, 1H, C=CH̲ olefinic + 1H, Ar–H), 11.91 (s, 1H, NH, D_2_O exchangeable); anal. calcd for C_22_H_23_N_3_O_5_S (441.5): C, 59.85; H, 5.25; N, 9.52; found: C, 59.66; H, 5.17; N, 9.60%.

###### 4.1.2.c.11. 4-((*E*)-2,4-Dihydroxybenzylidene)-2-(2-((*E*)-1-(3,4-dimethoxyphenyl)ethylid-ene)hydrazinyl)thiazol-5(4*H*)-one (4k)

Red crystals; mp > 300 °C; IR (KBr, cm^−1^): 3214, (NH), 3053 (CH-aromatic), 2975 (CH aliphatic), 1697 (CO), 1633 (C=N); ^1^H NMR (DMSO-*d*_*6*_) *δ* ppm: 3.38 (s, 3H, CH_3_), 3.77 (s, 3H, OCH_3_), 3.86 (s, 3H, OCH_3_), 6.29–6.38 (m, 3H, Ar–H), 7.31 (d, 2H, Ar–H), 8.47 (s, 1H, C=CH olefinic + 1H, Ar–H), 10.08 (s, 1H, NH, for thiazol tautomer, D_2_O exchangeable), 11.02 (s, 2H, OH, D_2_O exchangeable), 11.95 (s, 1H, NH, for thiazole tautomer, D_2_O exchangeable); anal. calcd for C_20_H_19_N_3_O_5_S (413.4): C, 58.10; H, 4.63; N, 10.16; found: C, 57.26; H, 4.55; N, 10.06%.

###### 4.1.2.c.12. (*E*)-4-(Thiophen-2-ylmethylene)-2-(2-((*E*)-1-(*p*-tolyl)ethylidene)hydrazinyl) thiazol-5(4*H*)-one (5a)

Orange crystals; mp 202–204 °C; IR (KBr, cm^−1^): 3122 (NH), 3051 (CH-aromatic), 2950 (CH aliphatic), 1707 (C=O), 1634 (C=N); ^1^H NMR (DMSO-*d*_*6*_) *δ* ppm: 3.86 (s, 6H, 2CH_3_), 7.06 (d, 2H, Ar–H, *J* = 8), 7.14 (m, 1H, Ar–H), 7.26 (d, 2H, Ar–H, *J* = 8), 7.56 (d, 1H, Ar–H), 7.73 (m, 1H, Ar–H), 8.66 (s, 1H, C=CH̲ olefinic), 11.75 (s, 1H, NH, D_2_O exchangeable); anal. calcd for C_17_H_15_N_3_OS_2_ (341.4): C, 59.80; H, 4.43; N, 12.31; found: C, 59.69; H, 4.35; N, 12.19%.

###### 4.1.2.c.13. (*E*)-2-(2-((*E*)-1-(3,4-Dimethoxyphenyl)ethylidene)hydrazinyl)-4-(thiophen-2-ylmethylene)thiazol-5(4*H*)-one (5b)

Pale yellow crystals; mp 208–210 °C; IR (KBr, cm^−1^): 3411, (NH), 3053 (CH-aromatic), 2951 (CH aliphatic), 1709 (CO), 1633 (C=N); ^1^H NMR (DMSO-*d*_*6*_) *δ* ppm: 3.79 (s, 3H, CH_3_), 3.82 (s, 3H, OCH_3_), 3.85 (s, 3H, OCH_3_), 7.13–7.18 (m, 2H, Ar–H), 7.47–7.48 (d, 1H, Ar–H), 7.67 (d, 2H, Ar–H, *J* = 5.2 Hz), 8.52 (s, 1H, C=CH̲ olefinic + 1H, Ar–H),11.90 (s, 1H, NH, D_2_O exchangeable); anal. calcd for C_18_H_17_N_3_O_3_S_2_ (387.5): C, 55.80; H, 4.42; N, 10.84; found: C, 55.69; H, 4.33; N, 10.72%.

###### 4.1.2.c.14. 4-((*E*)-4-Methoxybenzylidene)-2-(2-((*E*)-1-(thiophen-2-yl)ethylidene)hydra-zinyl)thiazol-5(4*H*)-one (8a)

White crystals; mp 243–245 °C; IR (KBr, cm^−1^): 3287, 3149, (NH), 3075 (CH-aromatic), 2934 (CH aliphatic), 1709 (C=O), 1605 (C=N); ^1^H NMR (DMSO-*d*_*6*_) *δ* ppm: 3.77 (s, 3H, CH_3_), 3.85 (s, 3H, OCH_3_), 6.98–7.02 (m, 3H, Ar–H), 7.66–7.69 (m, 4H, Ar–H), 8.31 (s, 1H, C=CH̲ olefinic), 11.74 (s, 1H, NH, D_2_O exchangeable); anal. calcd for C_17_H_15_N_3_O_2_S_2_ (357.4): C, 57.12; H, 4.23; N, 11.76; found: C, 56.90; H, 4.16; N, 11.64%.

###### 4.1.2.c.15. 4-((*E*)-4-Chlorobenzylidene)-2-(2-((*E*)-1-(thiophen-2-yl)ethylidene)hydra-zinyl)thiazol-5(4*H*)-one (8b)

Orange crystals; mp 252–254 °C; IR (KBr, cm^−1^): 3145 (NH), 3043 (CH-aromatic), 2936 (CH aliphatic), 1714 (C=O), 1640 (C=N); ^1^H NMR (DMSO-*d*_*6*_) *δ* ppm: 3.93 (s, 3H, CH_3_), 7.56–7.70 (m, 3H, Ar–H), 7.83–8.28 (m, 3H, Ar–H), 8.94 (s, 1H, C=CH̲ olefinic H), 9.02 (dd, 1H, Ar–H), 12.03 (s, 1H, NH, D_2_O exchangeable); ^13^C NMR (DMSO-*d*_*6*_) *δ* ppm: 163.2, 153.2, 139.2, 138.3, 138.1, 137.4, 137.2, 136.4, 135.3, 130.1(2), 129.1(2), 121.2, 120.9, 14.1; anal. calcd for C_16_H_12_ClN_3_OS_2_ (361.9): C, 53.11; H, 3.34; N, 11.61; found: C, 52.99; H, 3.26; N, 11.50%.

###### 4.1.2.c.16. 4-((*E*)-4-Hydroxybenzylidene)-2-(2-((*E*)-1-(thiophen-2-yl)ethylidene)hydra-zinyl)thiazol-5(4*H*)-one (8c)

Orange crystals; mp 178–180 °C; IR (KBr, cm^−1^): 3235 (OH), 3181 (NH), 3035 (CH-aromatic), 2964 (CH aliphatic), 1688 (C=O), 1641 (C=N); ^1^H NMR (DMSO-*d*_*6*_) *δ* ppm: 3.84 (s, 3H, CH_3_), 6.80–6.82 (m, 4H, Ar–H), 7.55–7.57 (m, 3H, Ar–H), 8.24 (s, 1H, C=CH̲ olefinic), 9.96 (s, 1H, NH, D_2_O exchangeable), 11.82 (s, 1H, OH, D_2_O exchangeable); anal. calcd for C_16_H_13_N_3_O_2_S_2_ (343.4): C, 55.96; H, 3.82; N, 12.24; found: C, 55.79; H, 3.75; N, 12.11%.

###### 4.1.2.c.17. 4-((*E*)-2,4-Dichlorobenzylidene)-2-(2-((*E*)-1-(thiophen-2-yl)ethylidene) hydrazinyl)thiazol-5(4*H*)-one (8d)

Pale yellow crystals; mp 274–276 °C; IR (KBr, cm^−1^): 3115 (NH), 3066 (CH-aromatic), 2946 (CH aliphatic), 1720 (C=O), 1643 (C=N); ^1^H NMR (DMSO-*d*_*6*_) *δ* ppm: 3.90 (s, 3H, CH_3_), 7.51–7.53 (dd, 2H, Ar–H, *J* = 8.4 Hz), 7.71–7.72 (m, 2H, Ar–H), 7.93–7.95 (m, 2H, Ar–H), 8.56 (s, 1H, C=CH̲ olefinic), 12.08 (s, 1H, NH, D_2_O exchangeable); anal. calcd for C_16_H_11_C_l2_N_3_OS_2_ (396.3): C, 48.49; H, 2.80; N, 10.60; found: C, 48.34; H, 2.71; N, 10.50%.

###### 4.1.2.c.18. 4-((*E*)-3,4-Dimethoxybenzylidene)-2-(2-((*E*)-1-(thiophen-2-yl)ethylidene) hydrazinyl) thiazol-5(4*H*)-one (8e)

Buff crystals; mp 248–250 °C; IR (KBr, cm^−1^): 3122, (NH), 3056 (CH-aromatic), 2943 (CH aliphatic), 1703 (C=O), 1648 (C=N); ^1^H NMR (DMSO-*d*_*6*_) *δ* ppm: 3.75 (s, 3H, CH_3_), 3.84 (s, 6H, OCH_3_), 6.97 (d, 2H, Ar–H, *J* = 8 Hz), 7.07 (s, 1H, Ar–H), 7.14 (d, 2H, Ar–H), 7.53–7.54 (m, 1H, Ar–H), 8.40 (s, 1H, C=CH̲ olefinic), 12.56 (s, 1H, NH, D_2_O exchangeable); anal. calcd for C_18_H_17_N_3_O_3_S_2_ (387.5): C, 55.80; H, 4.42; N, 10.84; found: C, 55.66; H, 4.31; N, 10.77%.

###### 4.1.2.c.19. 4-((*E*)-2,4-Dihydroxybenzylidene)-2-(2-((*E*)-1-(thiophen-2-yl)ethylidene) hydrazinyl) thiazol-5(4*H*)-one (8f)

Red crystals; mp > 300 °C; IR (KBr, cm^−1^): 3208 (OH, NH), 3092 (CH-aromatic), 2975 (CH aliphatic), 1697 (C=O), 1625 (C=N); ^1^H NMR (DMSO-*d*_*6*_) *δ* ppm: 3.92 (s, 3H, CH_3_), 6.29–6.34 (m, 1H, Ar–H), 6.34–6.36 (dd, 2H, Ar–H, *J* = 8.4 Hz), 7.21–7.33 (d, 2H, Ar–H), 8.40 (s, 1H, C=CH olefinic + 1H, Ar–H), 10.07 (s, 1H, NH, for thiazole tautomer D_2_O exchangeable), 11.02 (s, 2H, OH, D_2_O exchangeable), 11.94 (s, 1H, NH, for thiazole tautomer, D_2_O exchangeable); anal. calcd for C_16_H_13_N_3_O_3_S_2_ (359.4): C, 53.47; H, 3.65; N, 11.69; found: C, 53.25; H, 3.53; N, 11.58%.

###### 4.1.2.c.20. 2-(2-((*E*)-1-(Furan-2-yl)ethylidene)hydrazinyl)-4-((*E*)-4-methoxybenzylid-ene)thiazol-5(4*H*)-one (8g)

Yellow crystals; mp 288–290 °C; IR (KBr, cm^−1^): 3434 (NH), 2972 (CH aliphatic), 1722 (C=O), 1634 (C=N), 1602 (C=C); ^1^H NMR (DMSO-*d*_*6*_) *δ* ppm: 3.83 (s, 3H, CH_3_), 3.85 (s, 3H, OCH_3_), 6.98–7.01 (m, 3H, Ar–H), 7.66–7.69 (m, 4H, Ar–H), 8.31 (s, 1H, C=CH̲ olefinic), 11.88 (s, 1H, NH, D_2_O exchangeable); anal. calcd for C_17_H_15_N_3_O_3_S (341.4): C, 59.81; H, 4.43; N, 12.31; found: C, 59.69; H, 4.34; N, 12.20%.

###### 4.1.2.c.21. 4-((*E*)-4-Chlorobenzylidene)-2-(2-((*E*)-1-(furan-2-yl)ethylidene)hydra-zinyl)thiazol-5(4*H*)-one (8h)

Grye crystals; mp 272–274 °C; IR (KBr, cm^−1^): 3199 (NH), 3027 (CH-aromatic), 2938 (CH aliphatic), 1710 (C=O), 1643 (C=N); ^1^H NMR (DMSO-*d*_*6*_) *δ* ppm: 3.87 (s, 3H, CH_3_), 7.49–7.52 (m, 4H, Ar–H), 7.73–7.80 (m, 3H, Ar–H), 8.39 (s, 1H, C=CH̲ olefinic), 11.97 (s, 1H, NH, D_2_O exchangeable); ^13^C NMR (DMSO-*d*_*6*_) *δ* ppm: 162.1, 155.5, 143.1, 142.2, 139.1, 138.0, 136.0, 133.7, 129.6, 129.4, 128.2, 127.4 (2), 111.4, 110.0, 11.3; anal. calcd for C_16_H_12_ClN_3_O_2_S (345.8): C, 55.57; H, 3.50; N, 12.15; found: C, 55.35; H, 3.41; N, 12.04%.

###### 4.1.2.c.22. 2-(2-((*E*)-1-(Furan-2-yl)ethylidene)hydrazinyl)-4-((*E*)-4-hydroxybenzylid-ene)thiazol-5(4*H*)-one (8i)

Green crystals; mp 293–295 °C; IR (KBr, cm^−1^): 3238 (OH), 3155 (NH), 3033 (CH-aromatic), 2922 (CH aliphatic), 1689 (C=O), 1639 (C=N); ^1^H NMR (DMSO-*d*_*6*_) *δ* ppm: 3.81 (s, 3H, CH_3_), 6.76–6.82 (m, 4H, Ar–H), 7.55–7.57 (m, 1H, Ar–H), 7.58–7.60 (d, 2H, Ar–H, *J* = 8 Hz), 8.25 (s, 1H, C=CH̲ olefinic), 9.96 (s, 1H, NH, D_2_O exchangeable), 11.82 (s, 1H, OH, D_2_O exchangeable); ^13^C NMR (DMSO-*d*_*6*_) *δ* ppm: 160.3, 159.0, 156.8, 151.3, 153.4, 153.1, 149.1, 148.5, 134.3, 129.7(2), 125.5(2), 116.2, 115.6, 11.4; anal. calcd for C_16_H_13_N_3_O_3_S (327.4): C, 58.71; H, 4.00; N, 12.84; Found: C, 58.56; H, 3.88; N, 12.71%.

###### 4.1.2.c.23. 4-((*E*)-2,4-Dichlorobenzylidene)-2-(2-((*E*)-1-(furan-2-yl)ethylidene) hydrazinyl) thiazol-5(4*H*)-one (8j)

White crystals; mp 264–266 °C; IR (KBr, cm^−1^): 3159 (NH), 3066 (CH-aromatic), 2936 (CH aliphatic), 1720 (C=O), 1644 (C=N); ^1^H NMR (DMSO-*d*_*6*_) *δ* ppm: 3.91 (s, 3H, CH_3_), 7.51–7.53 (dd, 2H, Ar–H, *J* = 8.4 Hz), 7.72 (d, 1H, Ar–H), 7.93–7.95 (m, 2H, Ar–H), 8.56 (s, 1H, C=CH̲ olefinic + 1H, Ar–H), 12.08 (s, 1H, NH, D_2_O exchangeable); anal. calcd for C_16_H_11_C_l2_N_3_O_2_S (380.2): C, 50.54; H, 2.92; N, 11.05; found: C, 50.36; H, 2.81; N, 10.94%.

###### 4.1.2.c.24. 4-((*E*)-3,4-Dimethoxybenzylidene)-2-(2-((*E*)-1-(furan-2-yl)ethylidene)hydra-zinyl)thiazol-5(4*H*)-one (8k)

White crystals; mp 240–242 °C; IR (KBr, cm^−1^): 3441 (NH), 3088 (CH-aromatic), 2919 (CH aliphatic), 1706 (C=O), 1648 (C=N); ^1^H NMR (DMSO-*d*_*6*_) *δ* ppm: 3.74 (s, 3H, CH_3_), 3.82 (s, 3H, OCH_3_), 3.83 (s, 3H, OCH_3_), 7.07 (d, 2H, Ar–H, *J* = 8.4 Hz), 7.27 (m, 3H, Ar–H), 7.51 (m, 1H, Ar–H), 8.29 (s, 1H, C=CH̲ olefinic), 11.91 (s, 1H, NH, D_2_O exchangeable); anal. calcd for C_18_H_17_N_3_O_4_S (371.4): C, 58.21; H, 4.61; N, 11.31; found: C, 58.00; H, 4.50; N, 11.40%.

###### 4.1.2.c.25. 4-((*E*)-2,4-Dihydroxybenzylidene)-2-(2-((*E*)-1-(furan-2-yl)ethylidene) hydrazinyl) thiazol-5(4*H*)-one (8l)

Gray crystals; mp 282–284 °C; IR (KBr, cm^−1^): 3411 (OH), 3150 (NH), 3055 (CH-aromatic), 2952 (CH aliphatic), 1708 (C=O), 1632 (C=N); ^1^H NMR (DMSO-*d*_*6*_) *δ* ppm: 3.92 (s, 3H, CH_3_), 6.29–6.34 (m, 1H, Ar–H), 6.34–6.36 (d, 2H, Ar–H, *J* = 8.4 Hz), 7.31–7.37 (m, 2H, Ar–H), 8.47 (s, 1H, C=CH olefinic + 1H, Ar–H), 10.07 (s, 1H, NH, for thiazole tautomer, D_2_O exchangeable), 11.02 (s, 1H, 2OH, D_2_O exchangeable), 11.94 (s, 1H, NH, D_2_O exchangeable, for thiazole tautomer); anal. calcd for C_16_H_13_N_3_O_4_S (343.4): C, 55.97; H, 3.82; N, 12.24; found: C, 55.77; H, 3.73; N, 12.14%.

###### 4.1.2.c.26. (*E*)-2-(2-((*E*)-1-(Thiophen-2-yl)ethylidene)hydrazinyl)-4-(thiophen-2-ylmethylene)thiazol-5(4*H*)-one (9a)

Orang crystals; mp 214–216 °C; IR (KBr, cm^−1^): 3425 (NH), 1717 (C=O), 1635 (C=N); ^1^H NMR (DMSO-*d*_*6*_) *δ* ppm: 3.93 (s, 3H, CH_3_), 7.56–7.65 (m, 3H, Ar–H), 7.94–8.08 (dd, 2H, Ar–H), 8.95 (s, 1H, C=CH̲ olefinic H), 9.03 (dd, 1H, Ar–H), 12.03 (s, 1H, NH, D_2_O exchangeable); anal. calcd for C_14_H_11_N_3_OS_3_ (333.4): C, 50.43; H, 3.33; N, 12.60; found: C, 50.23; H, 3.40; N, 12.51%.

###### 4.1.2.c.27. (*E*)-2-(2-((*E*)-1-(Furan-2-yl)ethylidene)hydrazinyl)-4-(thiophen-2-ylmethylene) thiazol-5(4*H*)-one (9b)

Yellow crystals; mp 203–205 °C; IR (KBr, cm^−1^): 3216 (NH), 3091 (CH-aromatic), 2937 (CH aliphatic), 1696 (C=O), 1622 (C=N); ^1^H NMR (DMSO-*d*_*6*_) *δ* ppm: 3.85 (s, 3H, CH_3_), 7.12–7.14 (m, 2H, Ar–H), 7.47–7.48 (d, 2H, Ar–H), 7.66–7.67 (d, 2H, Ar–H), 8.53 (s, 1H, C=CH̲ olefinic), 11.90 (s, 1H, NH, D_2_O exchangeable); anal. calcd for C_14_H_11_N_3_O_2_S_2_ (317.4): C, 52.98; H, 3.49; N, 13.24; found: C, 52.77; H, 3.37; N, 13.13%.

### 4.2. Biological evaluation

#### 4.2.1. *In vitro* cytotoxic activity

Evalation of cytotoxic activity of the synthesized compounds was carried out using MTT assay protocol^49,50,57^ against a group of cancer cell lines namely; colorectal carcinoma (HCT-116), Hepatocellular carcinoma (HepG-2) and breast cancer (MCF-7) and colchicine was used as a standard drug. The cells were obtained from ATCC (American Type Culture Collection) *via* the Holding company for biological products and vaccines (VACSERA) (Cairo, Egypt). The anti-cancer activity was measured quantitatively as follows:

Into a medium of RPMI-1640 with 10% fetal bovine serum, the cell lines were cultured. Then, penicillin (100 units per mL) and streptomycin (100 μg mL^−1^) were added at 37 °C in a 5% CO_2_ incubator. Next, seeding the cell lines in a 96-well plate was achieved by a density of 1.0 × 10^4^ cells per well at 37 °C for 48 h under 5% CO_2_. After incubation period, the cell lines were treated with different concentration of the synthesized compounds and incubated for 24 h. After treatment by 24 h, 20 μL of MTT solution (5 mg mL^−1^) was added and incubated for 4 h. Dimethyl sulfoxide (100 μL) was added into each well to dissolve the formed purple formazan. The colorimetric assay was measured and recorded at absorbance of 570 nm using a plate reader (EXL 800, USA). The relative cell viability in percentage was calculated as (A570 of treated samples/A570 of untreated sample) X 100. Results for IC_50_ values of the active compounds were summarized in Table 1.

#### 4.2.2. *In vitro* tubulin polymerization assay

The effect of the synthesized compounds on tubulin polymerization was assessed turbidimetrically using a fluorescent plate reader method.^52^ At first, the synthesized compounds and reference drug (colchicine) were incubated in mixture of purified bovine tubulin (10 μM) and buffer system containing 20% glycerol and 1 mM ATP at 37 °C. Then, the mixture cooled to 0 °C. The IC_50_ value was defined as the compound concentration that inhibited the extent of tubulin assembly by 50%.

#### 4.2.3. Cell cycle analysis

HepG-2 cells were seeded at density of 2 × 10^5^ cells per well and incubated for 24 h in six-well plates. Fetal bovine serum (FBS, 10%) was added, after that cells were incubated at 37 °C and 5% CO_2_. The medium was replaced with (DMSO 1% v/v) containing the 2.5 μM of compound 5a, then incubated for 48 h, washed with cold phosphate buffered saline (PBS), fixed with 70% ethanol, rinsed with PBS then stained with the DNA fluorochrome PI, kept for 15 min at 37 °C. Then samples were analyzed with a FACS Caliber flow cytometer.^53^

#### 4.2.4. Annexin V-FITC apoptosis assay

The effect of the most cytotoxic compound 5a on apoptosis induction was analyzed using Annexin V-FITC/PI apoptosis detection kit. In this test, HepG-2 cells were stained with Annexin V fluorescein isothiocyanate (FITC) and counterstained with propidium iodide (PI). Then, HepG-2 cells in a density of 2 × 10^5^ per well were incubated with compound 5a for 48 h. Next, the cells were trypsinized, washed with phosphate-buffered saline (PBS), and stained for 15 min at 37 °C in the dark. Finally, FACS Caliber flow cytometer was used in analysis process.^55^

### 4.3. Docking studies

Crystallographic structure of tubulin [PDB ID: 1SA0, resolution 3.00 Å] was retrieved from Protein Data Bank (http://www.pdb.org) and considered as a target for docking simulation. The docking analysis was performed using MOE2014 software to evaluate the free energy and binding modes of the synthesized compounds against tubulin. At first, the crystal structure of the target was prepared by removing water molecules and retaining the two essential chains and the co-crystallized ligand, *N*-deacetyl-*N*-(2-mercaptoacetyl)-colchicine (DAMA-colchicine). Then, the protein structure was protonated, and the hydrogen atoms were hided. Next, the energy was minimized, and the binding pocket of the protein was defined.

The 2D structures of the synthesized compounds and reference ligand (DAMA–colchicine) were sketched using ChemBioDraw Ultra 14.0 and saved as MDL-SD format. Then, the saved files were opened using MOE and 3D structures were protonated. Next, energy minimization was applied. Before docking process, validation of the docking protocol was carried out by running the simulation only using the co-crystallized ligand (DAMA–colchicine) which showed low RMSD value. The molecular docking of the synthesized was performed using a default protocol against the target receptor. In each case, 30 docked structures were generated using genetic algorithm searches, London dG was used for scoring and forcefield (MMFF94) for refinement. The London dG scoring function estimates the free energy of binding of the ligand from a given pose. The functional form is a sum of terms:
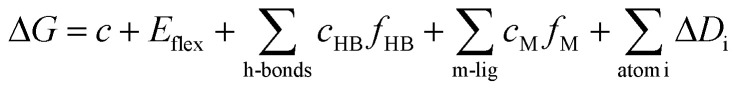
where *C* represents the average gain/loss of rotational and translational entropy; *E*_flex_ is the energy due to the loss of flexibility of the ligand (calculated from ligand topology only); FHB measures geometric imperfections of hydrogen bonds and takes a value in [0,1]; *c*_HB_ is the energy of an ideal hydrogen bond; *f*_M_ measures geometric imperfections of metal ligations and takes a value in [0,1]; *c*_M_ is the energy of an ideal metal ligation; and *D*_i_ is the desolvation energy of atom i.

The output from of MOE was further analyzed and visualized using Discovery Studio 4.0 software.^58–61^

## Conflicts of interest

This work was funded by the authors and there is no any conflict of interest.

## Supplementary Material

RA-010-C9RA10094F-s001
