# Supplementary material for: Design, eco-friendly synthesis, molecular modeling and anticancer evaluation of thiazol-5(4H)-ones as potential tubulin polymerization inhibitors targeting the colchicine binding site
Source: RSC Adv. 2020 Jan 15;10(5):2791–811. doi: 10.1039/c9ra10094f (PMC9048505; doi:10.1039/c9ra10094f)

Electronic Supplementary Material (ESI) for RSC Advances.
This journal is © The Royal Society of Chemistry 2020

Design, Eco-friendly synthesis, molecular modeling, and anticancer evaluation of thiazol-5(4*H*)-ones as potential tubulin polymerization inhibitors targeting colchicine binding site

Abeer M. El-Naggar*[a], Amira A. El-Sayed [a], Amany Belal [b], Ibrahim H. Eissa*[c]

[a] Chemistry Department, Faculty of science, Ain shams University, Abbassiya, Cairo, 11566, Egypt

[b] Medicinal Chemistry Department , Faculty of Pharmacy, Beni-Suef University, Beni-Suef 62415, Egypt

[c] Pharmaceutical Medicinal Chemistry & Drug Design Department, Faculty of Pharmacy (Boys), Al-Azhar University, Cairo, 11884, Egypt

*** Corresponding authors:**

Ibrahim H. Eissa

Pharmaceutical Medicinal Chemistry & Drug Design Department, Faculty of Pharmacy (Boys), Al-Azhar University, Cairo, 11884, Egypt

Email: Ibrahimeissa@azhar.edu.eg

Abeer M. El-Naggar

Chemistry Department, Faculty of science, Ain shams University, Abbassia, Cairo-11566, Egypt.

E-mail: elsayedam@sci.asu.edu.eg

IR of compound (3a)

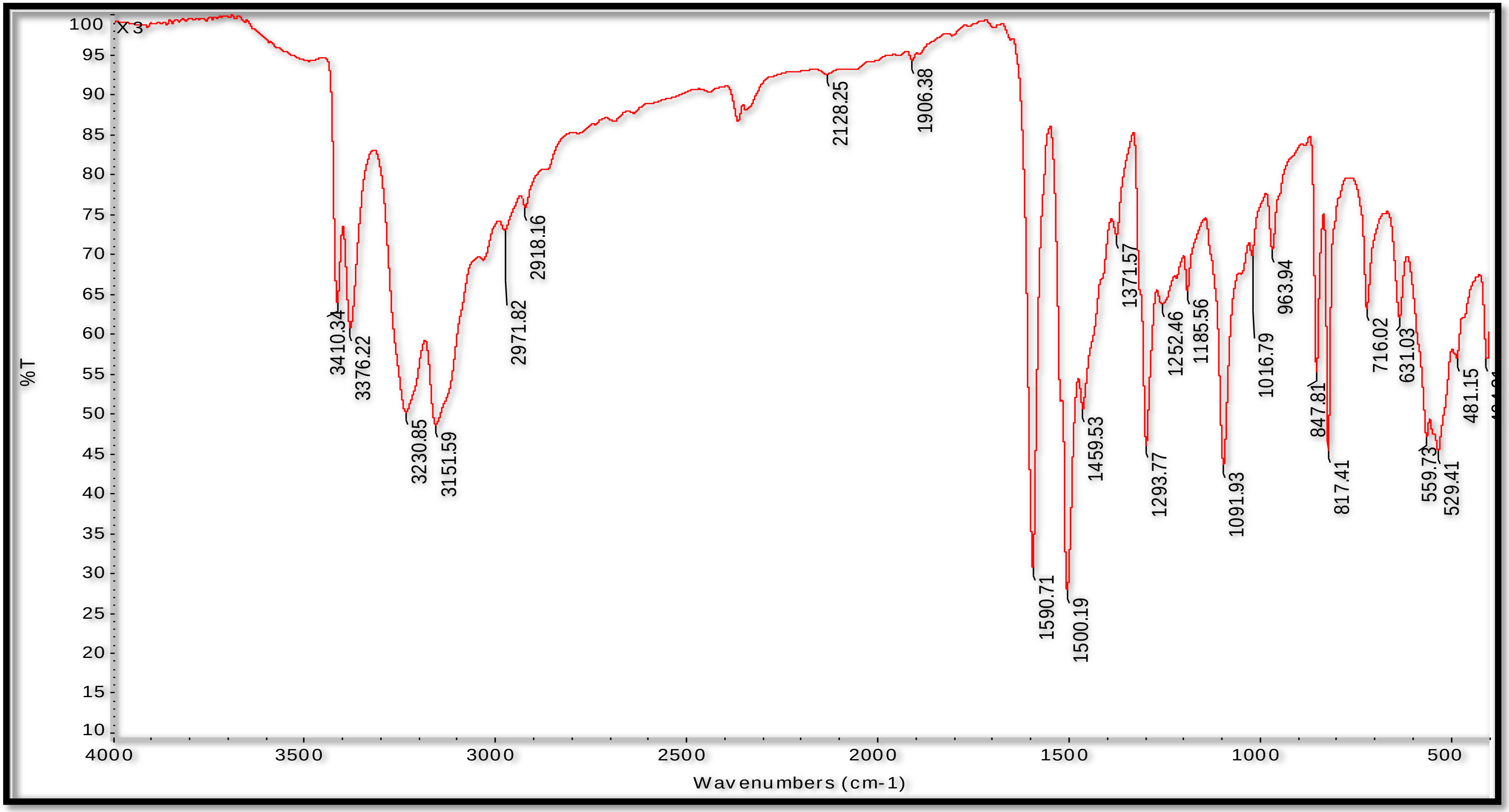

^{1}H NMR of compound (3a)

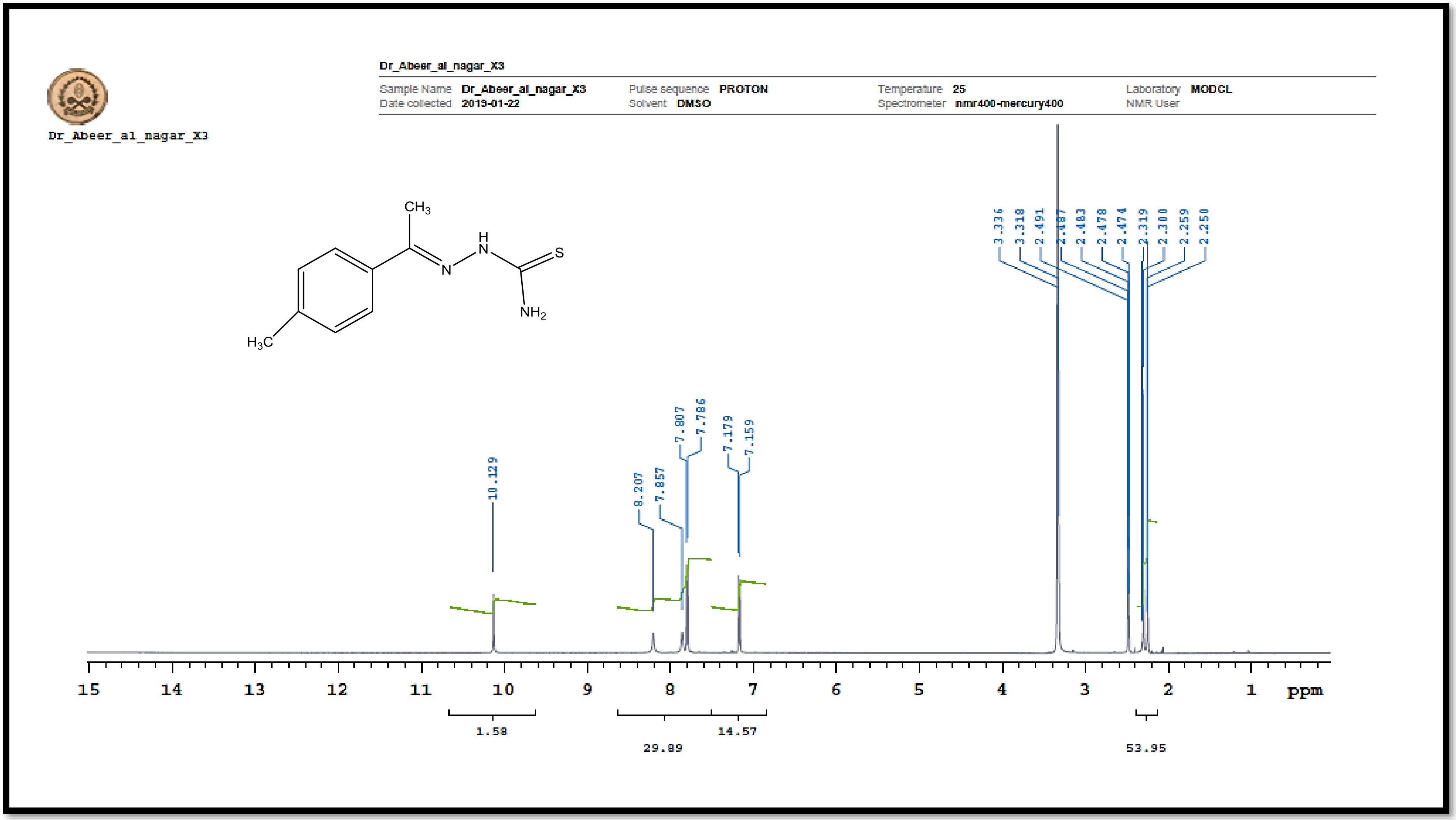

D₂O ¹H NMR of compound (3a)

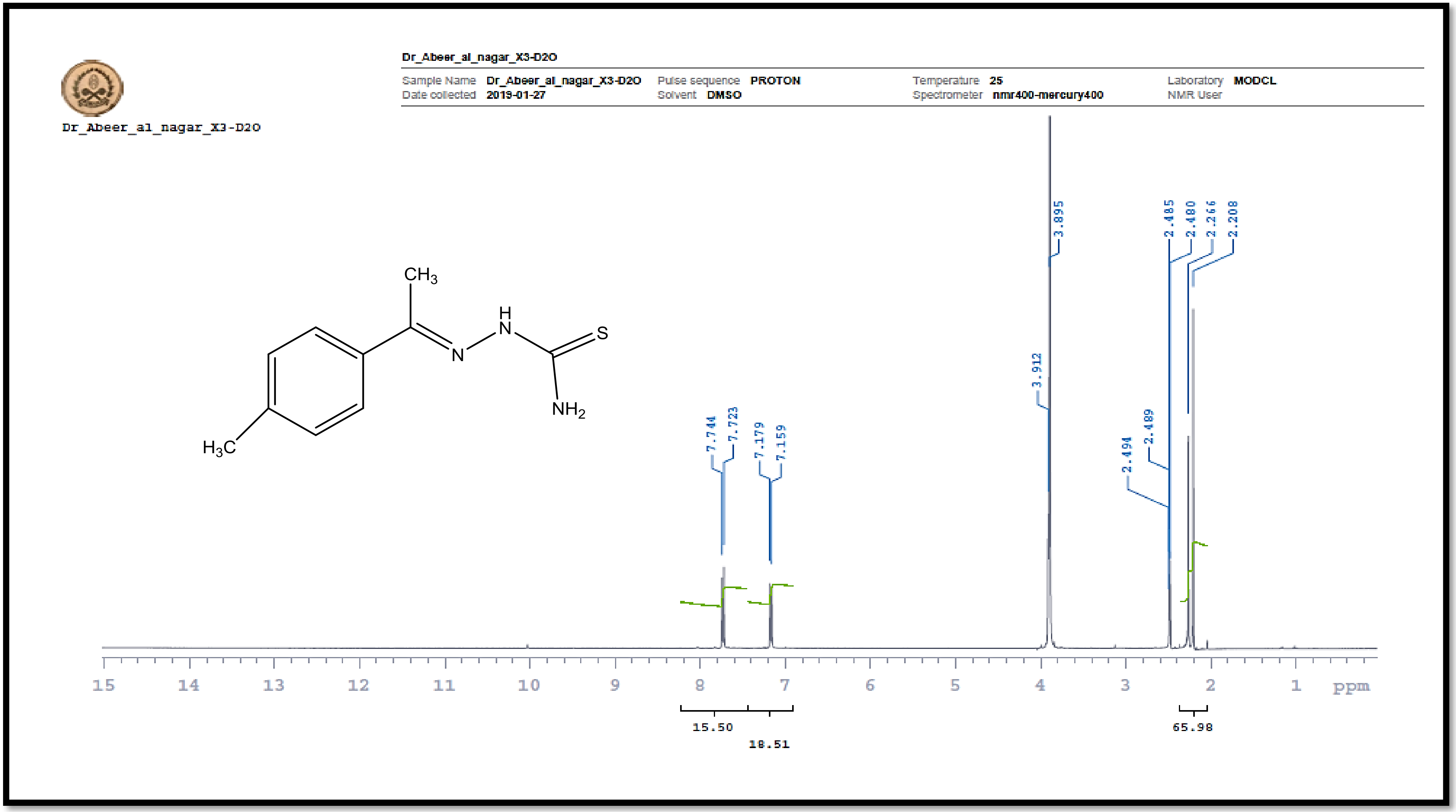

IR of compound (3b)

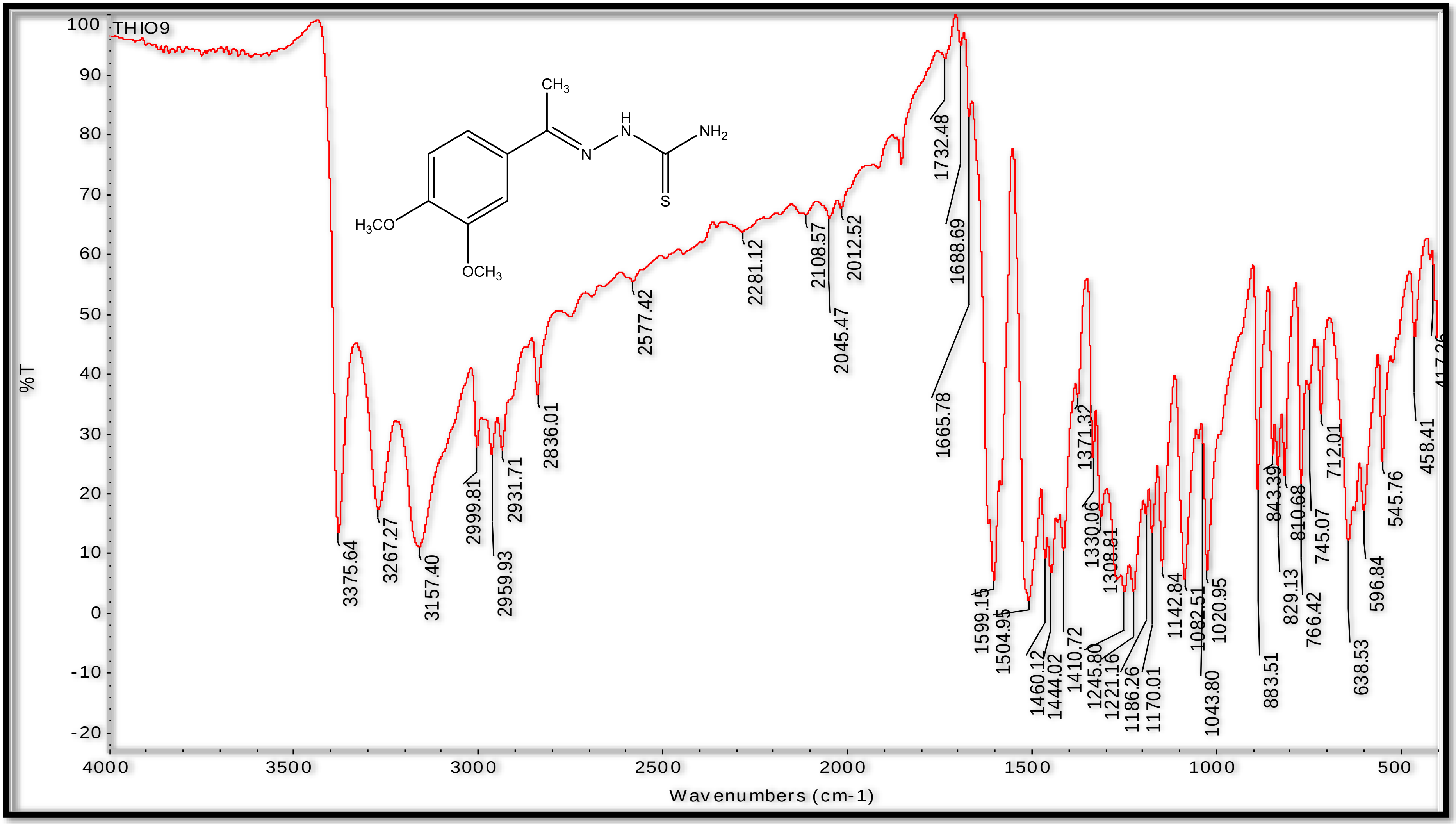

^{1}H NMR of compound (3b)

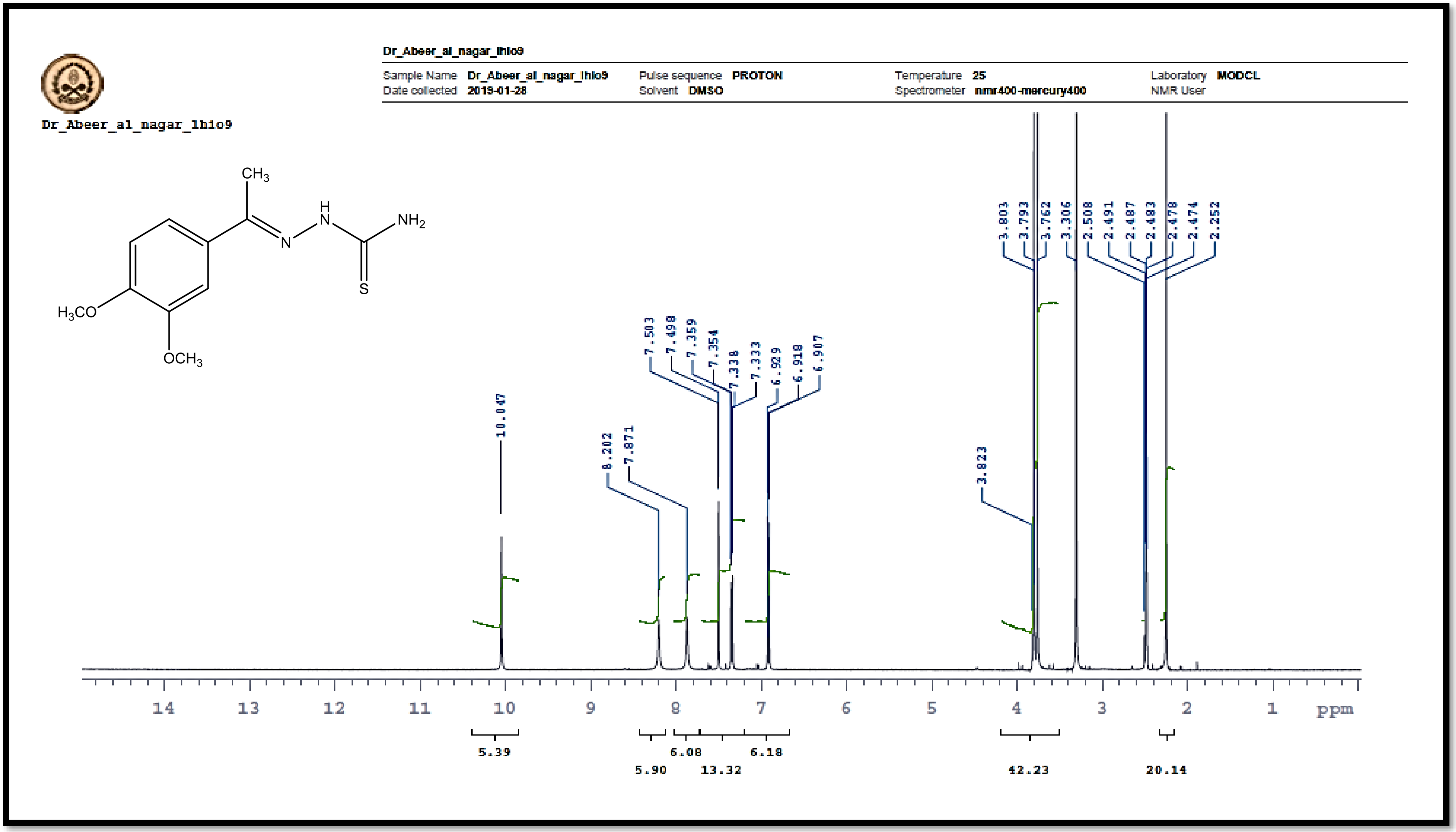

^{13}C NMR of compound (3b)

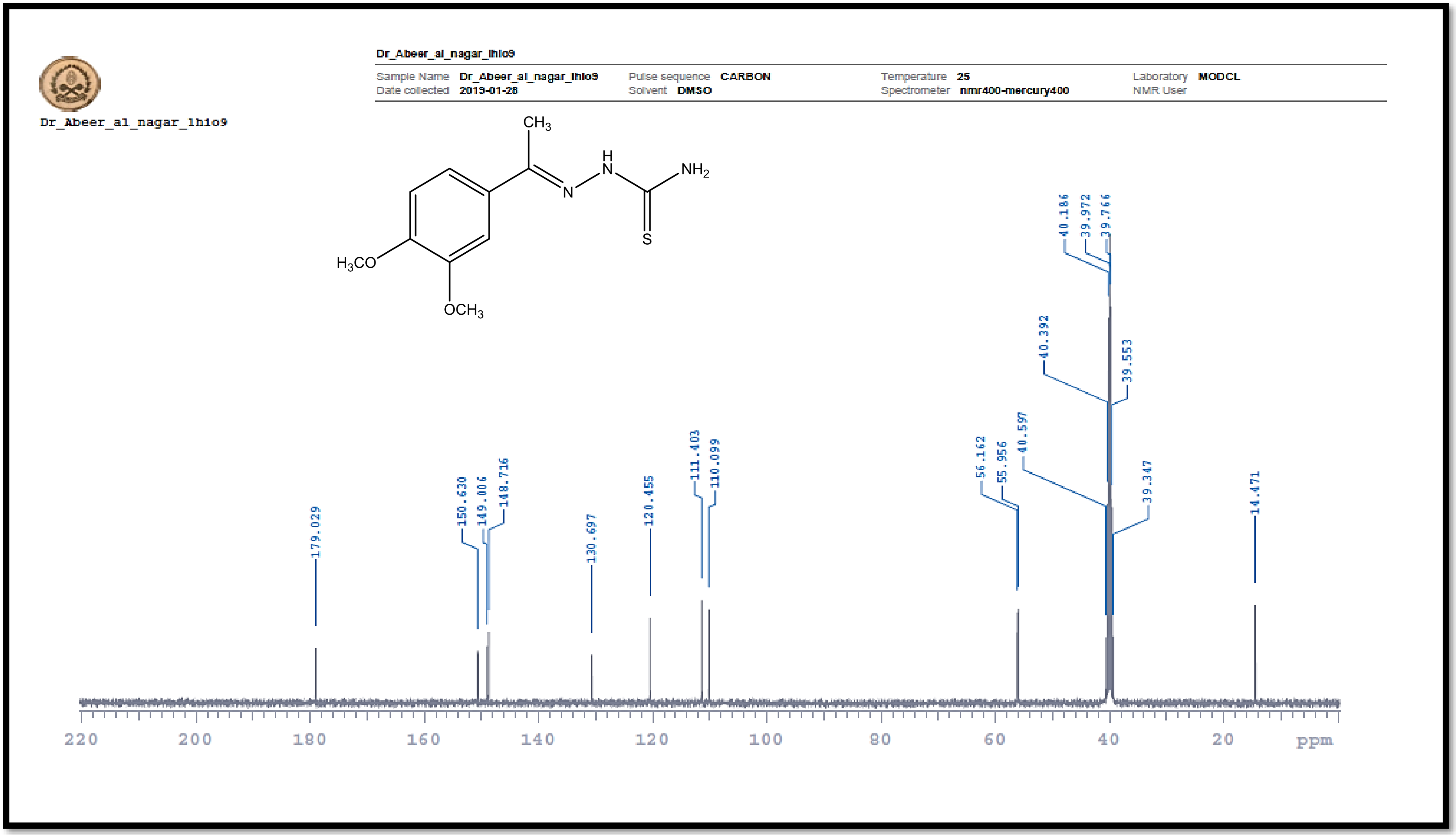

IR of compound (7a)

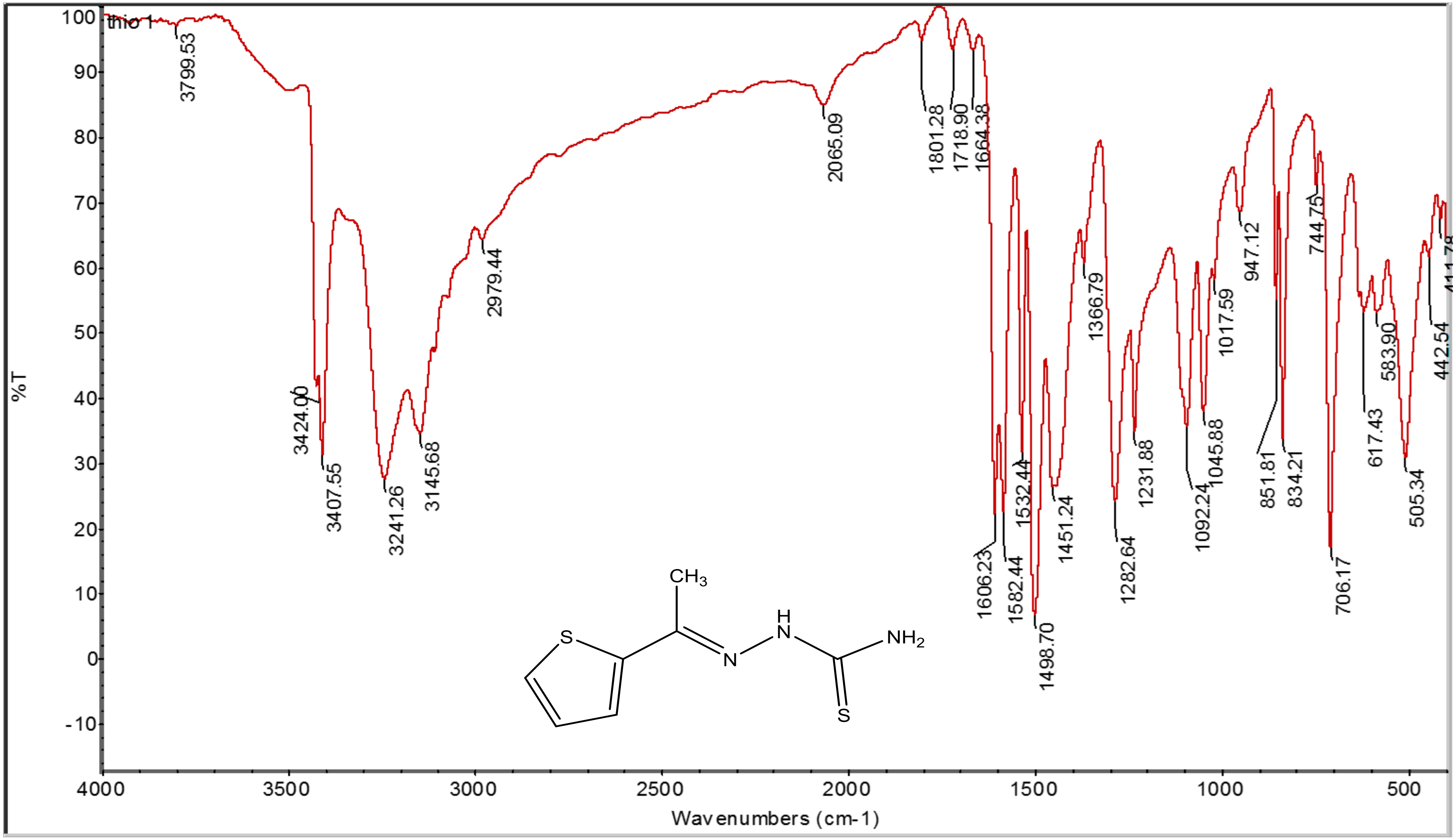

^{1}H NMR of compound (7a)

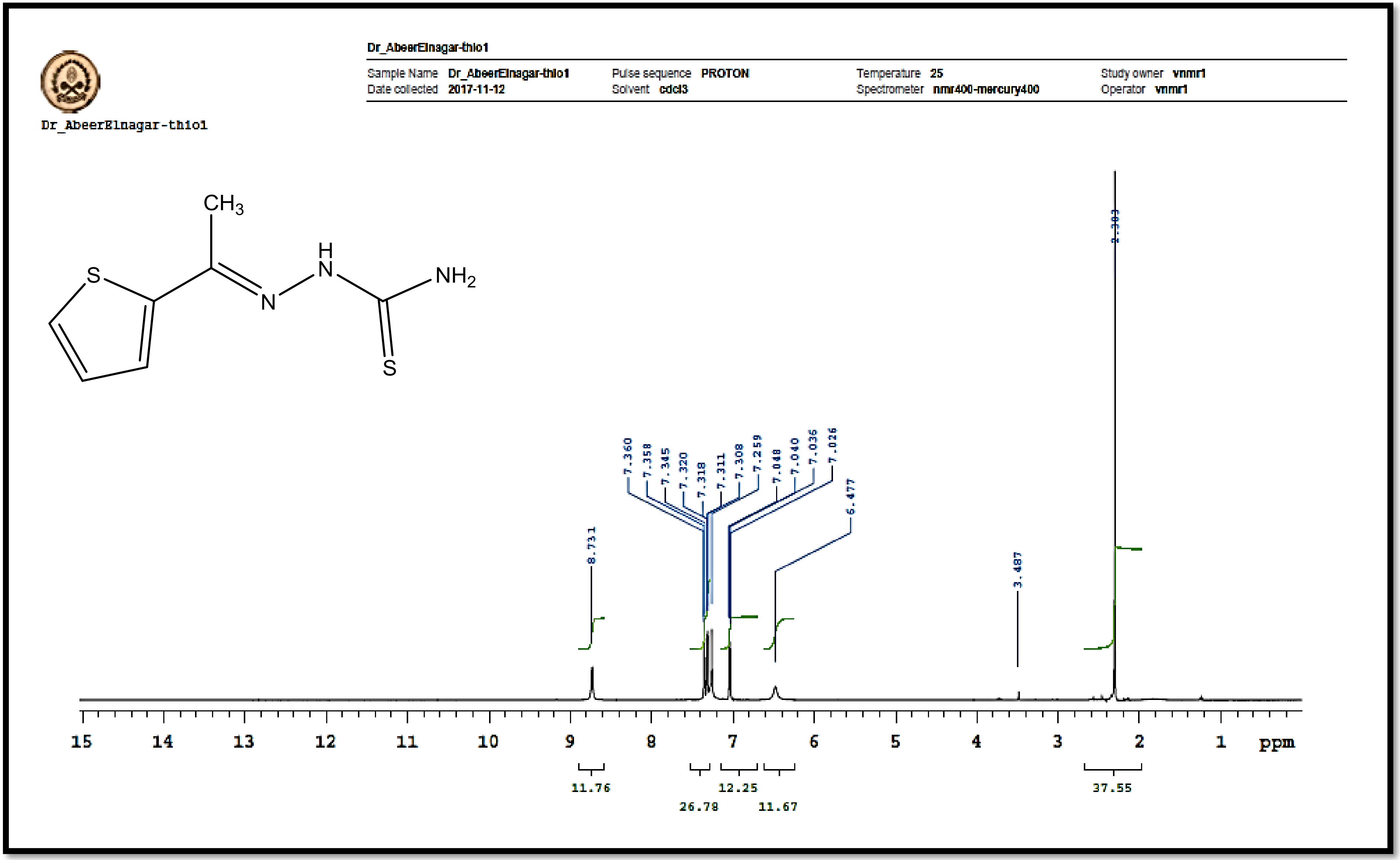

D$_2$O H NMR of compound (7a)

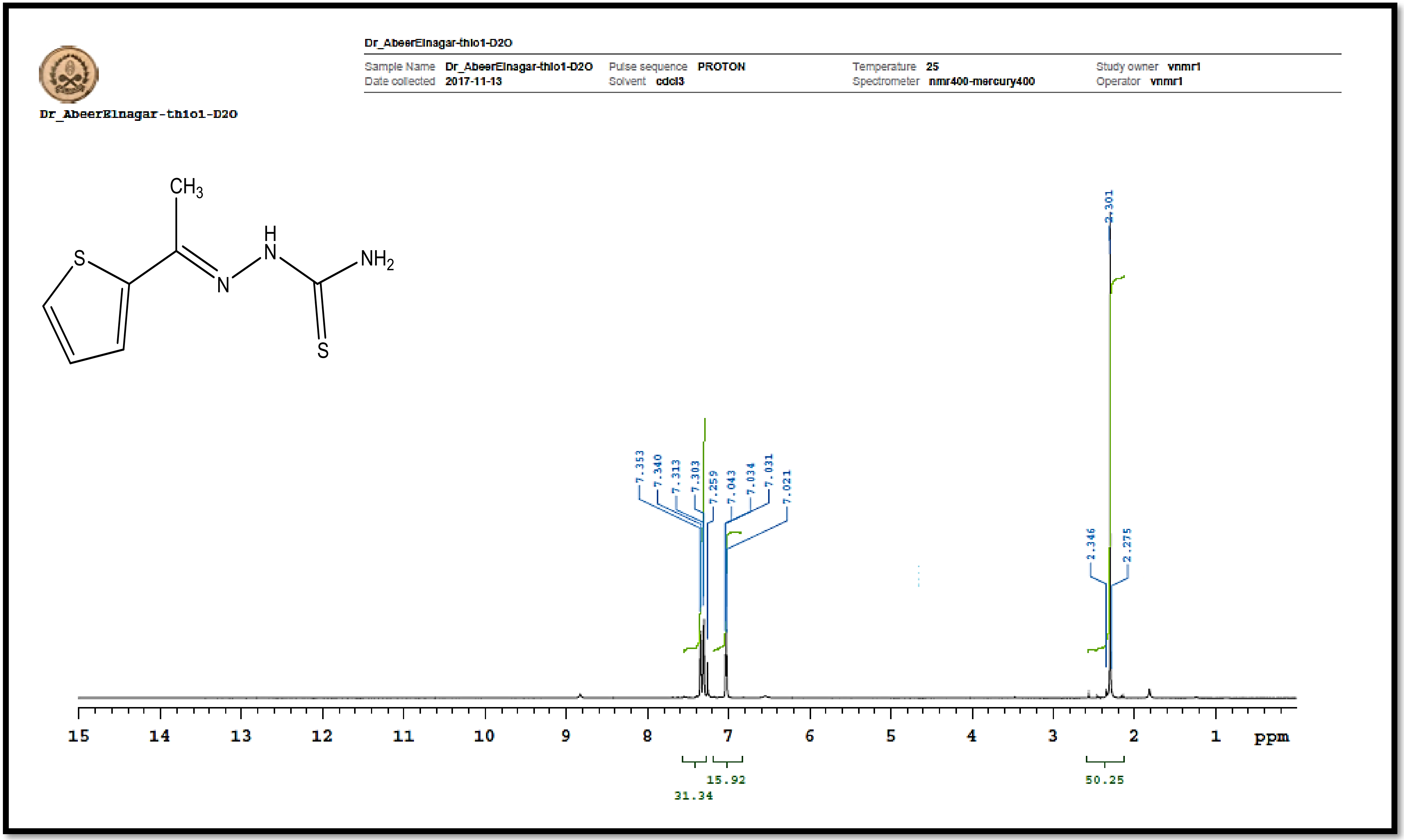

IR of compound (7b)

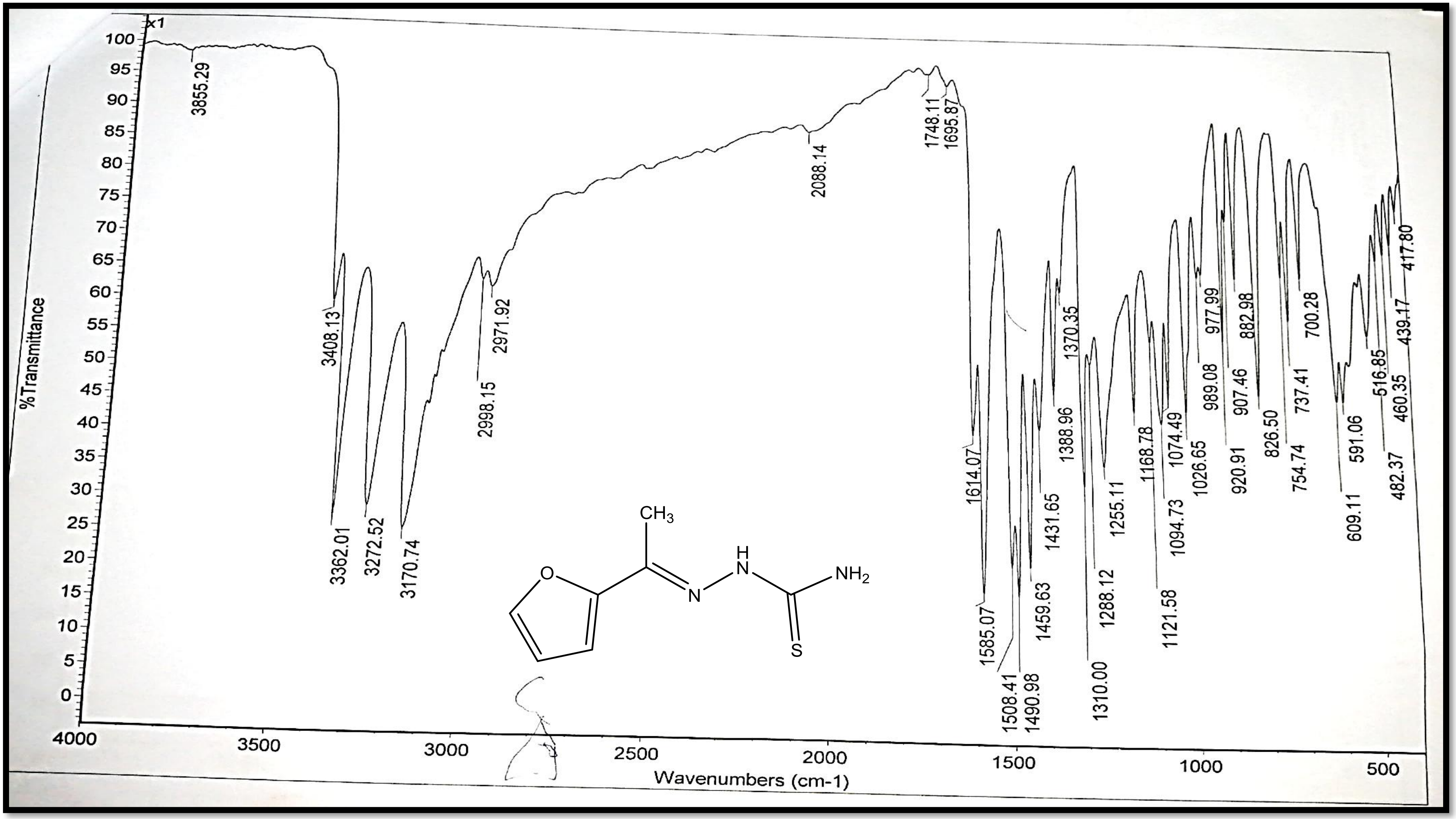

^{1}H NMR of compound (7b)

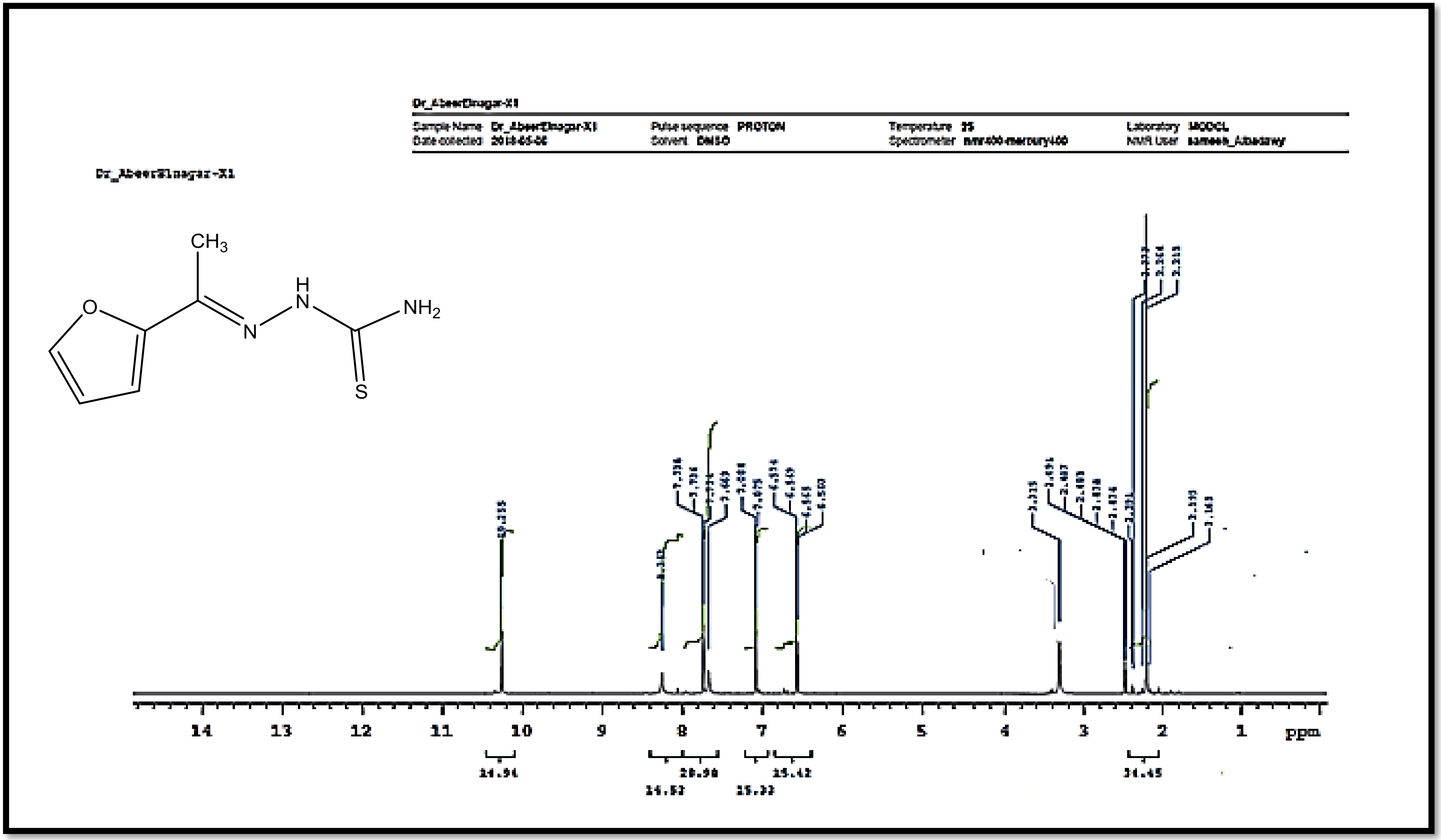

D_2O ^1H NMR of compound (7b)

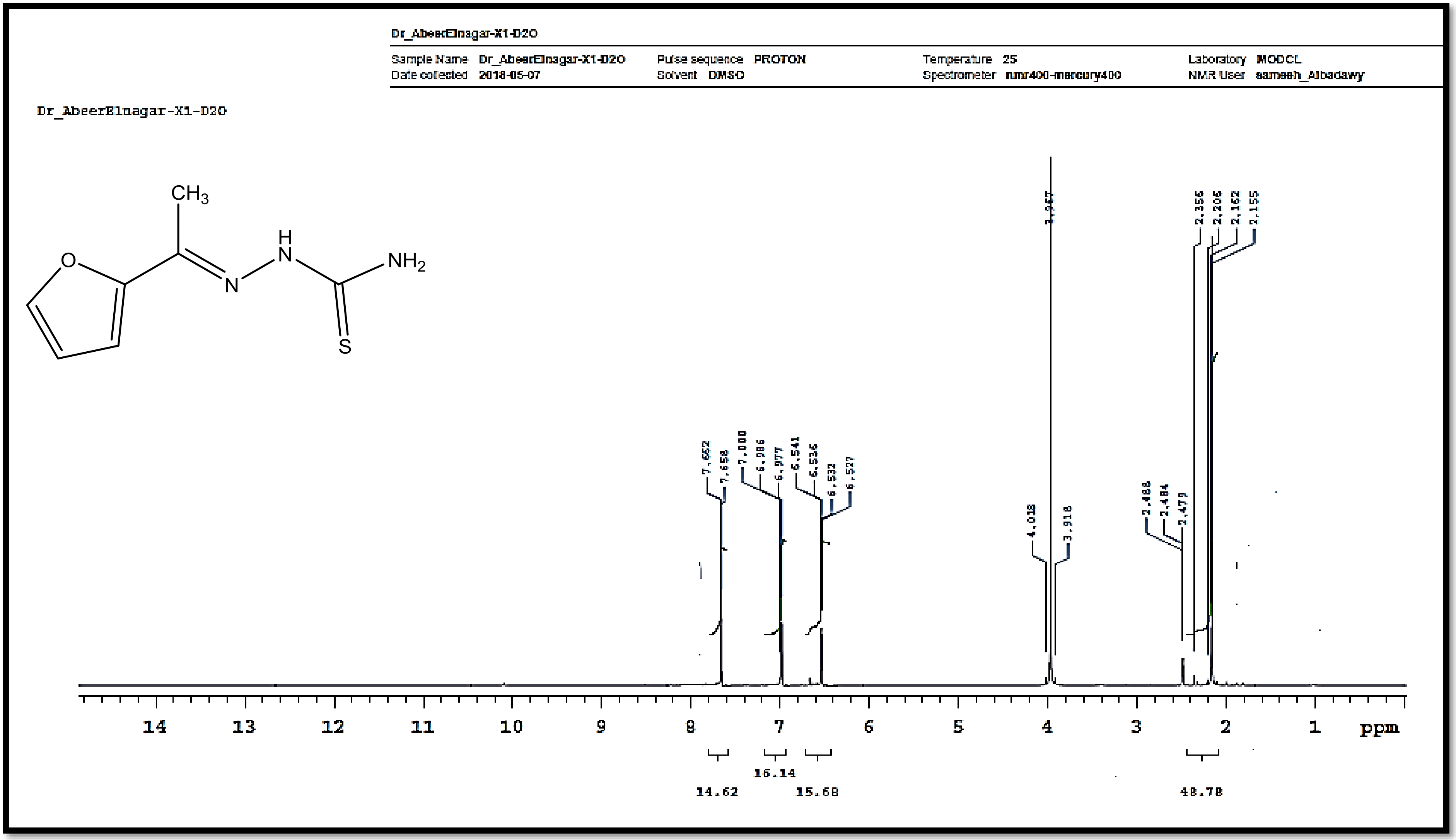

IR of compound (4a).

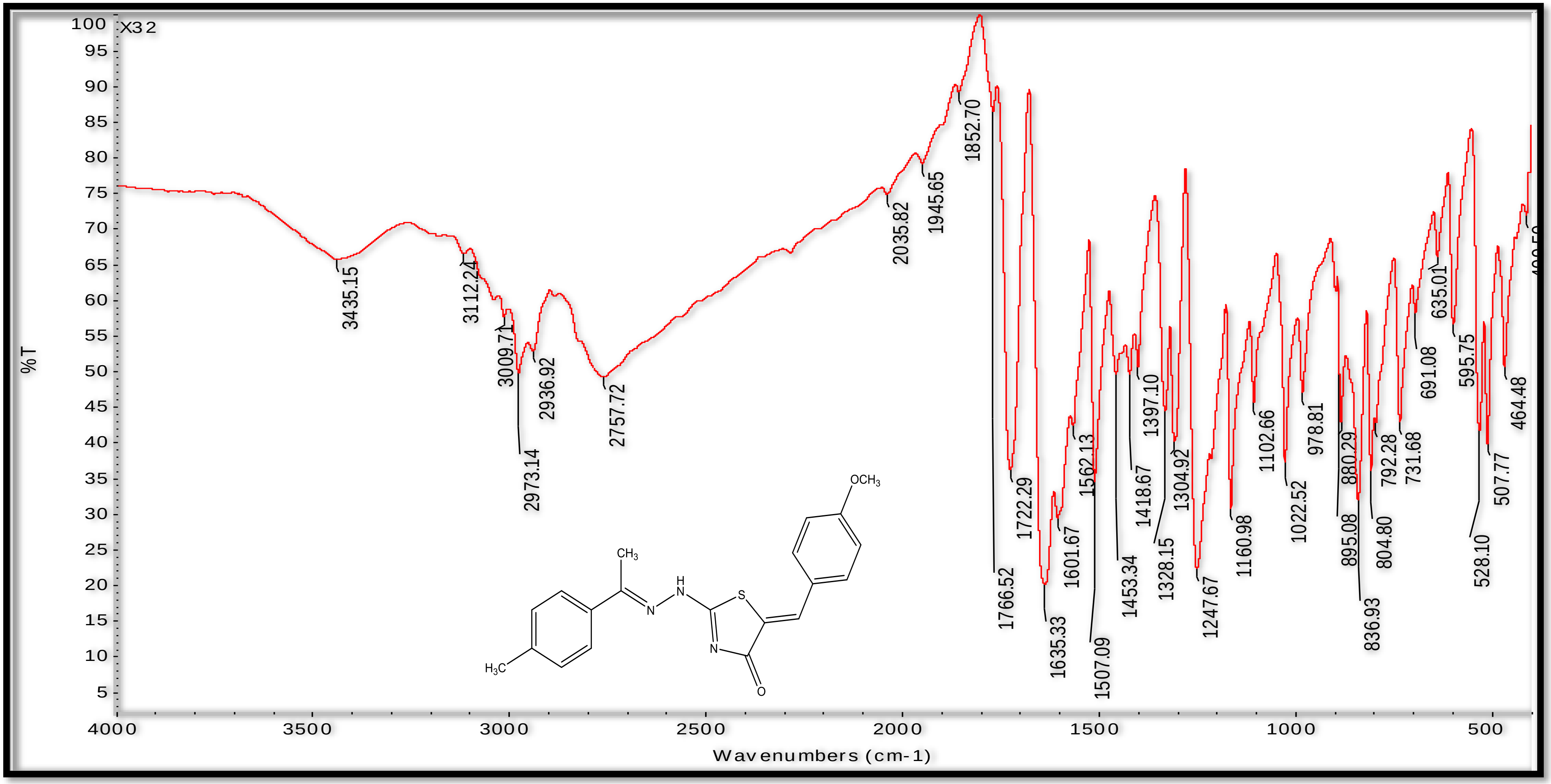

^{1}H NMR of compound (4a)

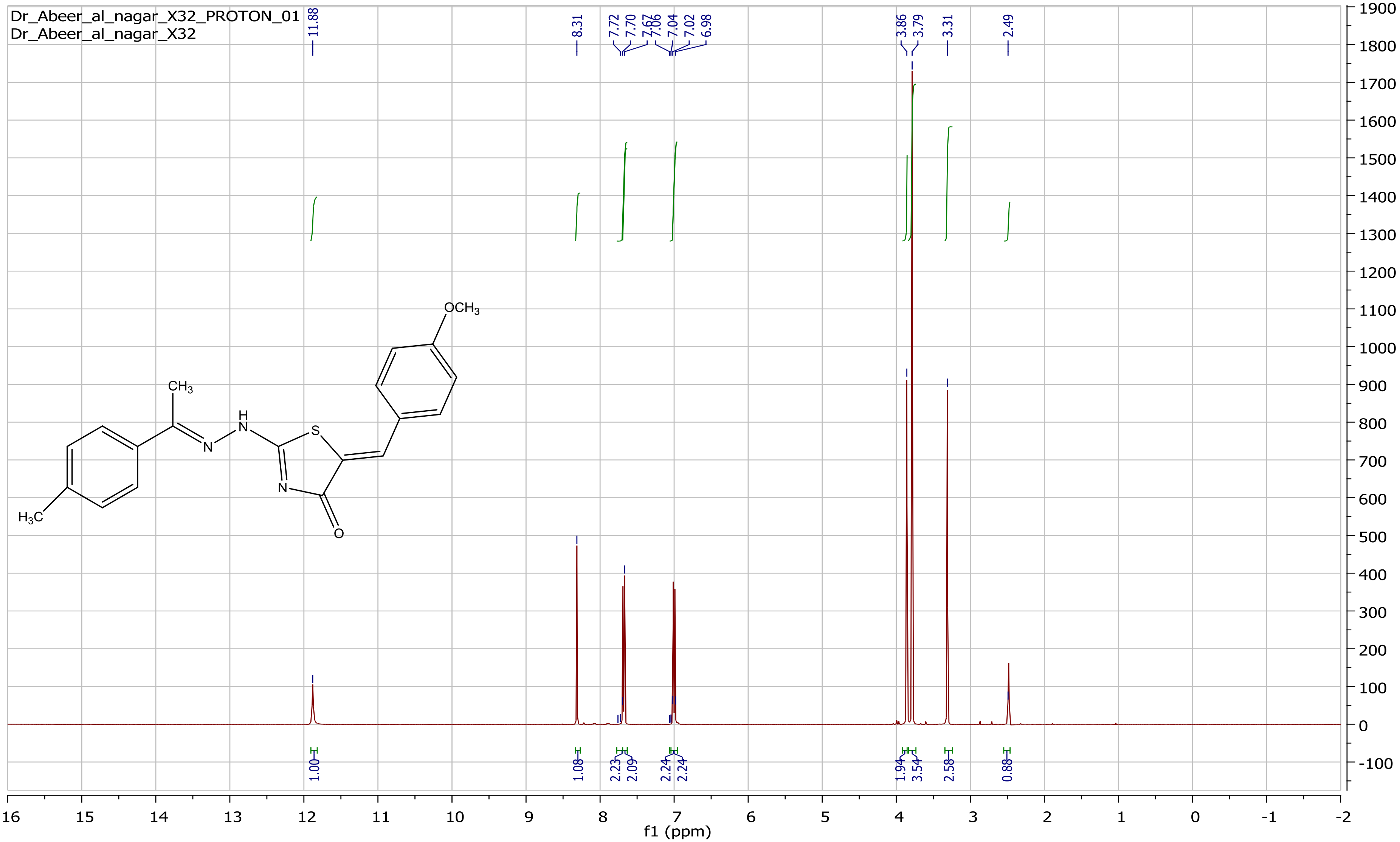

D$_2$O ^{1}H NMR of compound (4a)

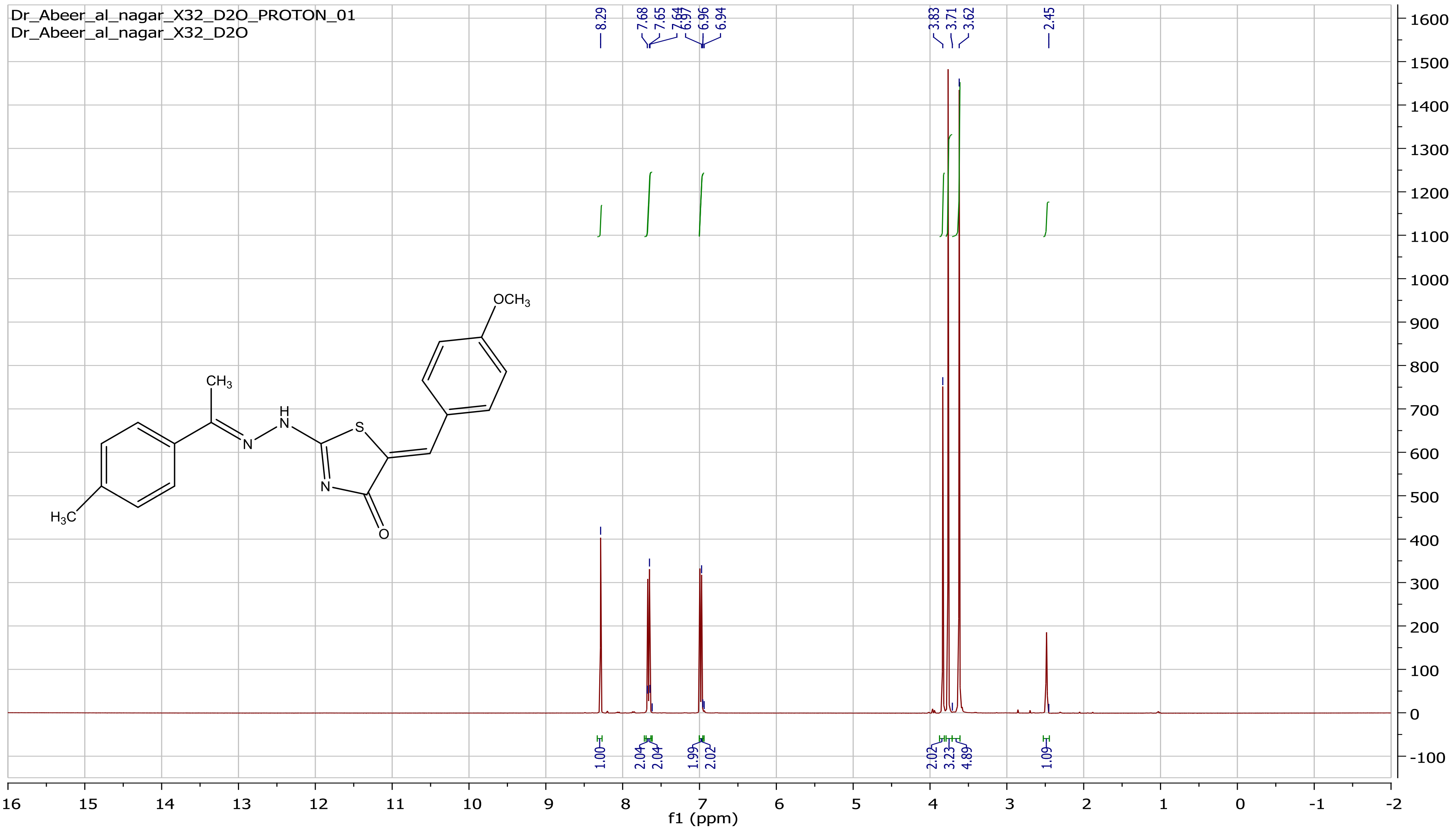

IR of compound (4b)

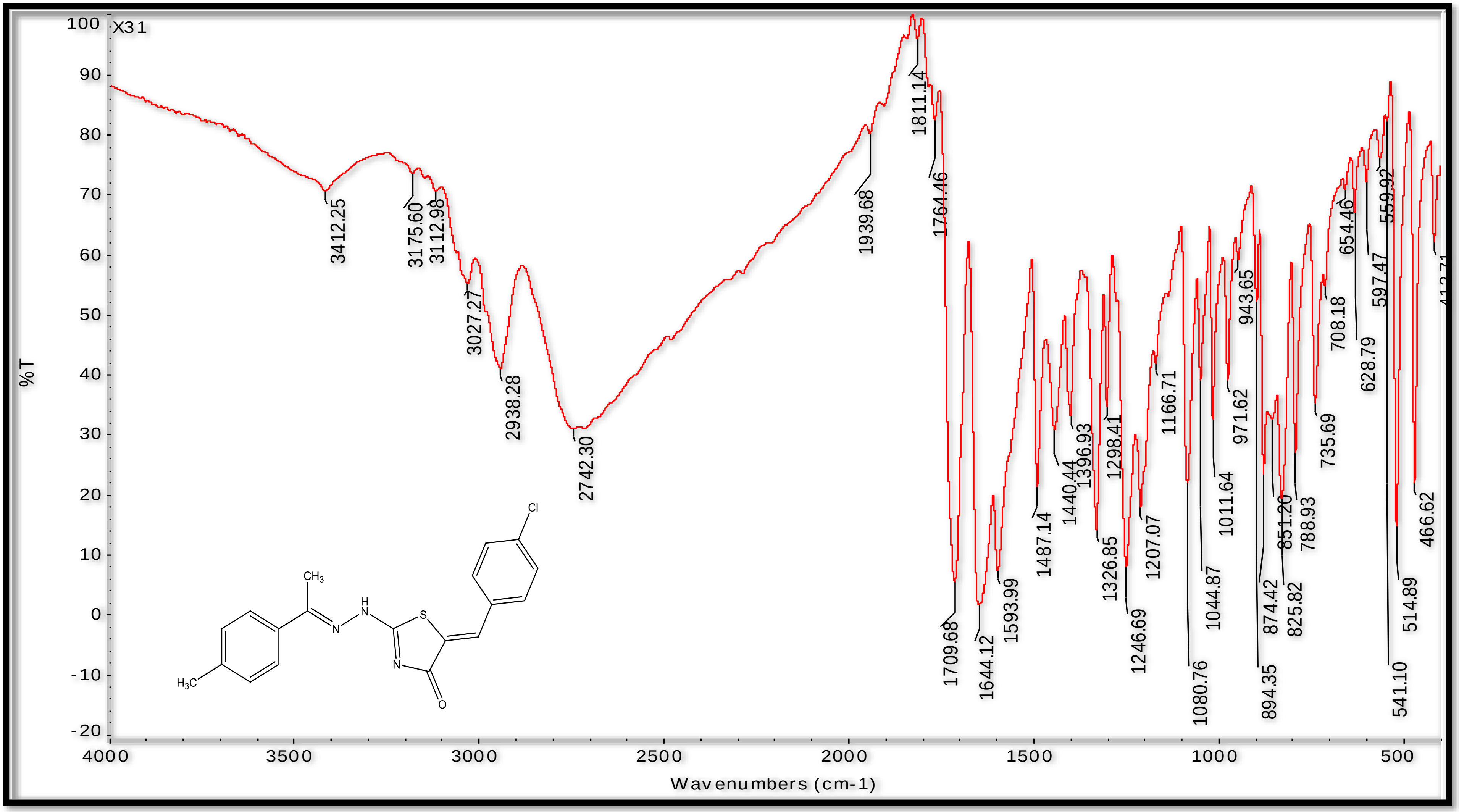

^{1}H NMR of compound (4b)

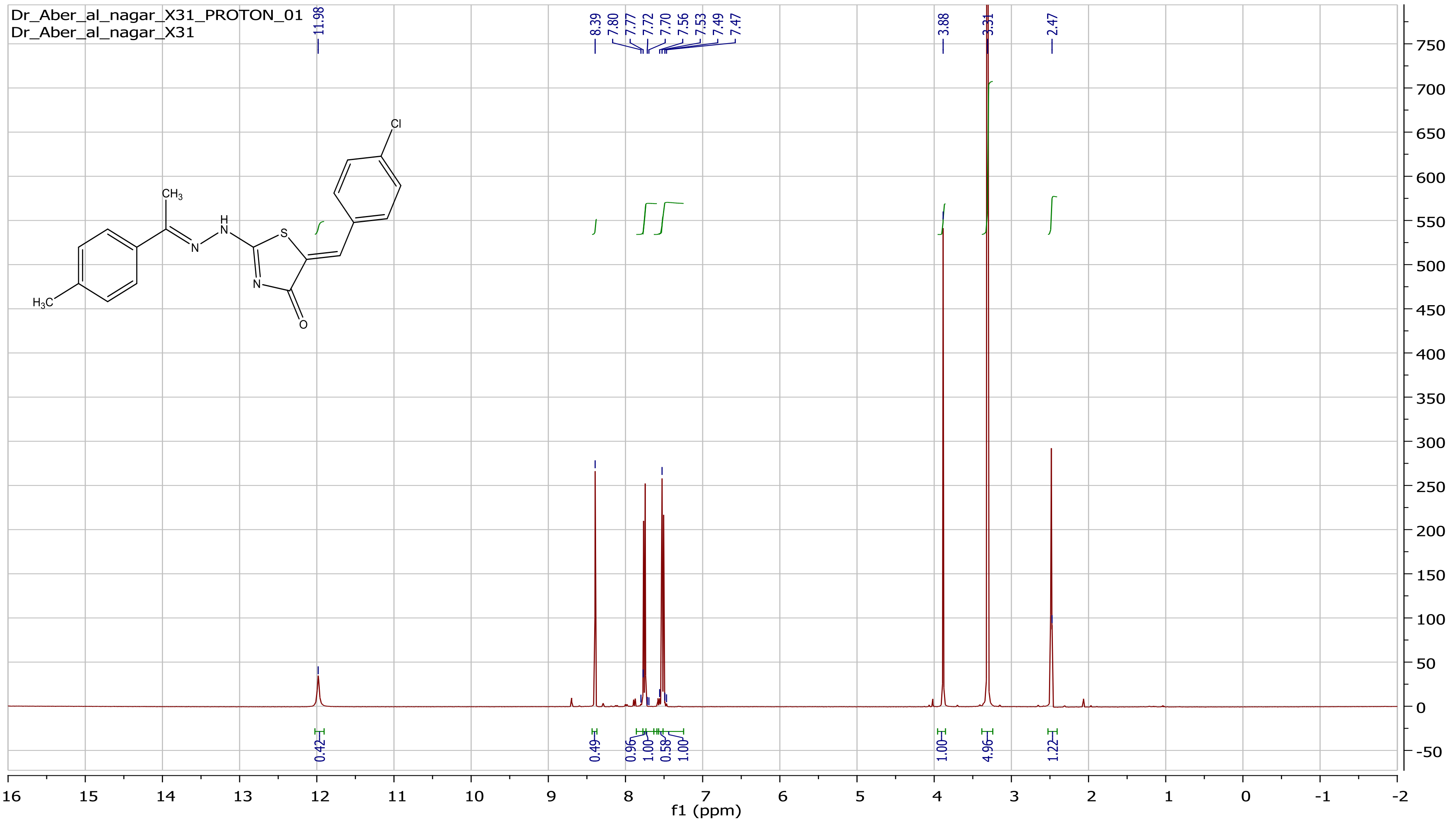

D2O ^{1}H NMR of compound (4b)

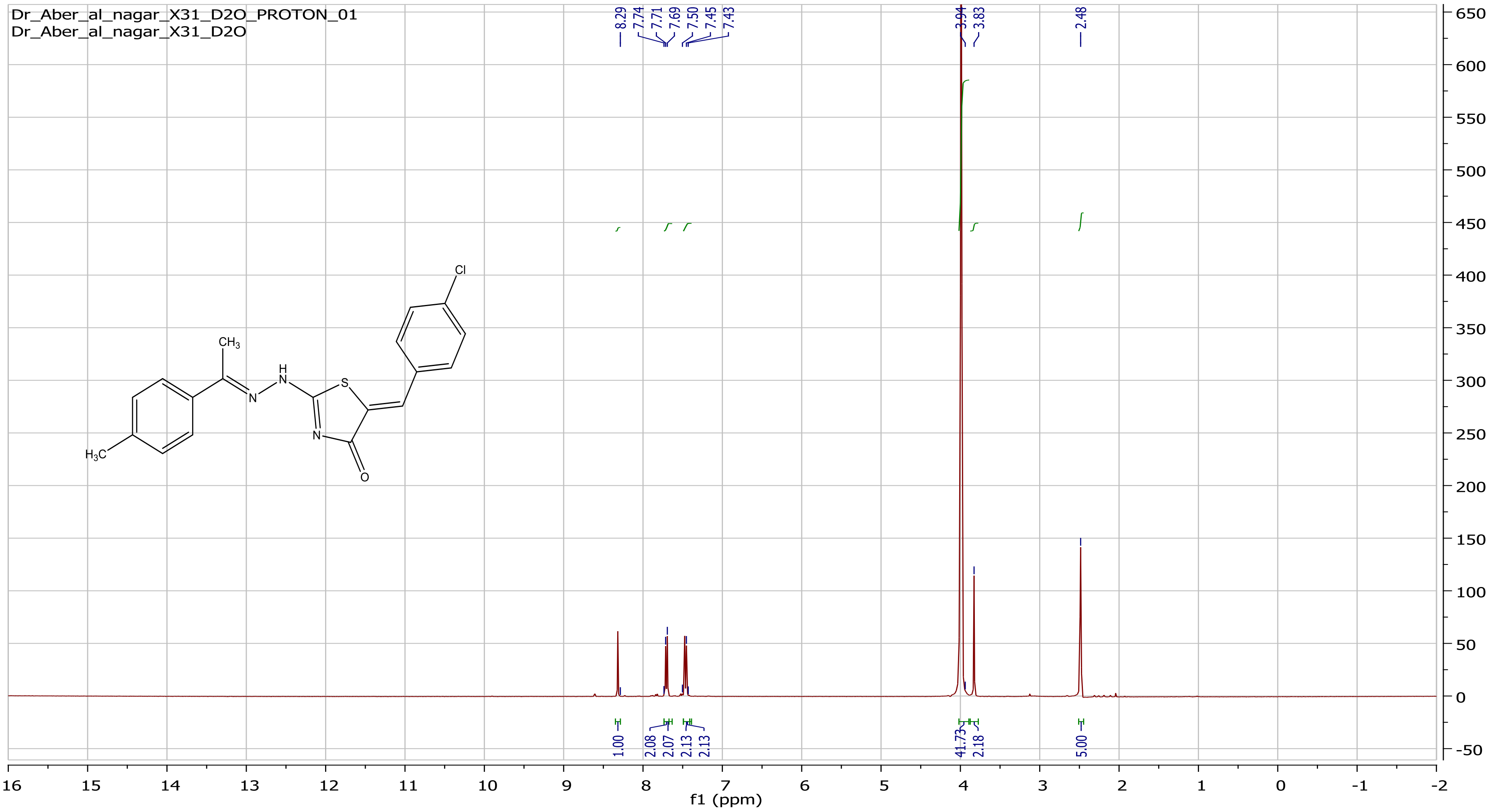

IR of compound (4c)

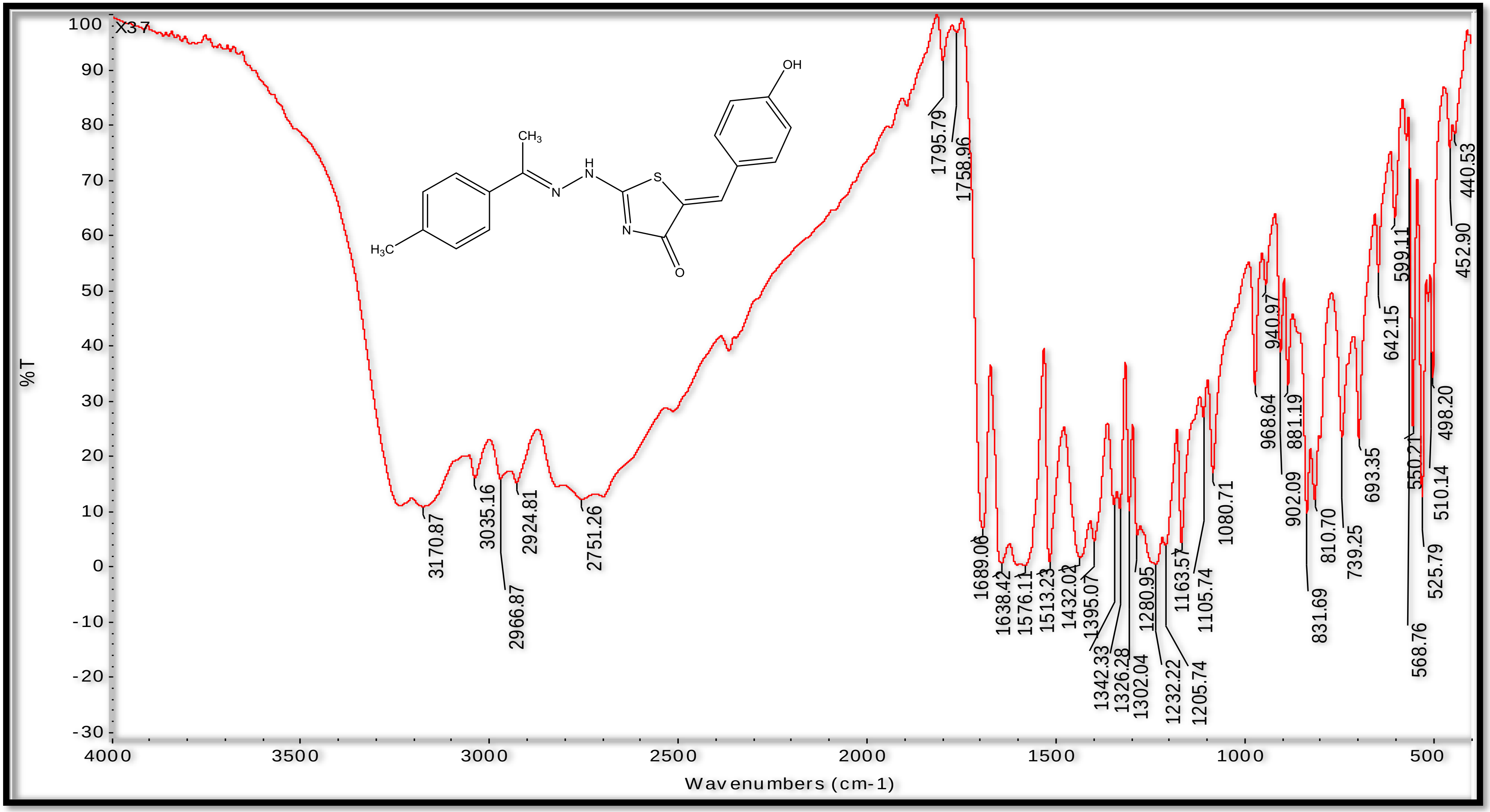

^{1}H NMR of compound (4c).

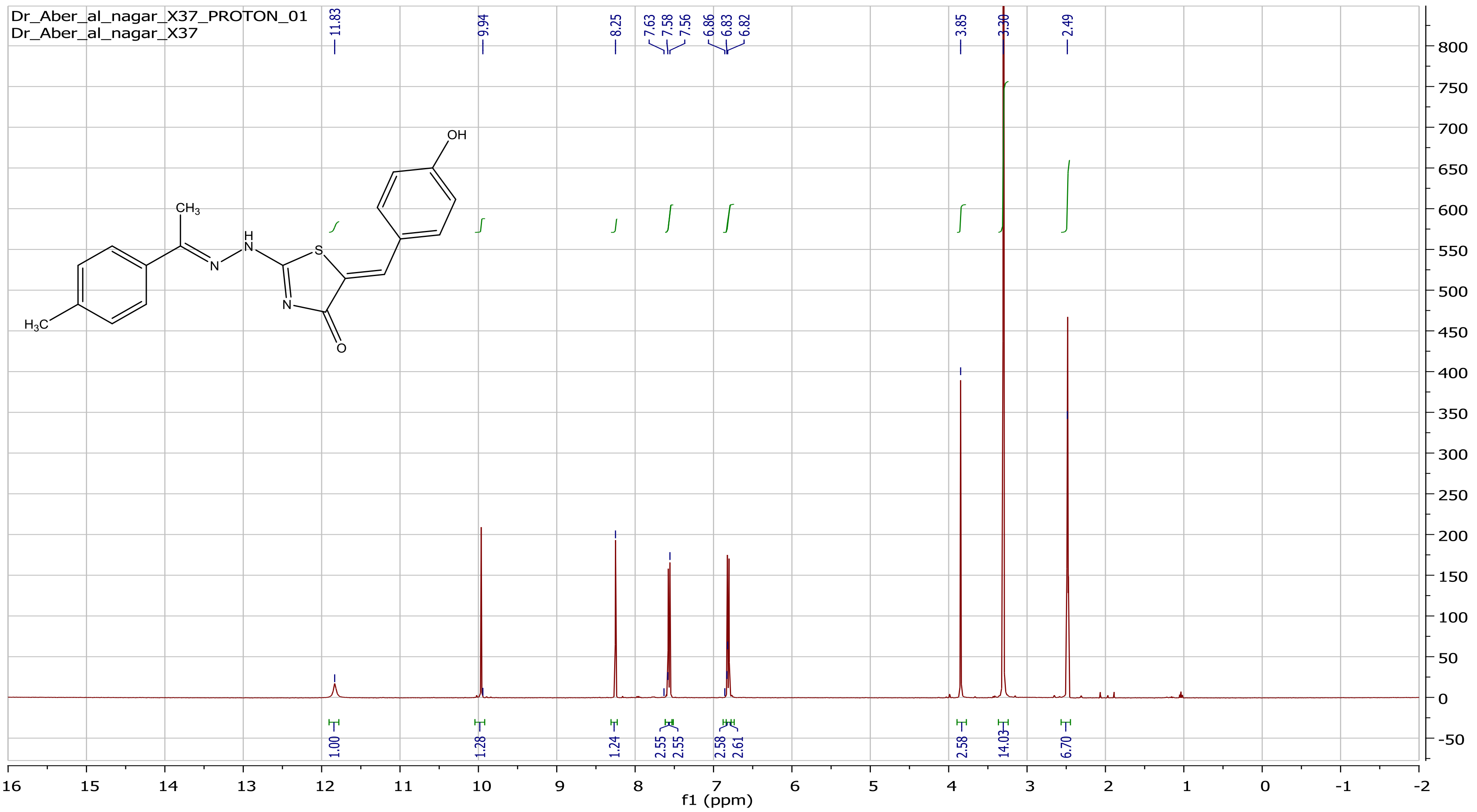

IR of compound (4d)

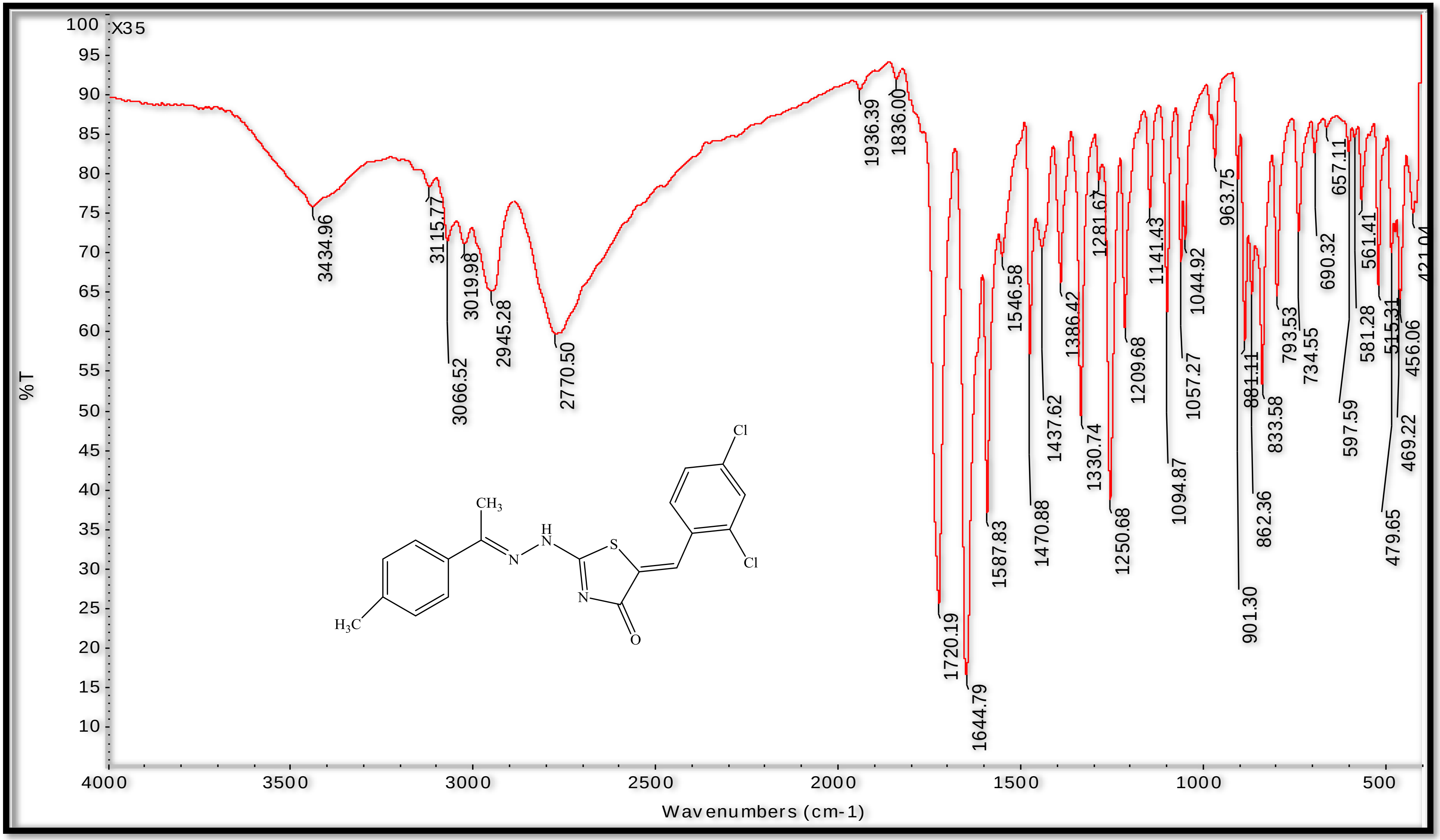

^{1}H NMR of compound (4d)

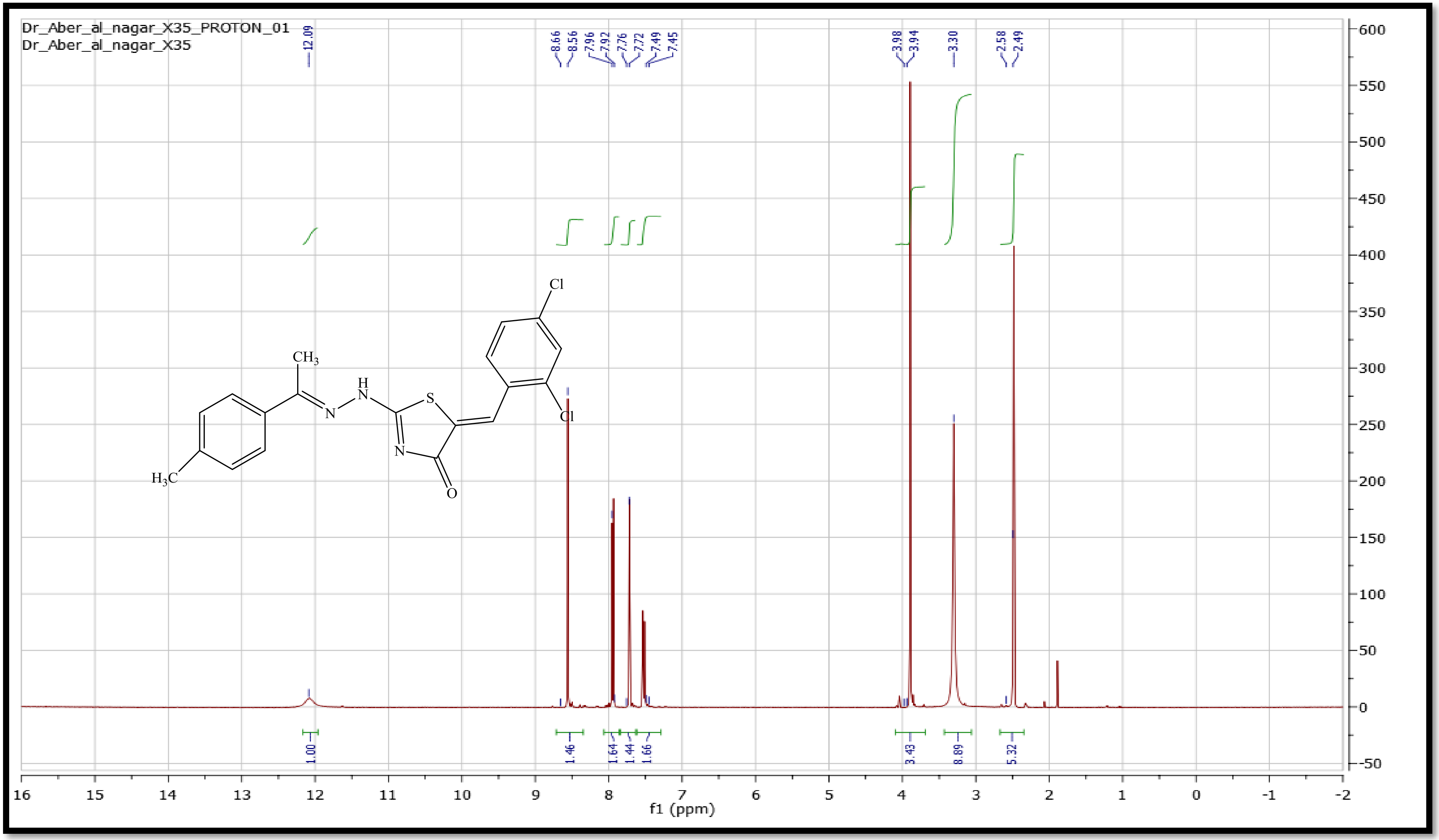

^{1}H NMR of compound (4d)

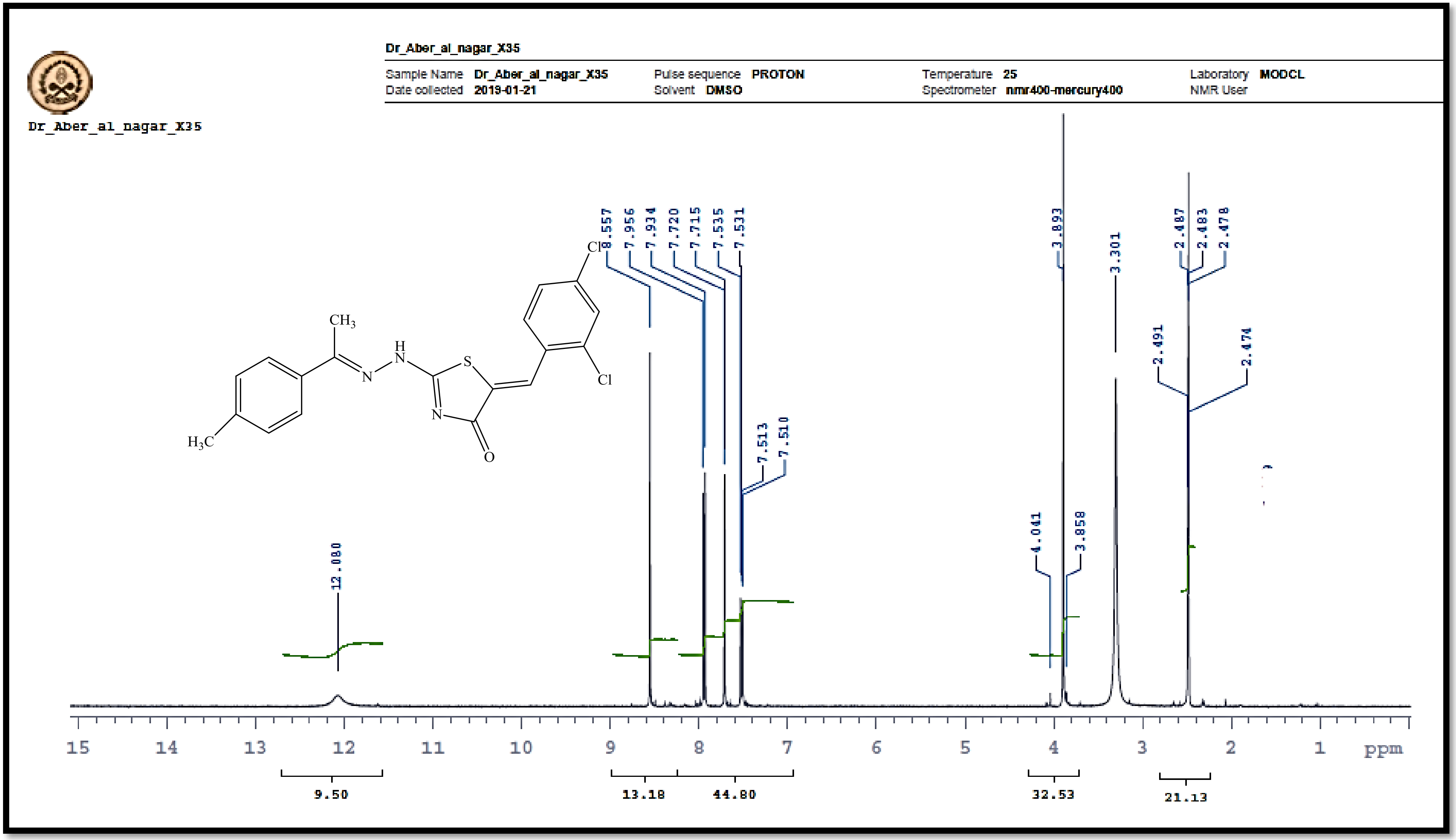

IR of compound (4e)

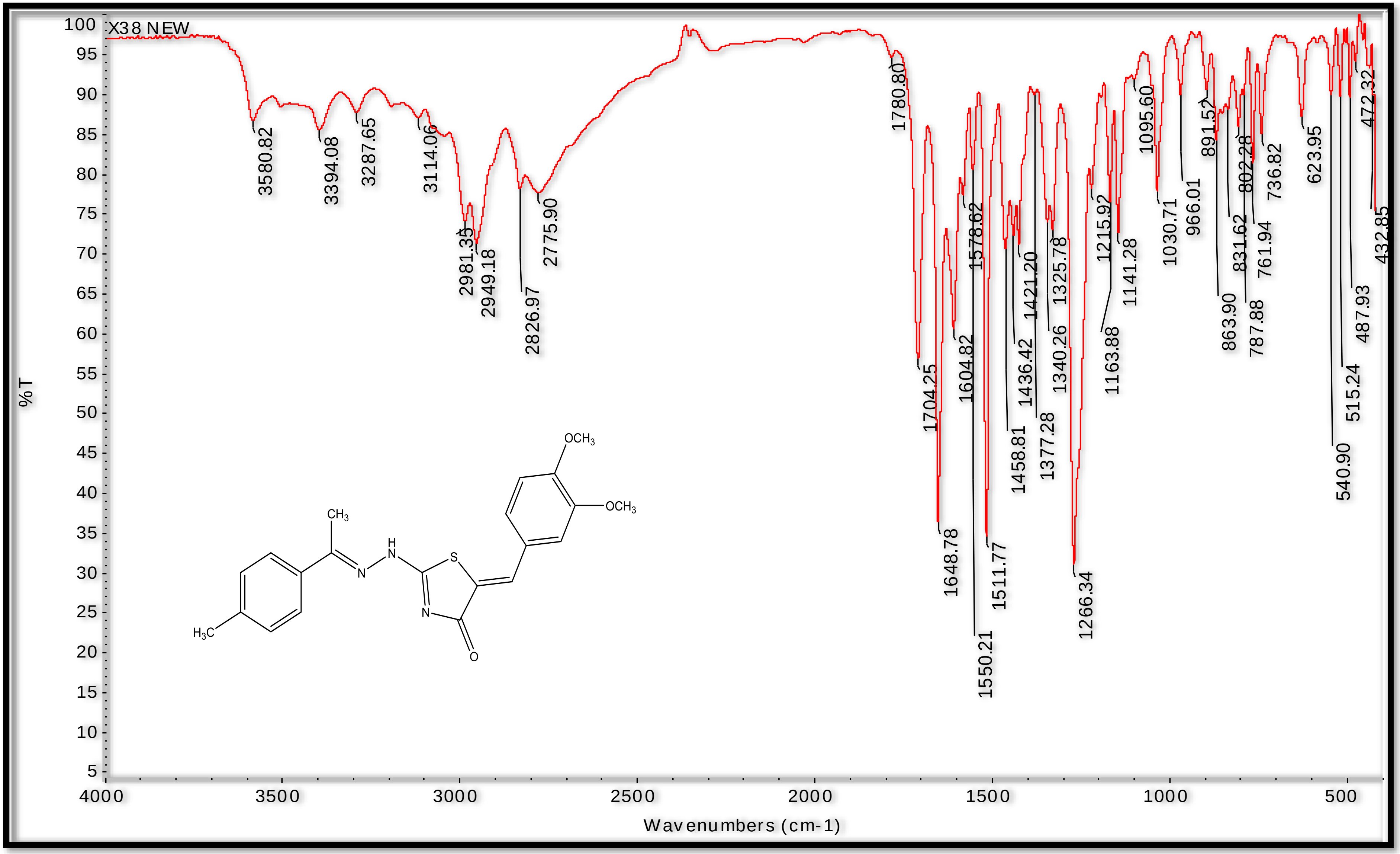

^{1}H NMR of compound (4e)

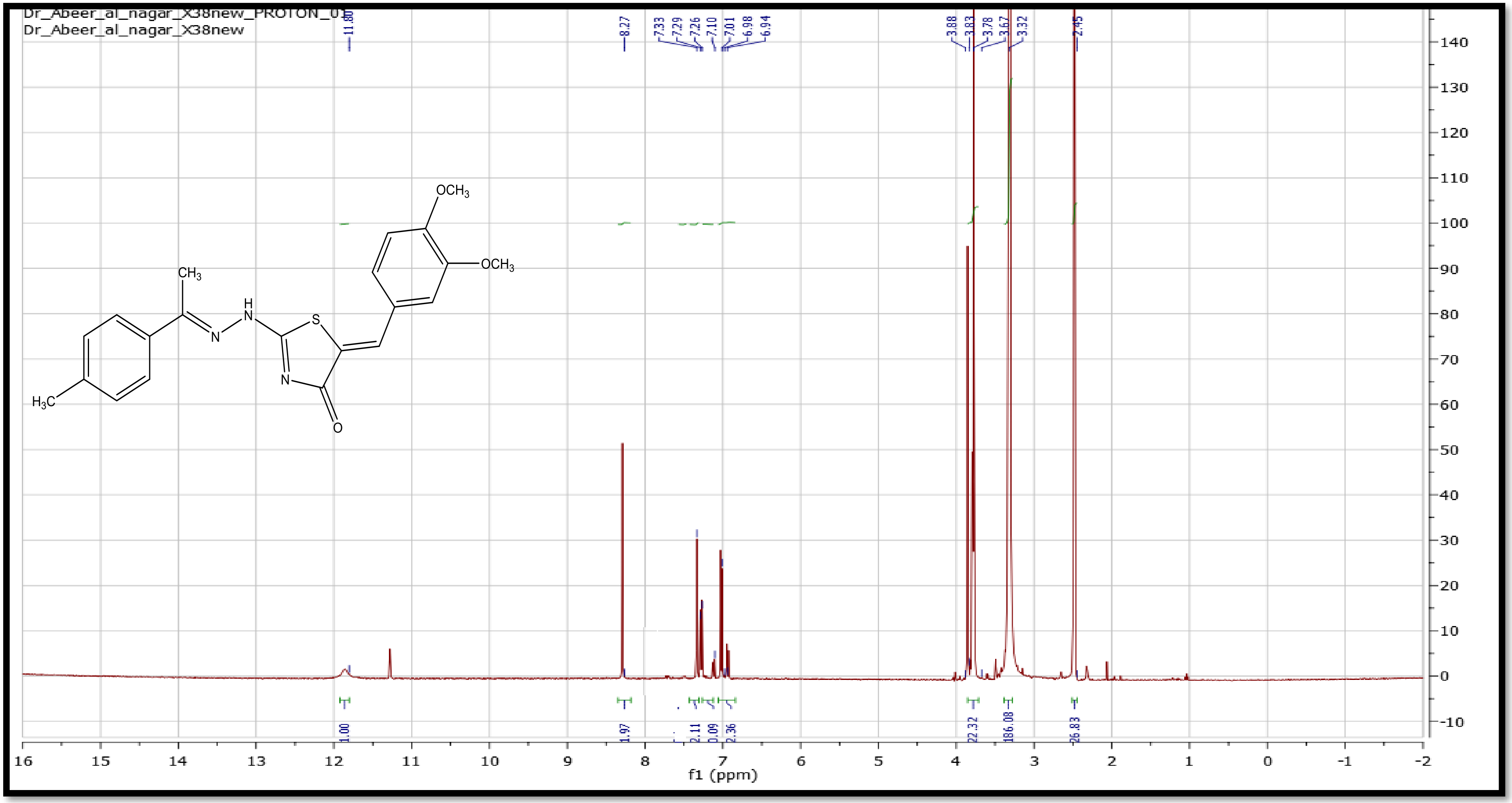

^{1}H NMR of compound (4e)

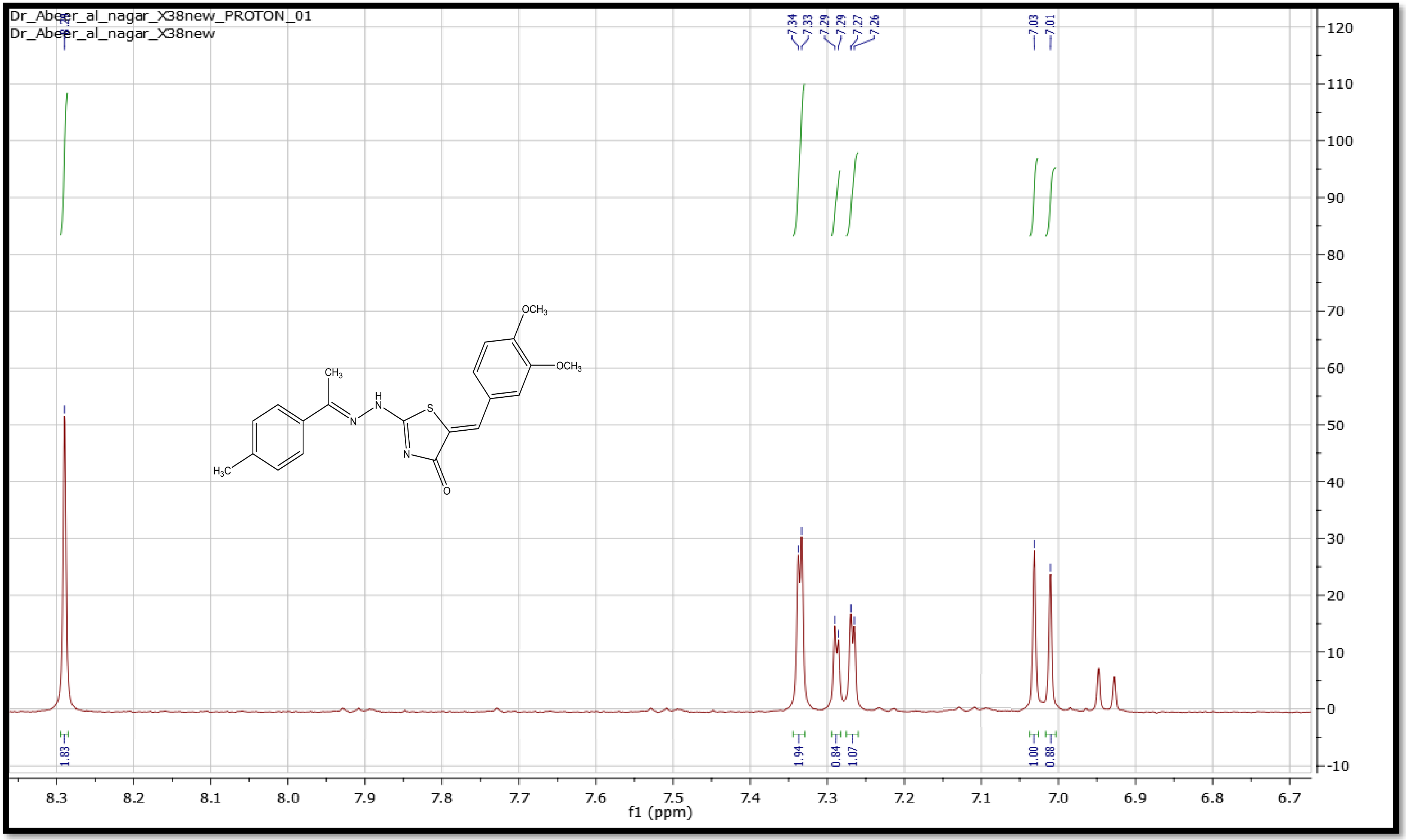

IR of compound (4f)

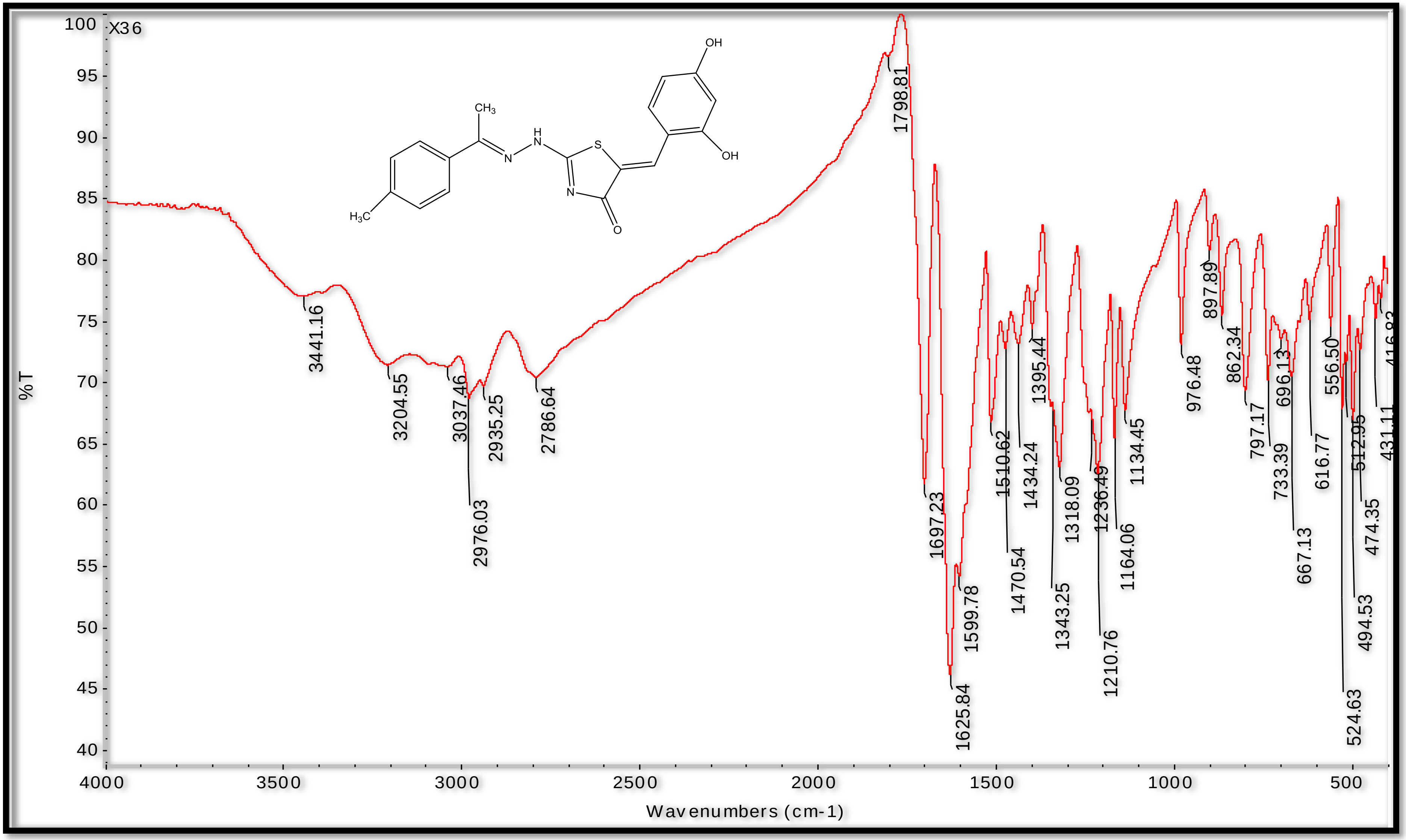

^{1}H NMR of compound (4f)

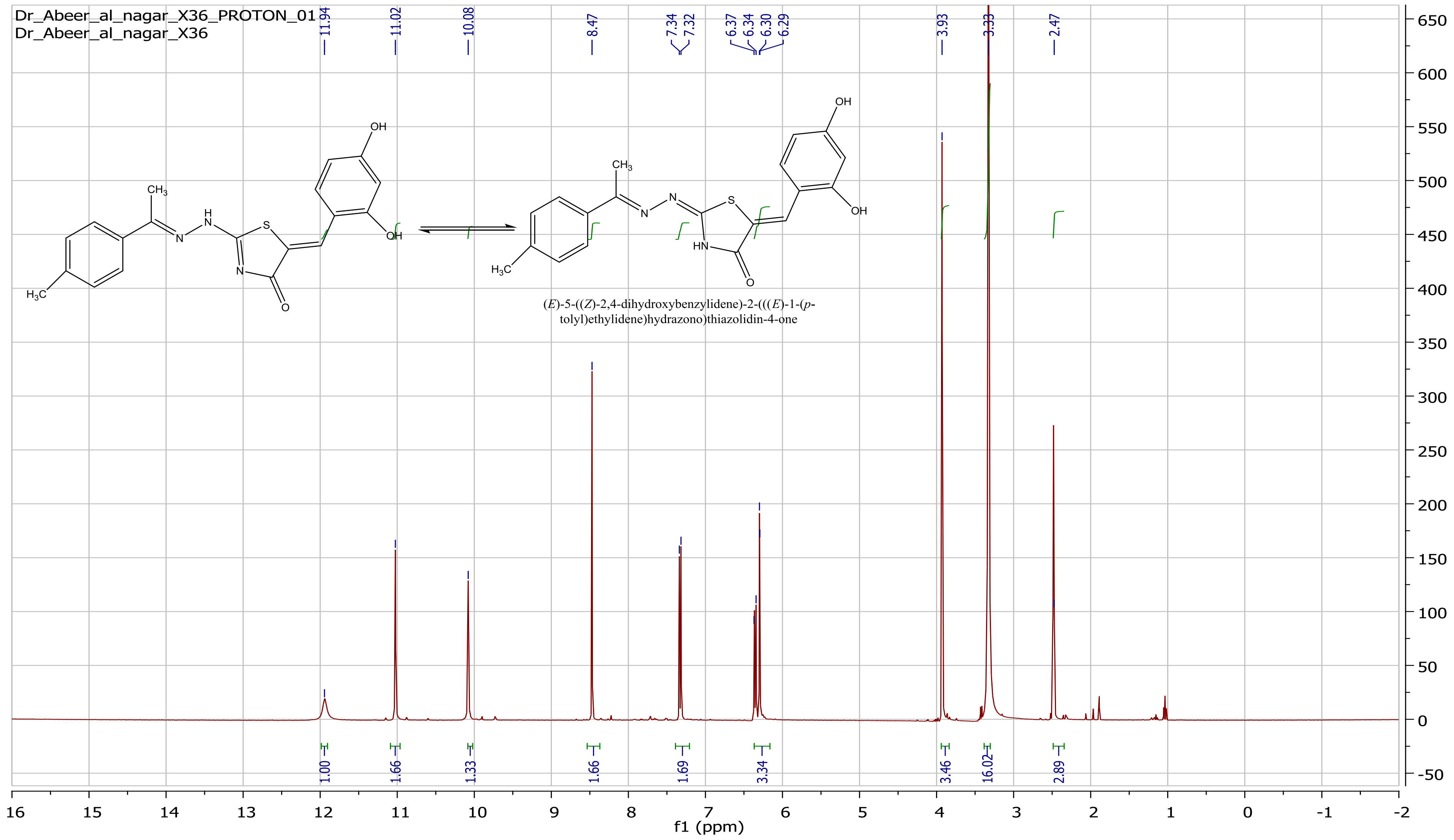

^{1}H NMR of compound (4f)

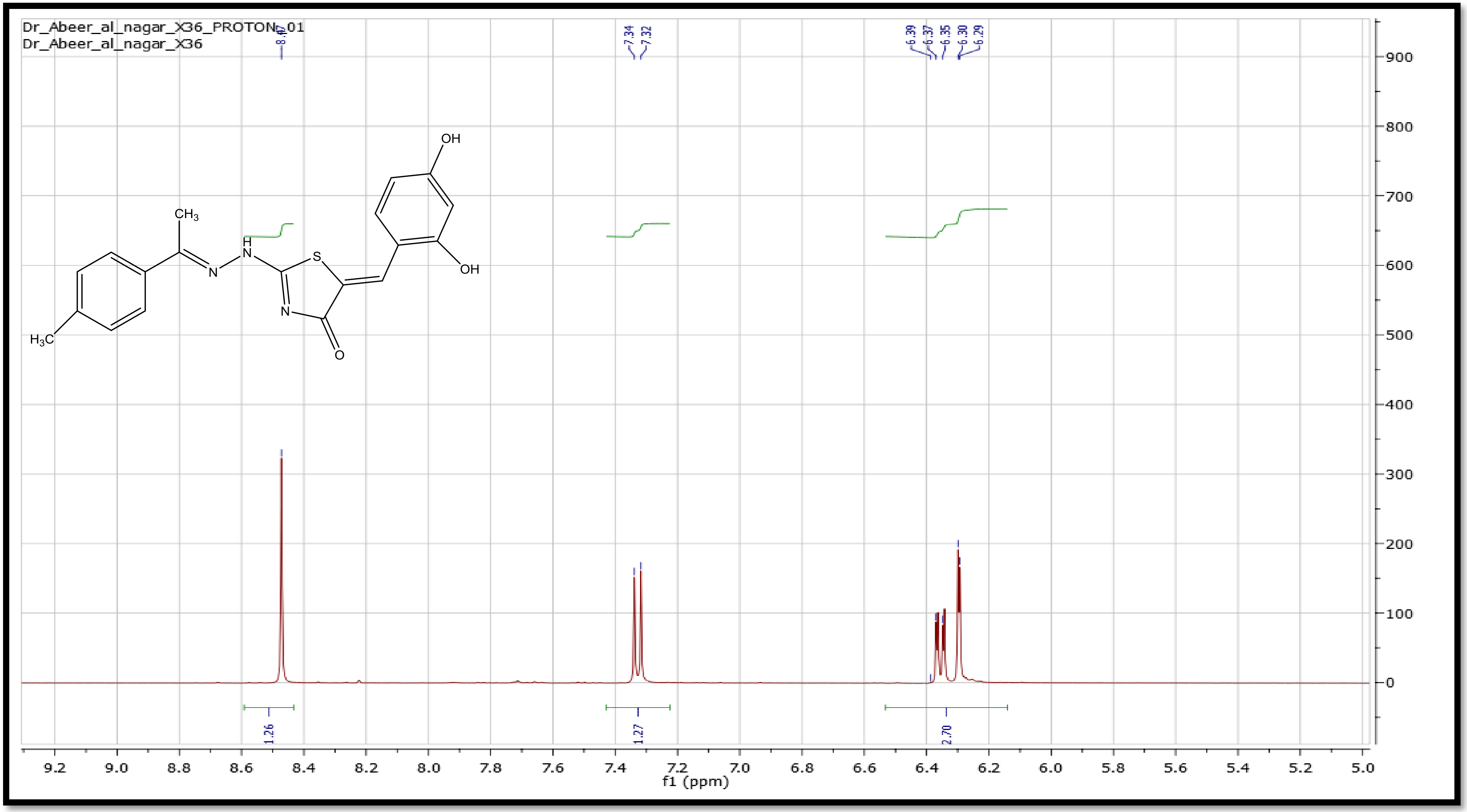

D$_2$O ^{1}H NMR of compound (4f)

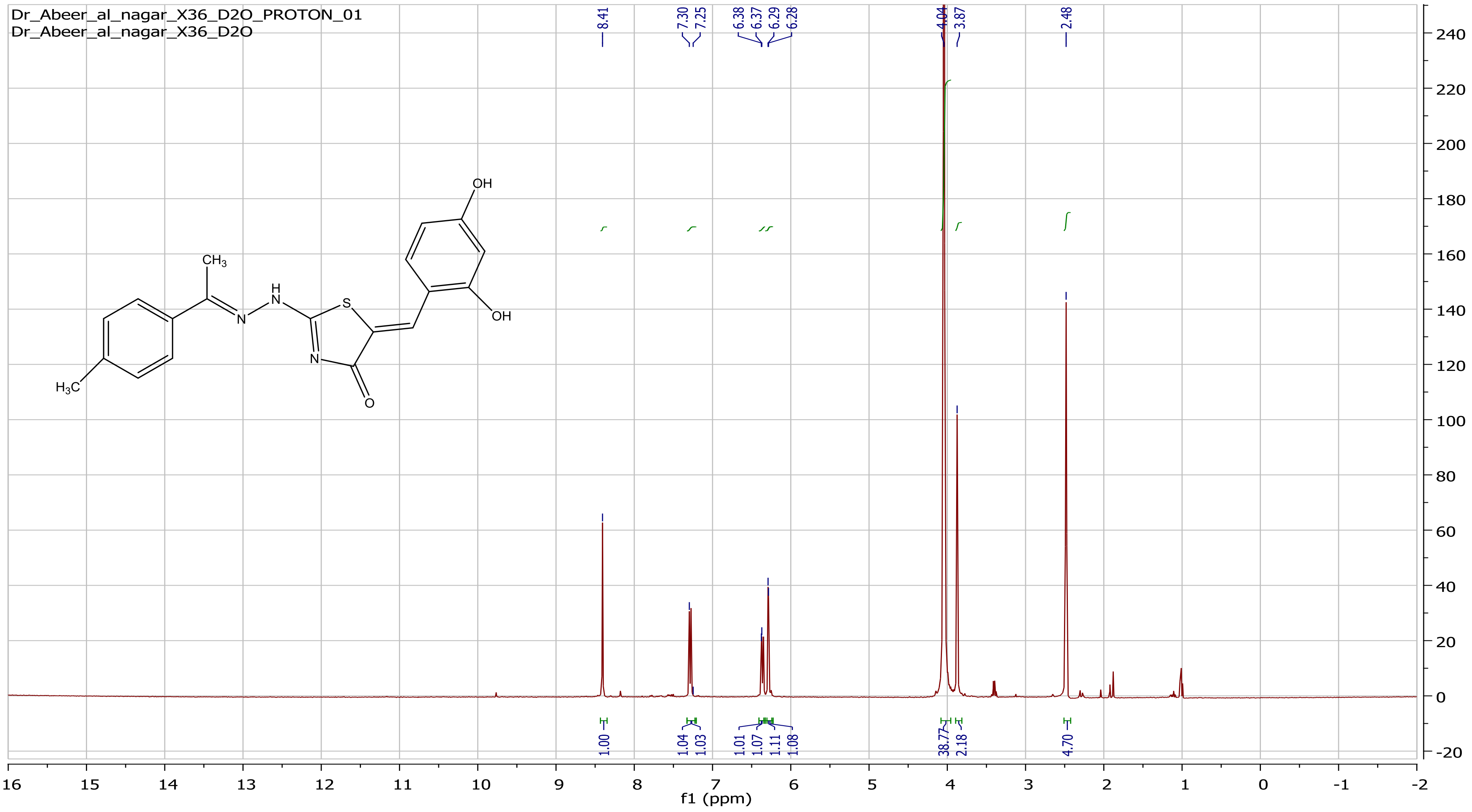

IR of compound (4g).

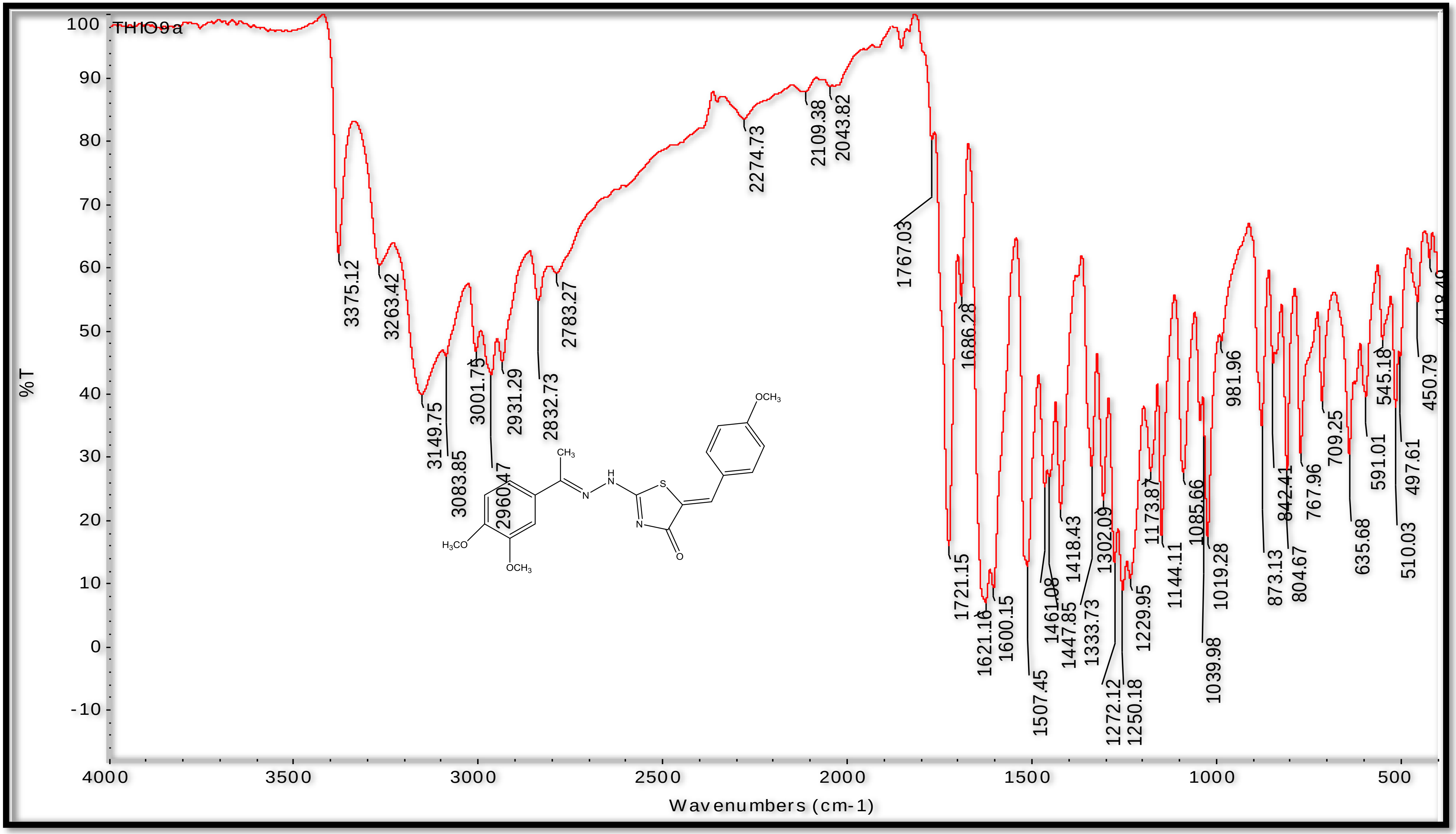

^{1}H NMR of compound (4g).

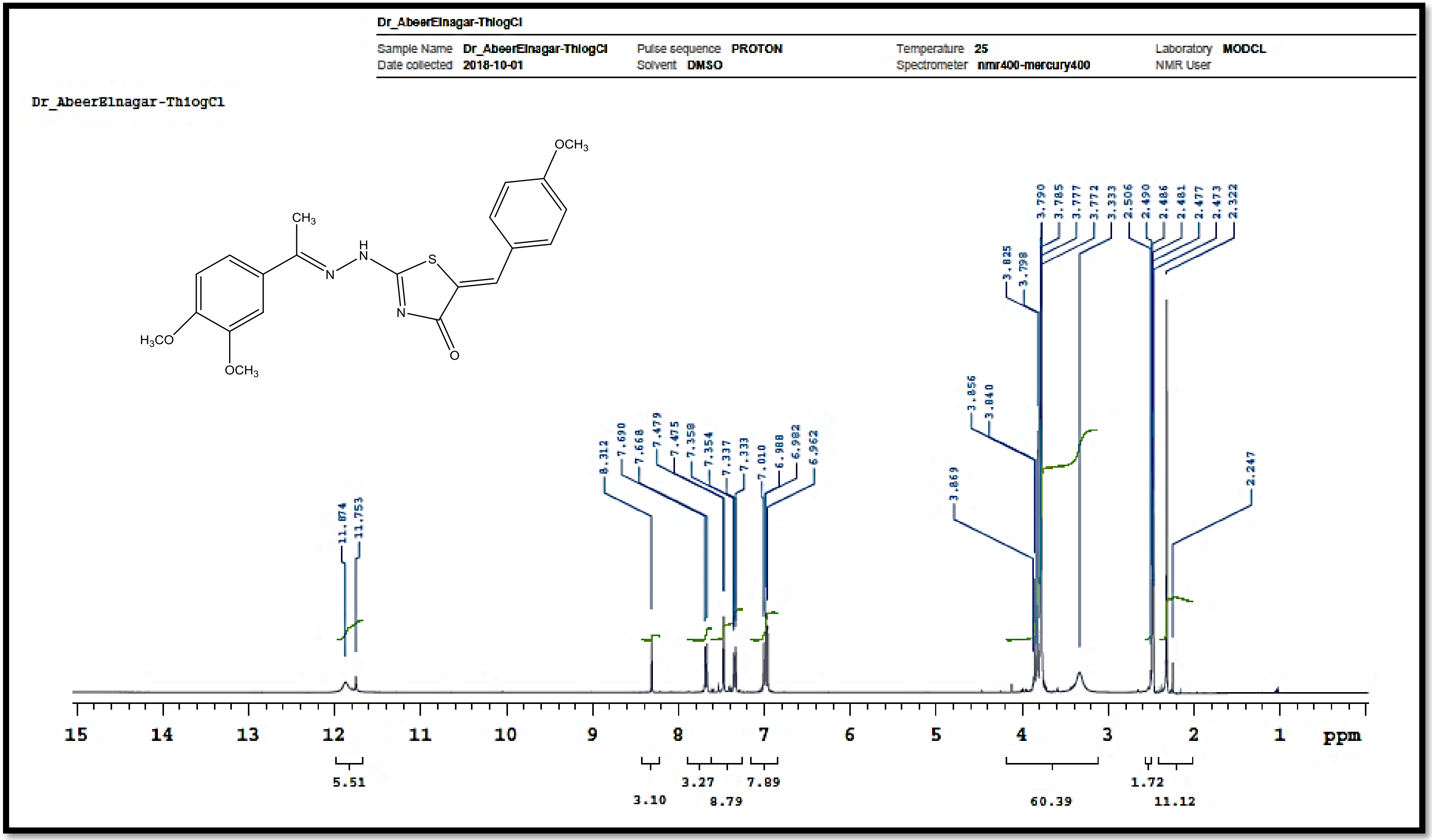

D$_2$O ^{1}H NMR of compound (4g)

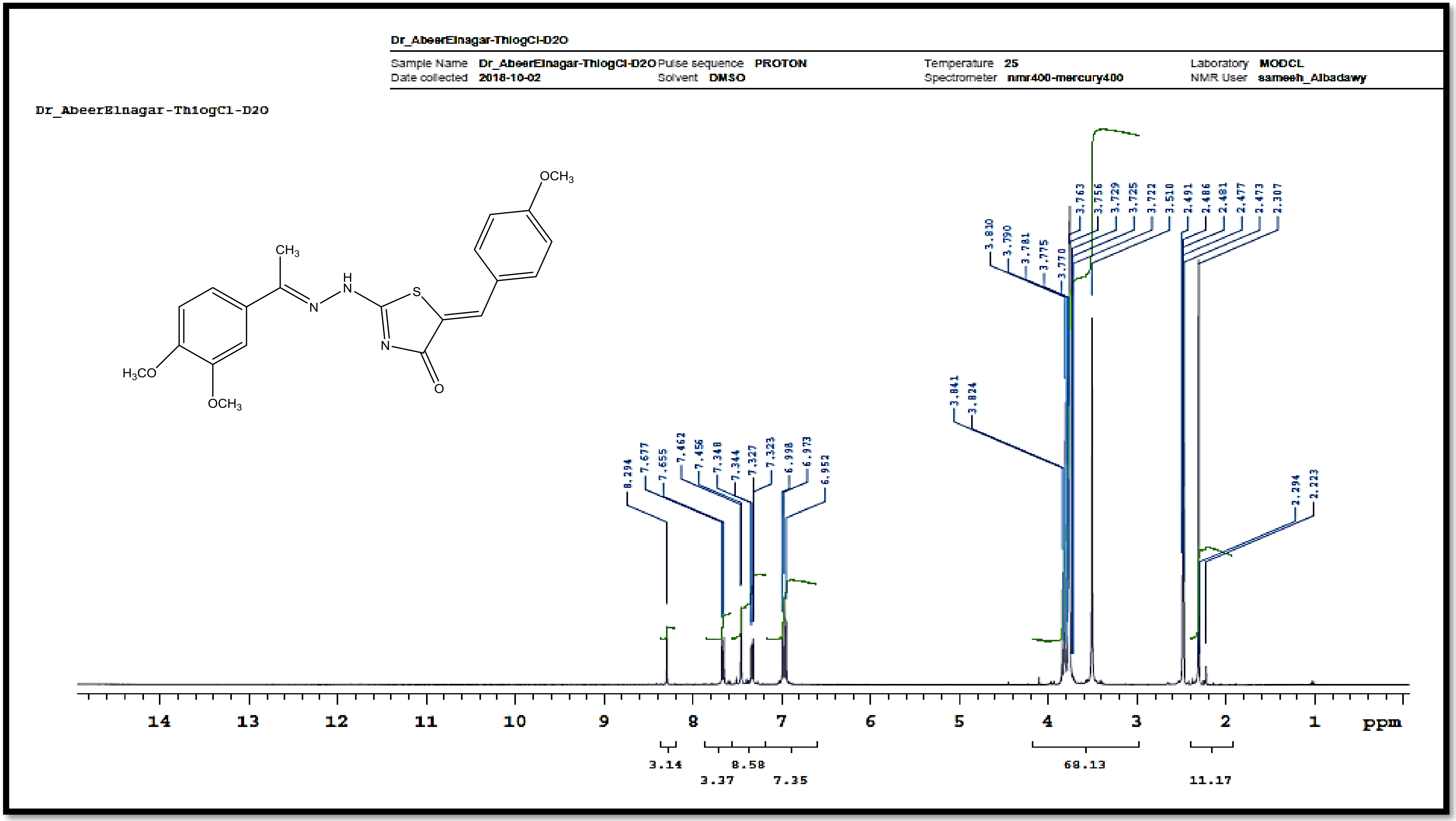

IR of compound (4h).

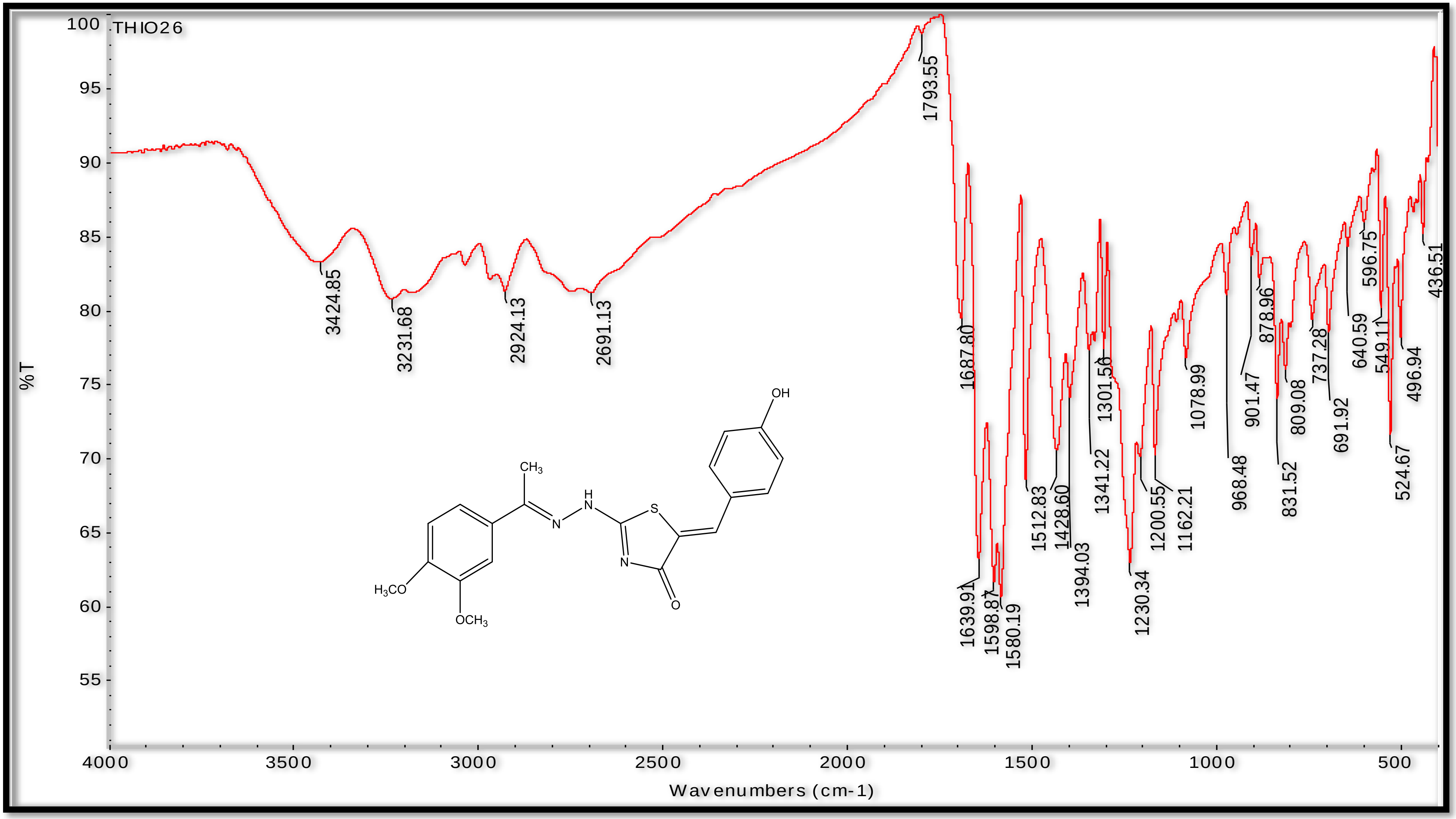

^{1}H NMR of compound (4h)

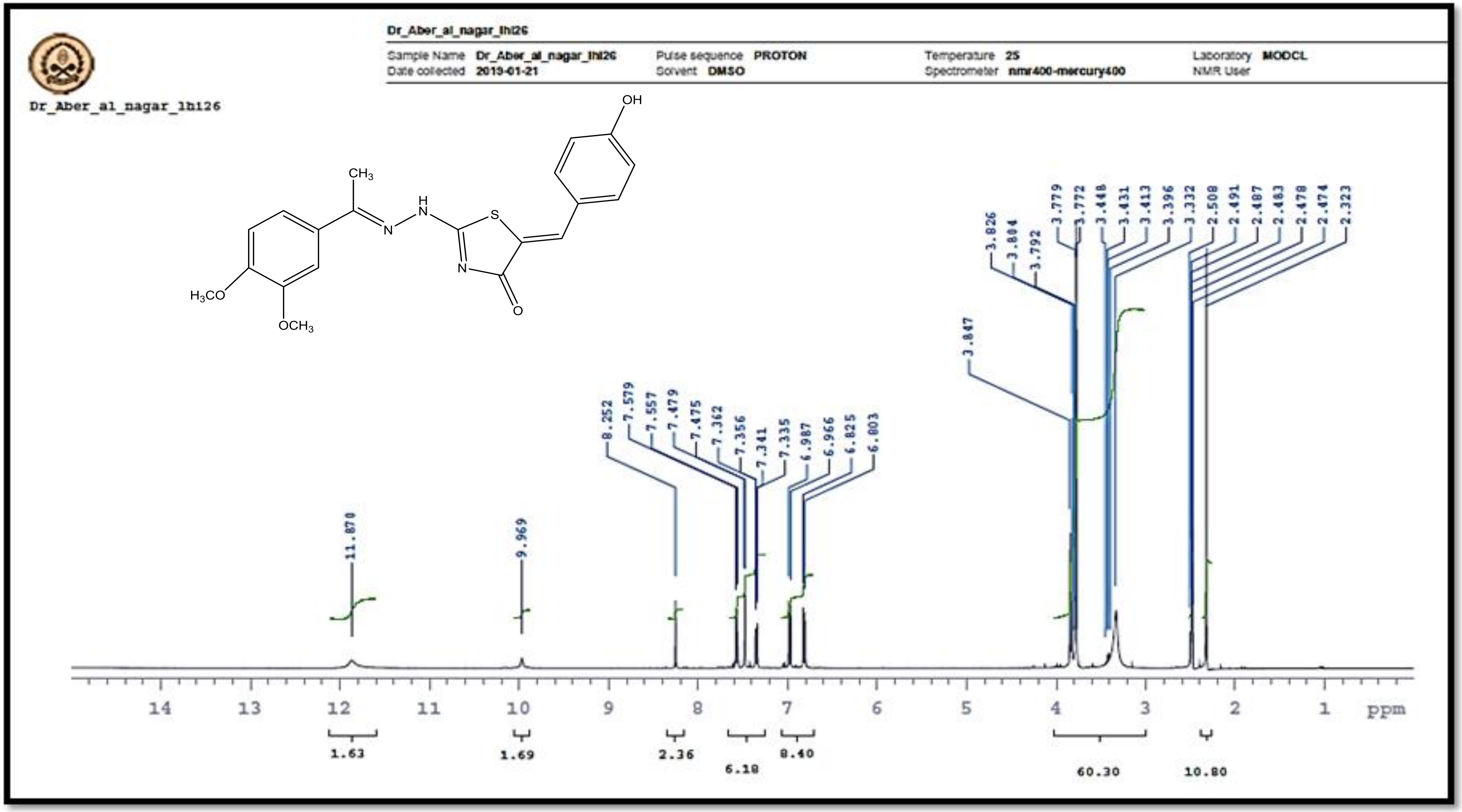

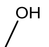

D_2O ^1H NMR of compound (4h)

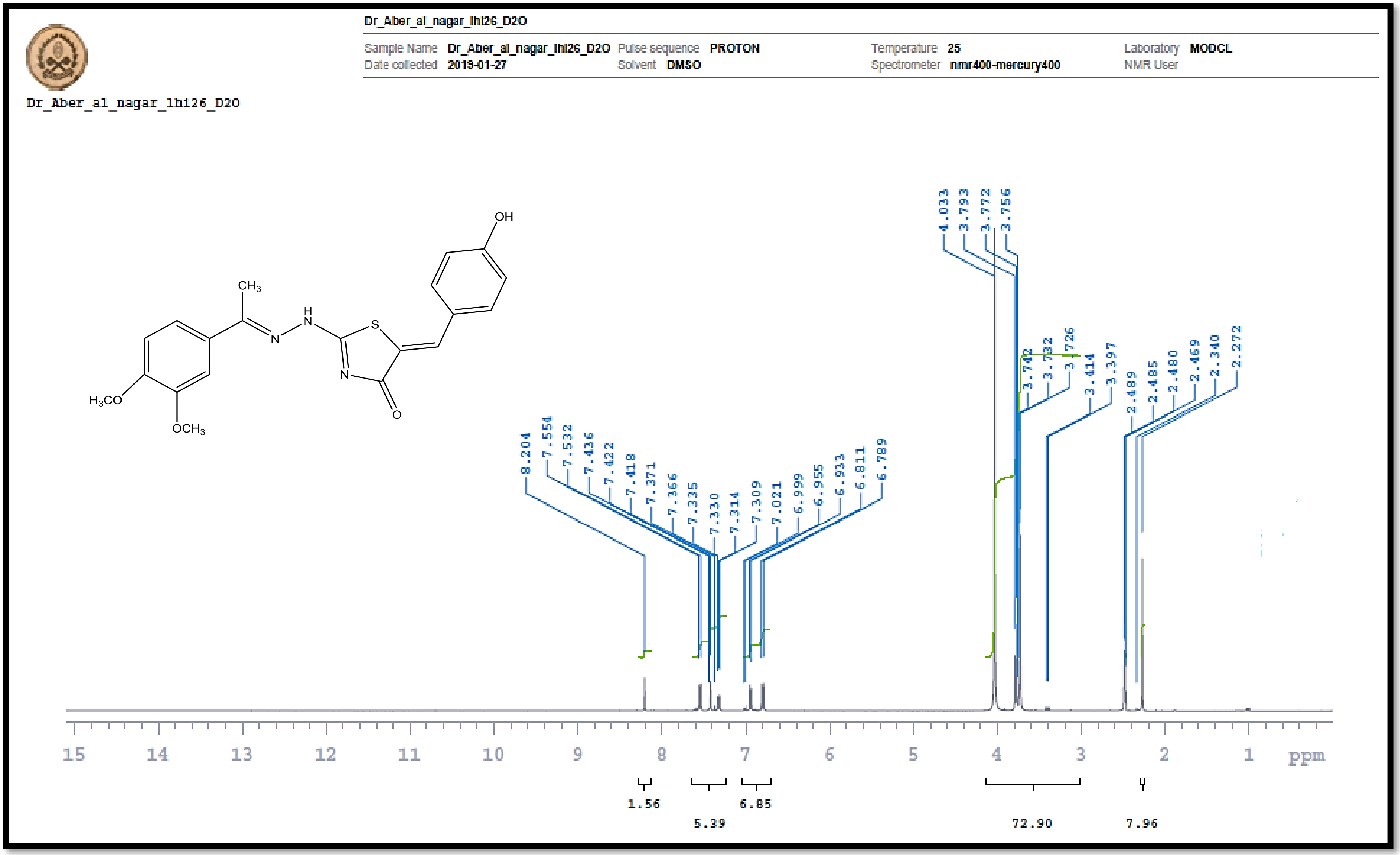

IR of compound (4i)

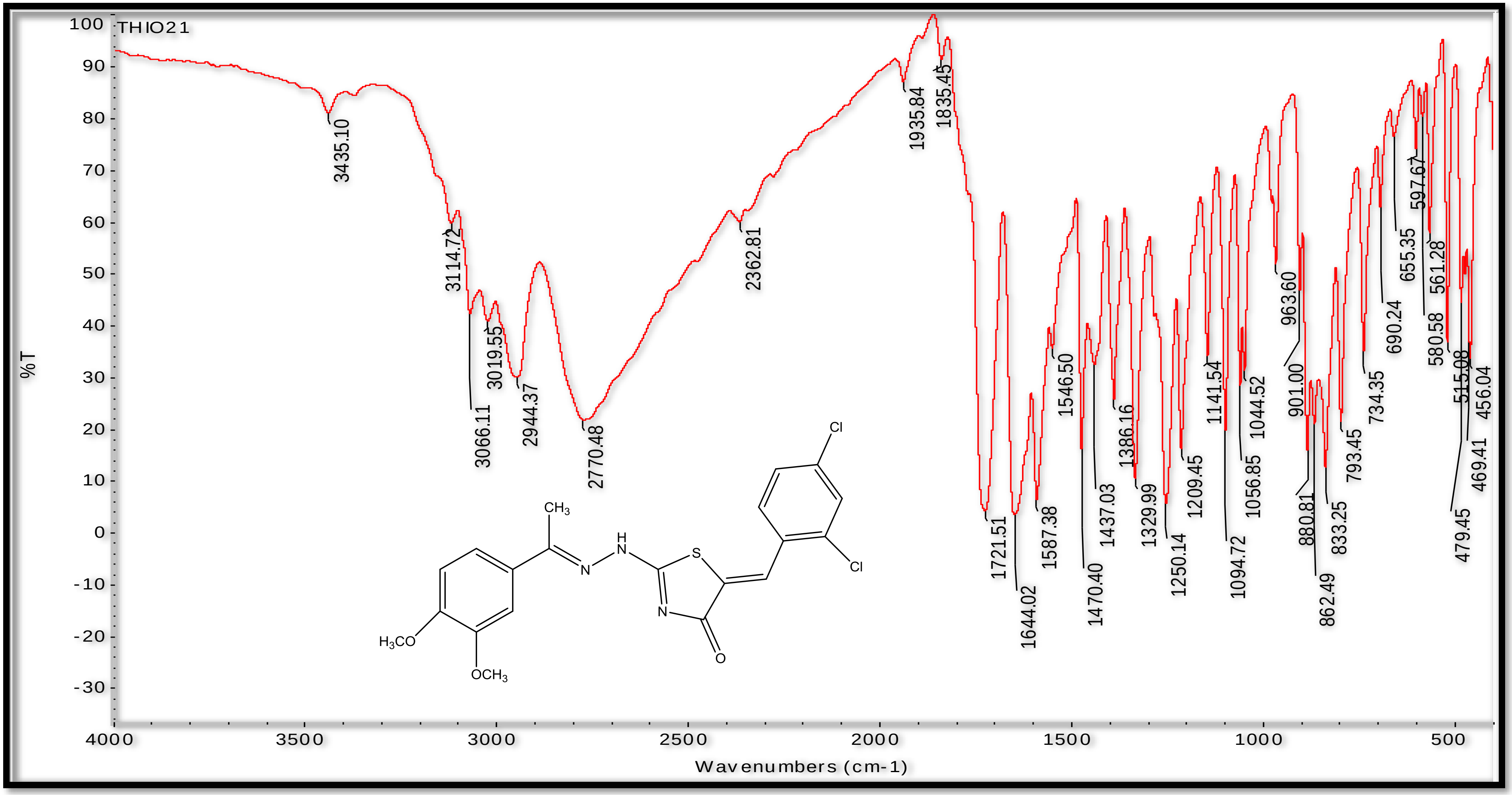

^{1}H NMR of compound (4i)

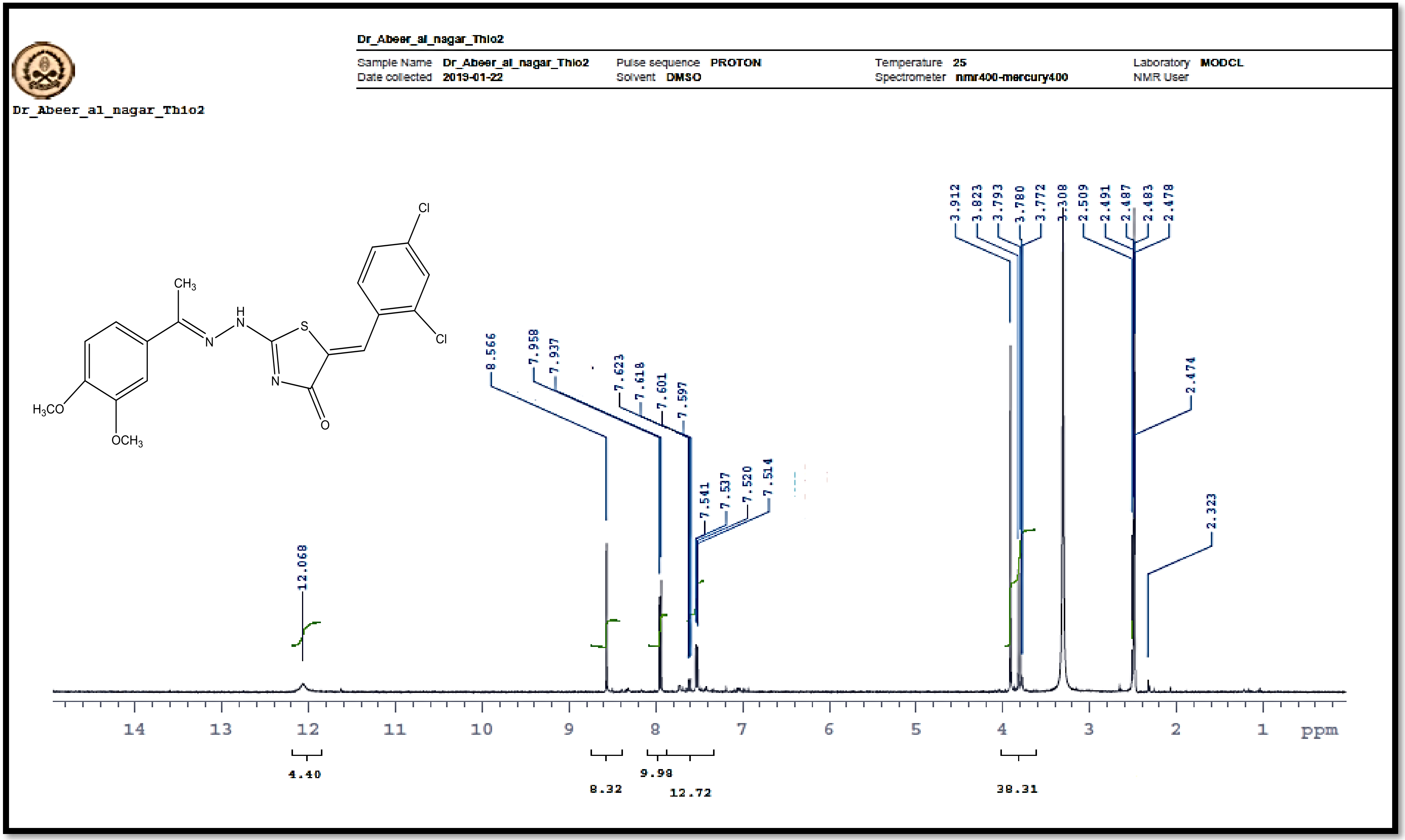

IR of compound (4j)

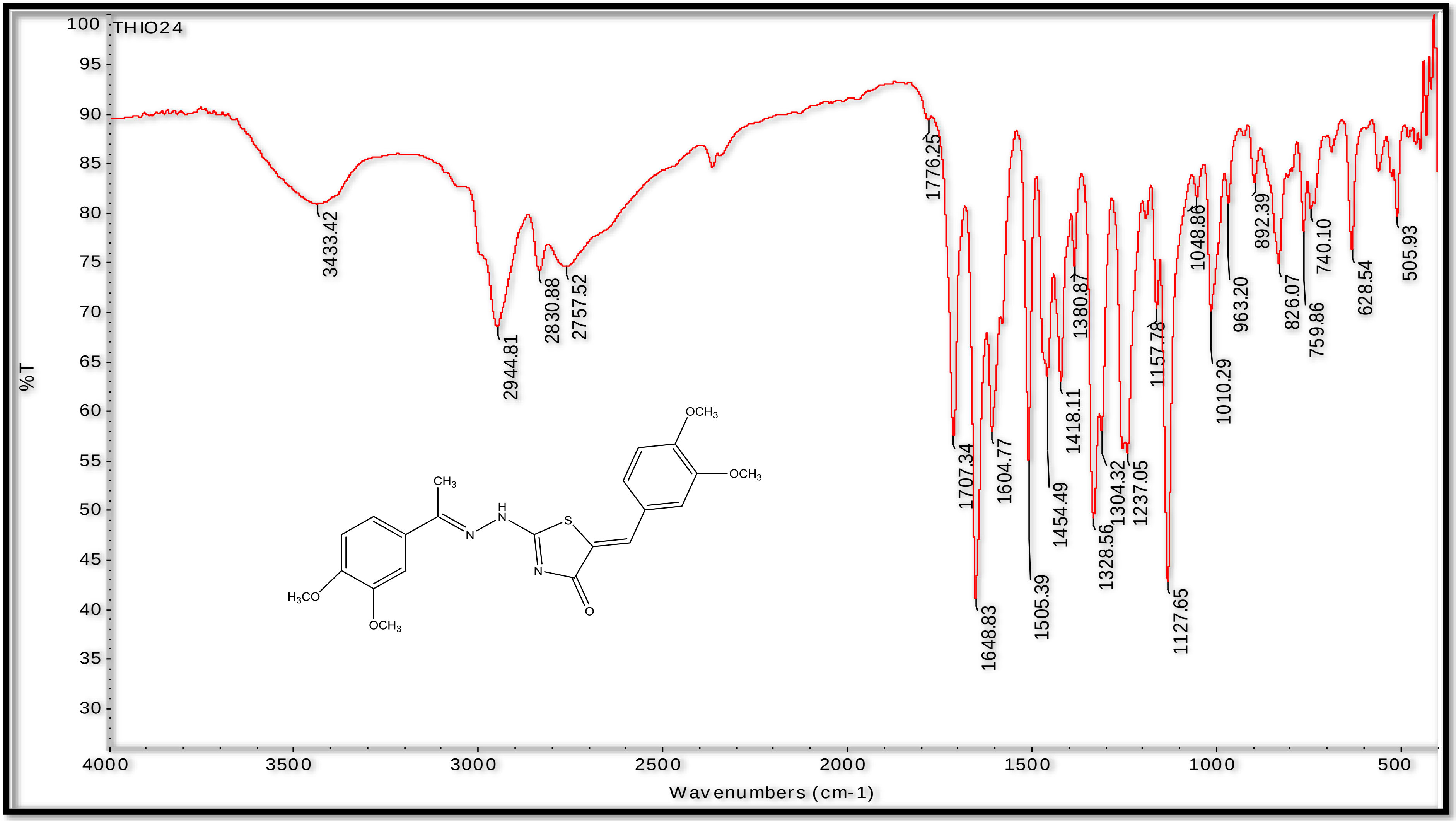

^{1}H NMR of compound (4j)

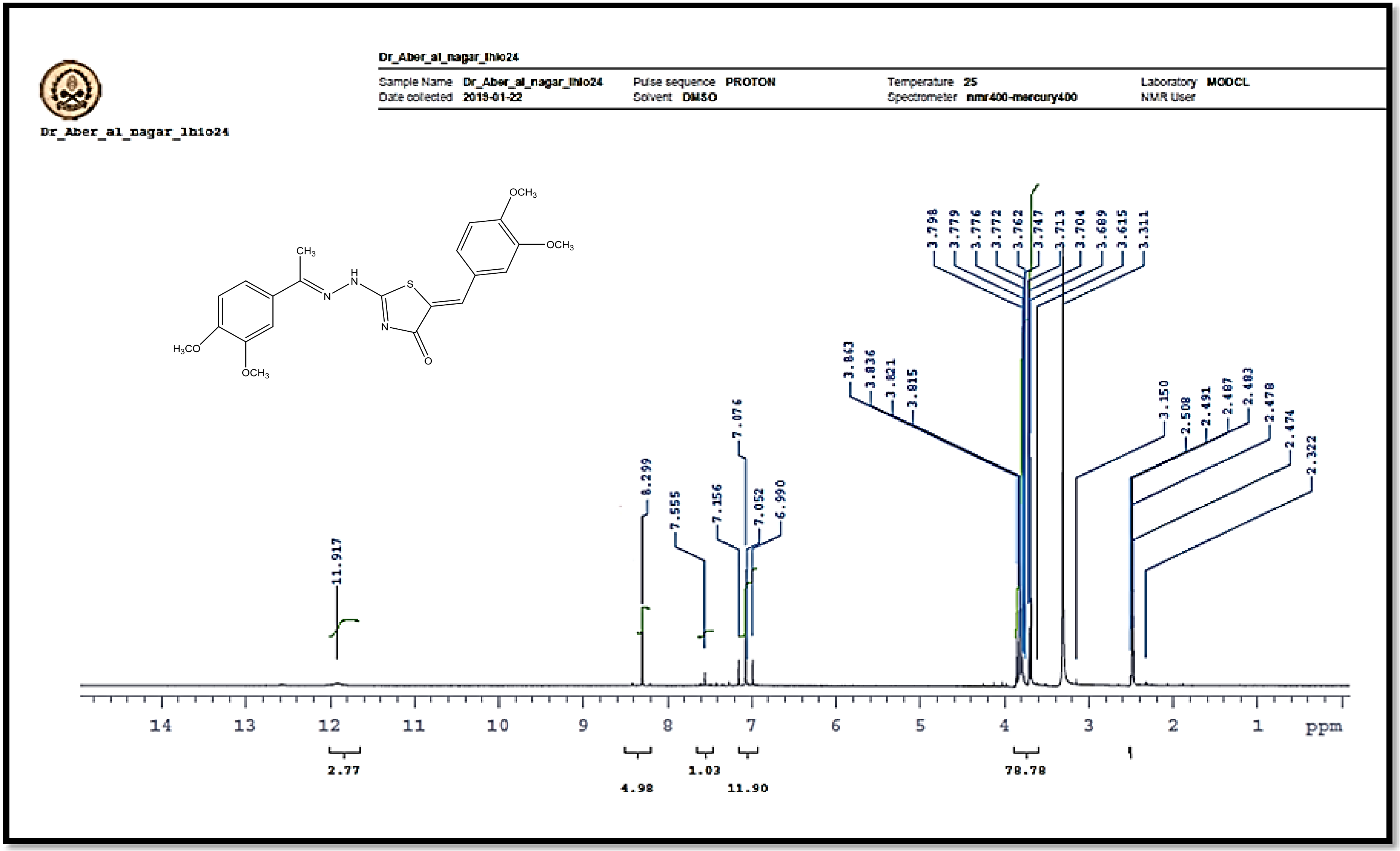

D2O ^{1}H NMR of compound(4j)

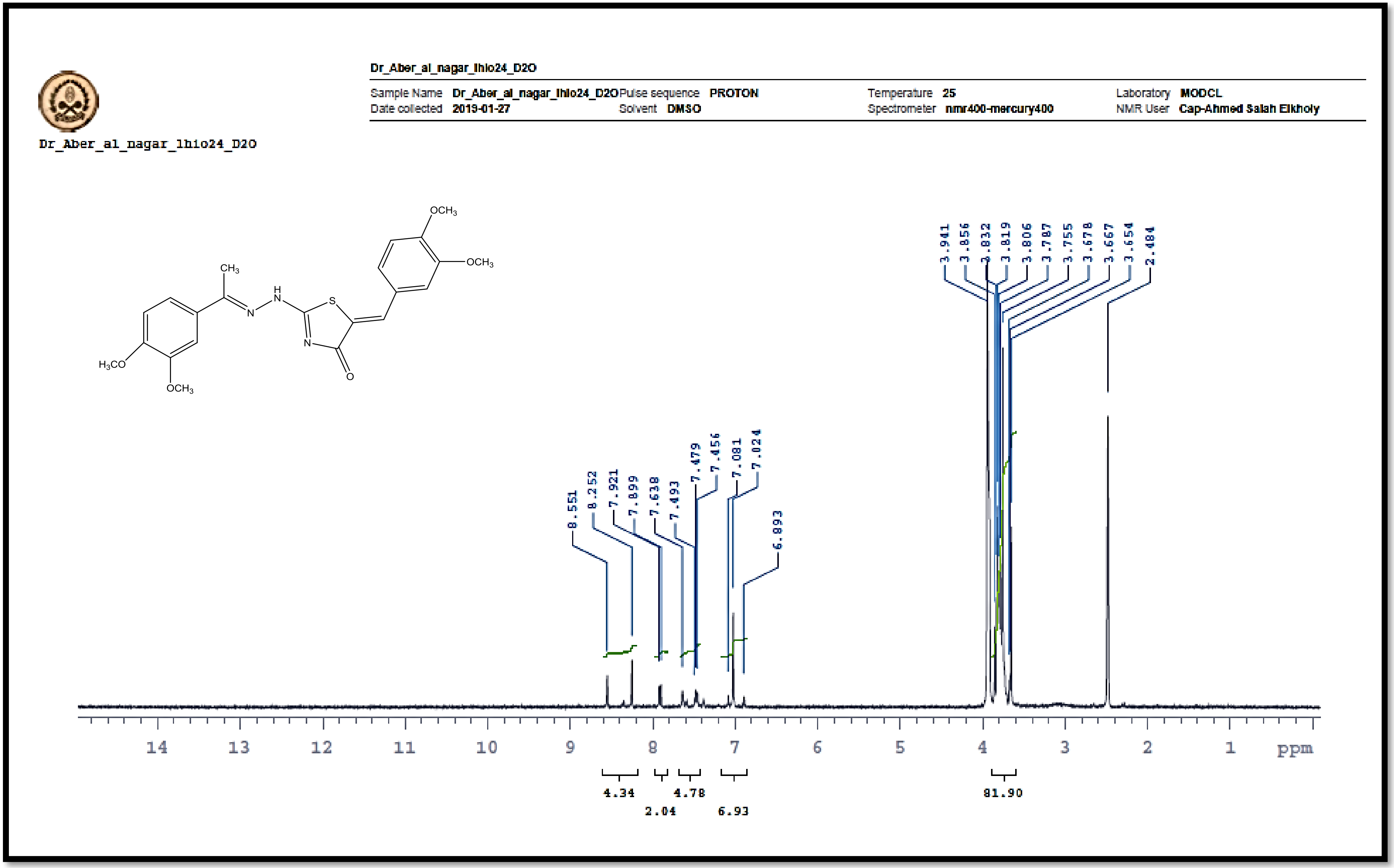

IR of compound (4k)

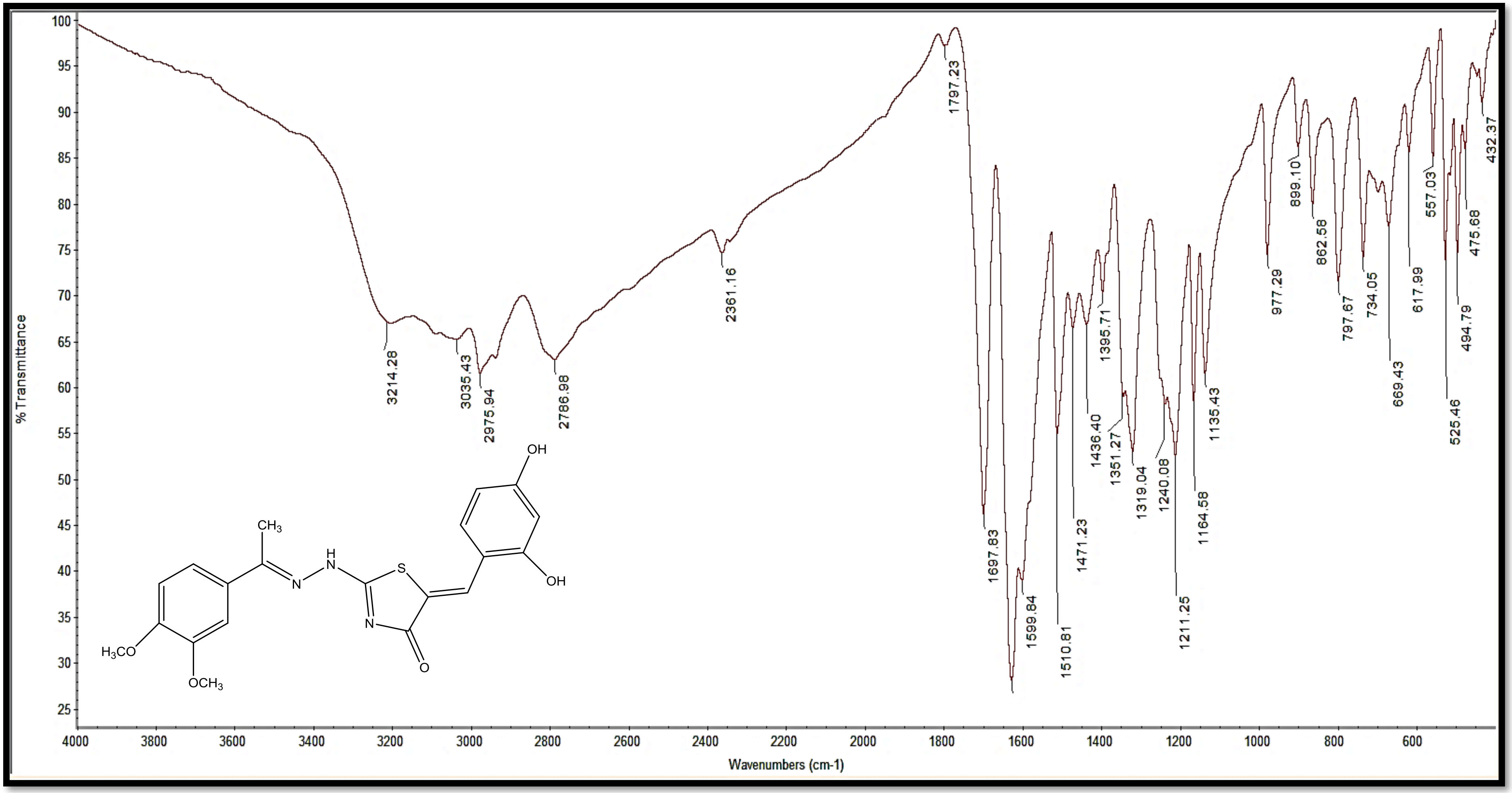

^{1}H NMR of compound (4k)

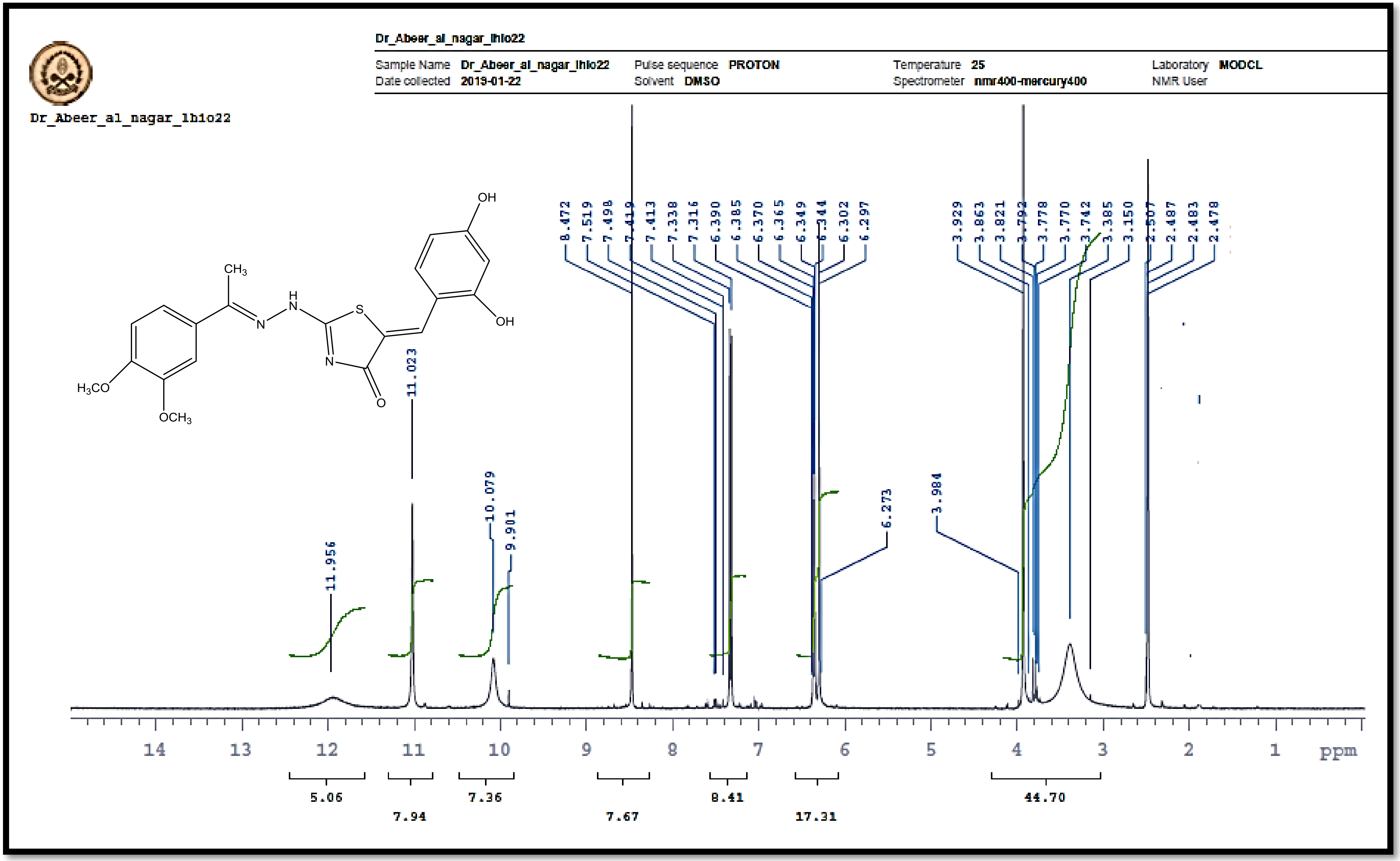

D$_2$O ^{1}H NMR of compound (4k)

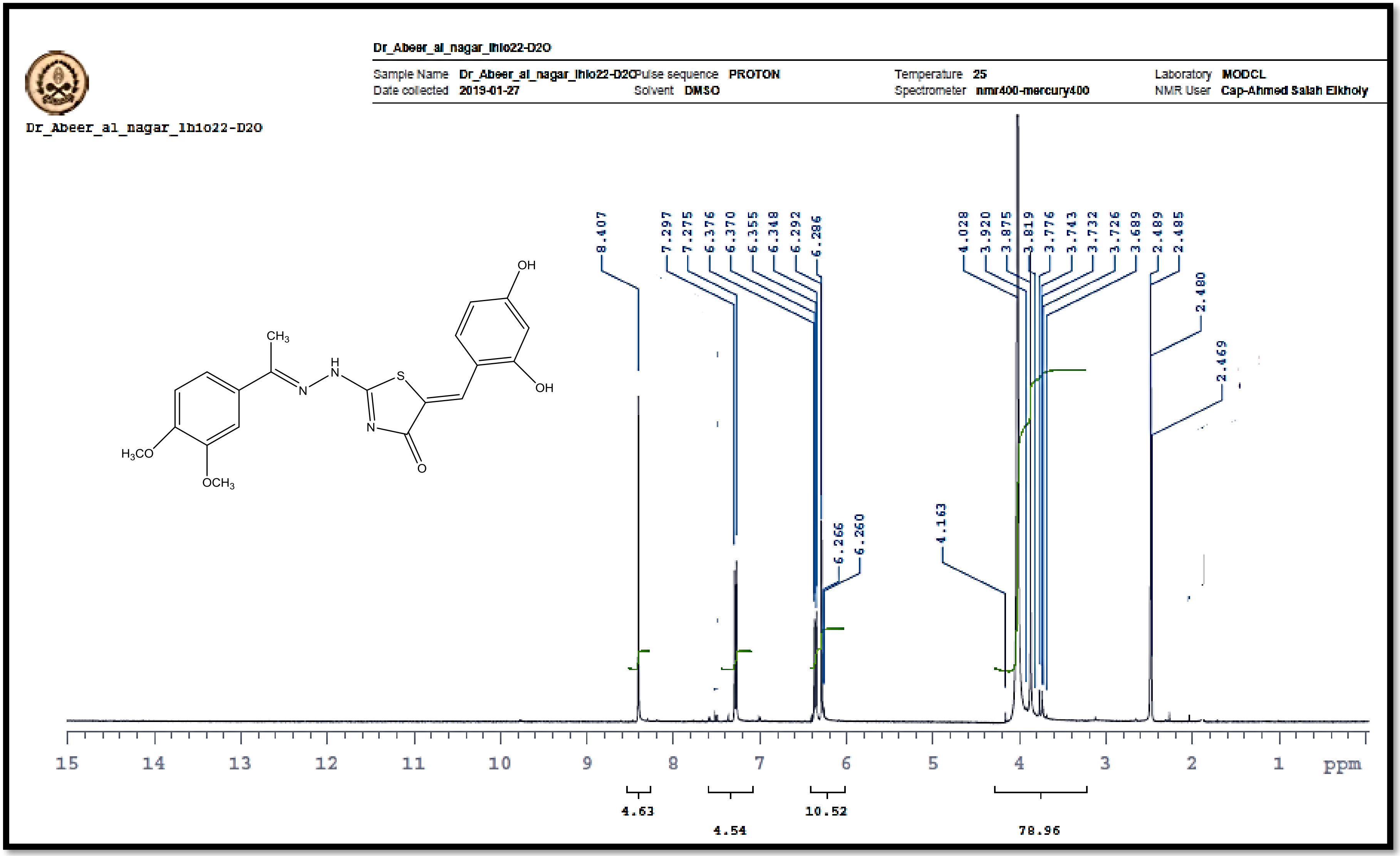

IR of compound (5a).

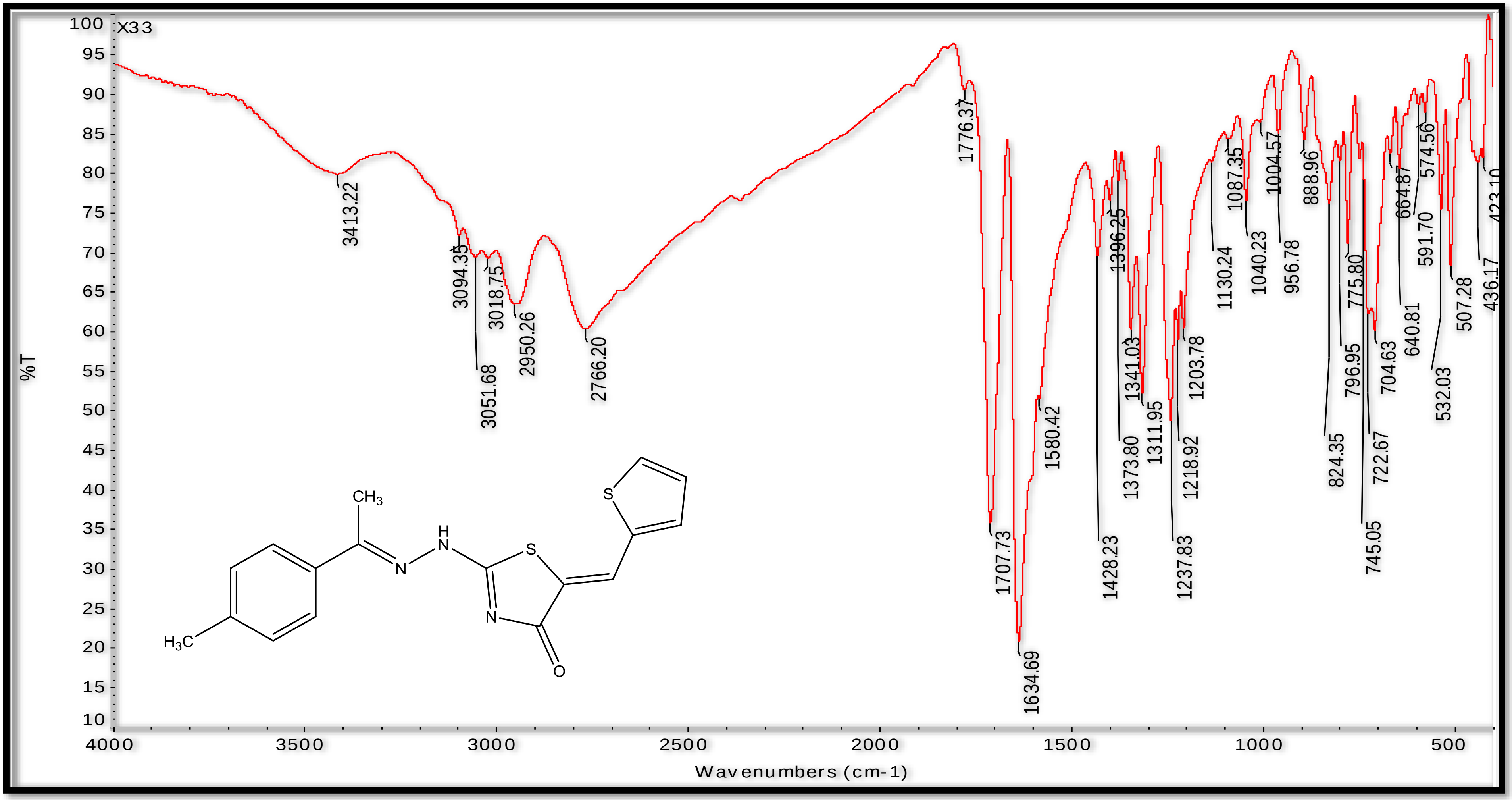

^{1}H NMR of compound (5a)

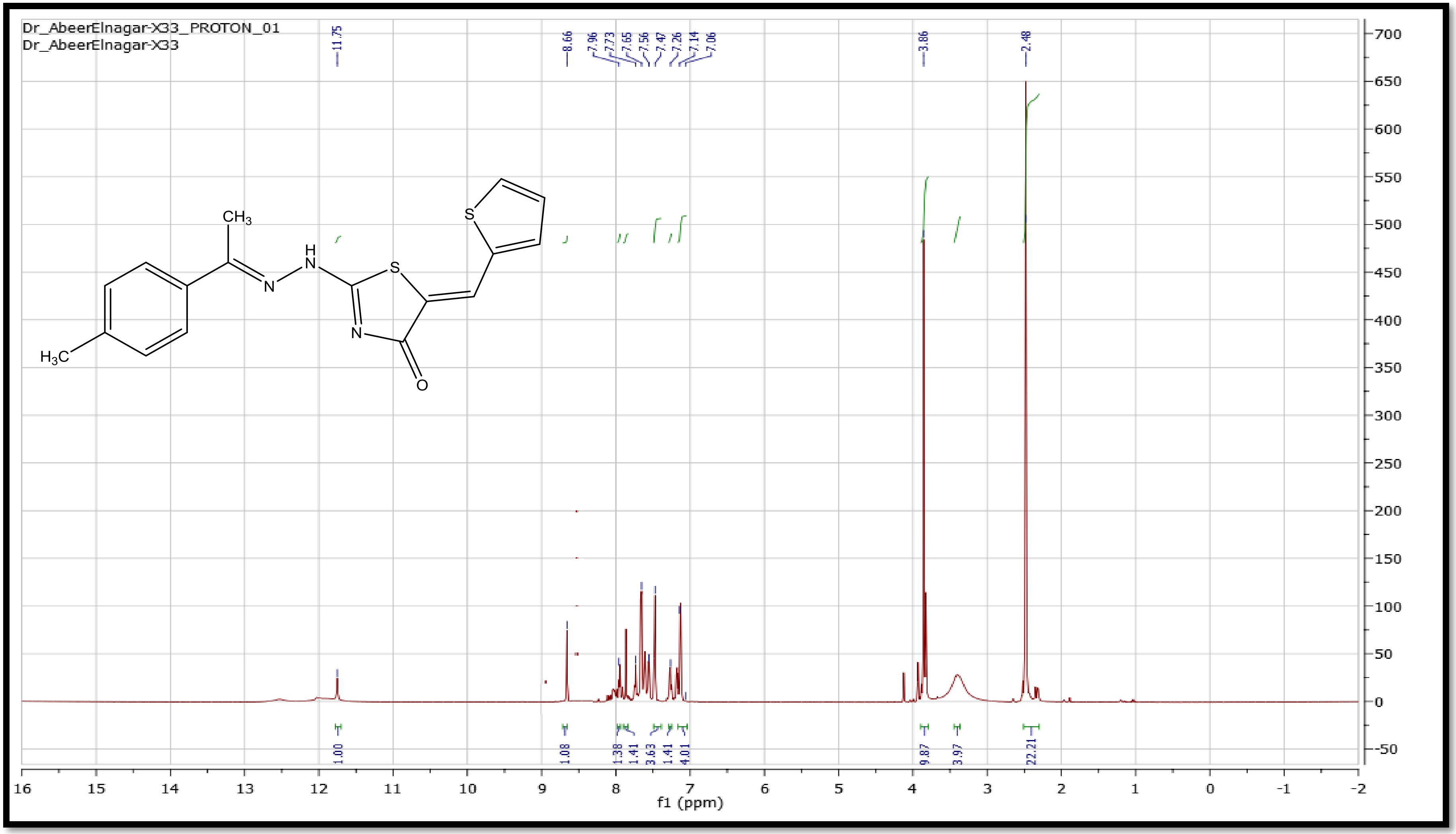

IR of compound (5b)

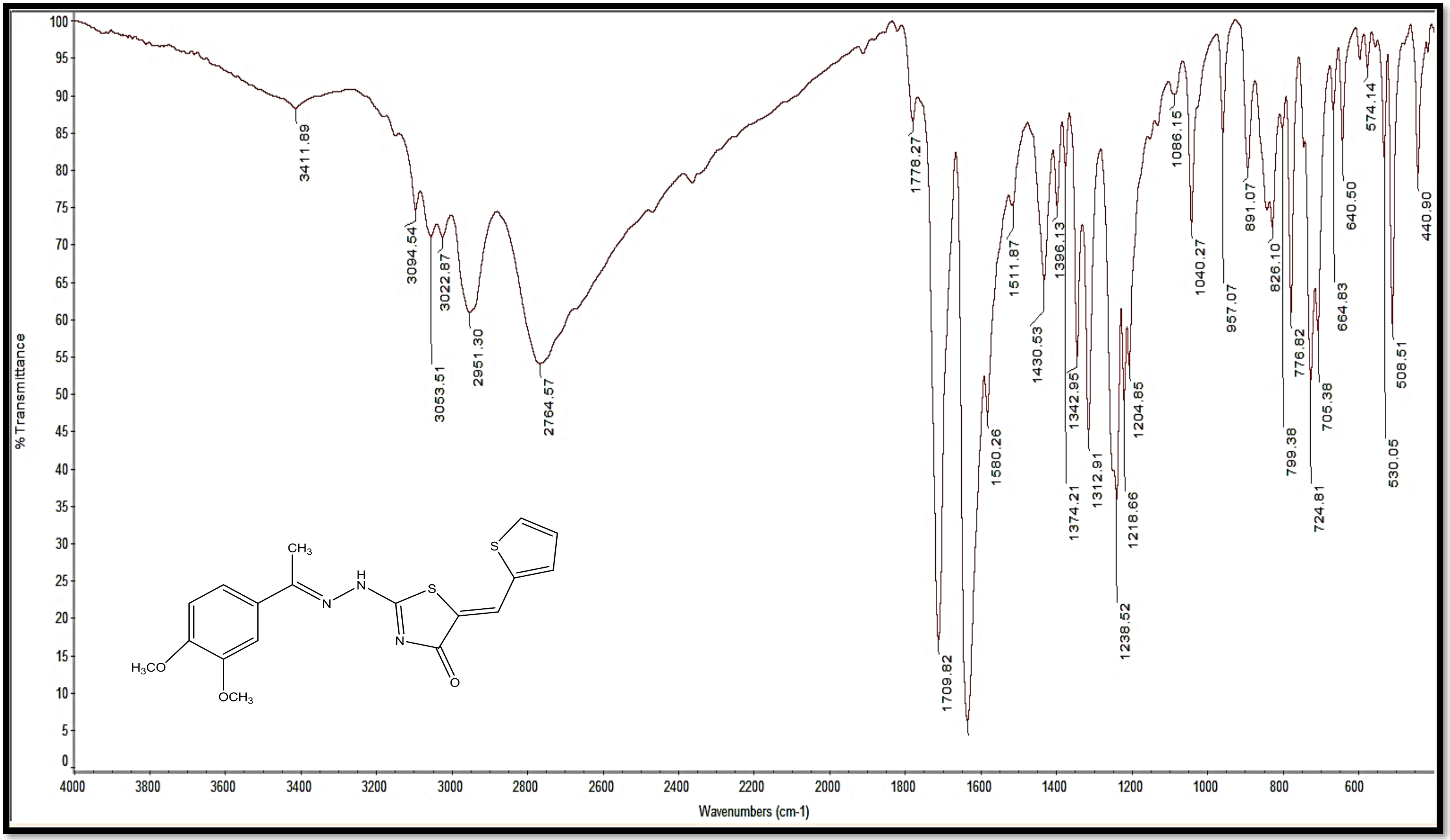

^{1}H NMR of compound (5b)

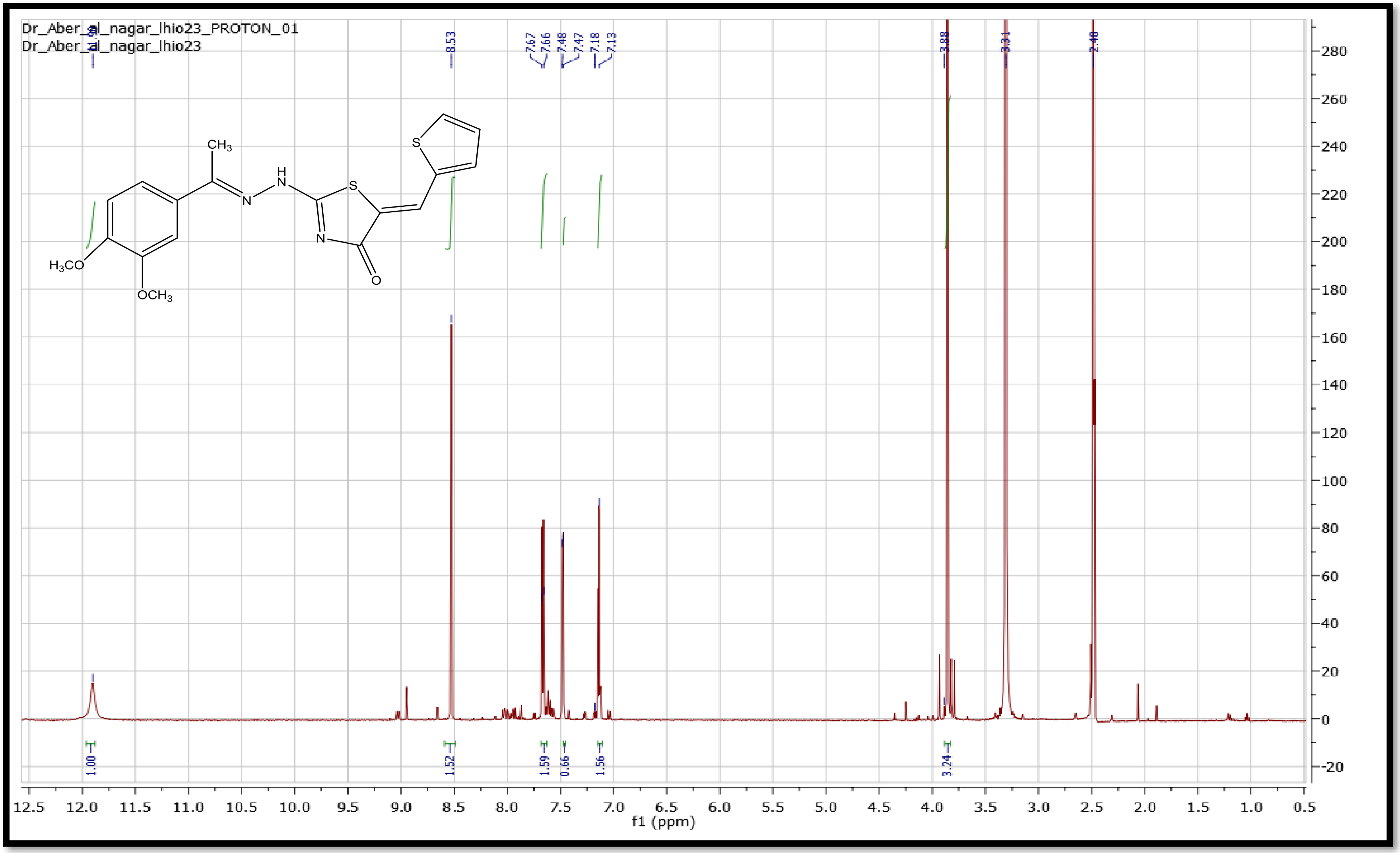

^{1}H NMR of compound (5b)

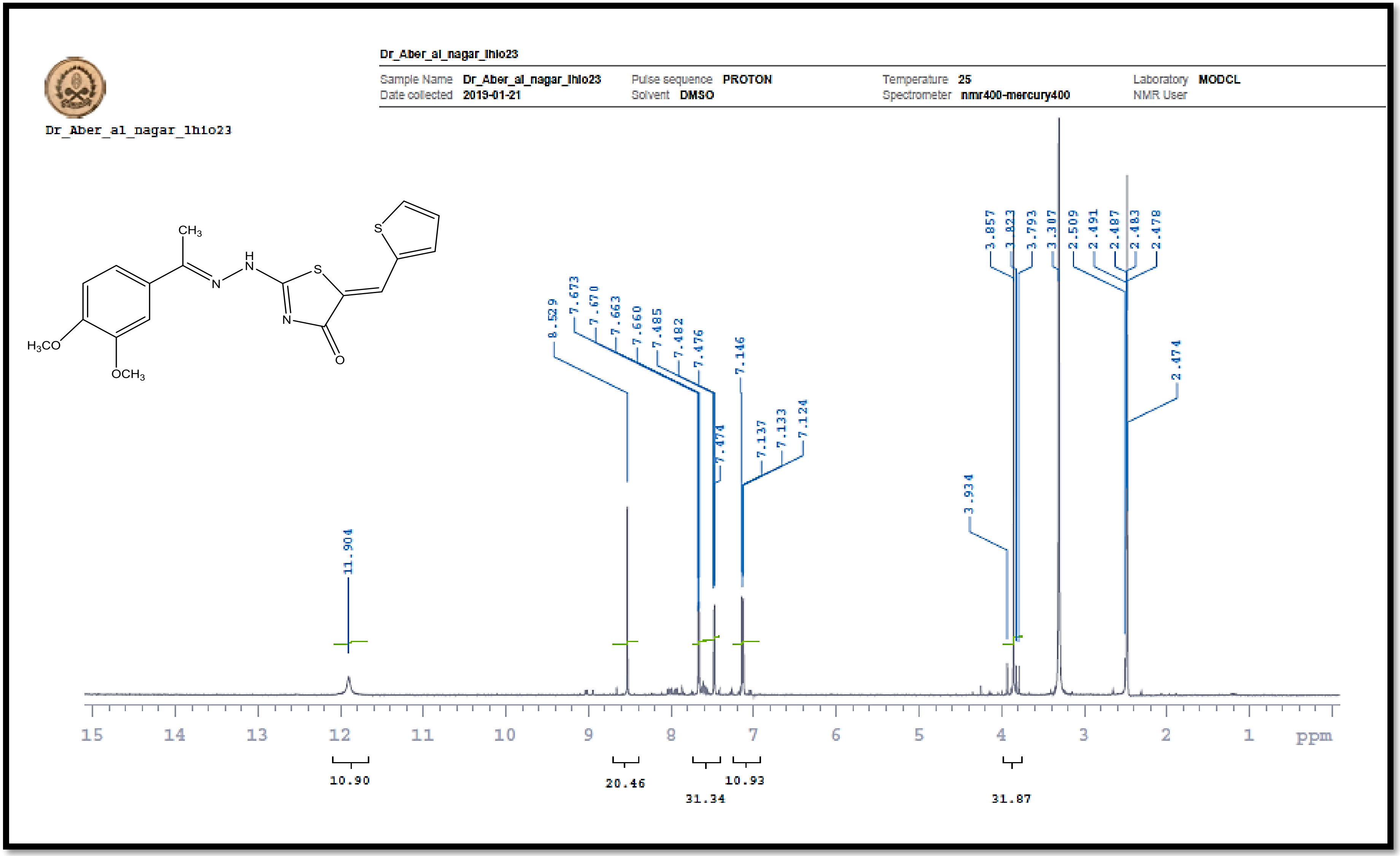

^{1}H NMR of compound (5b)

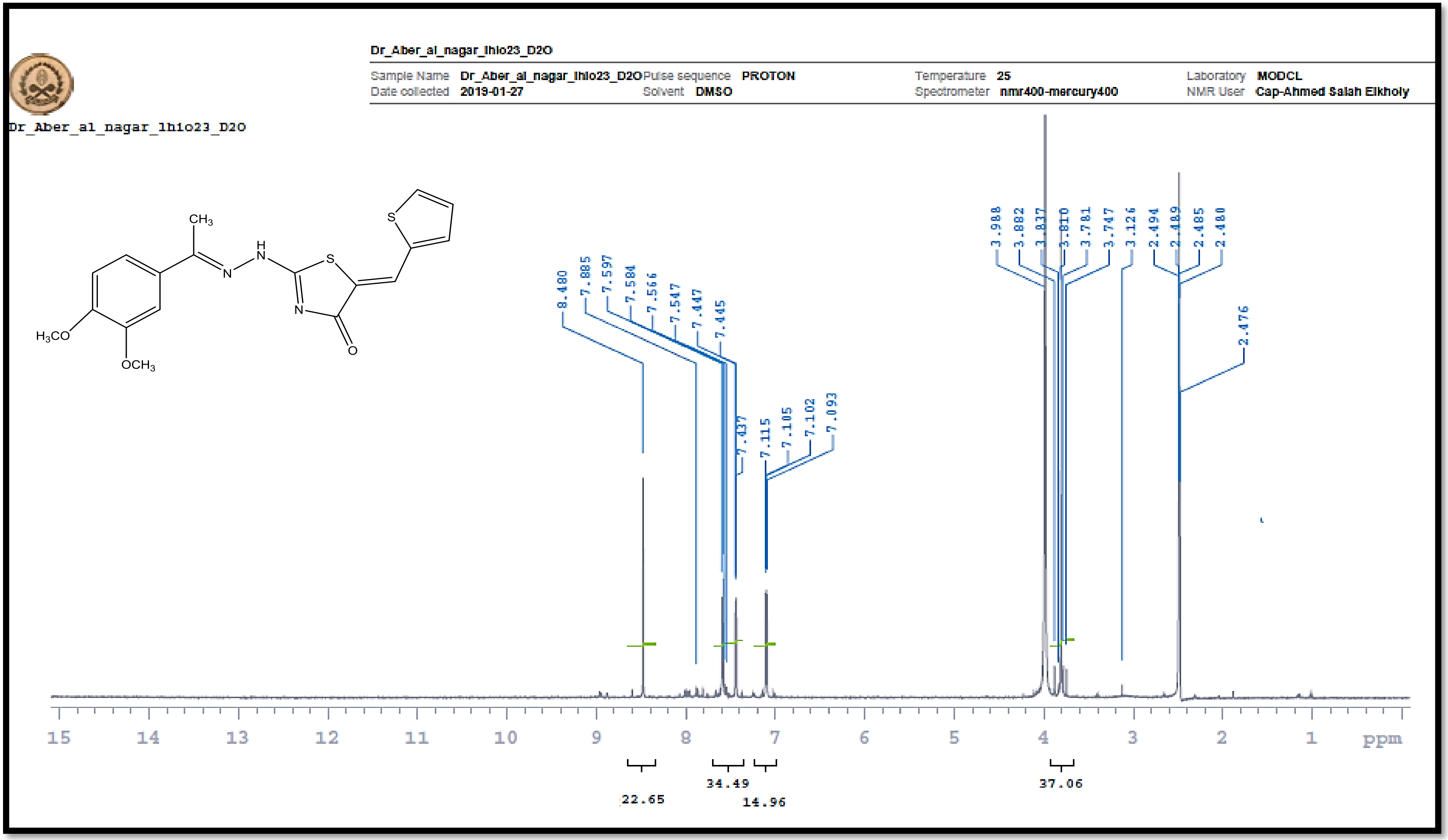

IR of compound (8a)

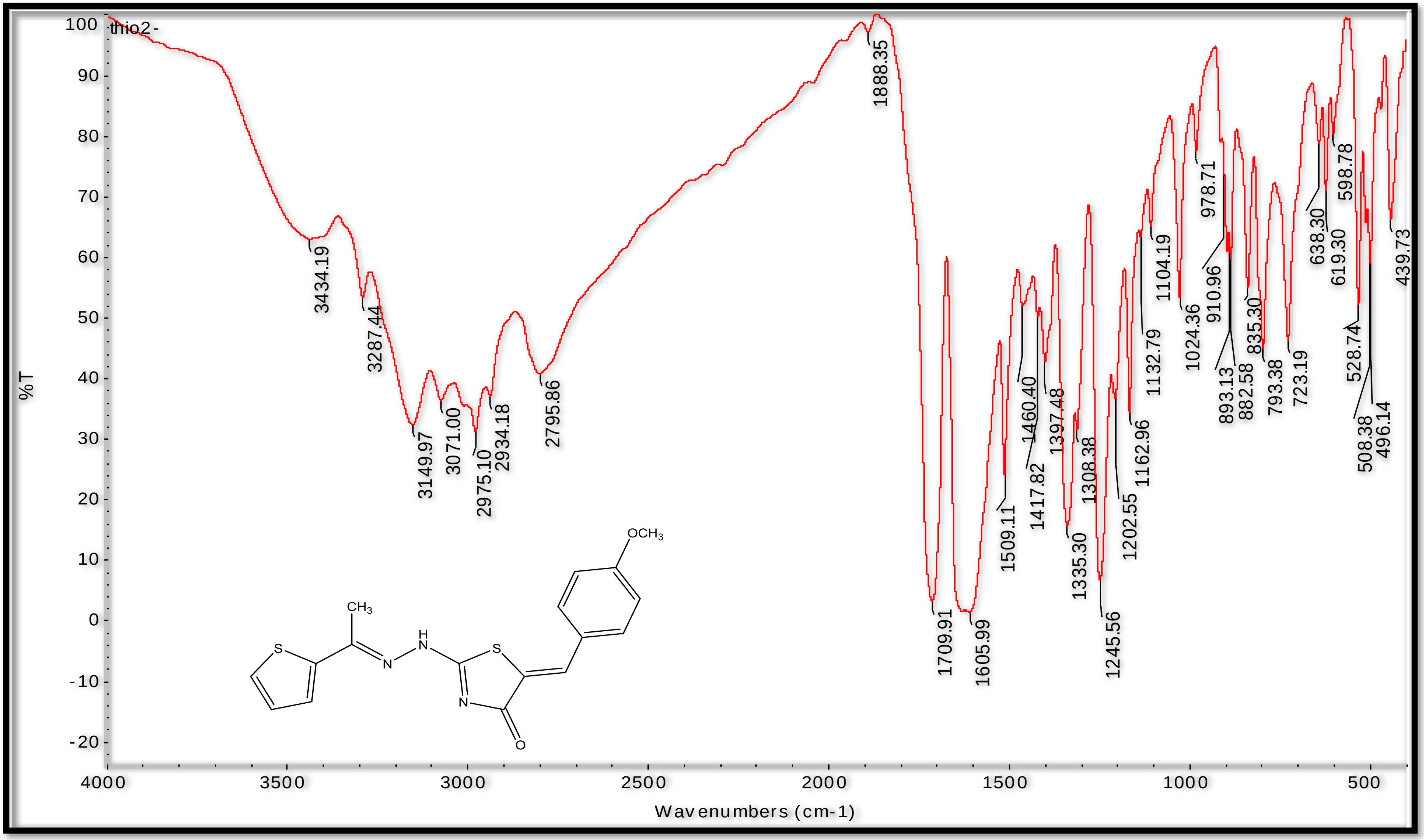

^{1}H NMR of compound (8a)

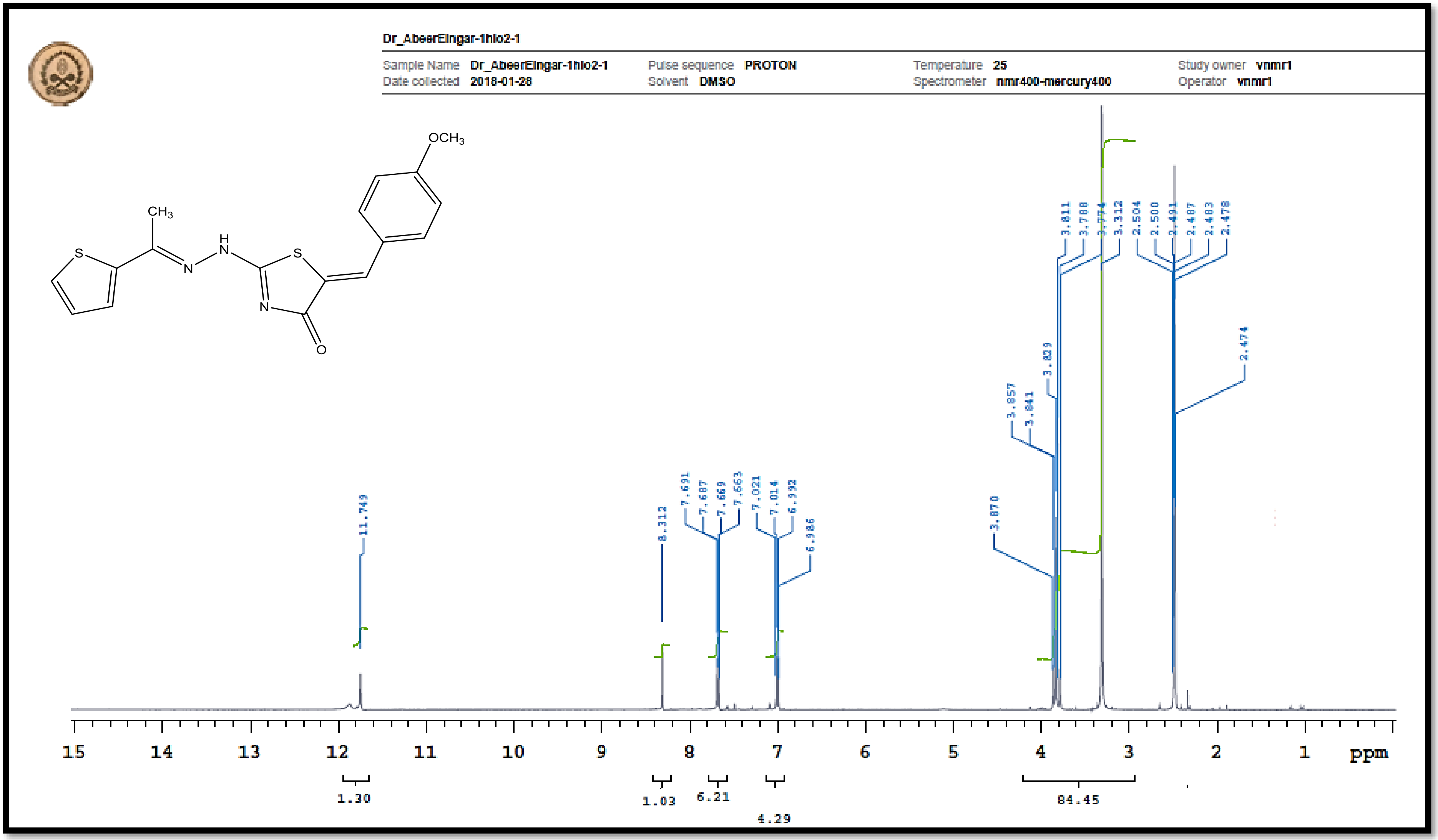

IR of compound (8b)

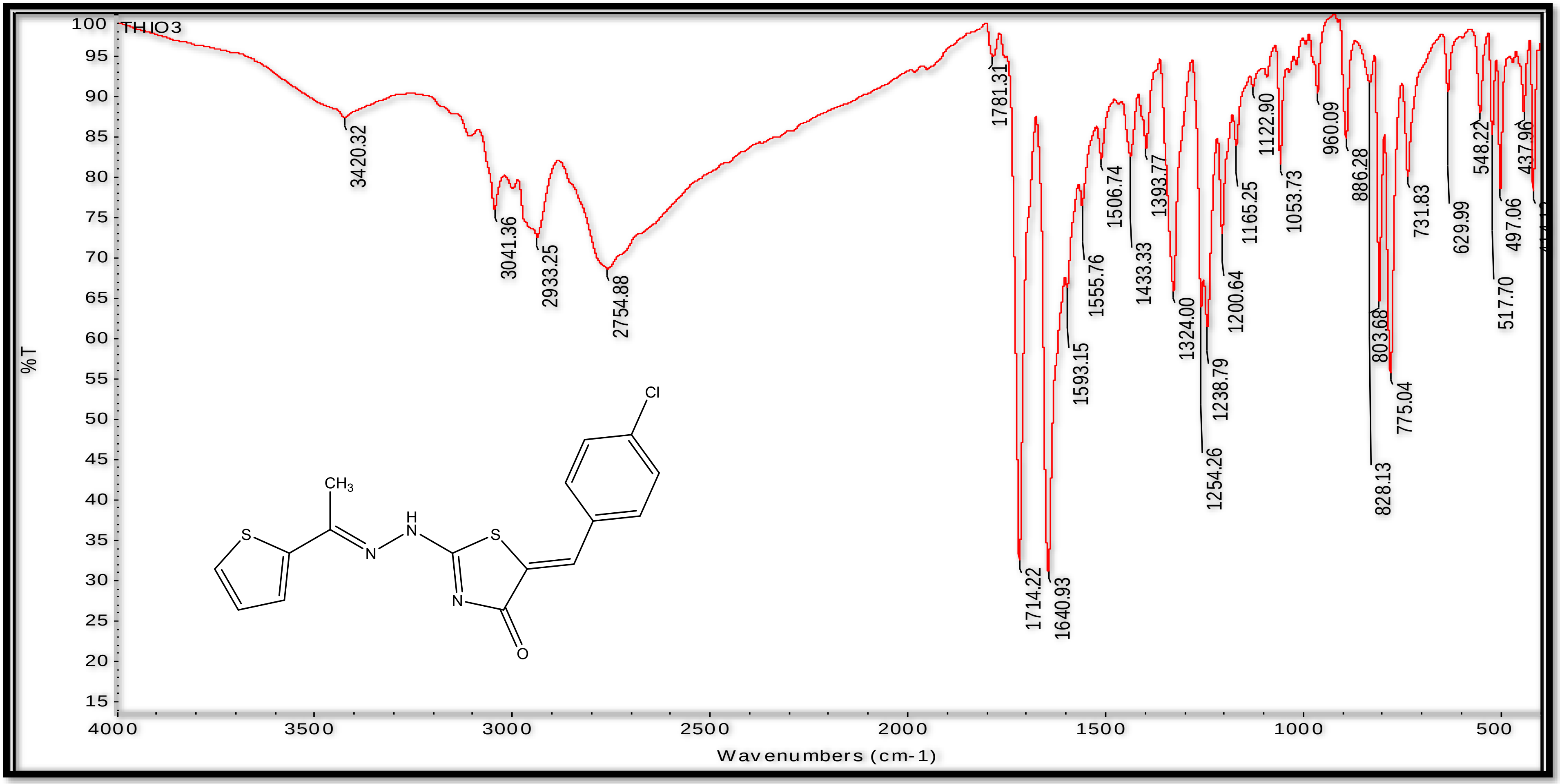

^{1}H NMR of compound (8b)

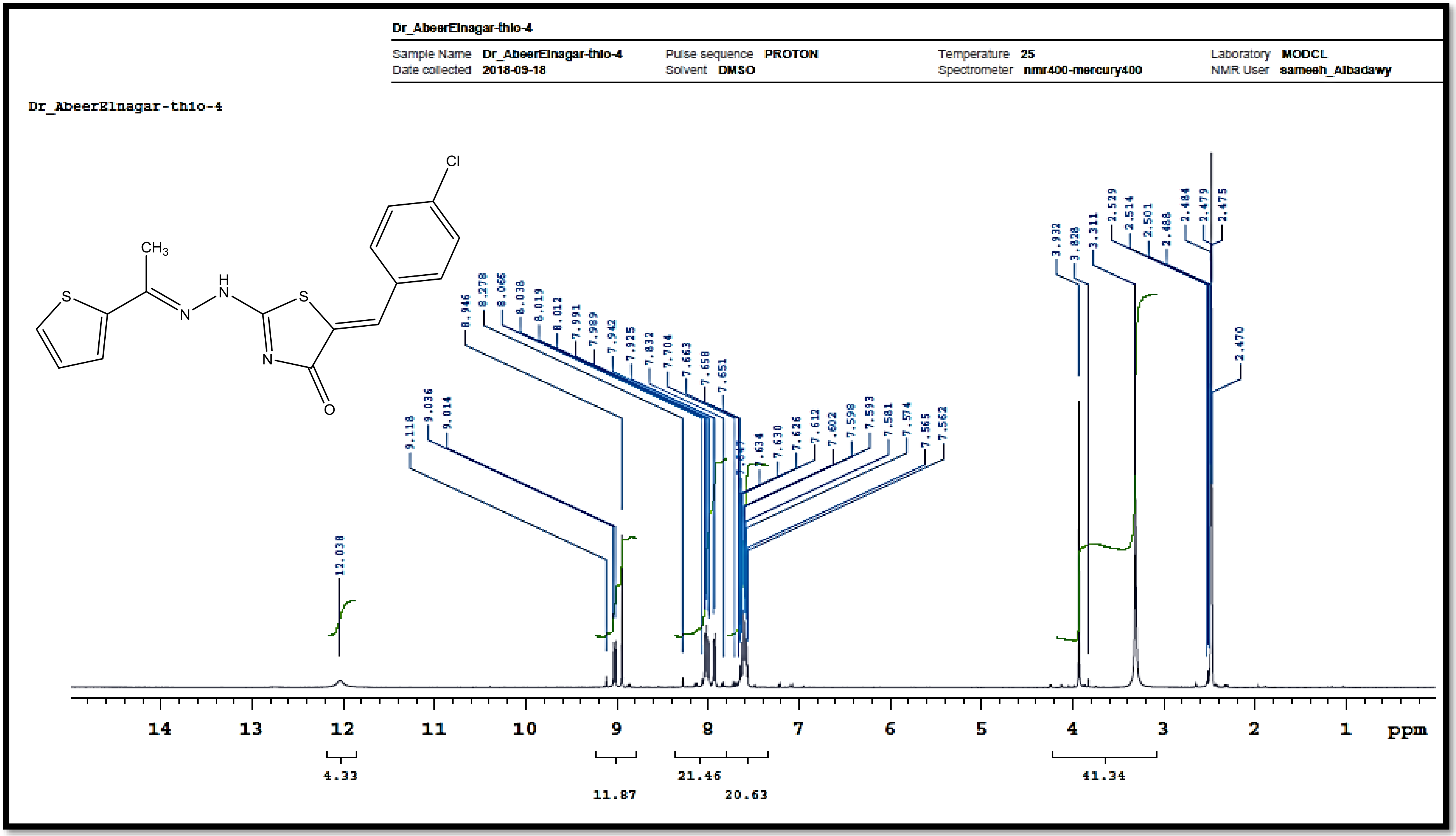

^{1}H NMR of compound (8b)

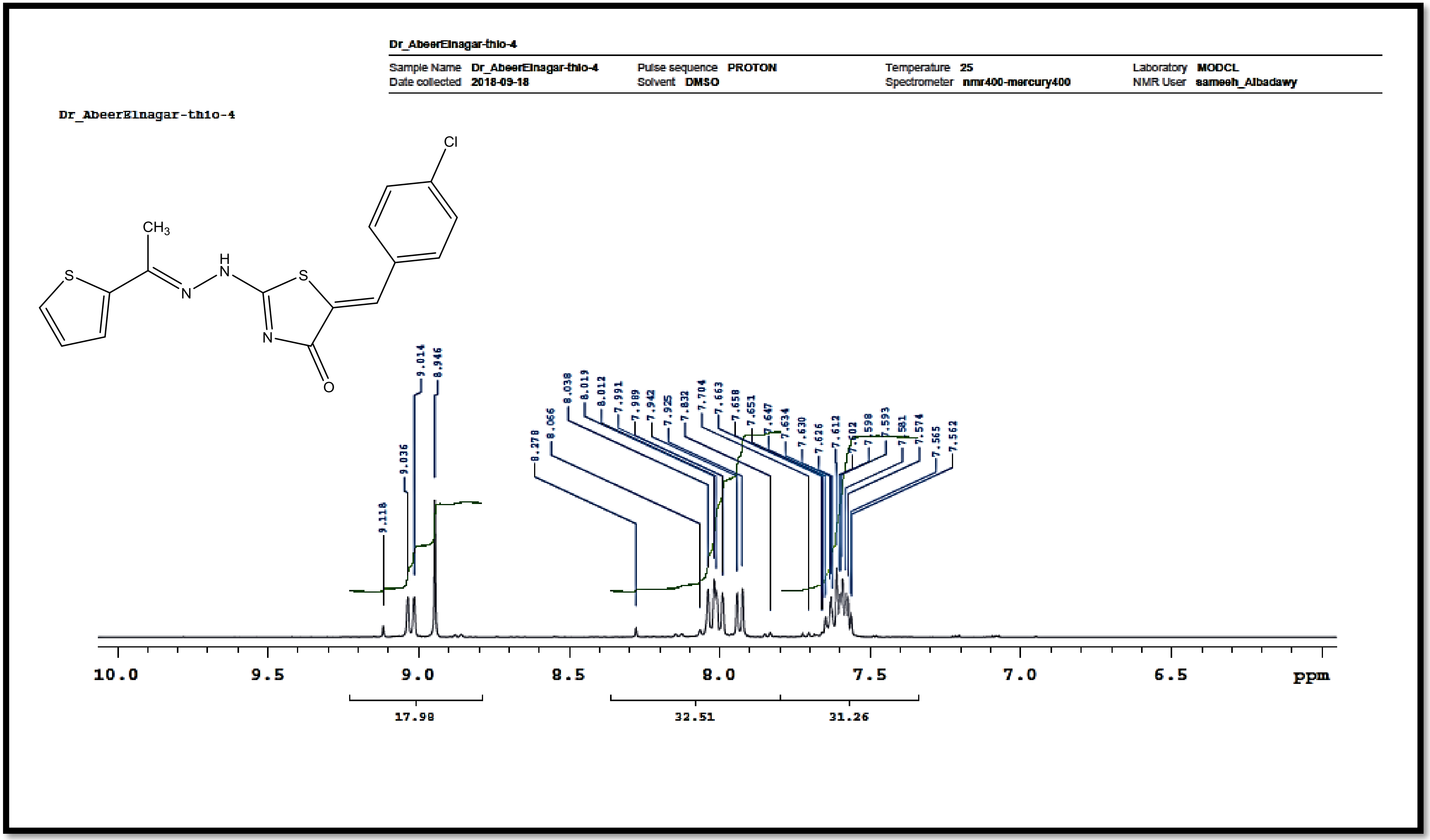

^{1}H NMR of compound (8b)

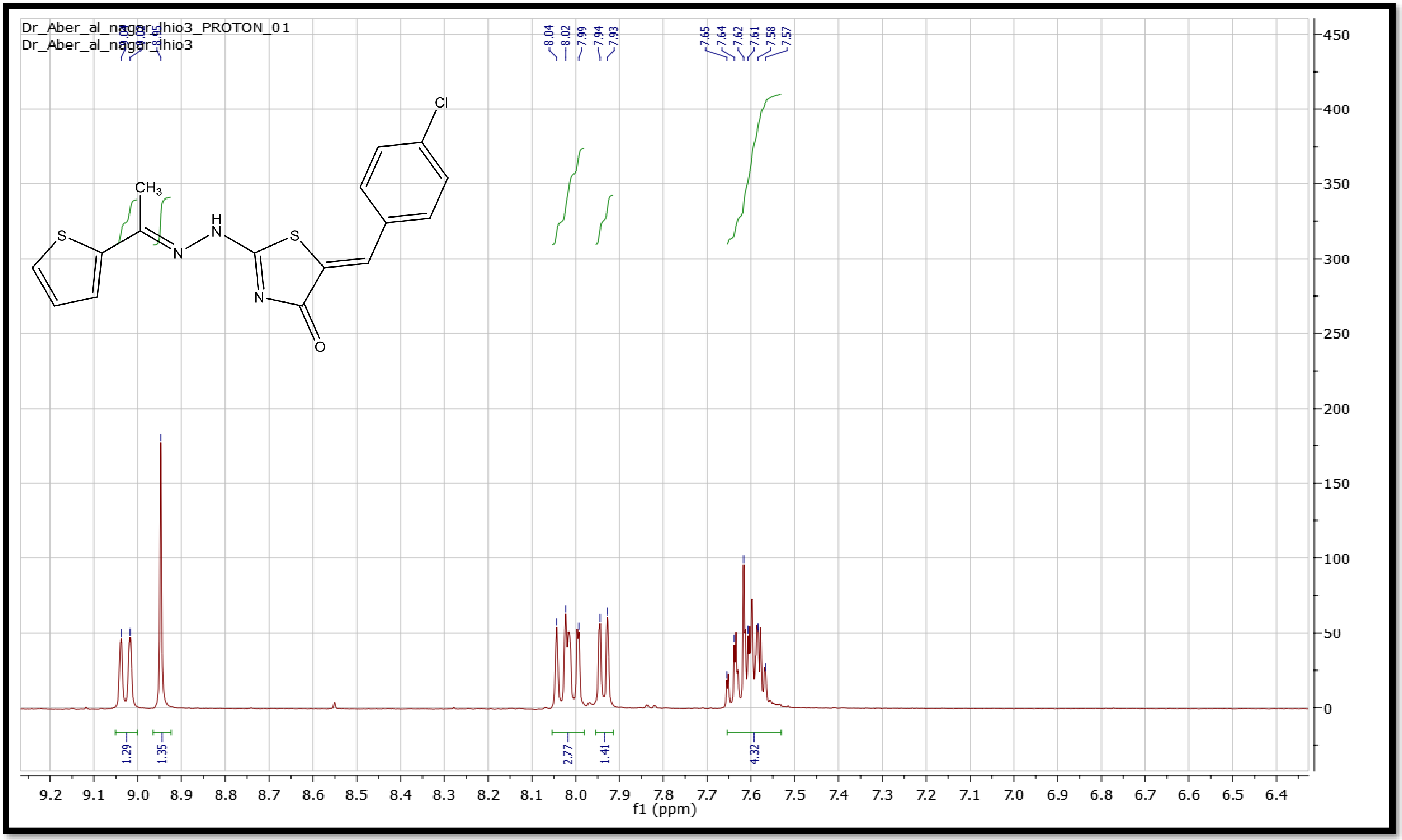

IR of compound (8c)

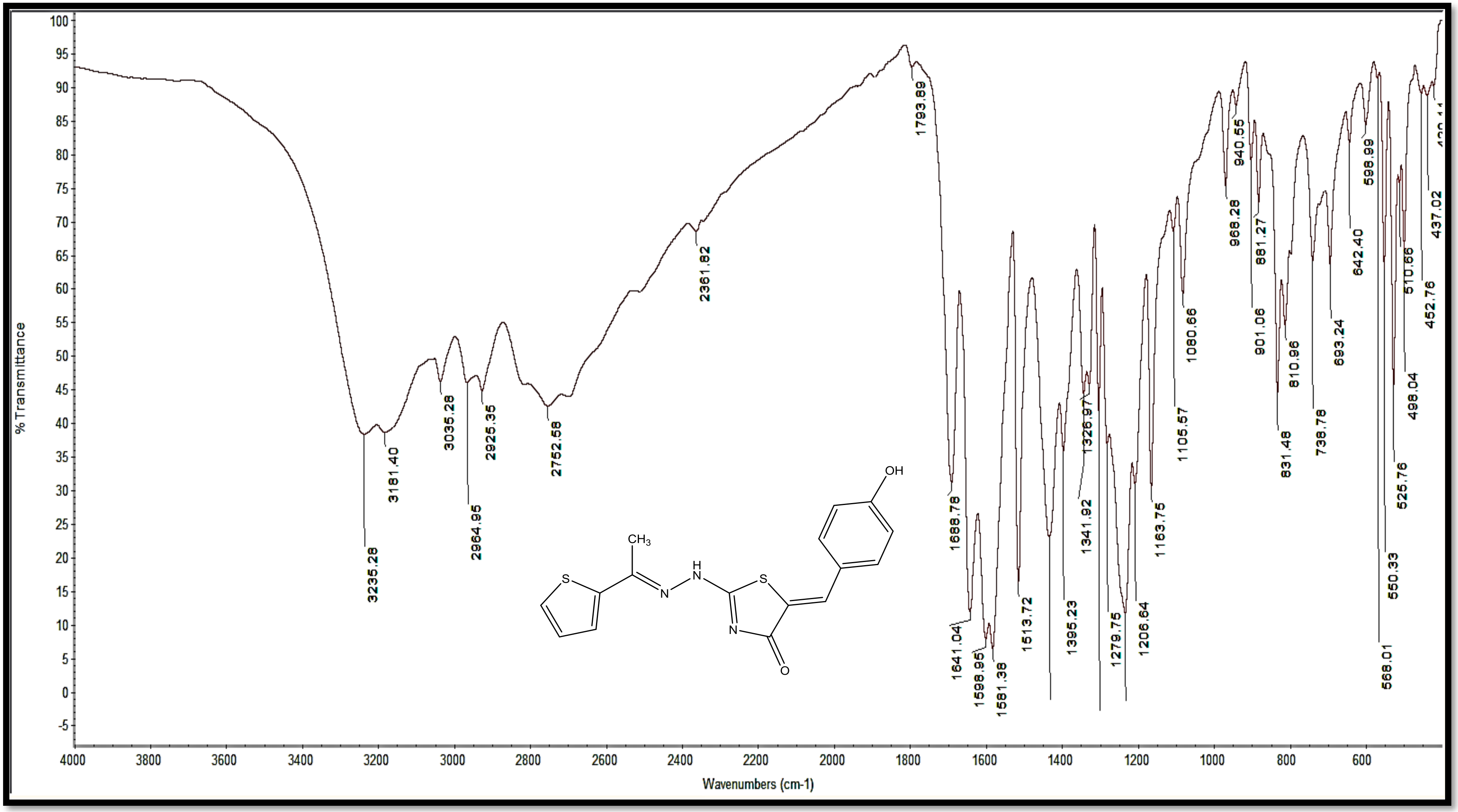

^{1}H NMR of compound (8c)

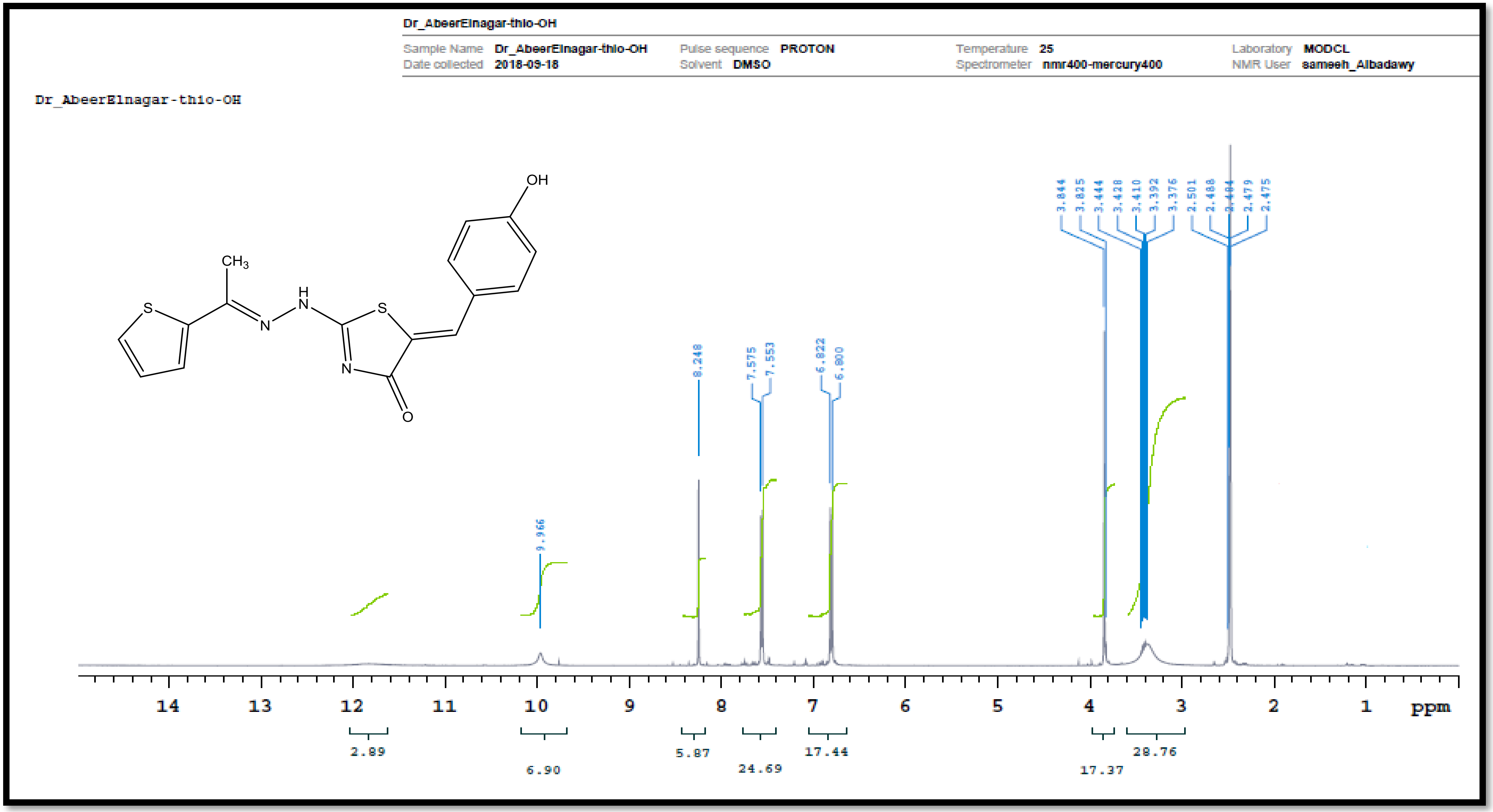

D$_2$O ^{1}H NMR of compound (8c)

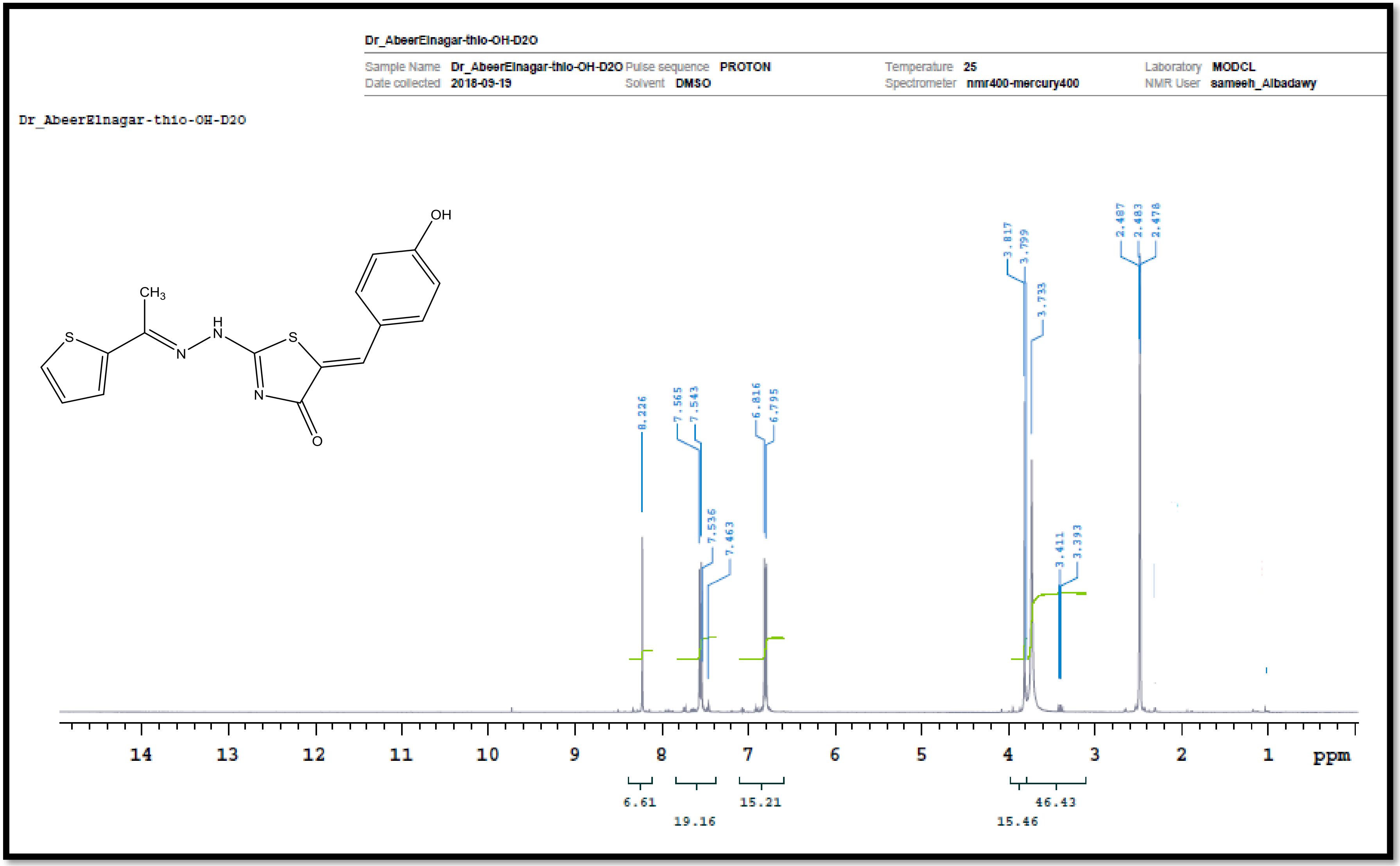

^{1}H NMR of compound (8c)

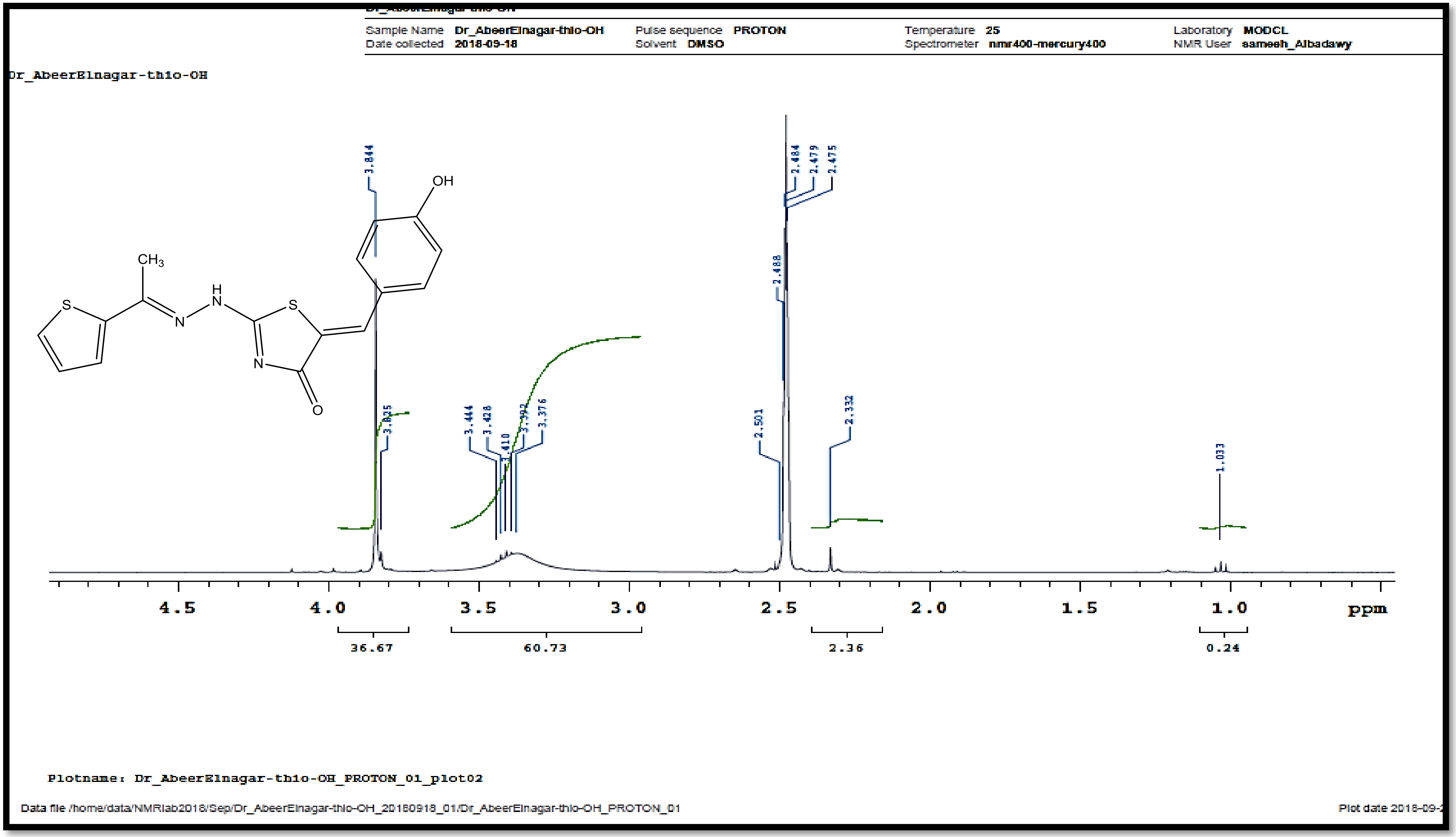

^{1}H NMR of compound (8c)

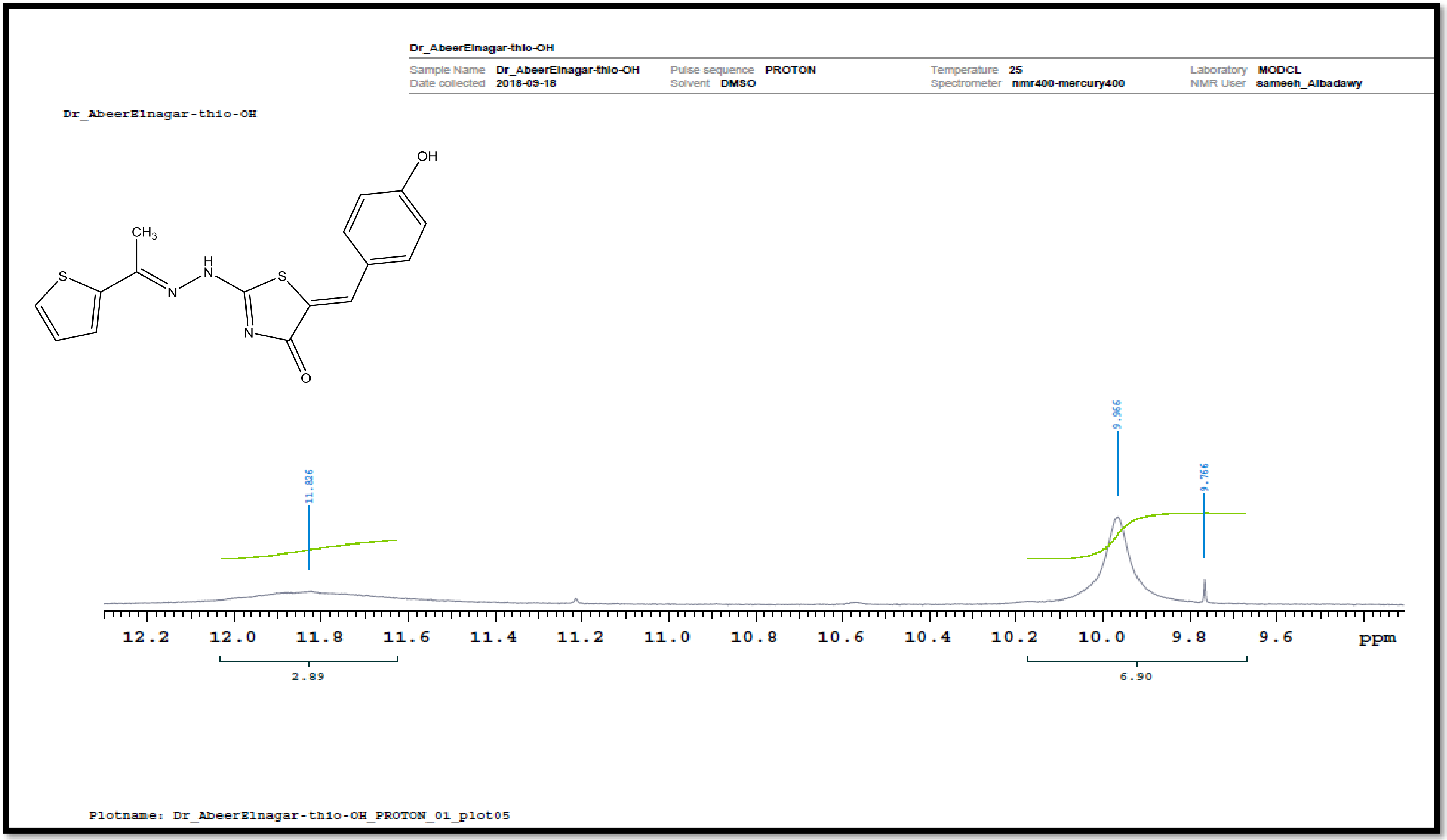

IR of compound (8d)

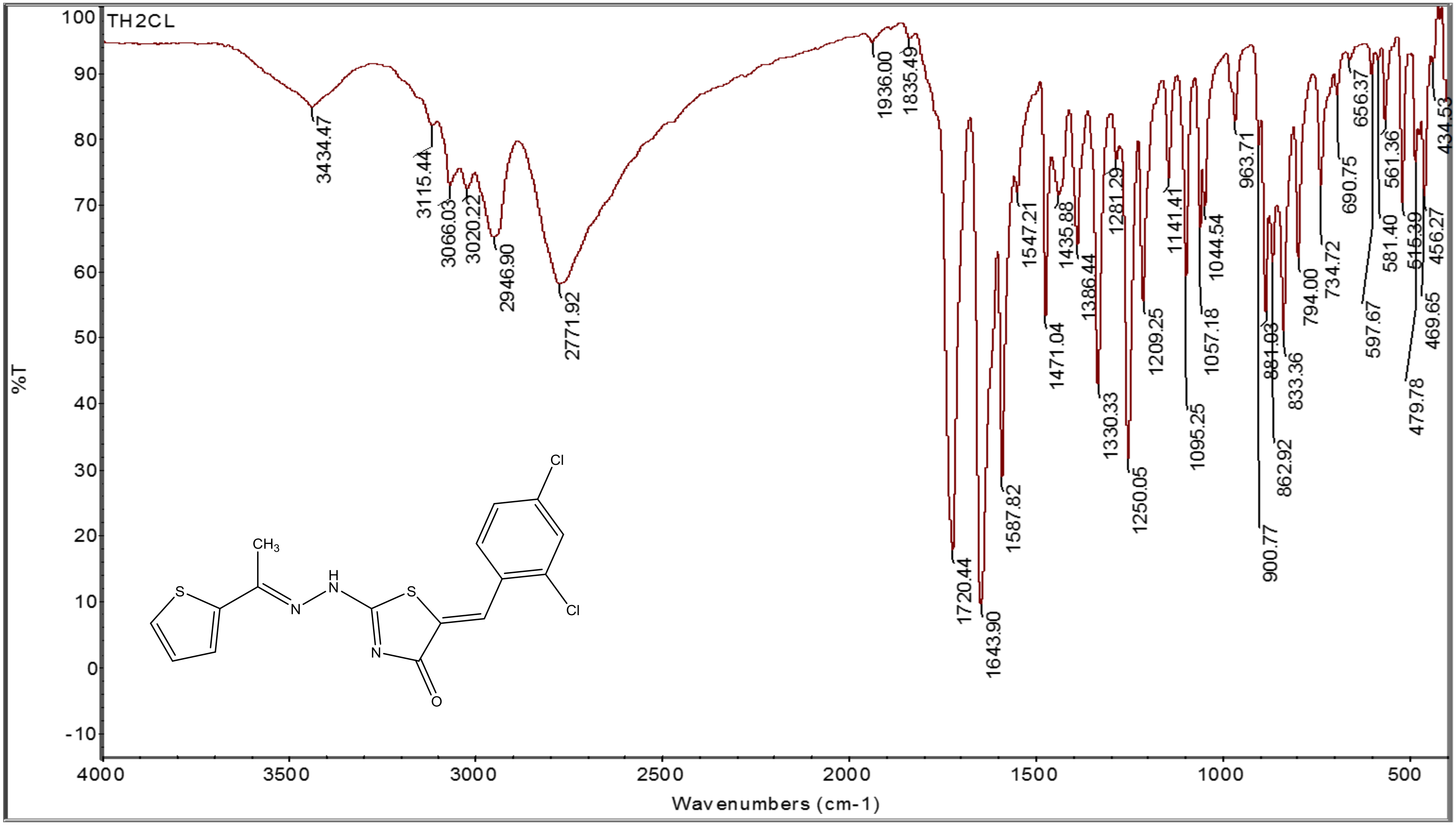

^{1}H NMR of compound (8d)

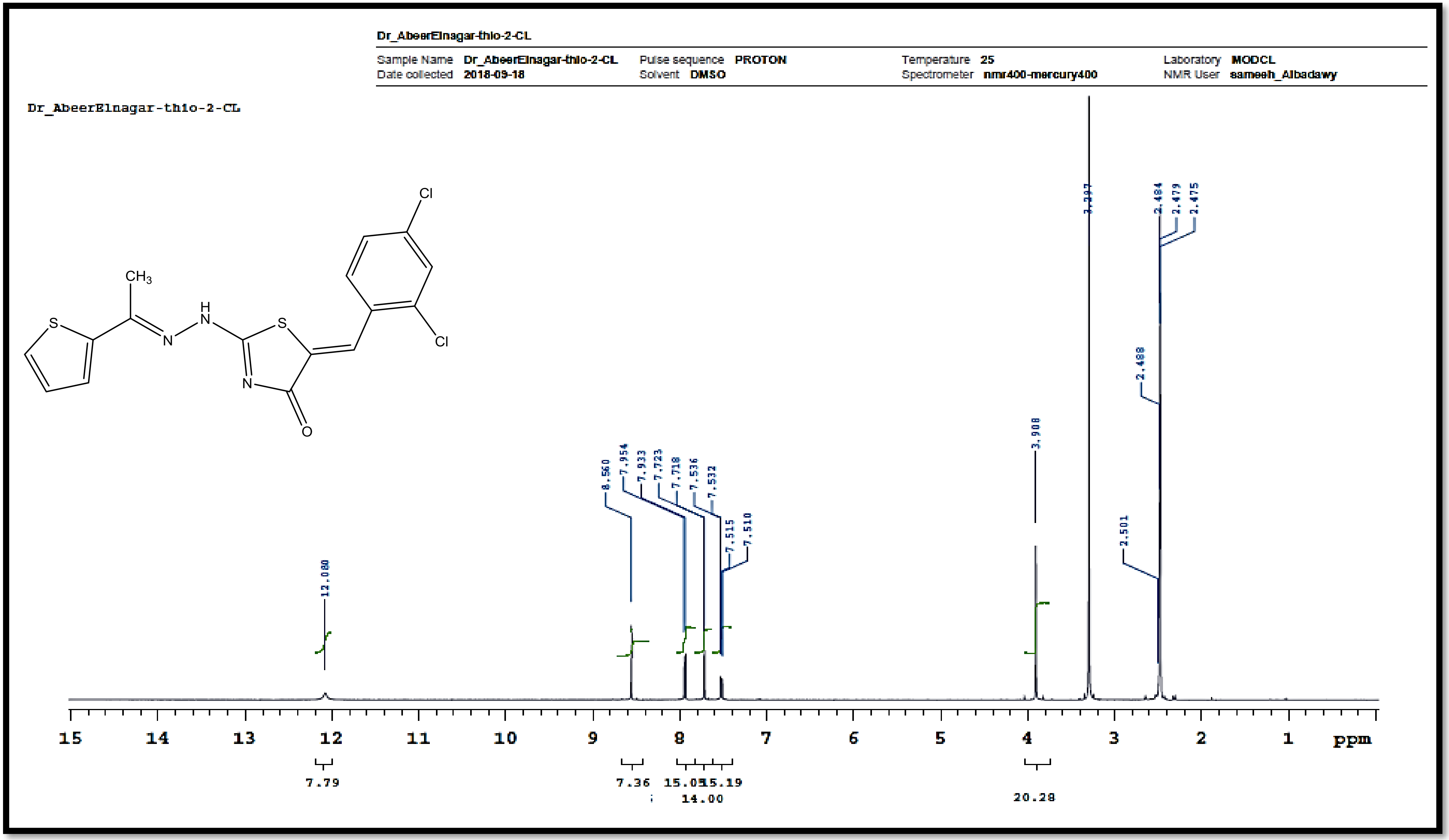

D_2O ^1H NMR of compound (8d)

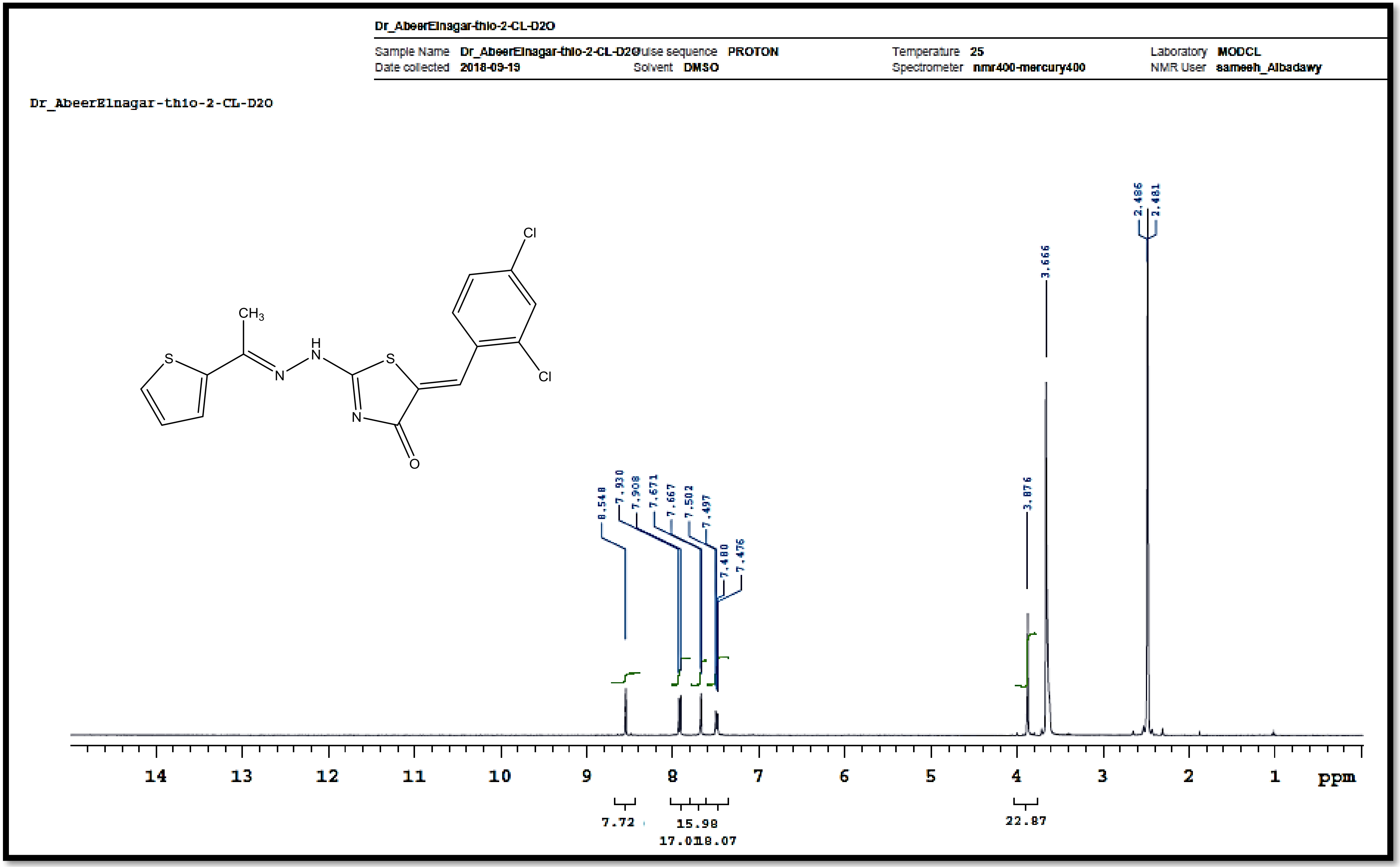

IR of compound (8e)

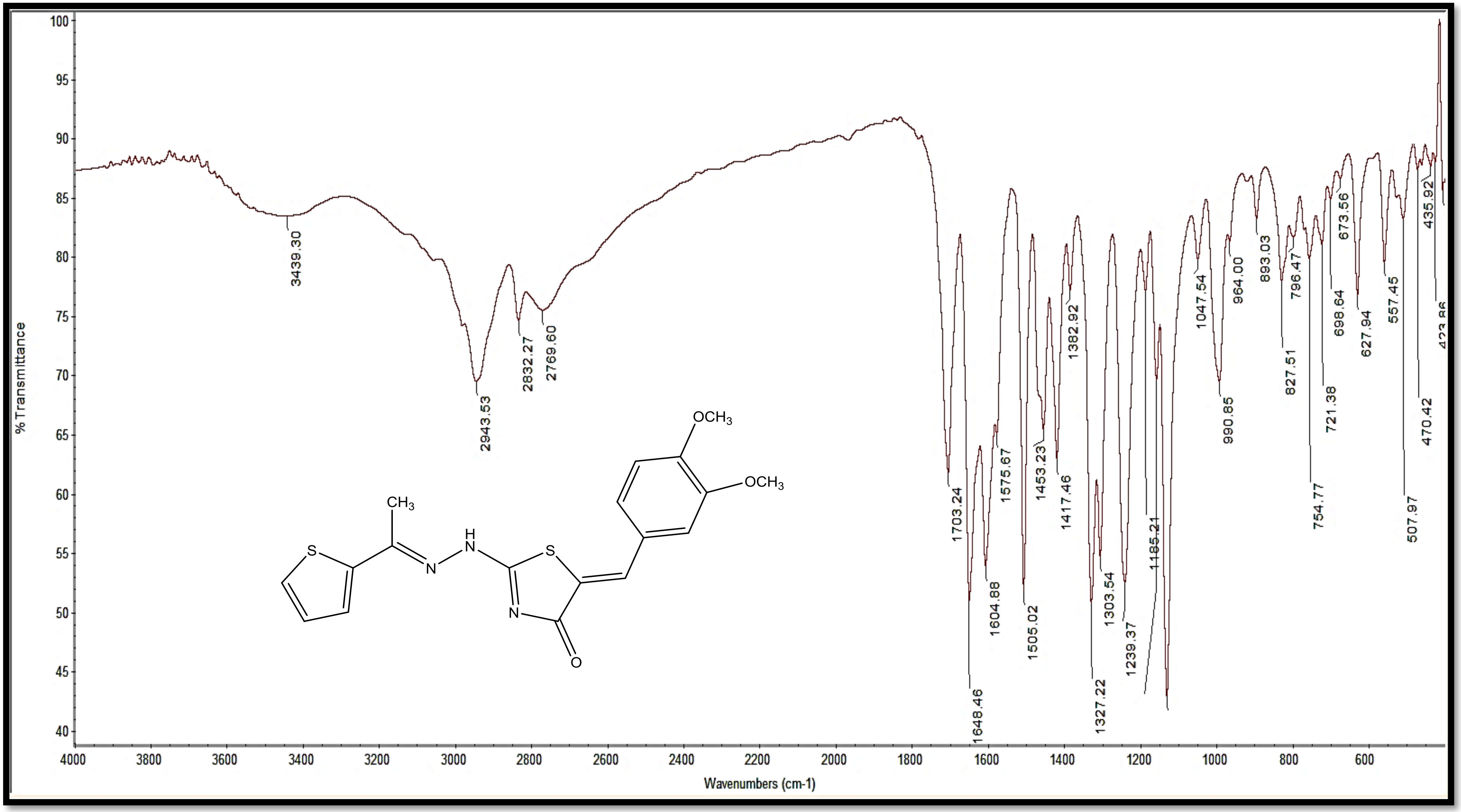

^{1}H NMR of compound (8e)

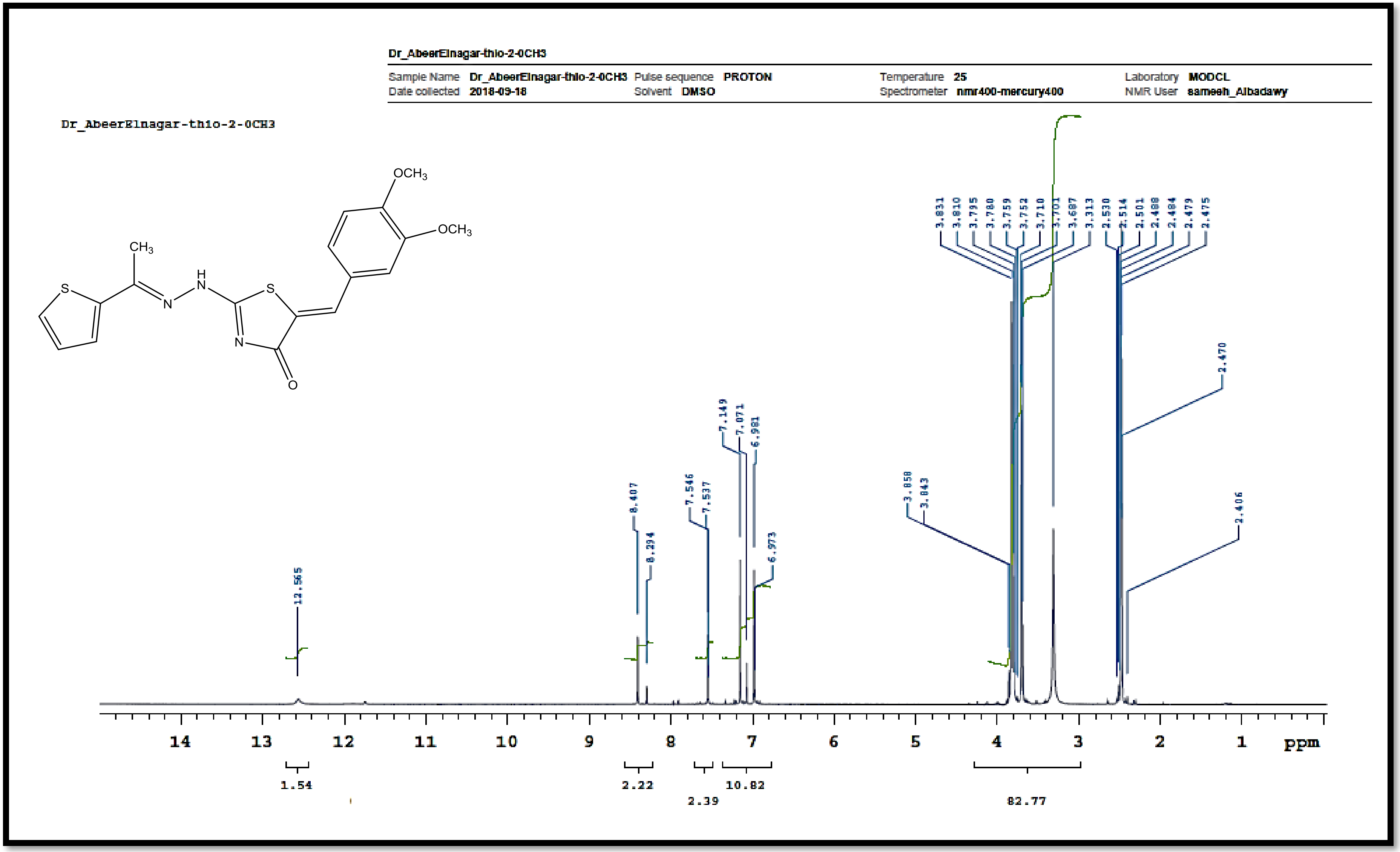

^{1}H NMR of compound (8e)

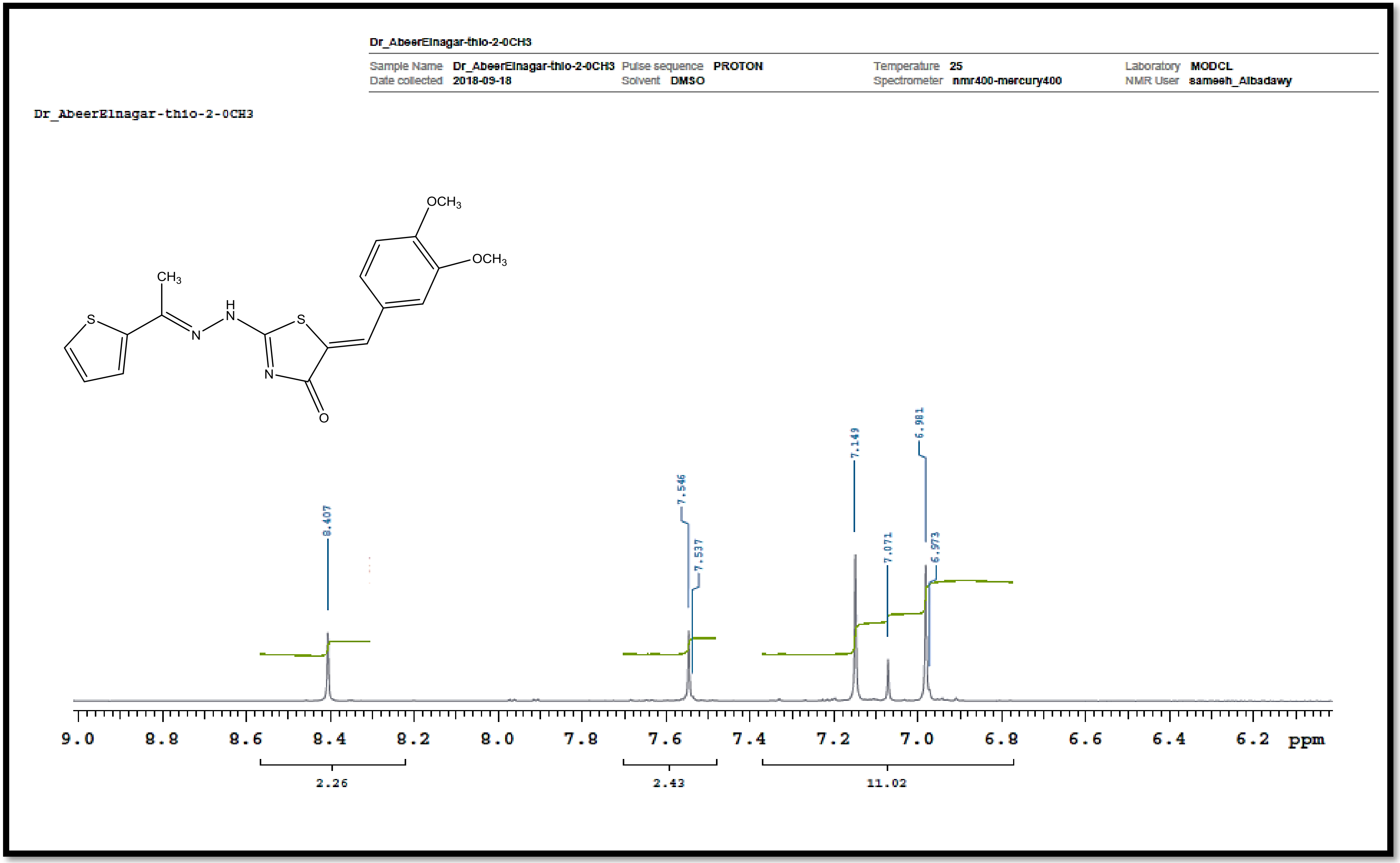

IR of compound (8f)

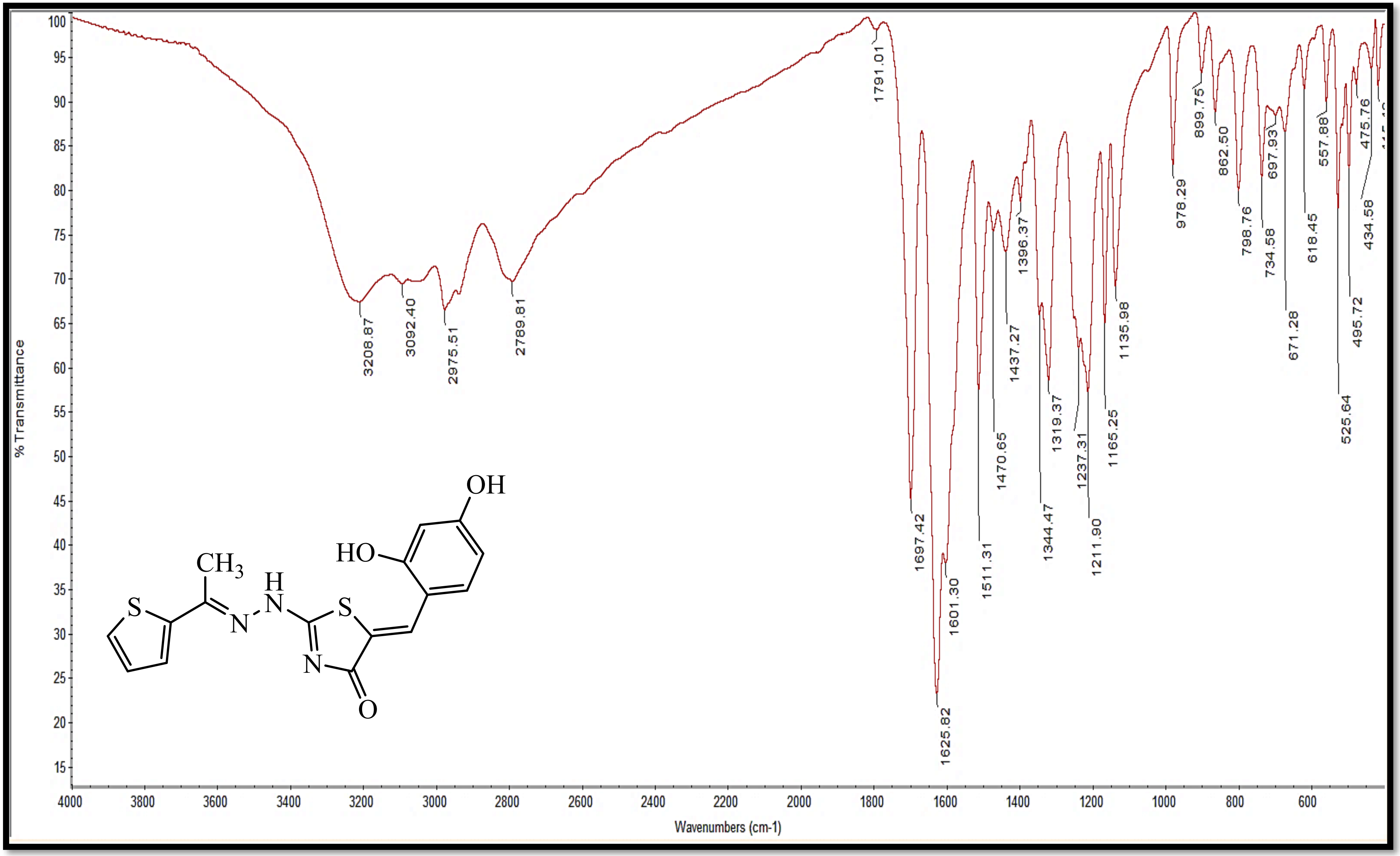

^{1}H NMR of compound (8f)

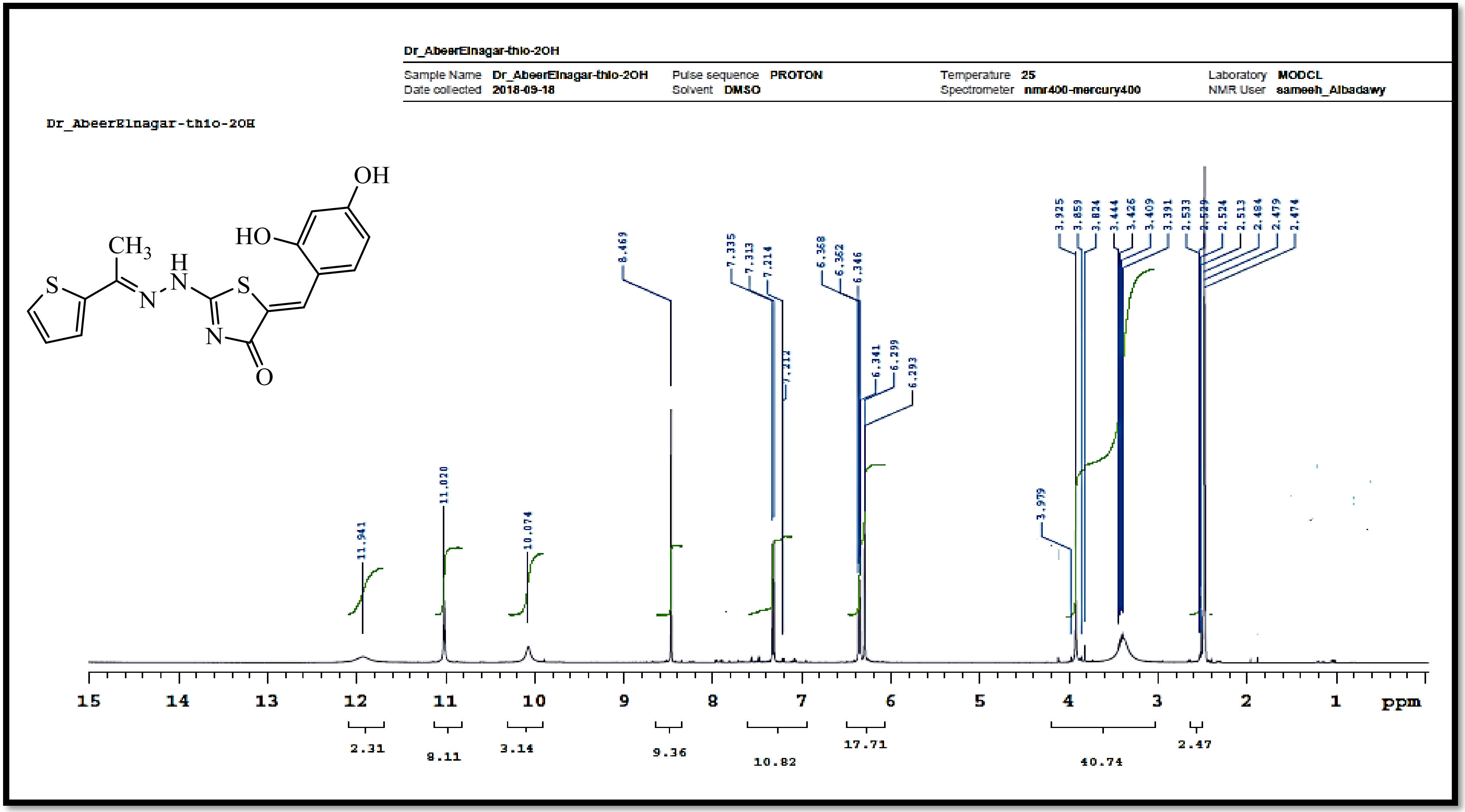

D_2O ^1H NMR of compound (8f)

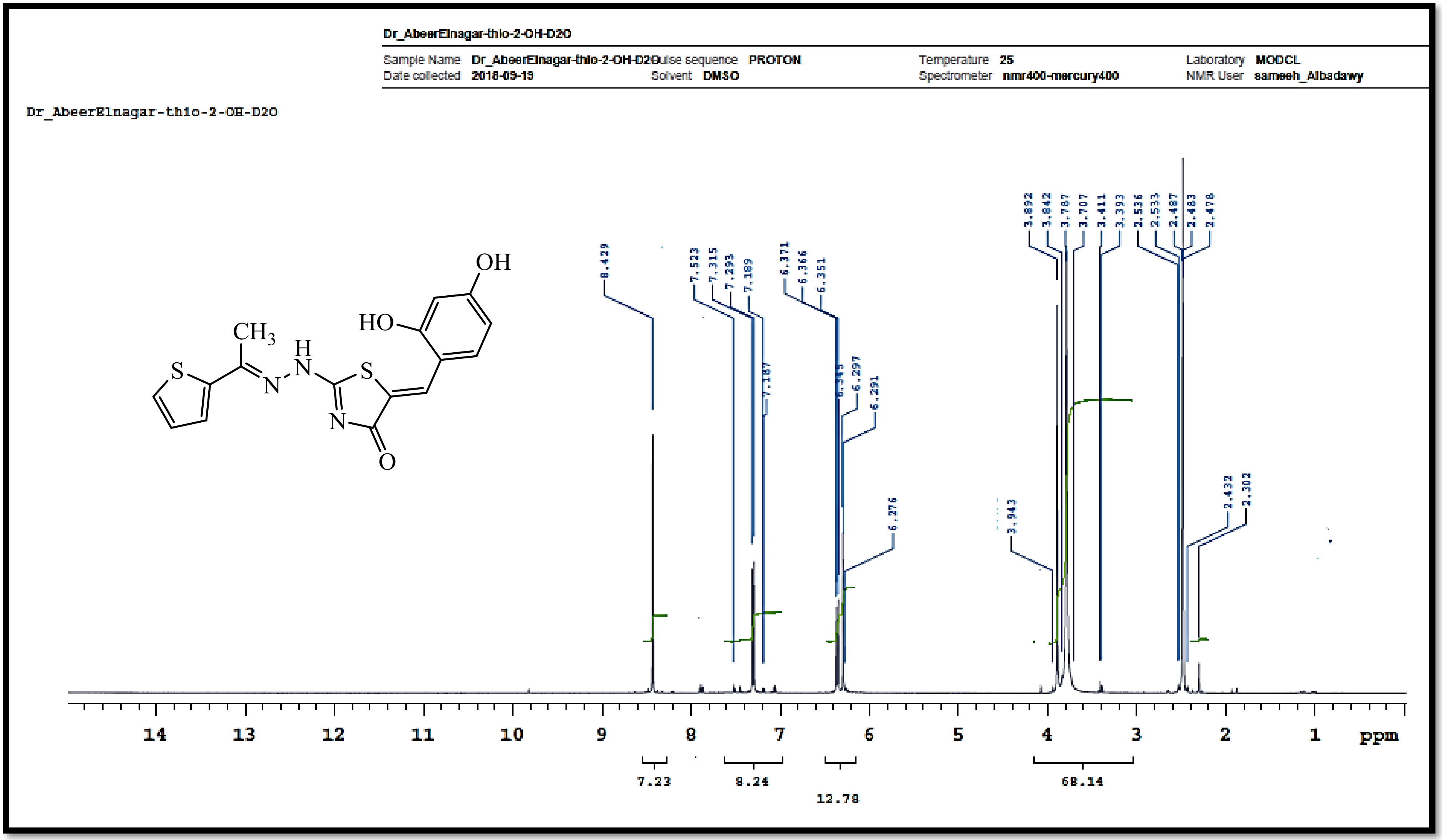

IR of compound (8g)

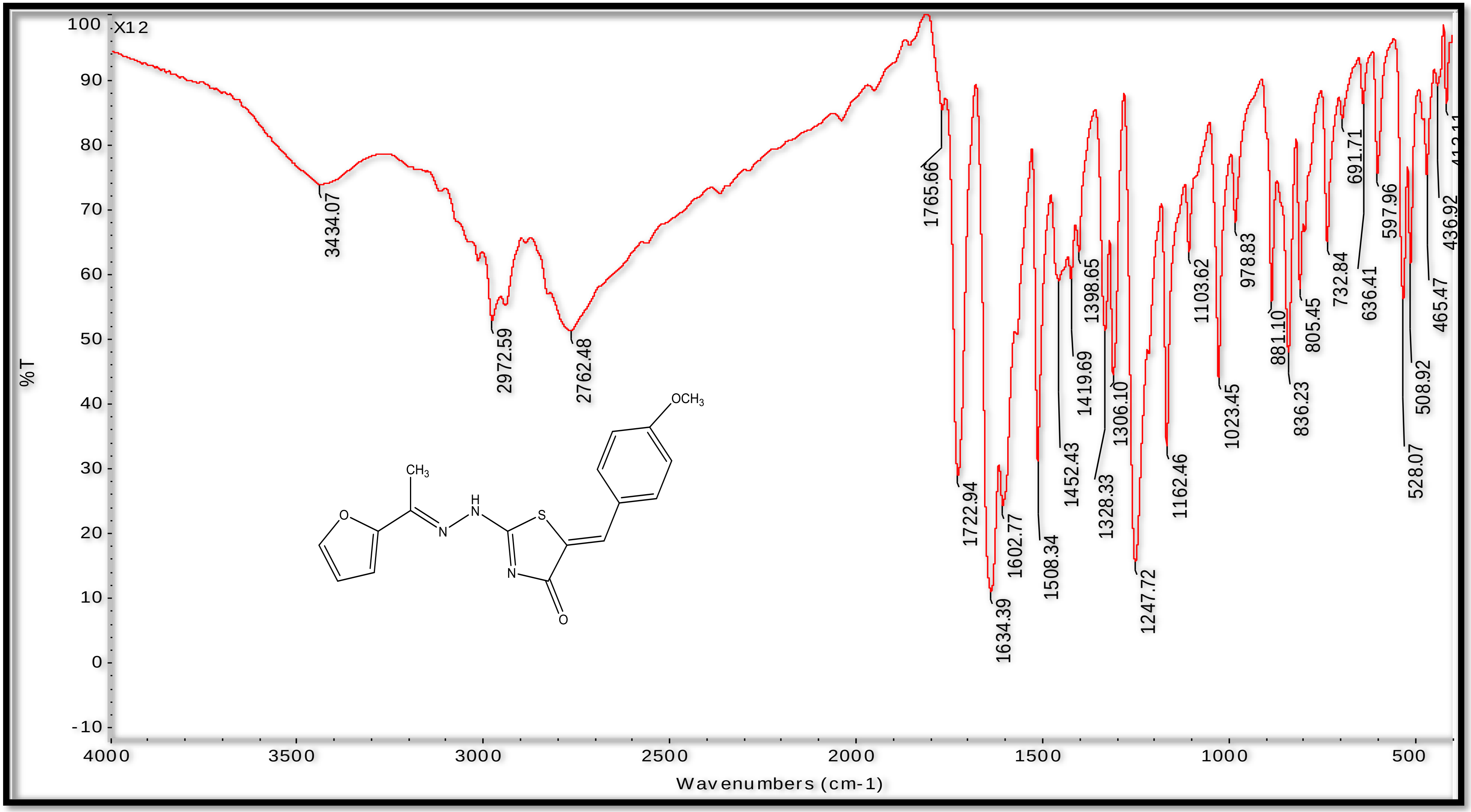

^{1}H NMR of compound (8g)

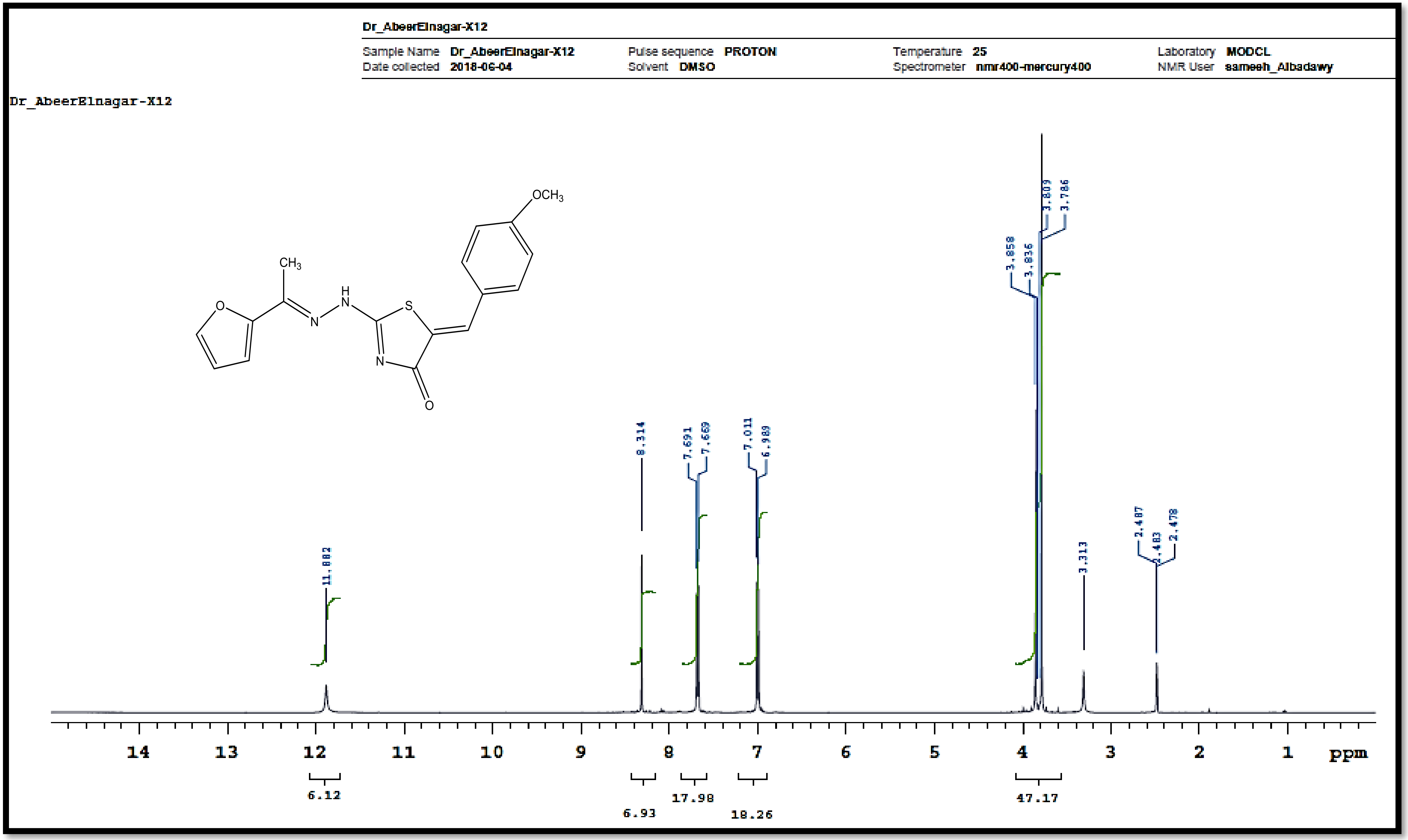

D_2O ^{1}H NMR of compound (8g)

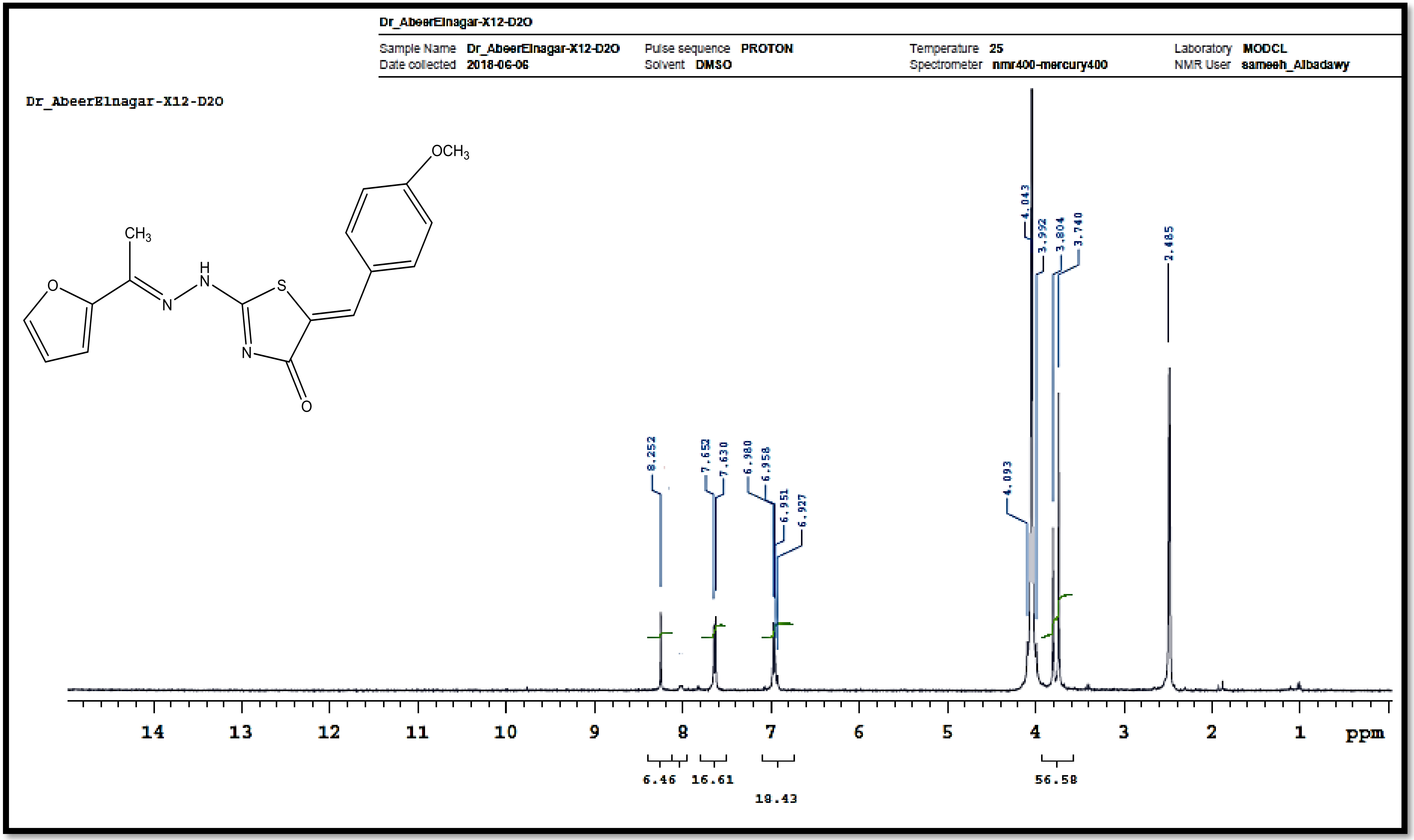

IR of compound (8h)

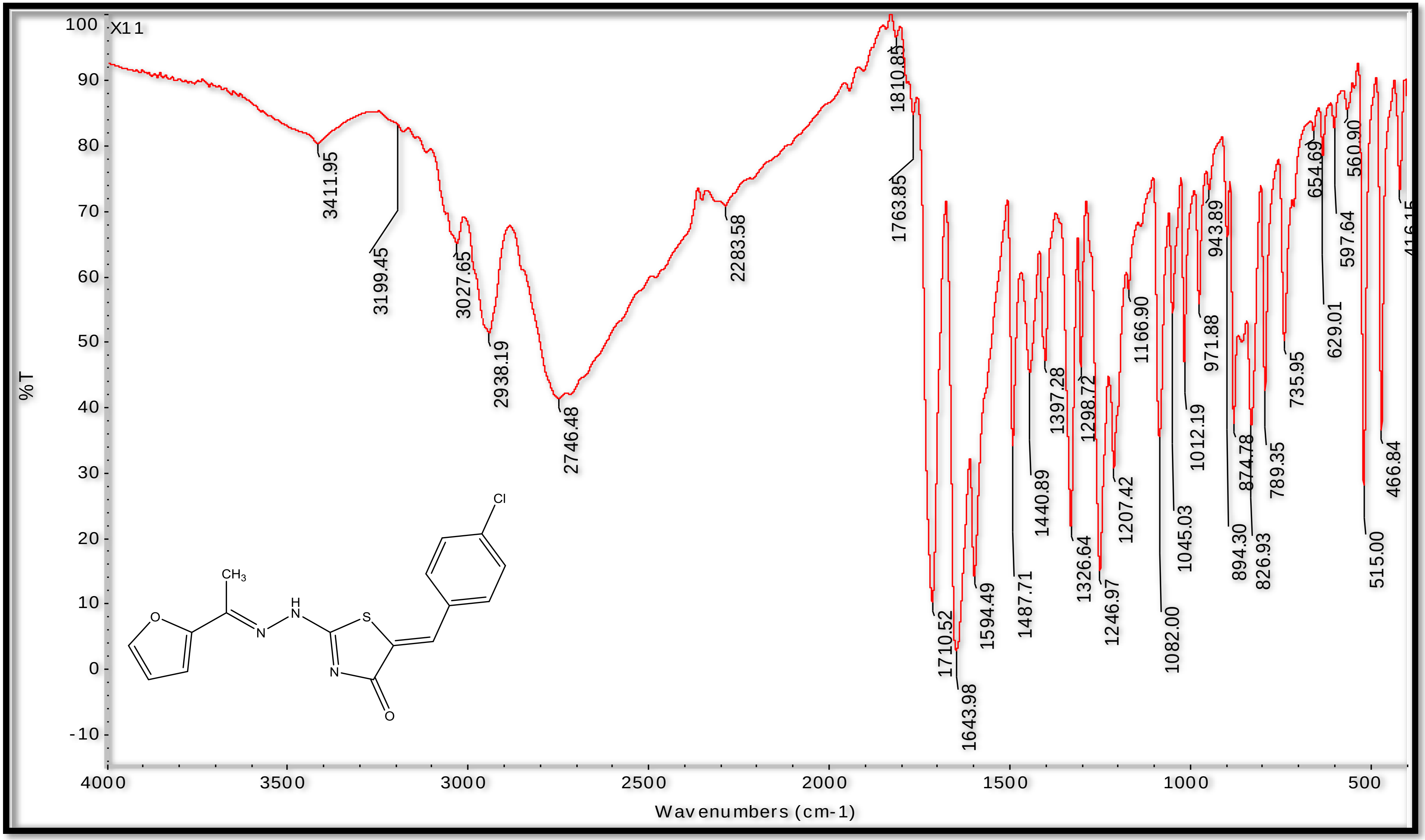

^{1}H NMR of compound (8h)

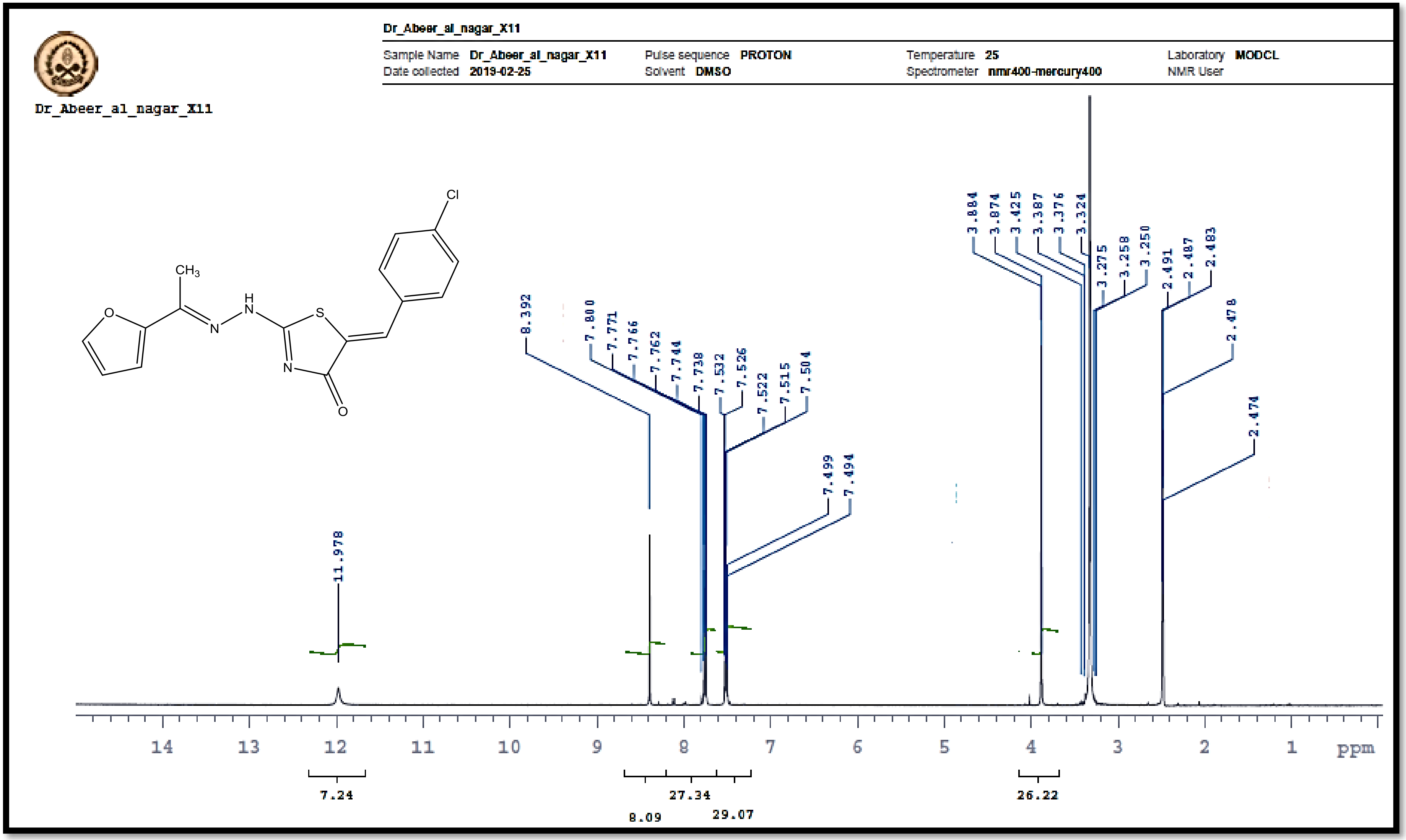

D_2O ^1H NMR of compound (8h)

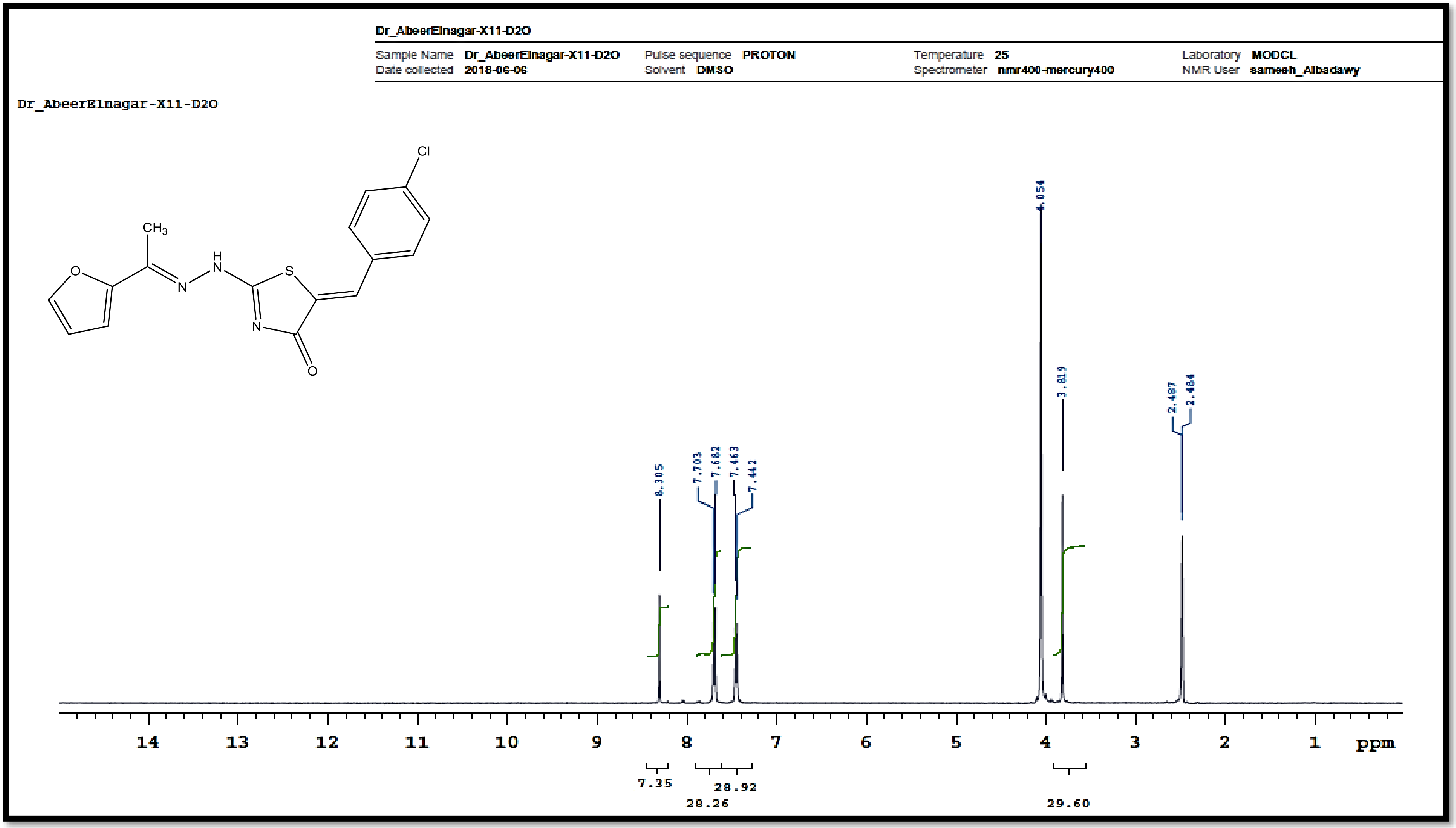

IR of compound (8i)

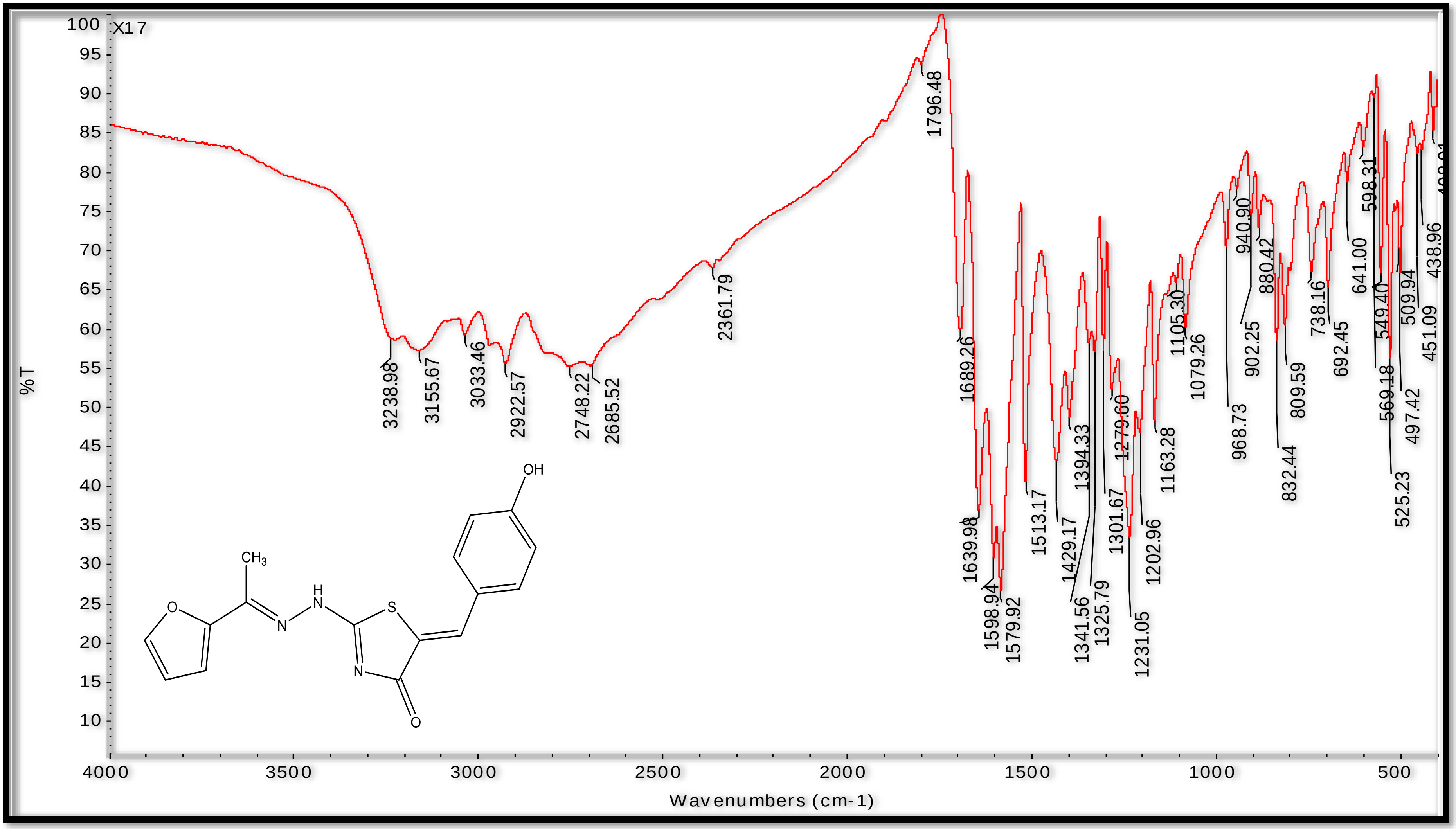

^{1}H NMR of compound (8i)

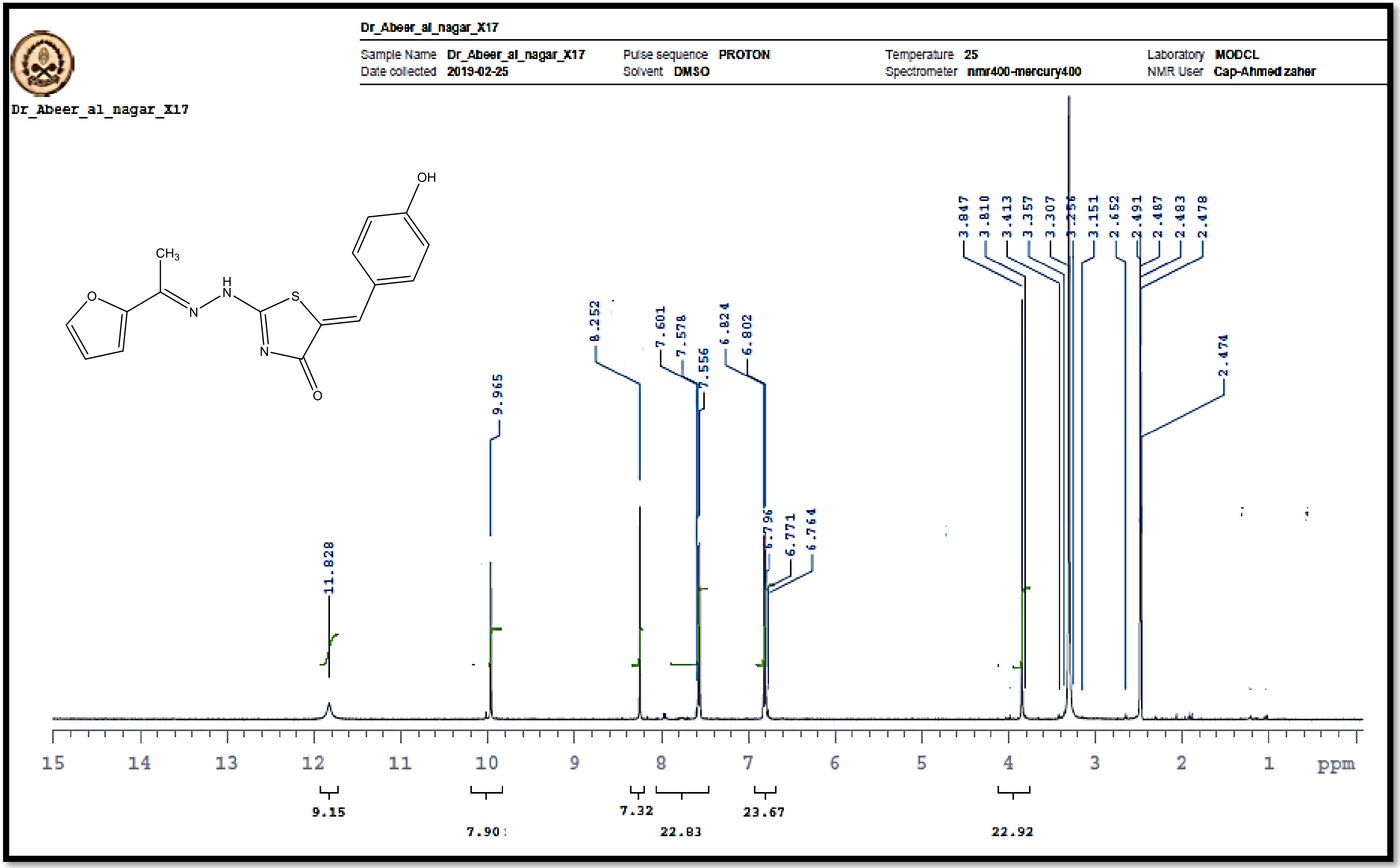

^{1}H NMR of compound (8i)

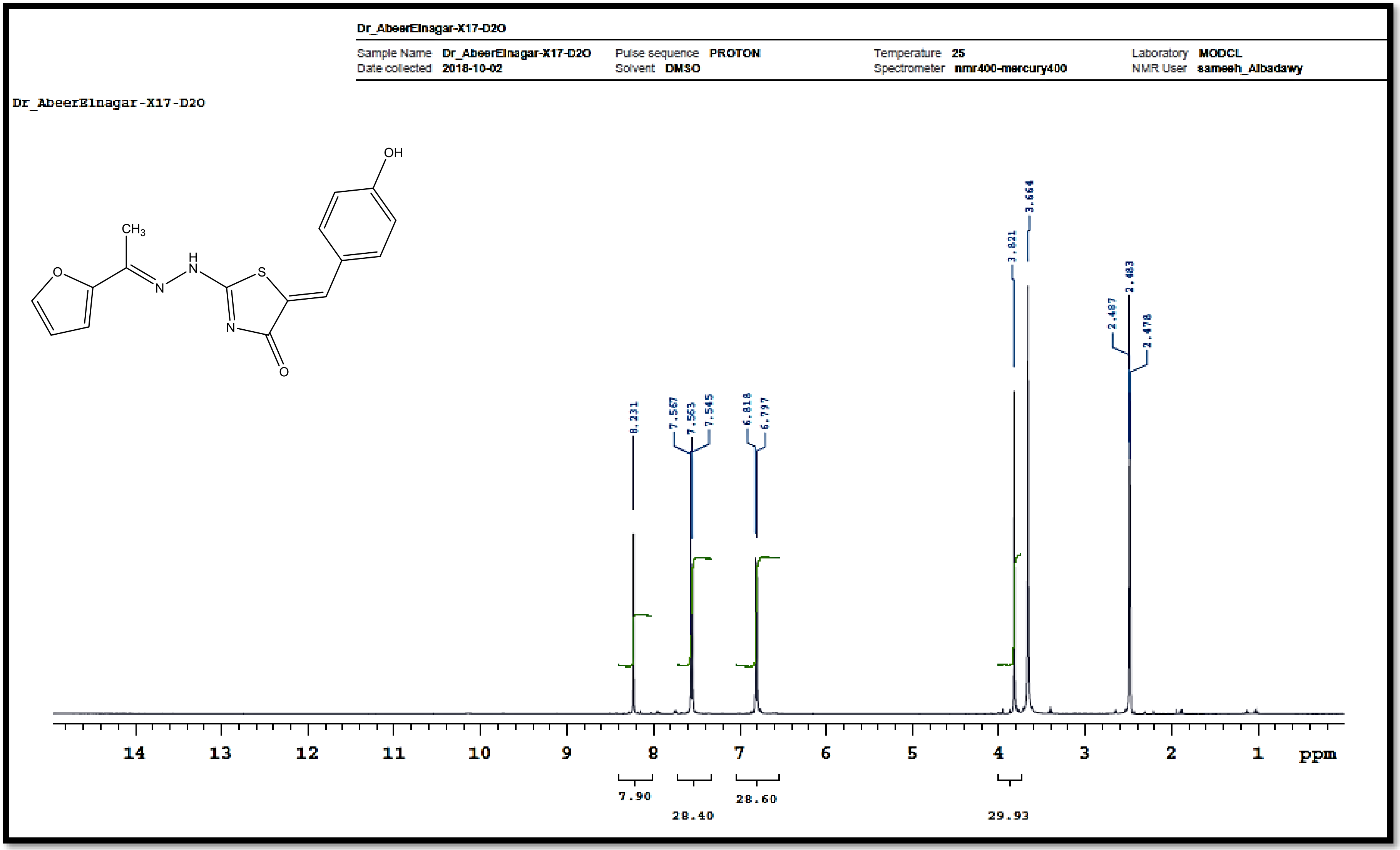

IR of compound (8j)

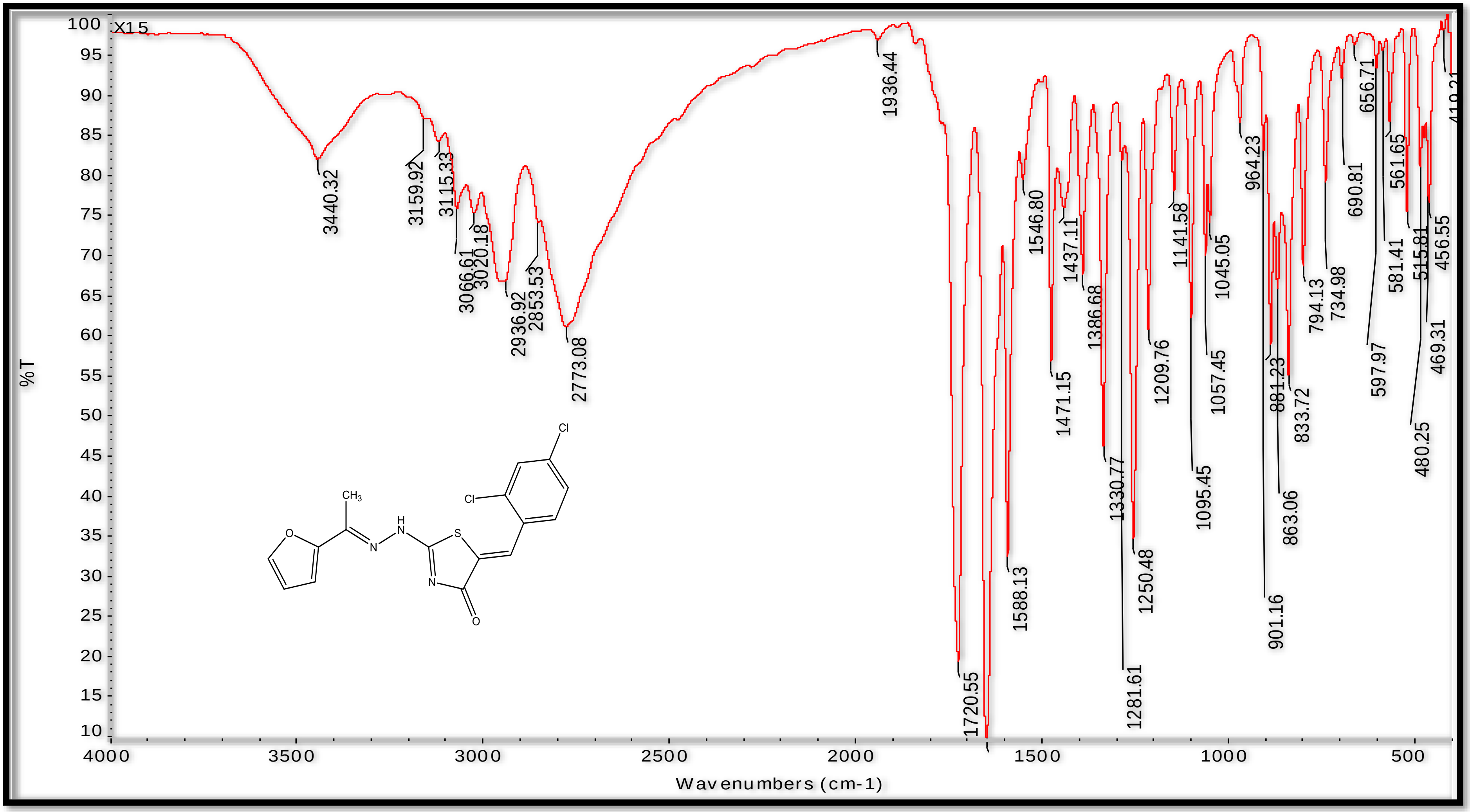

^{1}H NMR of compound (8j)

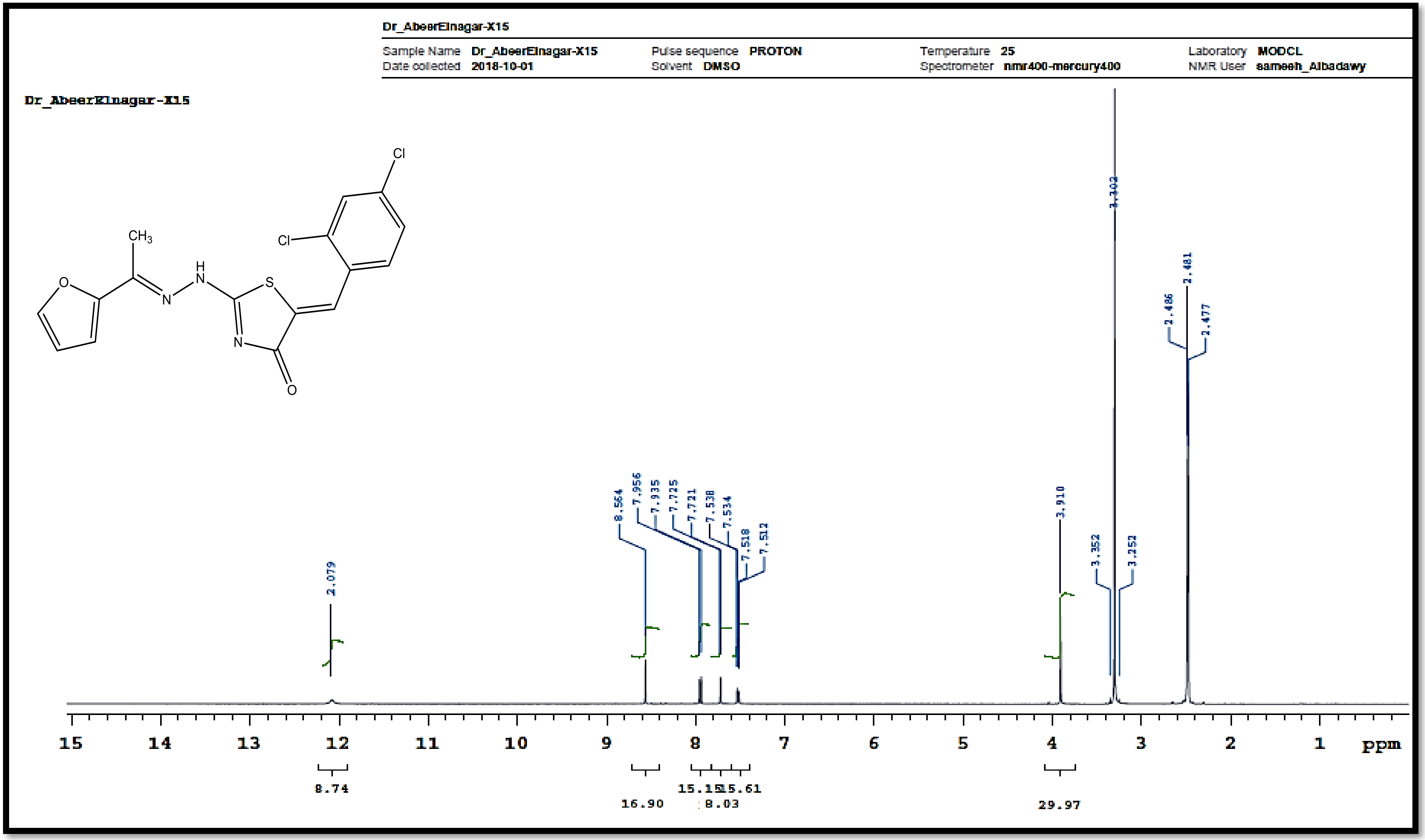

IR of compound (8k)

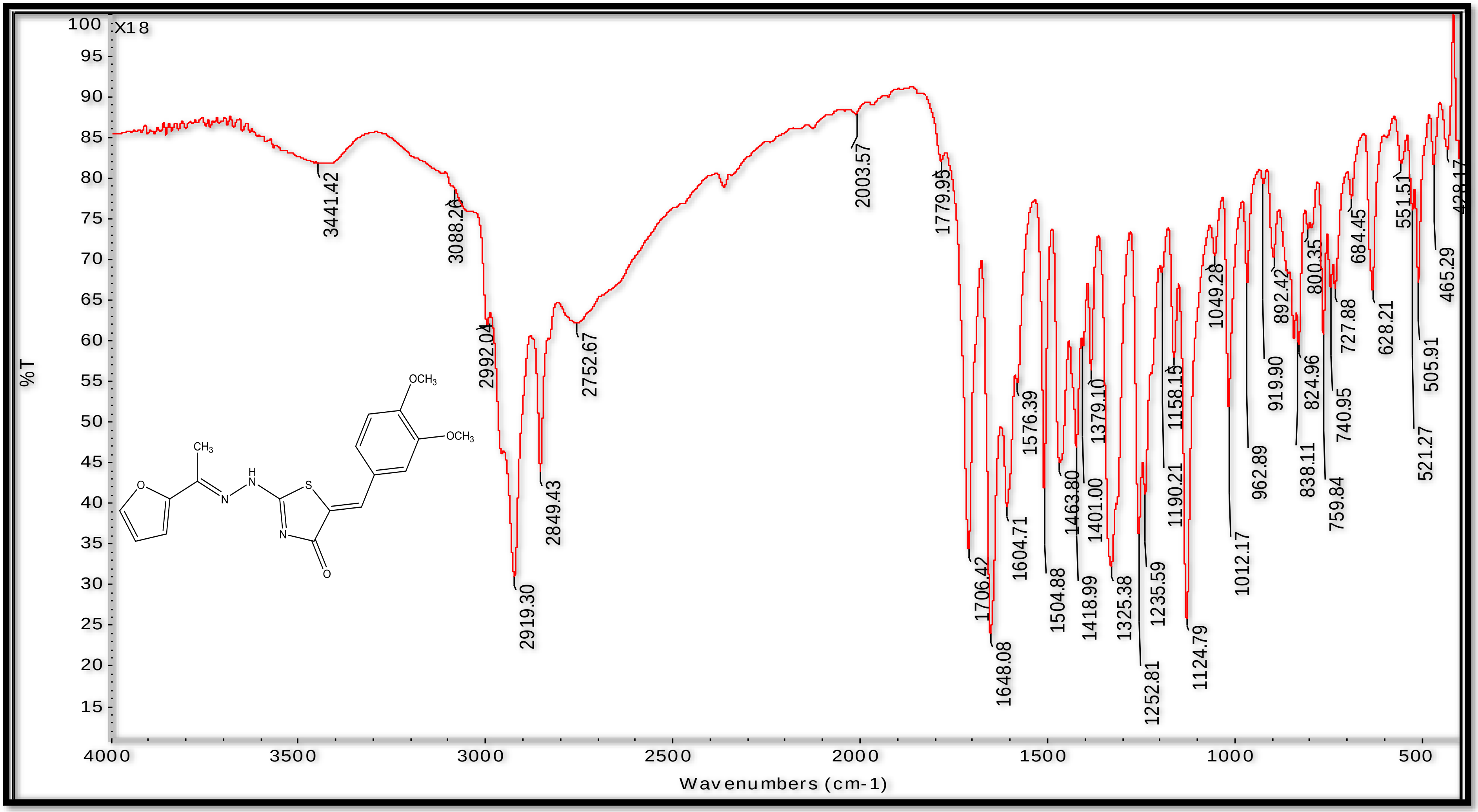

^{1}H NMR of compound (8k)

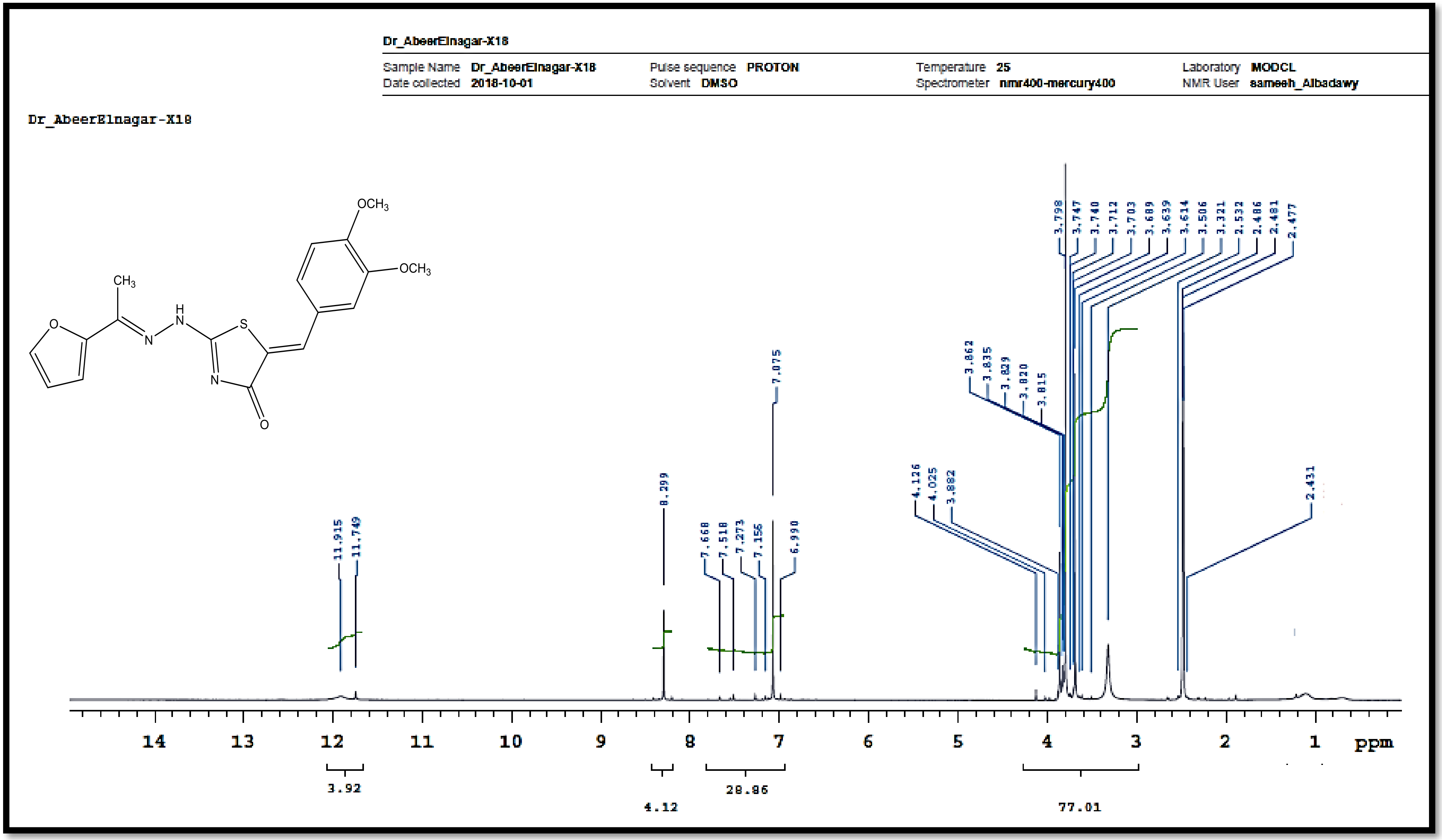

IR of compound (8l)

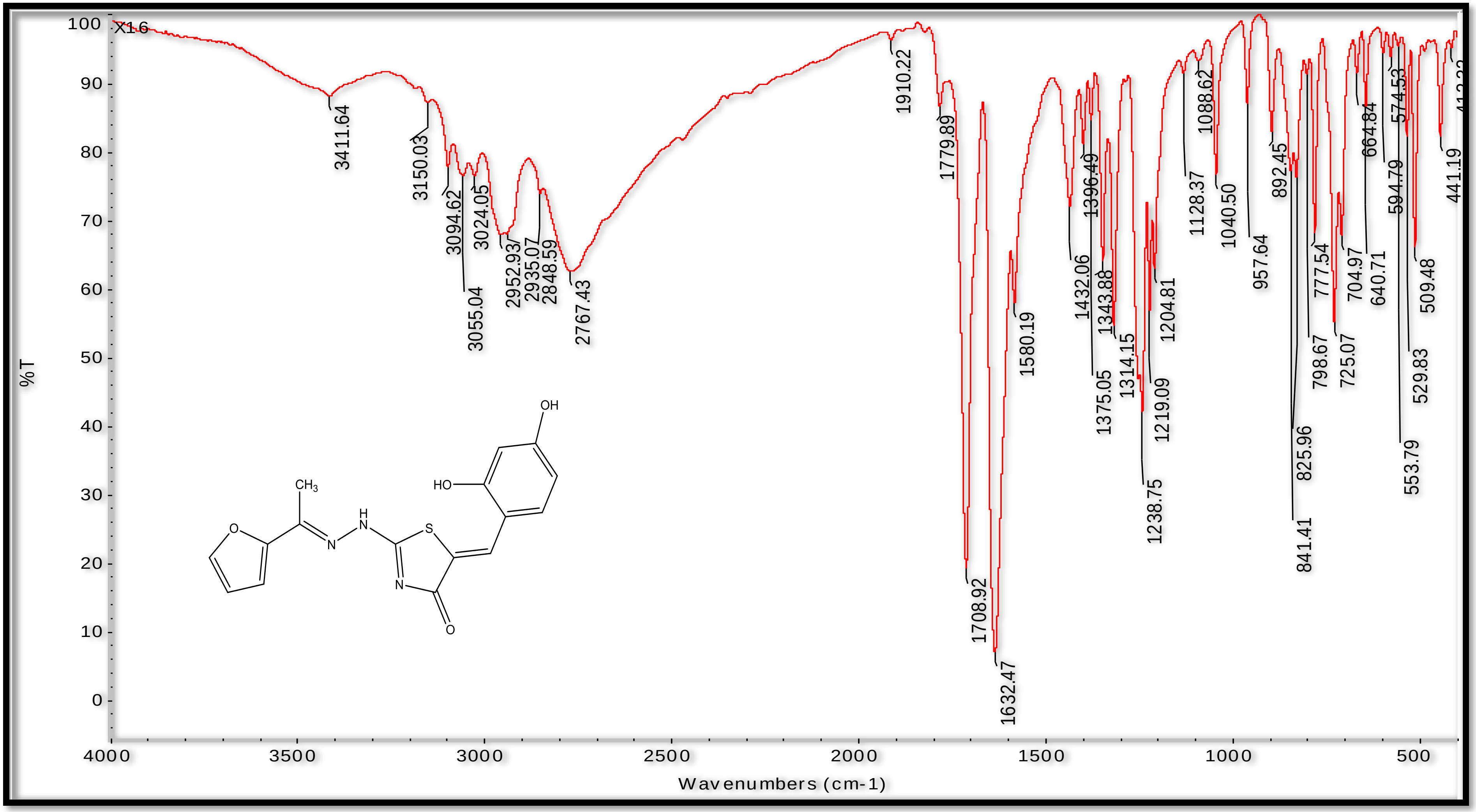

^{1}H NMR of compound (8l)

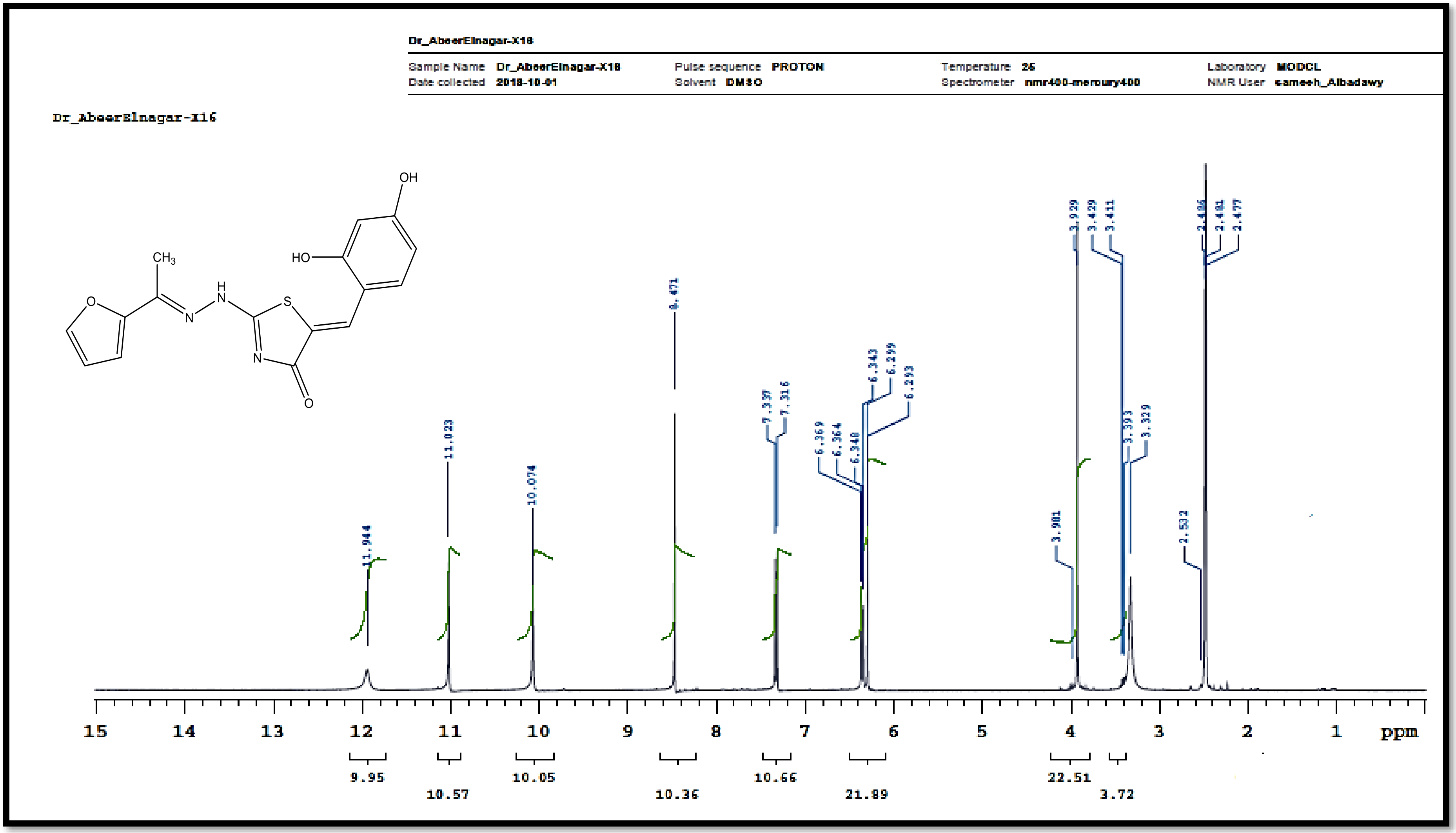

D$_2$O ^{1}H NMR of compound (8l)

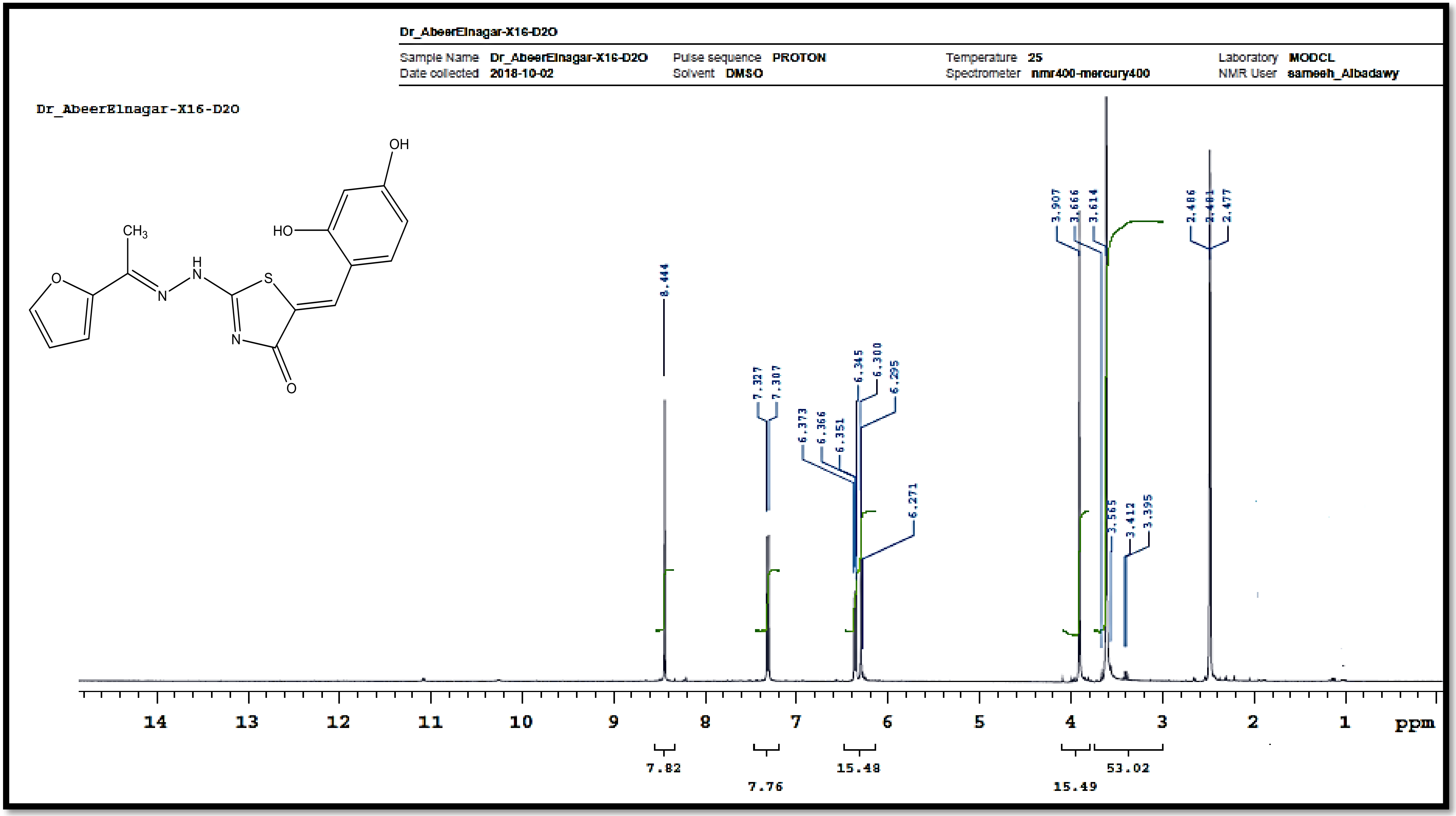

IR of compound (9a)

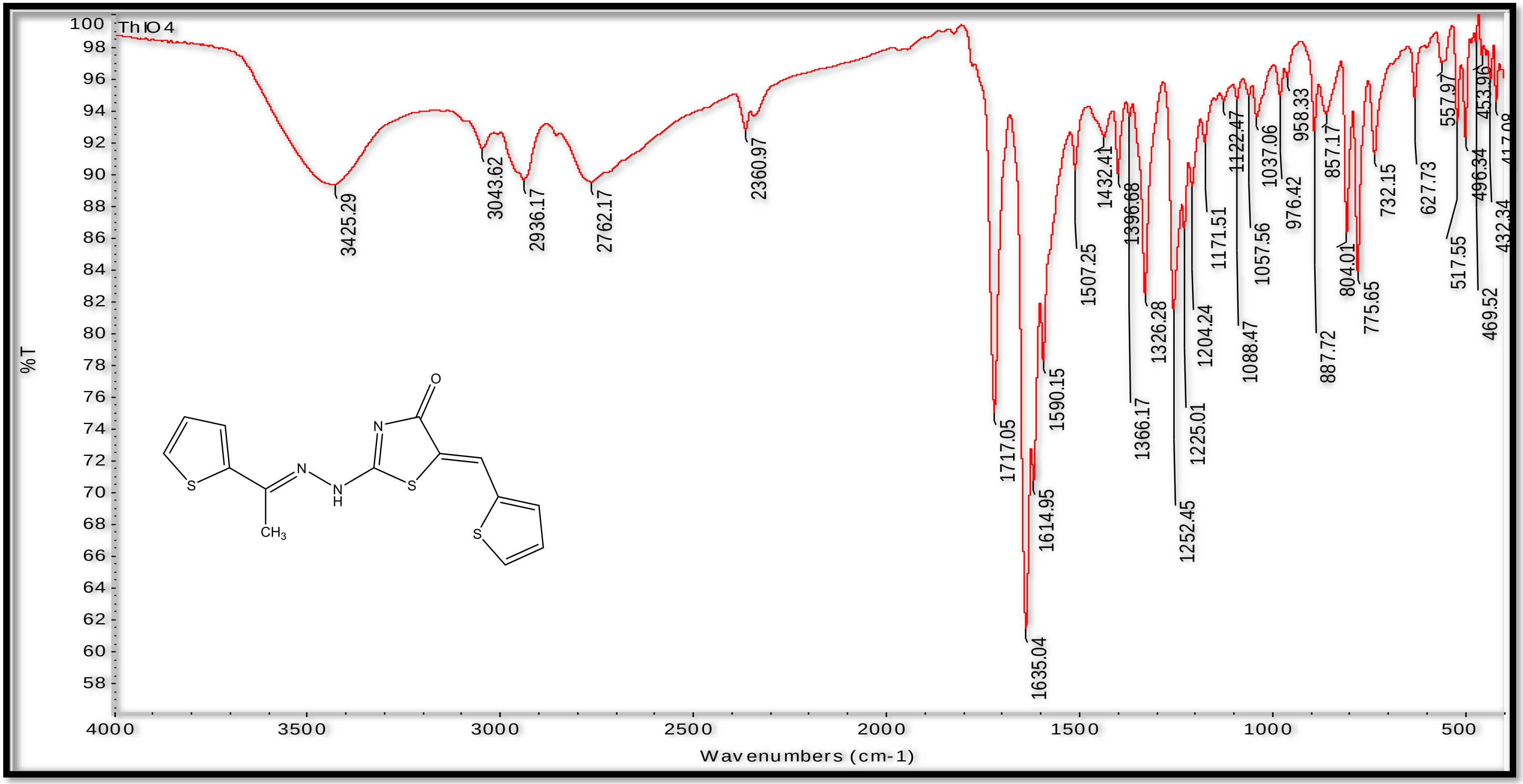

^{1}H NMR of compound (9a)

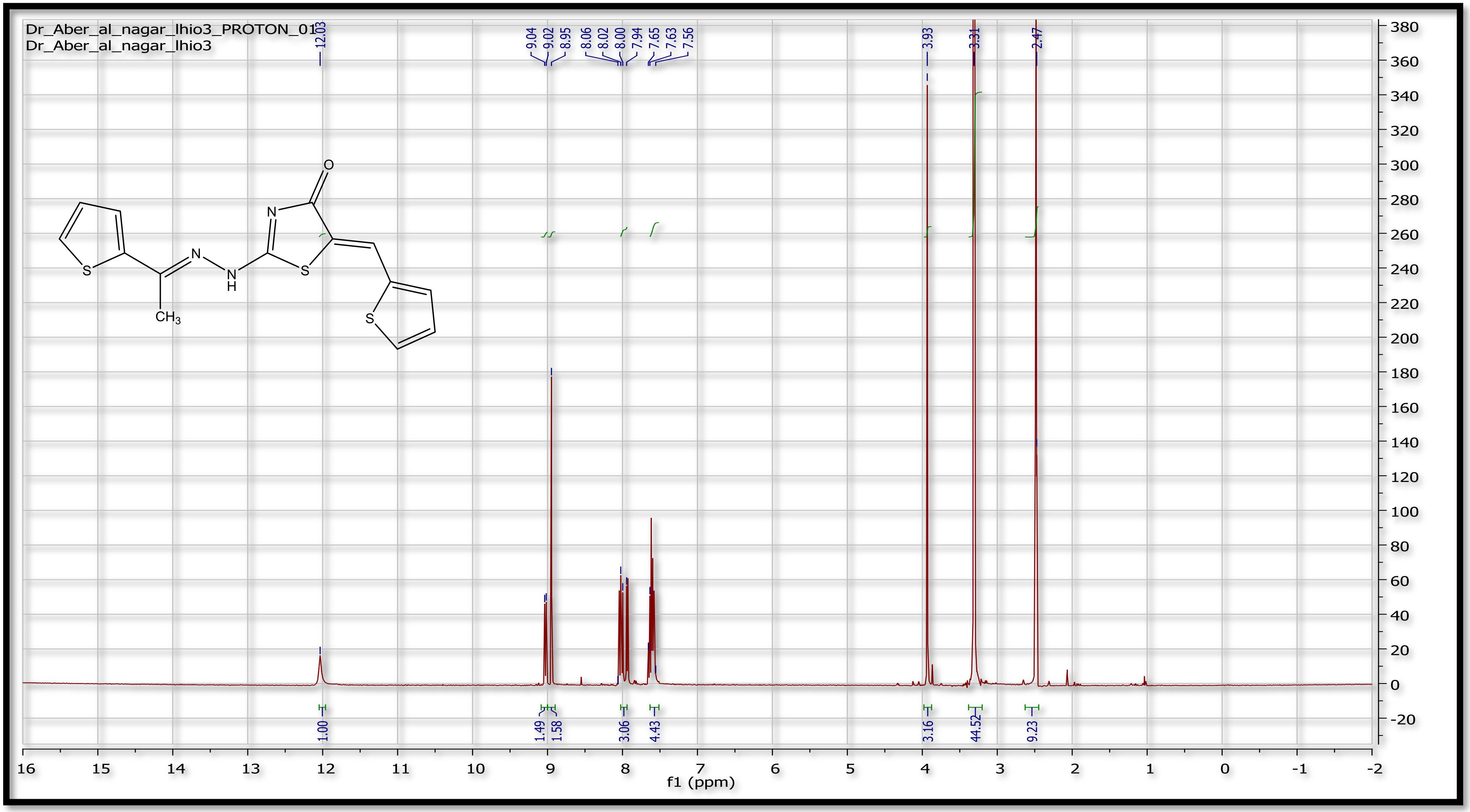

IR of compound (9b)

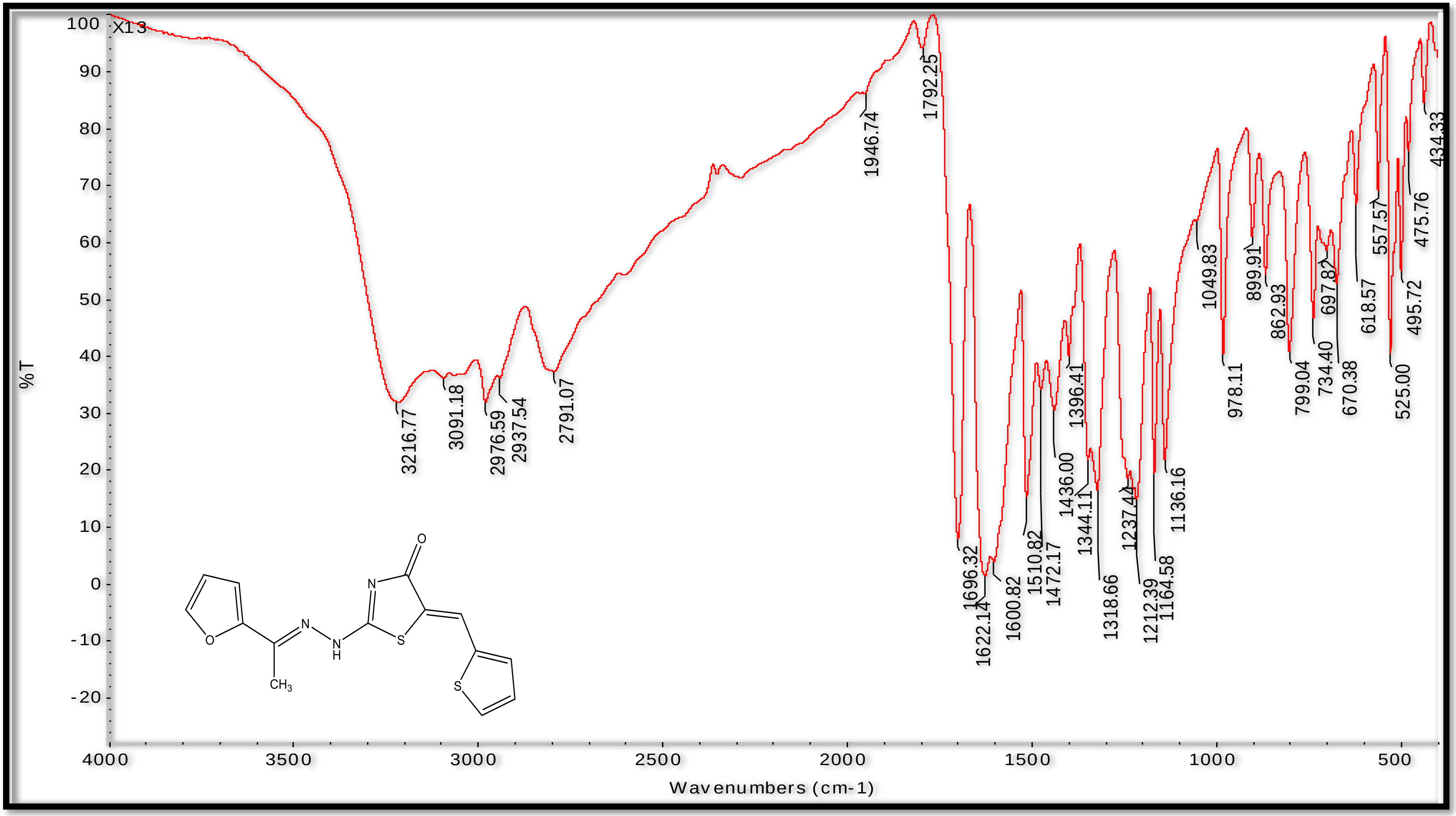

^{1}H NMR of compound (9b)

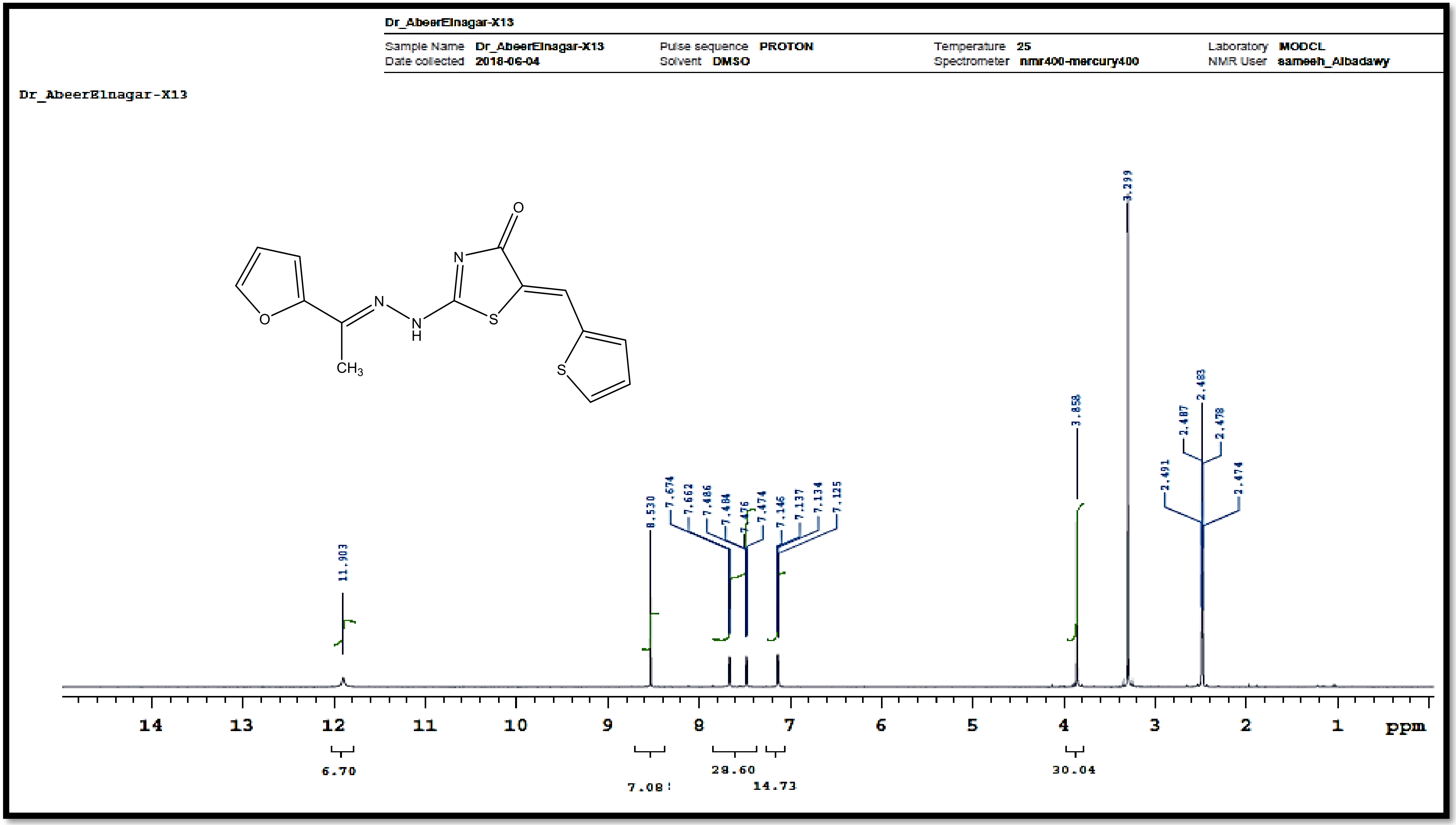

^{1}H NMR of compound (9b)

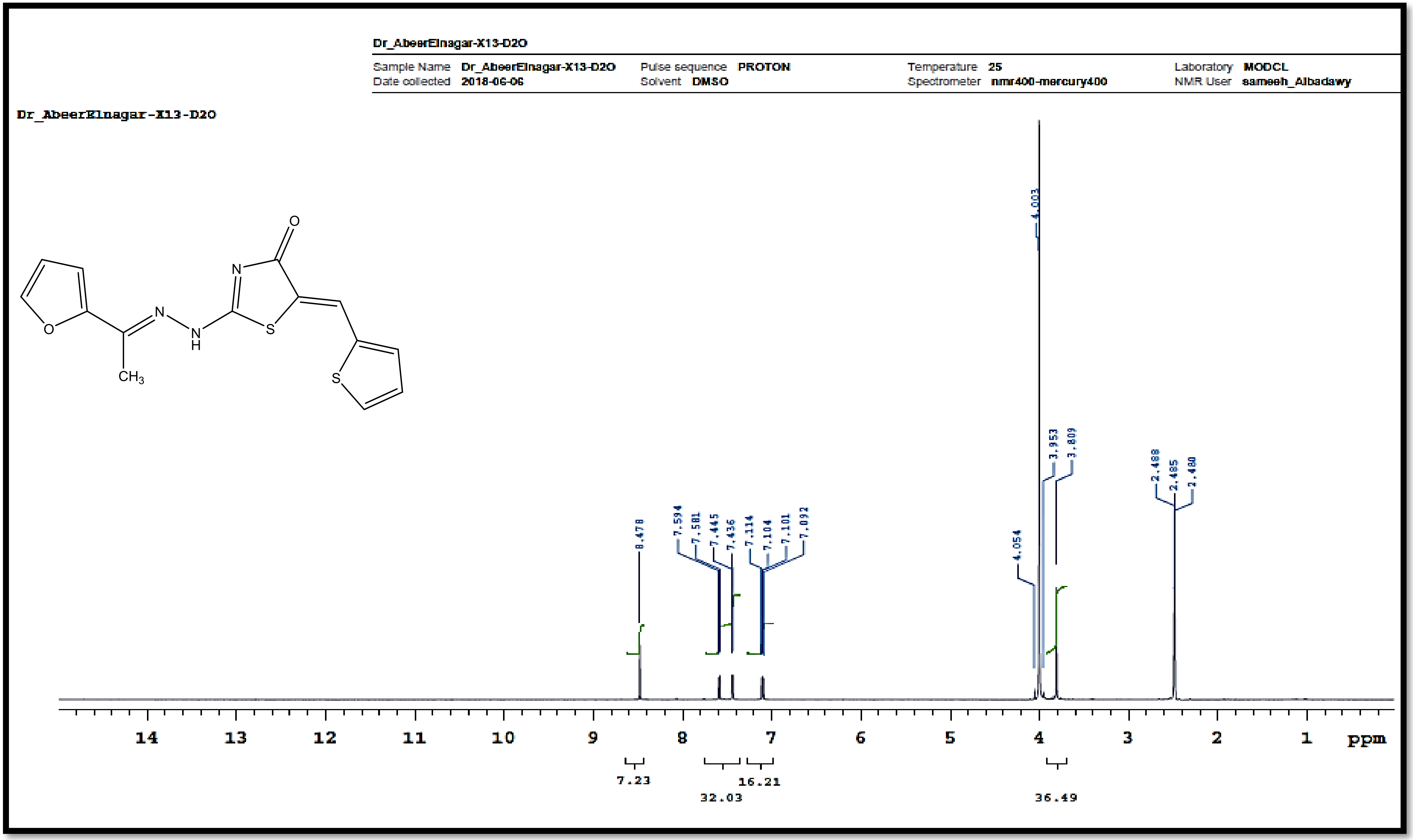

Supplement: RA-010-C9RA10094F-s001 [file RA-010-C9RA10094F-s001.pdf]
